# Stereospecific Radical Bromination of *β*‐Aryl Alcohols with Thiourea Additives Through A Serendipitous Discovery of A 1,2‐Aryl Migration

**DOI:** 10.1002/chem.202403831

**Published:** 2025-01-15

**Authors:** Habib Assy, Uttam K. Mishra, Tom Rösler, Raman Khurana, N. Gabriel Lemcoff, Ofer Reany

**Affiliations:** ^1^ Department of Chemistry Ben-Gurion University of the Negev Beer-Sheva 8410501 Israel; ^2^ Department of Natural Sciences The Open University of Israel Ra'anana 4353701 Israel.; ^3^ Ilse Katz Institute for Nanoscale Science and Technology Ben-Gurion University of the Negev Beer-Sheva 8410501 Israel.

**Keywords:** Alcohols, Thiourea, Halogenation, Stereoselectivity, Radical aryl migration

## Abstract

The development of new protocols for stereospecific and stereoselective halogenation transformations by mild reaction conditions is a highly desirable research target for the chemical and pharmaceutical industries. Following the straightforward methodology for directly transforming a wide scope of alcohols to alkyl bromides and chlorides using substoichiometric amounts of thioureas and *N*‐halo succinimides (NXS) as a halogen source in a single step, we noticed that in apolar solvents bromination of chiral secondary alcohols did not produce the expected racemates. In this study, the stereochemical aspects of the bromination reaction were examined. Surprisingly, bromination of (±)‐*threo*‐ or (±)‐*erythro*‐3‐phenyl‐2‐butanols revealed a single diastereomeric brominated product with retention of configuration. The scope of these reactions was expanded on several β‐aryl alcohols. During these studies, an unexpected stereospecific 1,2‐migration of the phenyl group was shown to take place. The proposed mechanism of the 1,2‐phenyl migration involves the formation of a spiro[2,5]octadienyl radical, which is then attacked by a bromide radical at any of the two cyclopropyl positions anti to the phenyl position, leading to products that retain the stereoisomeric configuration of the starting material.

## Graphical abstract

In this study, the bromination of diastereomeric *β*‐aryl alcohols using the *N*‐bromo‐succinimide (NBS) and thiourea protocol provides a novel pathway to a direct fully stereoretentive bromination of *β*‐aryl alcohols under very mild conditions.

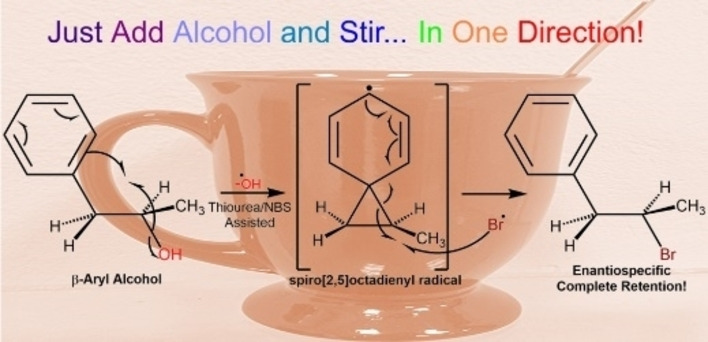

## Introduction

Halogen‐containing organic molecules have received great attention as they are important intermediates used in the chemical and pharmaceutical industries.[^1^, ^2^] Nearly 5000 halogenated compounds are known to appear as natural products, and approximately half of these compounds have a C(sp^3^)‐halogen bond.^[3]^ Many of these isolated halogenated natural products show unique bioactivity and potential medical benefits as drug candidates.^[1]^ Moreover, the stereochemistry of the halogen‐bearing carbons can significantly alter bioactivity and affect the pharmacokinetic properties of the product. Therefore, stereocontrolled halogenation in drug leads is a common strategy for generating natural products with improved bioactivity and specificities.^[4]^ Although the biosynthesis of halogenated natural products has been studied for several decades, their biotechnological potential is far from being a developed industrial process.^[5]^ Thus, developing new protocols for stereospecific and stereoselective halogenation transformation through operationally mild reaction conditions is an intriguing and highly desirable research target.

The typical reaction mechanisms by which halide nucleophiles displace leaving groups in sp^3^‐hybridized carbon atoms are classified as one of the two canonical pathways, *S*_N_1 or *S*_N_2. The *S*_N_1 path provides a mixture of retention and inversion of the original configuration at the carbon atom. In contrast, the *S*_N_2 mechanism provides halogenation with stereoinversion of configuration, offering an efficient synthetic route towards chiral bioactive halide products and pharmaceuticals.^[6]^ In this context, the transformation of alcohols into an intermediate that can be readily displaced by a halide nucleophile with a defined absolute configuration has attracted much attention.[^7^, ^8^, ^9^, ^10^]

In recent decades, several examples of nucleophilic substitutions have led to products with different stereochemical outcomes. For example, Toste, Bergman, *et al*. demonstrated that some solvolysis substitution reactions, which typically occur with inversion, proceeded with overall stereochemical retention when the substitution occurs within a supramolecular cavity.^[11]^ Likewise, Jacobsen *et al*. reported the asymmetric catalysis of an *S*_N_1‐type reaction mechanism that results in the enantioselective construction of well‐defined quaternary stereocentres from racemic precursors.^[12]^ In the context of halogenation reactions, Bellucci, Lepore, *et al*. reported on the stereoretentive halogenation of cyclic alcohols catalyzed by titanium(IV) tetrahalides (TiX_4_, X=Cl, Br).[^13^, ^14^] The stereochemical preference in this case was explained by a mechanism in which the nonplanar carbocation intermediate is stabilized through hyperconjugation. Stereoretentive nucleophilic substitution may also be achieved mechanistically through a double inversion[^15^, ^16^] or an internal nucleophilic substitution mechanism (*S*_N_i).^[17]^ Apart from nucleophilic halide substitutions that involve charged/ion pair intermediates, the stereochemistry of radical‐based halogenation involves the abstraction of a radical leaving group and subsequent formation of a trigonal planar intermediate with an unpaired electron in the *p* orbital of the carbon atom. The reacting radical halogen may attack from either side of the trigonal plane, leading to a racemized mixture of the newly formed halo‐product. Thus, a racemic mixture is typically obtained in these cases, somewhat similar to the *S*_N_1 mechanism (known as the *S*_RN_1 mechanism).^[18]^ Lately, Phipps *et al*. have shown a highly effective catalyst for the enantioselective epimerization of 1,2‐ and 1,3‐diols through a radical hydrogen atom abstraction (HAA).^[19]^

**Scheme 1:**
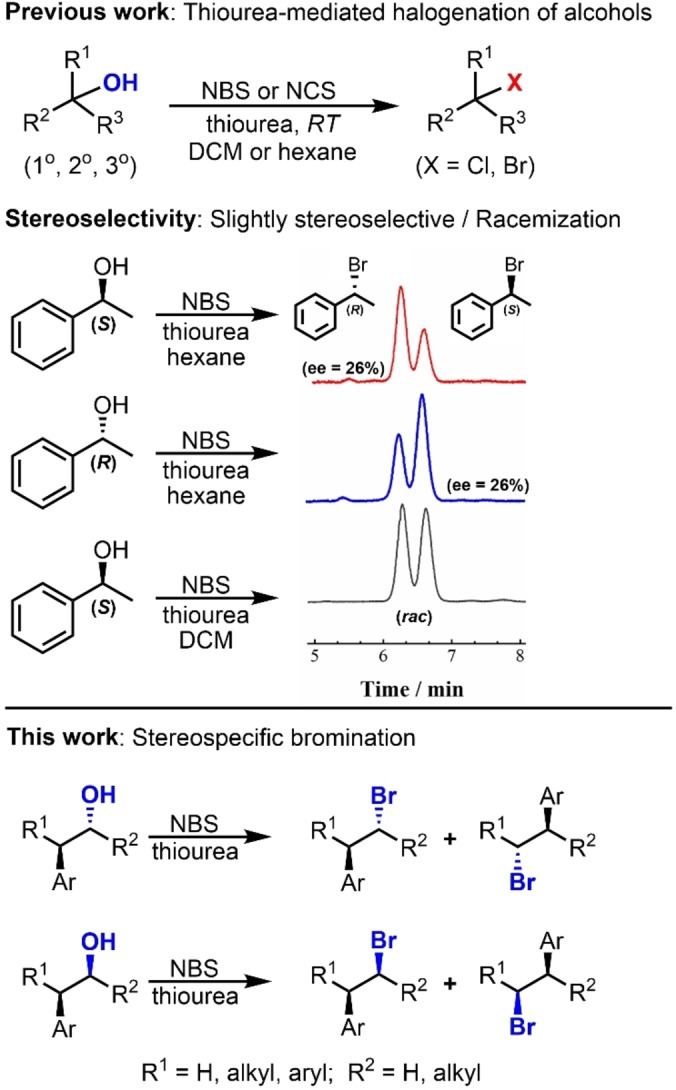
*Top*: A general scope for direct halogenation of alcohols mediated by NXS (X=Cl, Br) as a halogen source and a thiourea additive. *Middle*: Stereoselectivity in brominations of chiral alcohols. *Bottom*: Stereospecific bromination of alcohols following radical 1,2‐aryl migration.

Recently, we disclosed a straightforward methodology for directly transforming a wide scope of alcohols^[20],[21]^ to alkyl bromides and chlorides using thioureas and *N*‐halo succinimide (NXS) as halogen source in a single step under mild conditions (Scheme 1, *Top*).^[22]^ Detailed electron paramagnetic resonance (EPR) studies, isotopic labeling, and other control experiments strongly supported a radical‐based mechanism. The reagents′ ready availability, low cost, room temperature conditions, and the possibility of recycling the succinimide by‐product make this reaction highly convenient and atom‐efficient.

Regarding the stereochemical outcomes in this reaction, a fully racemized haloalkane was obtained when optically pure 1‐phenyl ethanol was mixed with dimethyl thiourea (DMTU) and *N*‐bromo‐succinimide (NBS) in methylene chloride. However, a partial preference for configuration inversion was observed when the bromination was conducted in *n*‐hexane (Scheme 1, *Middle*). However, allowing the reaction to continue for longer periods reduced the observed *ee* (from 26 % after one hour to 22 % *ee* after 3 hours), strongly suggesting that the alkyl halide continues to react, eventually leading to racemization as observed when the reaction was run in methylene chloride (see supporting information, Table S1). The possibility that aggregation‐induced chiral intermediates^[23],[24]^ could influence the mechanism was ruled out because no indication of radical aggregates was observed according to NMR and EPR spectroscopy^[25]^ under the employed reaction conditions.

Due to the critical importance of stereoselective conversions of alcohols to organic halides,^[26]^ we investigated the stereochemical outcomes of this reaction using alcohols containing two stereogenic centers. This approach allowed us to form diastereomeric products, which can be more readily distinguished than enantiomers using standard analytical techniques. Herein, we report both on the diastereospecific bromination of (*±*)‐*threo‐* or (*±*)‐*erythro*‐3‐aryl‐2‐butanols and on a novel enantiospecific bromination of chiral *β*‐aryl alcohols following the NBS and thiourea protocol (Scheme 1, *bottom*). The reaction can be carried out on several *β*‐aryl alcohols as long as the aryl groups are not too electron‐rich because of competing electrophilic aromatic substitution. The most surprising mechanistic finding of this study was its unexpected reaction pathway, where a spiro[2,5]octadienyl radical intermediate is formed and reacts with a bromide radical, leading to the observed overall retention of configuration. Understanding the mechanism of this reaction provided a novel pathway for the synthesis of chiral bromides from *β*‐aryl alcohols with inexpensive reagents, under very mild conditions, and with full retention of configuration.

## Results and Discussion

**Scheme 2:**
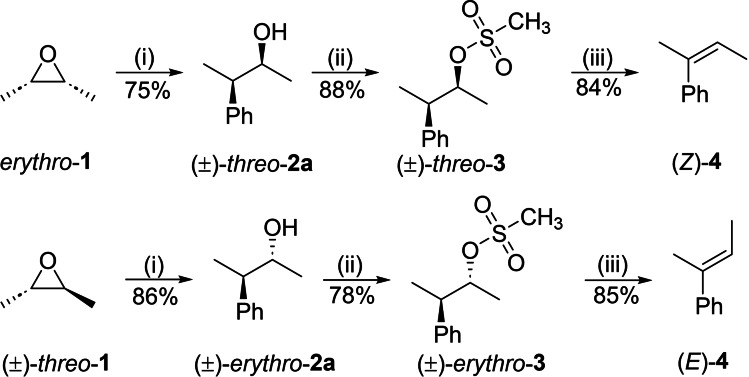
Synthesis of 3‐phenyl‐2‐butanol diastereomers and their elimination products. Reaction conditions: (i) PhMgBr (1.5 equiv), CuI (5 mol %), THF, 0 °C, 1 h; (ii) MsCl (1.2 equiv), pyridine (1.2 equiv), DCM, 0 °C‐*RT*, 12 h; (iii) KOH (1.2 equiv), MeOH‐DCM (3 : 1), reflux, 3 h.

Initially, the 3‐phenyl‐2‐butanols, (*±*)‐*threo*‐**2** and (*±*)‐*erythro*‐**2**, were synthesized as model substrates to study the stereoselectivity of the substitution. Compounds **2** were easily obtained starting from available (*±*)‐*threo‐* and *erythro*‐2,3‐dimethyloxirane **1**, followed by Grignard ring opening of the epoxide using copper iodide as a catalyst (Scheme 2).^[27]^

To verify the stereochemistry of **2** **a**, mesylation was carried out to provide mesylates (*±*)‐*threo*‐**3** and (*±*)‐*erythro*‐**3**, whereas subsequent *E*2 elimination afforded the (*Z*)‐ or (*E*)‐alkenes, respectively (Scheme S4).^[28],[29]^ With diastereomeric alcohols **2** in hand, the stereoselectivity of their bromination with the NBS/thiourea protocol was examined. Thus, (*±*)‐*erythro*‐**2** **a** was brominated using *N*‐bromo succinimide (NBS, 1.5 equiv) in the presence of DMTU under the standard reaction conditions,^[21]^ and the resulting stereochemistry of the product/s could be verified by *E*2 elimination. All products were fully characterized by NMR and GC‐MS analyses (see SI).

**Scheme 3:**
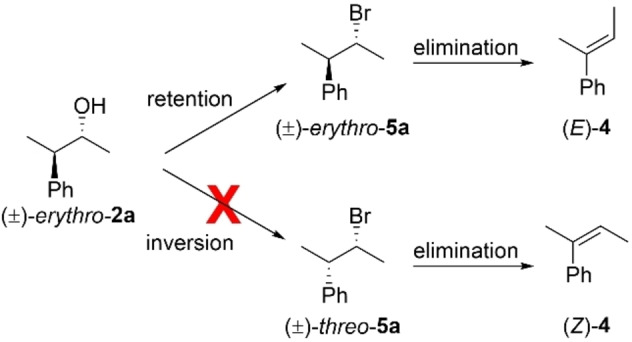
Stereoselective bromination pathways of (±)‐*erythro*‐**2** **a** and subsequent elimination.

Given the previous results (where chiral alcohols were fully or mostly racemized), it was very surprising to observe the exclusive formation of a single brominated diastereomer. The stereochemical outcome of the bromination was determined by the *E*2 elimination reaction of **5** **a** with KOH in reflux for 3 h. In this reaction, (*E*)‐**4**[^28^, ^29^] was exclusively obtained, thus revealing the stereoretentive nature of the bromination reaction (Scheme 3). This encouraging result prompted us to further optimize this reaction by exploring other thioureas and different reagent stoichiometries, as shown in Table 1 (see also SI).

**Table 1:** Reaction of (±)‐*erythro*‐**2** **a** with NBS and various disubstituted thioureas to afford (±)‐*erythro*‐**5** **a**.^[a]^

Entry	NBS (equiv)	Thiourea (equiv)^[b]^	Time (h)	Yield (%)^[c]^	SM (%)
1	1.5	DMTU (0.3)	12	37	58
2	1.5	PMTU (0.3)	12	40	55
3	2.0	PMTU (0.3)	12	65	25
4	2.0	PMTU (0.6)	12	52	37
**5**	**2.3**	**PMTU (0.3)**	**6**	**85** ^[d]^	**trace (<5)**
6	2.3	DMTU (0.3)	12	69	10
7	2.3	BnMTU (0.3)	12	70	9
8	2.3	NpMTU (0.3)	12	66	15
9	2.3	AdMTU (0.3)	12	64	13

[a] Reaction conditions: (*±*)‐*erythro*‐**2** **a** (0.33 mmol) was dissolved in dichloroethane (DCE, 2 mL) at *RT*. [b] DMTU: *N,N’*‐dimethylthiourea; PMTU: *N*‐propyl*‐N’*‐methylthiourea; BnMTU: *N*‐benzyl*‐N’*‐methylthiourea; NpMTU: *N*‐naphthyl*‐N’*‐methylthiourea; AdMTU: *N*‐adamantyl*‐N’*‐methylthiourea. [c] Yields were determined by ^1^H‐NMR using nitrobenzene as internal standard. [d] 79 % Isolated yield.

Following optimization, the combination of NBS (2.3 equiv) and PMTU (0.3 equiv) in dichloroethane provided (*±*)‐*erythro*‐**5** in 85 % yield in 6 h (79 % isolated yield). Reducing or increasing the amount of the thiourea did not improve the yields; on the contrary, product formation was suppressed in both cases. This behavior had been previously observed in other thiourea‐mediated halogenations of alcohols.^[22]^ As expected, no reaction occurred without NBS, and only oxidation of the alcohol to the carbonyl compound was detected in the absence of thiourea.

Solvent screening revealed that acetonitrile, 1,4‐dioxane, or tetrahydrofuran (THF) did not promote the reaction. In the case of dichloromethane (DCM), the bromination required longer reaction times (12 h) to afford a similar product yield (75 %). We also evaluated sterically hindered thiourea derivatives (entries 7–9); however, these did not improve the results compared to PMTU.

**Scheme 4:**
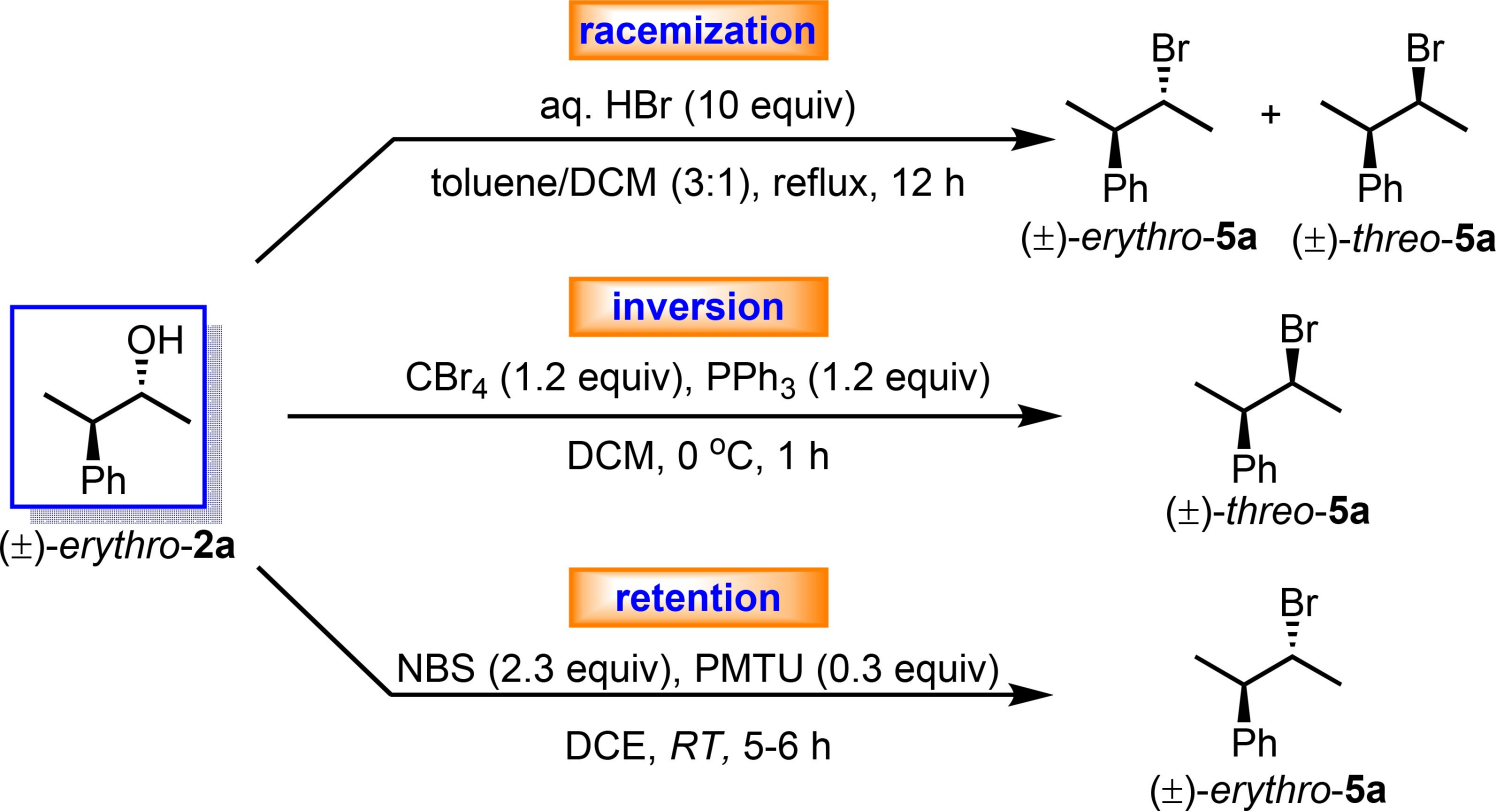
Transformation of (±)‐*erythro*‐3‐phenyl‐2‐butanol to the corresponding bromoalkane stereoisomers under three different reaction conditions.

To further highlight the uniqueness of this reaction, the stereospecific bromination of (*±*)‐*erythro*‐**2** **a** was compared with two classical bromination reactions: the Appel‐type bromination, which is known to afford inversion,^[30]^ and standard aqueous HBr‐mediated bromination, which is known to provide racemization. As expected, when (*±*)‐*erythro*‐**2** **a** was subjected to the Appel‐type bromination reaction with PPh_3_‐CBr_4_ in CH_2_Cl_2_, the resulting brominated product was found to be (*±*)‐*threo*‐**5** **a** as a single stereoisomer with complete inversion of stereochemistry at the carbon bearing the hydroxyl group. On the other hand, the aqueous HBr‐mediated bromination reaction of (*±*)‐*erythro*‐**2** provided a diastereomeric mixture of (*±*)‐*erythro*‐ and (*±)‐threo*‐**5** **a**. Thus, these different bromination conditions provide three different stereochemical outcomes (Scheme 4).

**Scheme 5:**
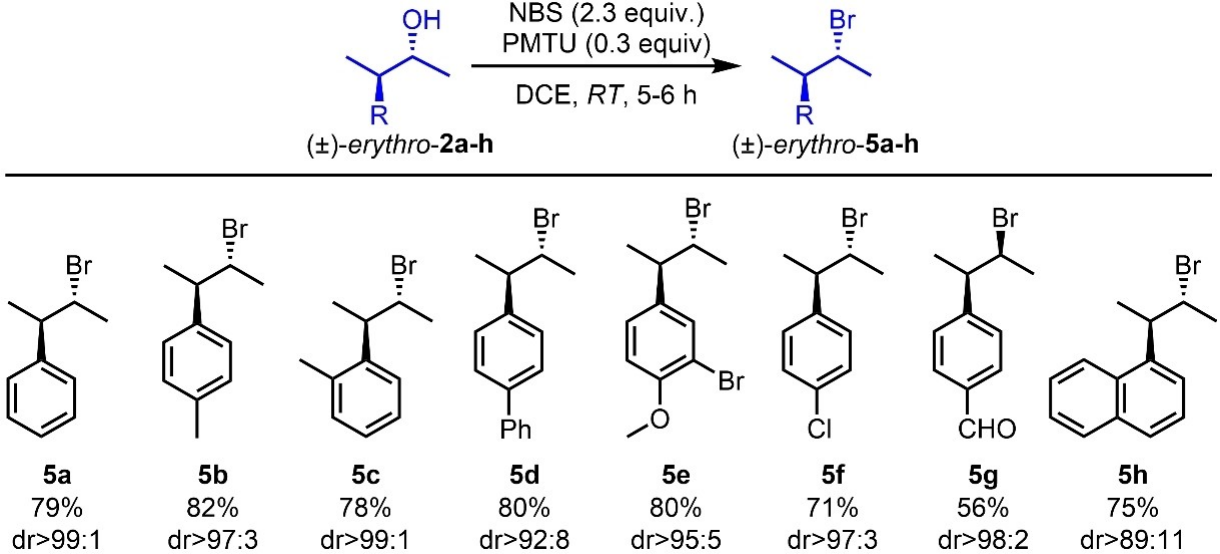
Synthesis of (*±*)‐*erythro*‐3‐phenyl‐2‐bromobutane derivatives, (*±*)‐*erythro* **‐5** **a‐h**. Yields are referred to as isolated products. Diastereomeric ratios (*dr*) were determined by GC‐MS analysis.

**Scheme 6:**
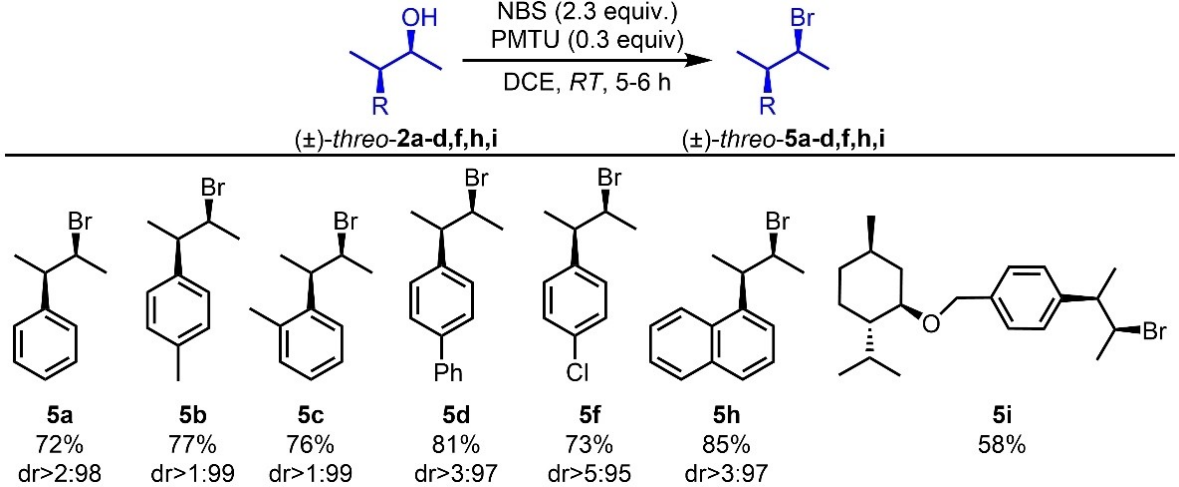
Synthesis of (*±*)‐*threo*‐3‐phenyl‐2‐bromobutane, (*±*)‐*threo* **‐5** **a**–**d**, **5** **g**, **5** **i** and **5** **j**. Yields and *dr* were determined as in Scheme 4.

Next, the reaction scope was investigated with other (*±*)‐*erythro‐* and (*±*)‐*threo‐*3‐aryl‐2‐butanols (Schemes 5 and 6). All reactions proceeded with good yields and excellent diastereospecificity ratios. However, compounds bearing strong electron‐donating groups, such as **2** **e**, also resulted in bromination of the aromatic ring. Furthermore, compound **2** **g** resulted in both bromination and oxidative cleavage at the benzylic position.^[31]^ Changing the 3‐aryl substituent by alkyl groups significantly hindered reaction progress. Indeed, the transformation of these alcohols to the corresponding bromoalkanes proceeded much more slowly, giving poor yields and substantial amounts of by‐products due to other bromination, elimination, and oxidation side reactions. For example, bromination of (−)‐menthol at a low thiourea/NBS ratio (0.3 : 1.5 equiv) afforded the brominated product in moderate yields (34 %), together with a substantial amount of unreacted starting material (57 %) (see SI).

At a higher thiourea/NBS ratio (0.6 : 2.5 equiv), the bromination of menthol proceeded with full consumption of the starting material to afford the desired product in better yields (61 %), but with many non‐specific side products (~35 % of the product mixture). These results demonstrate the vital role of the aryl group in achieving a clean stereospecific bromination.

**Scheme 7:**
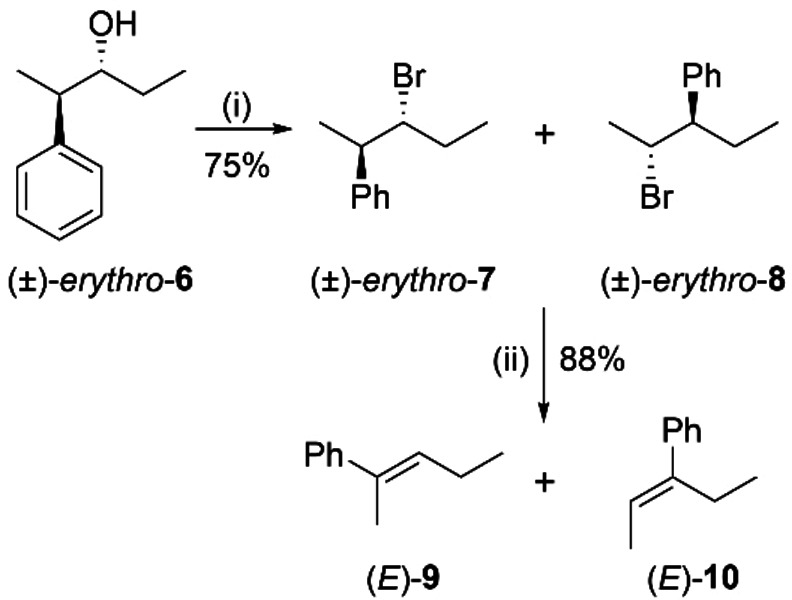
Bromination of (±)‐*erythro*‐2‐phenyl‐3‐pentanol, (±)‐*erythro* **‐6**. Reaction conditions: (i) NBS (2.3 equiv), PMTU (0.3 equiv), DCE, *RT*, 4 h; (ii) KOH (1.2 equiv), MeOH‐DCM (3 : 1), reflux, 3 h.

To additionally expand the scope of the reaction and gain further insight into its mechanism, the bromination of (*±*)‐*erythro*‐2‐phenyl‐3‐pentanol, (*±*)‐*erythro*‐**6**, was studied (Scheme 7). (*±*)‐*erythro* **‐6** was obtained by a stereoselective Felkin‐Ahn Grignard reaction from commercially available 2‐phenyl propanal.^[30]^

Unlike 3‐aryl‐2‐butanols, where each of the stereogenic centers contains a methyl group, in (*±*)‐*erythro* **‐6**, each stereogenic center is attached to a different alkyl group, *viz*. an ethyl and a methyl substituent. This modification proved critical for unveiling the mechanistic details of the bromination reaction. Much to our surprise, the reaction of (*±*)‐*erythro*‐**6** with NBS and PMTU led to a mixture of two products: the anticipated product with retention of configuration, (*±*)‐*erythro*‐2‐phenyl‐3‐bromopentane, (*±*)‐*erythro*‐**7**, alongside its isomeric product, (*±*)‐*erythro*‐3‐phenyl‐2‐bromopentane, (*±*)‐*erythro*‐**8**, in approximately equal yields (44 % and 56 %, respectively), as determined by NMR (Figures S57‐S59). Although the isolation of each of the two products from the reaction mixture on column chromatography was not successful in our hands due to their similarity, isolation from other impurities by preparative TLC was successful (1 % ethyl acetate in hexane). The ^1^H NMR spectrum of the product mixture and their elimination products unequivocally confirmed the presence of both isomers and their characterization.

**Scheme 8:**
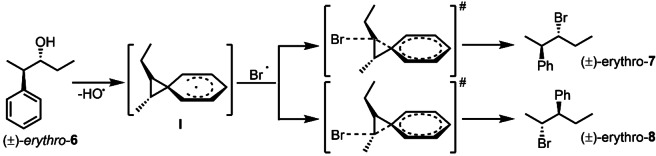
Bromination mechanism involving a spiro[2,5]octadienyl radical intermediate.

The unexpected formation of compound (*±*)‐*erythro*‐**8** led us to hypothesize the intermediacy of a spiro[2,5]octadienyl radical during the bromination reaction. Similar observations have been reported with allylic and propargylic alcohols.[^33^, ^34^] To the best of our knowledge, there is no precedence for a radical‐induced 1,2‐aryl migration through a Csp^3^‐Csp^3^ sigma bond.^[35]^ In analogy to the neophyl rearrangement,^[36]^ we propose that during homolytic cleavage of the hydroxy group in the presence of PMTU and NBS, a spiro[2,5]octadienyl radical (Scheme 8 intermediate **I**) is formed. Once intermediate **I** is formed, a bromide radical can attack any of the two cyclopropyl positions, forming compounds (*±*)‐*erythro*‐**7** and (*±*)‐*erythro*‐**8**. Hence, it was observed that both 1,2‐phenyl migration and bromination occur with double inversion of configuration, leading to products that retain the stereoisomeric configuration of the starting material.

In contrast to our previous alcohol halogenation examples,^[22]^ replacing the bromine donor, NBS, with molecular bromine proved less effective (33 % conversion according to GC) (Figure S70). Moreover, as a control experiment, butylated hydroxyl toluene (BHT) was added as a radical scavenger to the reaction of (±)‐*erythro‐* **6**, resulting in a full suppression of the reaction (Figure S68). Also, the reaction with bromine was fully suppressed by the addition of BHT (Figure S71). These results are aligned with the previously proposed radical mechanism in which thiourea and NBS generate radical species that activate the alcohol substrate, leading, in this case, to a 1,2‐phenyl migration and bromination.^[22]^

**Scheme 9:**
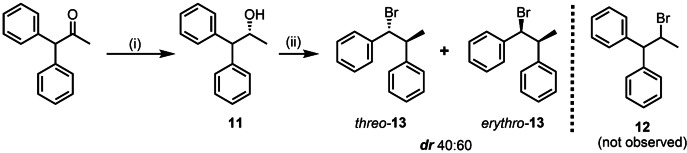
Reduction of 1,1‐diphenyl‐2‐propanone and subsequent bromination to afford sequential radical 1,2‐phenyl migration and bromination. Reaction conditions: (i) 1,1‐diphenyl‐2‐propanone (9.5 mmol), (S)‐B‐methyl‐oxazaborolidine (0.1 equiv); BH_3_‐Me_2_S complex (11 equiv), dry THF, N_2_, 50 °C, 1.5 h (chiral) or; 1,1‐diphenyl‐2‐propanone (0.95 mmol), LiAlH_4_ (1.75 equiv), dry THF, N_2_, 0 °C, 2 h (racemate) in 93–94 % yield for both conditions; (ii) (*rac*)**11** or (*R*)‐**11** (0.47 mmol), NBS (2.3 equiv.), PMTU (0.3 equiv), DCE, *RT*, 6 h.

1,1‐diphenyl‐2‐propanol (**11**) was probed as a model substrate (Scheme 9) because having two equivalent phenyl groups should provide a mixture of diastereomers if the aryl migration mechanism is valid. Thus, (*R*)‐(**11**) (70 % *ee*) was prepared from 1,1‐diphenyl‐2‐propanone by following asymmetric reduction with (*S*)‐B−Me oxazaborolidine catalyst,^[37]^ whereas (*rac*)‐**11** was synthesized by treating 1,1‐diphenyl‐2‐propanone with LiAlH_4_ in THF.

The bromination of (*R*)‐**11** with NBS and PMTU proceeded slowly, with low conversion. Nonetheless, following purification by preparative TLC, ^1^H NMR analysis and GC‐MS disclosed that the only brominated products obtained were the diastereoisomers of **13** and not compound **12**.^[38]^ This observation strongly supports our proposed mechanism that the intermediacy of a spiro[2,5]octadienyl radical is formed during the bromination reaction due to the stabilization of the incipient radical by one of the phenyl substituents, thus favoring the exclusive migration of the other phenyl ring.

The integration ratio in the ^1^H NMR spectrum of the crude reaction mixture between the assigned peaks for the CHBr protons of the *erythro*‐**13** isomer (at *δ*=5.09 ppm) and the *threo*‐**13** isomer (at *δ*=5.05 ppm) was approximately 60 : 40 (Figure S3). Following the use of preparative thin‐layer chromatography, *erythro*‐**13** could be isolated in 85 % purity.^[39]^ Notably, the conversion of **11** to the brominated products with NBS and PMTU could be slightly higher (37 %) if the temperature was raised to 40 °C and allowed to proceed for 20 h. Under such conditions, the ratio between the *erythro*‐ and *threo*‐**13** isomers was the same as previously observed, according to GC‐MS (Figure S4). As expected, very similar results were obtained when racemic **11** was brominated, with ~36 % conversion and the same product distribution according to ^1^H NMR and GC‐MS analyses (Schemes S7 and S8). These results are consistent with our proposed mechanism, where steric hindrance is a bit lower between the substituents in intermediate **I** for the *erythro* configuration (Scheme 8).

**Scheme 10:**
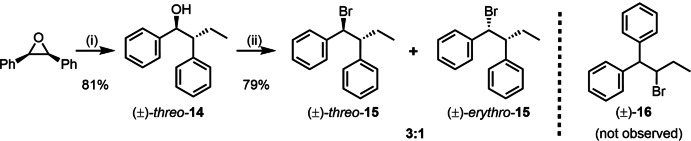
Stereoselective Lewis base‐catalyzed reaction of diphenyl oxirane with trialkyl aluminum and subsequent bromination. Reaction conditions: (i) (*2R*,*3S*)‐2,3‐diphenyl oxirane (0.51 mmol), AlEt_3_ (2 equiv), AsPh_3_ (10 mol‐%), *RT*, 24 h and (ii) (±)‐*threo*‐**14** (0.35 mmol), NBS (2.3 equiv.), PMTU (0.3 equiv), DCE, RT, 6 h.

So far, the proposed concerted radical 1,2‐phenyl migration and bromination have been examined in alcohols containing either a single or two geminal phenyl groups at the vicinal position to the hydroxy group. We also wanted to study the reaction with two vicinal phenyl groups, which in principle should provide similar results as the reaction from **11** to **13** (because the intermediate would be essentially the same). Therefore, 1,2‐diphenyl‐1‐butanol was prepared as a model substrate following a previously published procedure.^[40]^ Under these conditions, the alkylation proceeded with high stereoselectivity to afford a single diastereomer, namely, (±)‐*threo‐* **14** (Scheme 10).

To our great satisfaction, applying the standard bromination conditions to (±)‐*threo‐* **14** led to a mixture of brominated diastereomers (**15**) in 3 : 1 *dr* in 79 % isolated yield with no evidence for the formation of 1,1‐diphenyl‐2‐bromobutane (**16**), according to ^1^H NMR analysis. Hence, although the reaction afforded brominated products with good yields, the reduced stereoselectivity compared to our previous experiments (Schemes 5 and 6) can be explained by two types of bromination: a) by the 1,2‐phenyl migration mechanism, b) by the enhanced stabilization of a benzylic intermediate, and this stabilized radical species can also be brominated as we have previously shown.^[22]^

**Scheme 11:**
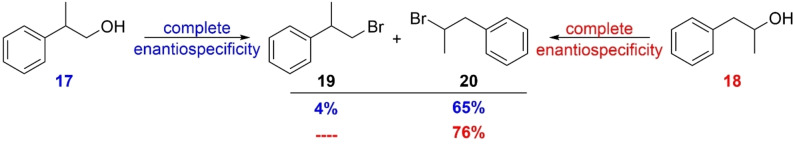
Bromination of **17** and **18** to develop enantioselective bromination methodologies. Reaction conditions: NBS (2.3 equiv.), PMTU (0.3 equiv), DCE, RT, 6 h.

Probably the most spectacular result was obtained by exploring bromination of 1‐phenyl‐2‐propanol (**17**) and 2‐phenyl‐1‐propanol (**18**), which is a useful intermediate for the preparation of the amphetamines and amphetaminil.^[41]^ In contrast to the previous substrates, **17** and **18** only have one asymmetric center. Given that we expect the bromination to follow the spiro[2,5]octadienyl radical intermediate mechanism (Scheme 8), both starting materials should form the same intermediate, and converge to give compound **20** as the major product. Moreover, we also expect full retention of configuration after bromination (Scheme 11). Initially, the bromination of *rac*‐**17** was performed under standard conditions. Indeed, bromination of *rac*‐**17** led almost exclusively to product **20**, indicating the formation of the spiro[2,5]octadienyl radical followed by the bromide radical attack on the more stable cyclopropyl radical position (see Figure S8, intermediate **I**), as expected. A 1,2‐dibromo minor side‐product could also be identified in the reaction mixture (~30 %, see SI). Next, the bromination reaction of 2‐phenyl‐1‐propanol (*rac*‐**18**) was studied. As predicted, direct bromination yielded *rac*‐**20** (76 %) as the major product, identical to that obtained from **17**, with complete suppression of the 1,2‐phenyl migration. In this reaction, compound **19** was not detected by either GC or NMR. Moreover, a similar amount of the 1,2‐dibromo side‐product could also be observed in the reaction mixture (~25 %, see SI). Finally, the bromination of chiral **18** was attempted. Remarkably, the reaction proceeded with complete enantiospecificity to yield chiral **20**, representing a rare example of alcohol substitution by a halide with retention of configuration.

## Conclusions

The direct stereoselective transformation of a wide scope of asymmetric secondary alcohols to alkyl bromides by using thioureas and NBS in a single step under mild conditions has been studied in depth. Based on preliminary observations that showed that bromination of a chiral secondary alcohol showed modest enantioselectivity in nonpolar solvents, the stereochemical outcomes of this reaction were investigated further. To this end, several diastereomeric alcohols were probed to enable the detection of the expected brominated diastereomers by means of simple NMR and chromatography techniques. Thus, the diastereospecific bromination of (*±*)‐*threo‐* or (*±*)‐*erythro*‐3‐phenyl‐2‐butanols was studied, surprisingly exclusively revealing a single diastereomeric brominated product with full retention of configuration. The reaction was carried out efficiently and diastereoselectively on many types of *β*‐aryl alcohols with similar results.

Moreover, the substitution of the hydroxy group on (*±*)‐*threo‐*2‐phenyl‐3‐pentanol and (*R*)‐1,1‐diphenyl‐2‐propano revealed a previously undetected (due to symmetry reasons) stereospecific 1,2‐migration of the phenyl group, occurring concurrently with the bromination of these compounds. The proposed mechanism of the 1,2‐phenyl migration involves the formation of a spiro[2,5]octadienyl radical, which is then attacked by a bromide radical at any of the two cyclopropyl positions, leading to products that retain the stereoisomeric configuration of the starting material. Further systematic studies pointed to the crucial role of the phenyl groups in different positions with respect to the alcohol group. Finally, the understanding of the mechanistic details allowed us to provide a simple methodology for bromination of *β*‐phenyl alcohols with full retention of configuration under very mild and inexpensive conditions to produce chiral derivatives of activated alkyl halides starting from readily available alcohols.

This serendipitous discovery provides a foundation for developing efficient stereospecific and stereoselective halogenation of chiral alcohols, with potential applications in natural product synthesis and the preparation of bioactive molecules. We are currently expanding the scope (substrates, solvents, and reagents) and studying the limitations of this new reaction, as well as its selectivity compared to our previously reported halogenations utilizing the same reagents.

## Supporting Information Summary

Detailed experimental methods, synthetic procedures, and full characterization data are included in the Supporting Information.

## Conflict of Interests

The authors declare no conflict of interest.

1

## Supporting information

As a service to our authors and readers, this journal provides supporting information supplied by the authors. Such materials are peer reviewed and may be re‐organized for online delivery, but are not copy‐edited or typeset. Technical support issues arising from supporting information (other than missing files) should be addressed to the authors.

Supporting Information

## Data Availability

The data that support the findings of this study are available in the supplementary material of this article.
