# Supplementary material for: Stereospecific Radical Bromination of β‐Aryl Alcohols with Thiourea Additives Through A Serendipitous Discovery of A 1,2‐Aryl Migration
Source: Chemistry. 2025 Jan 15;31(19):e202403831. doi: 10.1002/chem.202403831 (PMC11962344; doi:10.1002/chem.202403831)
Supplement: Supplementary file 1 — Supporting Information [file CHEM-31-e202403831-s001.pdf]

Chemistry–A European Journal

Supporting Information

Stereospecific Radical Bromination of *β*-Aryl Alcohols with Thiourea Additives Through A Serendipitous Discovery of A 1,2-Aryl Migration

Habib Assy, Uttam K. Mishra, Tom Rösler, Raman Khurana, N. Gabriel Lemcoff,* and Ofer Reany*

Stereospecific Radical Bromination of *β*-Aryl Alcohols with Thiourea Additives Through A Serendipitous Discovery of A 1,2-Aryl Migration

Habib Assy, Uttam K. Mishra, Tom Rösler, Raman Khurana, N. Gabriel Lemcoff,* and Ofer Reany*

TABLE OF CONTENTS

Entry	Content	Page
**1**	General information	S6
**2**	Synthesis	S6
**2.1**	General synthesis of (±)-*erythro*- and (±)-*threo*-**2a-h**	S6
	**Scheme S1.** Synthesis of (±)-*erythro*-3-aryl-2-butanol derivatives, **2a-h**	S6
**2.1.1**	(±)-*erythro*-3-phenyl-2-butanol (**2a**)	S7
**2.1.2**	(±)-*erythro*-3-(*p*-tolyl)-2-butanol (**2b**)	S7
**2.1.3**	(±)-*erythro*-3-(*o*-tolyl)-2-butanol (**2c**)	S7
**2.1.4**	(±)-*erythro*-3-([1,1'-biphenyl]-4-yl)-2-butanol (**2d**)	S7
**2.1.5**	(±)-*erythro*-3-(4-methoxyphenyl)-2-butanol (**2e**)	S7
**2.1.6**	(±)-*erythro*-3-(4-chlorophenyl)-2-butanol (**2f**)	S7
**2.1.7**	(±)-*erythro*-3-(4-(methoxymethyl)phenyl)-2-butanol (**2g**)	S8
**2.1.8**	(±)-*erythro*-3-(naphthalen-1-yl)-2-butanol (**2h**)	S8
	**Scheme S2.** Synthesis of (±)-*threo*-3-aryl-2-butanol derivatives, **2a-d**, **2f**, and **2h**.	S8
**2.1.9**	(±)-*threo*-3-phenyl-2-butanol (**2a**).	S8
**2.1.10**	(±)-*threo*-3-(*p*-tolyl)-2-butanol (**2b**)	S8
**2.1.11**	(±)-*threo*-3-(*o*-tolyl)-2-butanol (**2c**)	S9
**2.1.12**	(±)-*threo*-3-([1,1'-biphenyl]-4-yl)-2-butanol (**2d**)	S9
**2.1.13**	(±)-*threo*-3-(4-chlorophenyl)-2-butanol (**2f**)	S9
**2.1.14**	(±)-*threo*-3-(naphthalene-1-yl)-2-butanol (**2h**)	S9
**2.2**	General synthesis of (±)-*erythro*- and (±)-*threo*-**5a-h**	S9
**2.2.1**	(±)-*erythro*-3-phenyl-2-bromobutane, (±)-*erythro*-**5a**	S9
**2.2.2**	(±)-*threo*-3-phenyl-2-bromobutane, (±)-*threo*-**5a**	S10
**2.2.3**	(±)-*erythro* -3-(*p*-tolyl)-2-bromobutane, (±)-*erythro*-**5b**	S10
**2.2.4**	(±)-*threo*-3-(*p*-tolyl)-2-bromobutane, (±)-*threo*-**5b**	S10
**2.2.5**	(±)-*erythro* -3-(*o*-tolyl)-2-bromobutane, (±)-*erythro*-**5c**	S10
**2.2.6**	(±)-*threo*-3-(*o*-tolyl)-2-bromobutane, (±)-*threo*-**5c**	S10
**2.2.7**	(±)-*erythro* -3-([1,1'-biphenyl]-4-yl)-2-bromobutane, (±)-*erythro*-**5d**	S10
**2.2.8**	(±)-*threo*-3-([1,1'-biphenyl]-4-yl)-2- bromobutane, (±)-*threo*-**5d**	S10
**2.2.9**	(±)-*erythro*-3-(4-methoxyphenyl)-2-bromobutane, (±)-*erythro*-**5e**	S11
**2.2.10**	(±)-*erythro*-3-(4-chlorophenyl)-2-bromobutane, (±)-*erythro*-**5f**	S11
**2.2.11**	(±)-*threo*-3-(4-chlorophenyl)-2-bromobutane (±)-*threo*-**5f**	S11
**2.2.12**	(±)-*erythro*-3-(4-(caboxaldehyde)phenyl)-2-bromobutane, (±)-*erythro*-**5g**	S11
**2.2.13**	(±)-*erythro*-3-(naphthalene-1-yl)-2-bromobutane, (±)-*erythro*-**5h**	S11
**2.2.14**	(±)-*threo*-3-(naphthalene-1-yl)-2-bromobutane, (±)-*threo*-**5h**	S11
**2.3**	Synthesis of (±)-**5i**	S12
	**Scheme S3**. Synthesis of (±)-*threo*-**2i** and **5i**	S12
**2.3.1**	Synthesis of 4-bromobenzyl (-)menthyl ether	S12
**2.3.2**	Synthesis of (±)-*threo*-**2i**	S12
**2.3.3**	Synthesis of (±)-*threo*-**5i**	S13
**2.4**	Stereoisomer formation in *E*2 reactions of mesylated (±)-*erythro*- and (±)-*threo*-**2a.**	S14
	**Scheme S4**. Stereoselective synthesis of alkene **4** via mesylation (i) and elimination (ii) reactions of 3-phenyl-2-butanol diastereomers	S14
**2.4.1**	(±)-*erythro- and threo*-3-phenyl-2-butyl methanesulfonate, **3**.	S14
**2.4.2**.	(*E*)- *and* (*Z*)-2-phenyl-2-butene, **4**.	S14
**2.5**	Bromination of (±)-*erythro*-2-phenyl-3-pentanol.	S15

	**Scheme S5.** Synthesis of (±)-*erythro*-**6** and its brominated products	S15
**2.5.1**	Synthesis of (±)-*erythro*-2-phenyl-3-pentanol, (±)-*erythro*-**6**:	S15
**2.5.2**	Synthesis of (±)-*erythro*-**7** and (±)-*erythro*-**8**	S16
**2.6**	Stereochemical *E*2 reaction of mesylated (±)-*erythro*-2-phenyl-3-pentanol.	S16
	**Scheme S6**. Stereochemical *E*2 reaction of (±)-*erythro*-**6**.	S16
**2.6.1**	Synthesis of (±)-*erythro*-2-phenyl-3-pentyl methanesulfonate	S16
**2.6.2**	Synthesis of (*E*)-2-phenyl-2-pentene.	S17
**2.7**	Bromination of (*R*)-1,1-diphenyl-2-propanol, (*R*)-**11:**	S17
	**Scheme S7.** Synthesis of (*R*)-**11** and its brominated products	S17
**2.7.1**	Synthesis of (*R*)-1,1-diphenyl-2-propanol, (*R*)-**11:**	S17
	**Figure S1**. HPLC chromatography of (*R*)-1,1-diphenyl-2-propanol and *ee* determination. M means manual, and F means forced. Hence, MF means manual-to-forced integration and vice versa for FM.	S18
**2.7.2**	Bromination of (*R*)-1,1-diphenyl-2-propanol, (*R*)-**11:**	S19
	**Figure S2.** GC-MS chromatograph showing products obtained in the bromination reaction of (R)-11.	S19
	**Figure S3**. ^{1}H NMR (400 MHz, $CDCl_3$) spectrum of crude reaction following bromination of *(R)*-**11**.	S19
	**Figure S4**. GC-MS chromatograph showing the separation of all products in the bromination reaction of (R)-11 at 40 ºC over 20 h.	S20
**2.8**	Bromination of (±)-1,1-diphenyl-2-propanol.	S20
	**Scheme S8.** Synthesis of (±)-**11** and its brominated products	S20
**2.8.1**	Synthesis of (±)-1,1-diphenyl-2-propanol, (±)-**11:**	S20
	**Figure S5**. GC-MS chromatograph presenting the purity of (±)-**11**.	S21
	**Figure S6**. HPLC chromatography of (±)-1,1-diphenyl-2-propanol and *ee* determination. BB means baseline-to-baseline integration.	S21
**2.8.2**	Bromination of (±)-1,1-diphenyl-2-propanol, (±)-**11:**	S21
	**Figure S7**. GC-MS chromatograph presenting the diastereomeric ratio (58:42) between (±)-*erythro*- and (±)-*threo*-**13**.	S22
	**Figure S8**. ^{1}H NMR (400 MHz, $CDCl_3$) spectrum of crude reaction following bromination of *(*±)-**11**.	S22
**2.9**	Bromination of (±)-*threo*-1,2-diphenyl-1-butanol, (±)-*threo*-**14**	S23
	**Scheme S9.** Synthesis of (±)-*threo*-**14** and its brominated products	S23
**2.9.1**	Synthesis of (±)-*threo*-**14**	S23
**2.9.2**	Synthesis of (±)-*threo*- and (±)-ery*thro*-1,2-diphenyl-1-bromobutane, (±)-*threo*-**15** and (±)-ery*thro*-**15**.	S23
**3.**	Stereoselective bromination of chiral 1-phenyl-1-ethanol	S24
	**Table S1**. Stereoselective bromination of chiral 1-phenyl-1-ethanol in dichloromethane and hexane.	S24
**4.**	Proving the stereoselective bromination pathways of (±)-*erythro*-**2a.**	S25
	**Scheme S10**. Stereoselective synthesis of alkene **4** via bromination (i) and elimination (ii) reactions of 3-phenyl-2-butanol diastereomers. Reaction conditions: (i) NBS (2.3 equiv), PMTU (0.3 equiv), dichloroethane, *RT*, 6 h; (ii) KOH (1.2 equiv), 25% dichloromethane in methanol, 90 ºC, 3 h.	S25
	**Figure S9**. ^{1}H NMR (400 MHz) spectra of crude products resulted from elimination reactions of **5a**. *Top*: (±)-*erythro*-3-phenyl-2-butanol; *Bottom*: and (±)-*threo*-3-phenyl-2-butanol.	S26
**6.**	Optimization of reaction parameters in the bromination of (±)-*erythro*-**2a** with NBS and various disubstituted thioureas to afford (±)-*erythro*-**5a**.	S27
**6.1**	General procedure.	S27
	**Figure S10.** ^{1}H NMR (400 MHz) of (±)-*erythro*-**2a** with nitrobenzene.	S27
	**Figure S11.** ^{1}H NMR (400 MHz) spectra of products following the bromination of (±)-*erythro*-**2a** under specific reaction conditions given in Table 1: *Top*: Entry 1.; *Bottom:* Entry 2.	S28
	**Figure S12.** ^{1}H NMR (400 MHz) spectra of products following the bromination of (±)-*erythro*-**2a** under specific reaction conditions given in Table 1: *Top*: Entry 3.; *Bottom:* Entry 4.	S29

	**Figure S13.** ^{1}H NMR (400 MHz) spectra of products following the bromination of (±)-*erythro*-**2a** under specific reaction conditions given in Table 1: *Top*: Entry 5.; *Bottom:* Entry 6.	S30
	**Figure S14.** ^{1}H NMR (400 MHz) spectra of products following the bromination of (±)-*erythro*-**2a** under specific reaction conditions given in Table 1: *Top*: Entry 7; *Bottom:* Entry 8.	S31
	**Figure S15.** ^{1}H NMR (400 MHz) spectrum of products following the bromination of (±)-*erythro*-**2a** under specific reaction conditions given in Table 1, entry 9.	S32
**6**	Transformation of (±)-erythro-3-phenyl-2-butanol to the corresponding bromoalkane stereoisomers under three different reaction conditions.	S32
	**Figure S16**. Appel-type bromination of (±)-*erythro*-3-phenyl-2-butanol and GC analysis of the crude reaction.	S32
	**Figure S17**. Appel-type bromination of (±)-*threo*-3-phenyl-2-butanol and GC analysis of the crude reaction.	S33
	**Figure S18**. Bromination of (±)-*erythro*-3-phenyl-2-butanol with aqueous HBr and GC analysis of the crude reaction.	S34
**7**	NMR data	S35
	**Figure S19**. NMR spectra of (±)-*erythro*-3-phenyl-2-butanol in $CDCl_3$. *Top*: ^{1}H NMR (400 MHz), *Bottom:* ^{13}C NMR (100 MHz).	S35
	**Figure S20**. NMR spectra of (±)-*threo*-3-phenyl-2-butanol, (±)-*threo*-**2a**, (±)-*erythro*-**2a** in $CDCl_3$. *Top*: ^{1}H NMR (400 MHz), *Bottom:* ^{13}C NMR (100 MHz).	S36
	**Figure S21**. NMR spectra of (±)-*erythro*-3-(*p*-tolyl)-2-butanol, (±)-*erythro*-**2b** in $CDCl_3$. *Top*: ^{1}H NMR (400 MHz), *Bottom:* ^{13}C NMR (100 MHz).	S37
	**Figure S22**. NMR spectra of (±)-*threo*-3-(*p*-tolyl)-2-butanol, (±)-*threo*-**2b** in $CDCl_3$. *Top*: ^{1}H NMR (400 MHz), *Bottom:*^{13}C NMR (100 MHz).	S38
	**Figure S23**. NMR spectra of (±)-*erythro*-3-(*o*-tolyl)-2-bromobutane, (±)-*erythro*-**2c** in $CDCl_3$. *Top*: ^{1}H NMR (400 MHz), *Bottom:* ^{13}C NMR (100 MHz).	S39
	**Figure S24**. NMR spectra of (±)-*threo*-3-(*o*-tolyl)-2-butanol, (±)-*threo*-**2c** in $CDCl_3$. *Top*: ^{1}H NMR (400 MHz), *Bottom:* ^{13}C NMR (100 MHz).	S40
	**Figure S25**. NMR spectra of (±)-*erythro*-3-([1,1'-biphenyl]-4-yl)-2-bromobutane, (±)-*erythro*-**2d** in $CDCl_3$. *Top*: ^{1}H NMR (400 MHz), *Bottom:* ^{13}C NMR (100 MHz).	S41
	**Figure S26**. NMR spectra of (±)-*threo*-3-([1,1'-biphenyl]-4-yl)-2-bromobutane, (±)-*threo*-**2d** in $CDCl_3$. *Top*: ^{1}H NMR (400 MHz), *Bottom:* ^{13}C NMR (100 MHz).	S42
	**Figure S27**. NMR spectra of (±)-*erythro*-3-(4-methoxyphenyl)-2-butanol, (±)-*erythro*-**2e** in $CDCl_3$. *Top*: ^{1}H NMR (400 MHz), *Bottom:* ^{13}C NMR (100 MHz).	S43
	**Figure S28**. NMR spectra of (±)-*erythro*-3-(4-chlorophenyl)-2-butanol, (±)-*erythro*-**2f** in $CDCl_3$. *Top*: ^{1}H NMR (400 MHz), *Bottom:* ^{13}C NMR (100 MHz).	S44
	**Figure S29**. NMR spectra of (±)-*threo*-3-(4-chlorophenyl)-2-butanol, (±)-*threo*-**2f** in $CDCl_3$. *Top*: ^{1}H NMR (400 MHz), *Bottom:* ^{13}C NMR (100 MHz).	S45
	**Figure S30**. NMR spectra of (±)-*erythro*-3-(4-(methoxymethyl)phenyl)-2-butanol, (±)-*erythro*-**2g** in $CDCl_3$. *Top*: ^{1}H NMR (400 MHz), *Bottom:* ^{13}C NMR (100 MHz).	S46
	**Figure S31**. NMR spectra of (±)-*erythro*-3-(1-naphthyl)-2-butanol, (±)-*erythro*-**2h** in $CDCl_3$. *Top*: ^{1}H NMR (400 MHz), *Bottom:* ^{13}C NMR (100 MHz).	S47
	**Figure S32**. NMR spectra of (±)-*threo*-3-(1-naphthyl)-2-butanol, (±)-*threo*-**2h** in $CDCl_3$. *Top*: ^{1}H NMR (400 MHz), *Bottom:* ^{13}C NMR (100 MHz).	S48
	**Figure S33**. NMR spectra of (±)-*erythro*-3-phenyl-2-bromobutane, (±)-*erythro*-**5a** in $CDCl_3$. *Top*: ^{1}H NMR (400 MHz), *Bottom:* ^{13}C NMR (100 MHz).	S49
	**Figure S34**. NMR spectra of (±)-*threo*-3-phenyl-2-bromobutane, (±)-*threo*-**5a** in $CDCl_3$. *Top*: ^{1}H NMR (400 MHz), *Bottom:* ^{13}C NMR (100 MHz).	S50
	**Figure S35**. NMR spectra of (±)-*erythro*-3-(*p*-tolyl)-2-bromobutanel, (±)-*erythro*-**5b** in $CDCl_3$. *Top*: ^{1}H NMR (400 MHz), *Bottom:* ^{13}C NMR (100 MHz).	S51
	**Figure S36**. NMR spectra of (±)-*threo*-3-(*p*-tolyl)-2-bromobutane, (±)-*threo*-**5b** in $CDCl_3$. *Top*: ^{1}H NMR (400 MHz), *Bottom:*^{13}C NMR (100 MHz).	S52
	**Figure S37**. NMR spectra of (±)-*erythro*-3-(*o*-tolyl)-2-bromobutane, (±)-*erythro*-**5c** in $CDCl_3$. *Top*: ^{1}H NMR (400 MHz), *Bottom:* ^{13}C NMR (100 MHz).	S53
	**Figure S38**. NMR spectra of (±)-*threo*-3-(*o*-tolyl)-2-bromobutane, (±)-*threo*-**5c** in $CDCl_3$. *Top*: ^{1}H NMR (400 MHz), *Bottom:* ^{13}C NMR (100 MHz).	S54
	**Figure S39**. NMR spectra of (±)-*erythro*-3-([1,1'-biphenyl]-4-yl)-2-bromobutane, (±)-*erythro*-**5d** in $CDCl_3$. *Top*: ^{1}H NMR (400 MHz), *Bottom:* ^{13}C NMR (100 MHz).	S55

	**Figure S40**. NMR spectra of (±)-*threo*-3-([1,1'-biphenyl]-4-yl)-2-bromobutane, (±)-*threo*-**5d** in $CDCl_3$. *Top*: ^{1}H NMR (400 MHz), *Bottom:* ^{13}C NMR (100 MHz).	S56
	**Figure S41**. NMR spectra of (±)-*erythro*-3-(3-bromo-4-methoxyphenyl)-2-bromobutane, (±)-*erythro*-**5e** in $CDCl_3$. *Top*: ^{1}H NMR (400 MHz), *Bottom:* ^{13}C NMR (100 MHz).	S57
	**Figure S42**. NMR spectra of (±)-*erythro*-3-(4-chlorophenyl)-2-bromobutane, (±)-*erythro*-**5f** in $CDCl_3$. *Top*: ^{1}H NMR (400 MHz), *Bottom:* ^{13}C NMR (100 MHz).	S58
	**Figure S43**. NMR spectra of (±)-*threo*-3-(4-chlorophenyl)-2-bromobutane, (±)-*threo*-**5f** in $CDCl_3$. *Top*: ^{1}H NMR (400 MHz), *Bottom:* ^{13}C NMR (100 MHz).	S59
	**Figure S44**. NMR spectra inof (±)-*erythro*-3-(4-(carbaldehyde)phenyl)-2-bromobutane, (±)-*erythro*-**5g** in $CDCl_3$. *Top*: ^{1}H NMR (400 MHz), *Bottom:* ^{13}C NMR (100 MHz).	S60
	**Figure S45**. NMR spectra of (±)-*erythro*-3-(1-naphthyl)-2-bromobutane, (±)-*erythro*-**5h** in $CDCl_3$. *Top*: ^{1}H NMR (400 MHz), *Bottom:* ^{13}C NMR (100 MHz).	S61
	**Figure S46**. NMR spectra of (±)-*threo*-3-(1-naphthyl)-2-bromobutane, (±)-*threo* -**5h** in $CDCl_3$. *Top*: ^{1}H NMR (400 MHz), *Bottom:* ^{13}C NMR (100 MHz).	S62
	**Figure S47**. *Top:* ^{1}H NMR spectra of (±)-*threo*-4-bromobenzyl (-)-menthyl ether, **5i** in $CDCl_3$. *Bottom:* GC-MS chromatogram confirming a single diastereomer product.	S66
	**Figure S48**. NMR spectra of (±)-*threo*-**2i** in $CDCl_3$. *Top*: ^{1}H NMR (400 MHz), *Bottom:* ^{13}C NMR (100 MHz).	S64
	**Figure S49**. NMR spectra of (±)-*threo*-**5i** in $CDCl_3$. *Top*: ^{1}H NMR (400 MHz), *Bottom:* ^{13}C NMR (100 MHz).	S65
	**Figure S50**. NMR spectra of (±)-*erythro* -3-phenyl-2-butyl methanesulfonate, (±)-*erythro*-**3** in $CDCl_3$. *Top*: ^{1}H NMR (400 MHz), *Bottom:* ^{13}C NMR (100 MHz).	S66
	**Figure S51**. GC-MS of (±)-*erythro*-3-phenyl-2-butyl methanesulfonate, (±)-*erythro*-**3**.	S67
	**Figure S52**. ^{1}H NMR (400 MHz) spectrum of (±)-*threo*-3-phenyl-2-butyl methanesulfonate, (±)-*threo*-**3** in $CDCl_3$.	S67
	**Figure S53**. ^{13}C NMR (100 MHz) spectrum of (±)-*threo*-3-phenyl-2-butyl methanesulfonate, (±)-*threo*-**3** in $CDCl_3$ (*top)* and GC-MS chromatogram (*bottom*).	S68
	**Figure S54**. NMR spectra of (*E*)-2-phenyl-2-butene, (*E*)-**4** in $CDCl_3$. *Top*: ^{1}H NMR (400 MHz), *Bottom:* ^{13}C NMR (100 MHz).	S69
	**Figure S55**. NMR spectra of (*Z*)-2-phenyl-2-butene, (*Z*)-**4** in $CDCl_3$. *Top*: ^{1}H NMR (400 MHz), *Bottom:* ^{13}C NMR (100 MHz).	S70
	**Figure S56**. NMR spectra of (±)-*erythro*-2-phenyl-3-pentanol, (±)-*erythro*-**6** in $CDCl_3$. *Top*: ^{1}H NMR (400 MHz), *Bottom:* ^{13}C NMR (100 MHz).	S71
	**Figure S57**. NMR spectra of (±)-*erythro*-2-phenyl-3-pentanyl methane sulfonate, (±)-*erythro*-mesylated-**6** in $CDCl_3$. *Top*: ^{1}H NMR (400 MHz), *Bottom:* ^{13}C NMR (100 MHz).	S72
	**Figure S58**. NMR spectra of (*E*)-2-phenyl-2-pentene, (*E*)-**9**, in $CDCl_3$. *Top*: ^{1}H NMR (400 MHz), *Bottom:* ^{13}C NMR (100 MHz).	S73
	**Figure S59**. NMR spectra of (±)-*erythro*-2-phenyl-3-bromopentane, (±)-*erythro*-**7**, and (±)-*erythro*-3-phenyl-2-bromopentane, (±)-*erythro*-**8** in $CDCl_3$. *Top*: ^{1}H NMR (400 MHz), *Bottom:* Expansion of the NMR spectrum at selected chemical shift ranges.	S74
	**Figure S60**. NMR spectra of (±)-*erythro*-2-phenyl-3-bromopentane, (±)-*erythro*-**7**, and (±)-*erythro*-3-phenyl-2-bromopentane, (±)-*erythro*-**8** in $CDCl_3$. *Top*: ^{13}C NMR (100 MHz), *Bottom:* DEPT-135 NMR experiment	S75
	**Figure S61**. NMR spectra of (±)-*erythro*-2-phenyl-3-bromopentane, (±)-*erythro*-**7**, and (±)-*erythro*-3-phenyl-2-bromopentane, (±)-*erythro*-**8** in $CDCl_3$. *Top*: COSY 2D-NMR (400 MHz), *Bottom:* HMQC 2D-NMR	S76
	**Figure S62**. NMR spectra of (*R*)-1,1-diphenyl-2-propanol, (*R*)-**11** in $CDCl_3$. *Top*: ^{1}H NMR (400 MHz), *Bottom:* ^{13}C NMR (100 MHz).	S77
	**Figure S63**. Stereoselective bromination of (R)-**11**. *Top*: HPLC chromatograph of enantiopure substrate. *Bottom*: HPLC chromatographs of diastereomeric products of **13**.	S78
	**Figure S64**. NMR spectra in $CDCl_3$ following bromination of (*R*)-**11** (70% *ee*) at *RT* for 6 h. *Top*: ^{1}H NMR (400 MHz); *Bottom*: ^{13}C NMR (100 MHz).	S79
	**Figure S65**. NMR data of (±)-*threo*-1,2,-diphenyl-1-butanol, (±)-*threo*-**14**. *Top*: ^{1}H NMR (400 MHz, $CDCl_3$). *Bottom*: ^{13}C NMR (100 MHz, $CDCl_3$)	S80

	**Figure S66**. GC-MS chromatogram presenting the *dr* between (±)-*threo*- and (±)-*erythro*-**14**.	S81
	**Figure S67**. ^{1}H NMR spectrum (400 MHz, $CDCl_3$) of (±)-*threo* and (±)-*erythro*-1,2,-diphenyl-1-bromobutane, (±)-*threo*-**15** and (±)-*erythro*-**15** (*dr* 3:1).	S81
	**Figure S68**. GC-MS chromatogram of the crude reaction containing (±)-*threo*-**15** and (±)-*erythro*-**15** (*dr* 3:1)	S82
**8**	Miscellaneous experiments in the bromination reaction of (±)-*erythro*-**6**.	S82
**8.1**	Reaction with butylated hydroxyl toluene (BHT) as a radical scavenger.	S82
	**Figure S69**. GC-MS chromatogram of the crude reaction containing (±)-*erythro*-**6** and BHT. The fact that neither the bromination products (±)-*erythro*-**7** and (±)-*erythro*-**8** nor their elimination products were found indicates that the radical bromination reaction did not occur at all.	S82
**8.2**	Reaction without thiourea	S83
	**Figure S70**. GC-MS chromatogram of the crude reaction containing (±)-*erythro*-**6** and succinimide. The fact that neither the brominated products (±)-*erythro*-**7** and (±)-*erythro*-**8** nor their elimination products were found proves that radical bromination is suppressed if thioureas do not take place in this type of transformation	S84
**8.3**	Reaction with molecular bromine (Br_2) as a different bromine donor with or without thiourea	S84
	**Figure S71**. GC-MS chromatograms of the crude reaction containing (±)-*erythro*-**6** and Br_2 with (*left*) or without (*right*) PMTU. The fact that both brominated products (±)-*erythro*-**7** and (±)-*erythro*-**8** are present in the crude reaction indicates that both procedures followed radical bromination	S85
**8.4**	Reaction with Br_2 and BHT	S85
	**Figure S72.** GC-MS chromatogram of the crude reaction following the bromination of (±)-*erythro*-**6** with Br_2, PMTU and BHT. The fact that neither the bromination products (±)-*erythro*-**7** and (±)-*erythro*-**8** nor their elimination products were found indicates that the radical bromination reaction did not occur.	S86
**9**	Bromination reactions of β-aryl propanols	S86
	**Scheme S11**. Bromination *rac*-**17** and **18**	S86
**9.1**	Reaction of (*rac*)-2-phenyl-1-propanol (*rac*-**17**) with NBS and PMTU	S86
**9.2**	Reaction of (*rac*)-1-phenyl-2-propanol (*rac*-**18**) with NBS and PMTU	S87
	**Figure S73**. GC-MS chromatogram of the crude reaction following the bromination of *rac*-**17** with NBS and PMTU (*left*) and bromination unde the same conditions with rac-**18** as substrate (*right*).	S87
**9.3**	NMR data of compounds **20** and **20’**	S88
	**Figure S74**. ^{1}H NMR data (400 MHz, $CDCl_3$) of compounds **20** and **20'** following bromination of substrate **17**	S88
	**Figure S75**. ^{1}H NMR data (400 MHz, $CDCl_3$) of compounds **20'** following bromination of substrate **18**	S89
**10**	Enantiospecificity in bromination of enantiomeric enriched 1-phenyl-2-propanol, **18**	S89
**10.1**	Mosher ester of (*rac*)-1-phenyl-2-propanol (*rac*-**18**)	S89
	**Figure S76**. HPLC chromatogram of Mosher ester (isolated diastereomer by preparative TLC).	S89
**10.2**	Hydrolysis of Mosher ester to afford enantiomerically enriched **18**	S90
	**Figure S77**. HPLC chromatogram of (*rac*)-18 (**A**) and enantiomerically enriched **18** after alkaline hydrolysis of Mosher ester (**B**).	S90
**10.3**	Bromination of enantiomerically enriched **18**	S91
	**Figure S78**. HPLC chromatogram of (*rac*)-**20** (**A**) and enantiomerically enriched **20** following bromination of enantiomerically enriched **18** (**B**).	S92

1. General Information

Reagents, chemicals and solvents were purchased from various commercial sources and used without further purification unless otherwise noted. NBS was recrystallized from hot water, stored in the dark and used as required. Thin-layer chromatography (TLC) was performed using silica gel 60 F254 pre-coated on aluminum plates.

^{1}H, ^{13}C and NMR spectra were recorded with the solvents indicated using AVIII400 Bruker spectrometer. Chemical shifts (δ) are given in ppm relative to TMS. The residual solvent signals were used as references, and the chemical shifts were converted to the TMS scale: $CDCl_3$: δ_H = 7.26 ppm, δ_C = 77.16 ppm. Coupling constants *J* (Hz) were directly taken from the spectra. Splitting patterns are designated as *s* (singlet), *d* (doublet), *t* (triplet), *q* (quartet), *m* (multiplet), or *b* for broad and overlapped signals.

GC-MS spectra were recorded by using an Agilent 6850 instrument equipped with an Agilent 5973 MSD working under standard conditions and an Agilent HP5-MS column.

HPLC analysis was carried out on Agilent 1260 instrument G4212-60008 photodiode array detector and Daicel Chiralpak AS-H column 4.6 mm × 250 mm (5 µm). The mobile phase contained a solvent mixture of 1% isopropanol in hexane at a flow rate of 1.0 mL/min.

2. Synthesis

2.1 General synthesis of (±)-*erythro*- and (±)-*threo* -**2a-h**

To an oven-dried round bottom flask filled with dry THF (10 mL) under nitrogen atmosphere, *erythro*- or (±)-*threo*-2,3-dimethyloxirane **1** (400 mg, 5.55 mmol) and CuI (52.8 mg, 0.28 mmol) were added. Then, the solution was cooled to 0 °C, and the Grignards reagent, RMgBr (1.5 eq), was added dropwise over 10 min. The reaction mixture was maintained at 0 °C and left under stirring for an additional 50 min. Then, the reaction mixture was quenched with saturated aqueous ammonium chloride solution and extracted with ethyl acetate (4x5 mL). The organic extracts were combined, dried over anhydrous sodium sulfate and concentrated under a vacuum. The crude product was purified by silica gel column chromatography using hexanes/ethyl acetate (4:1) mixture as eluent to afford the desired products in 69-88% (Scheme S1 and Scheme S2).

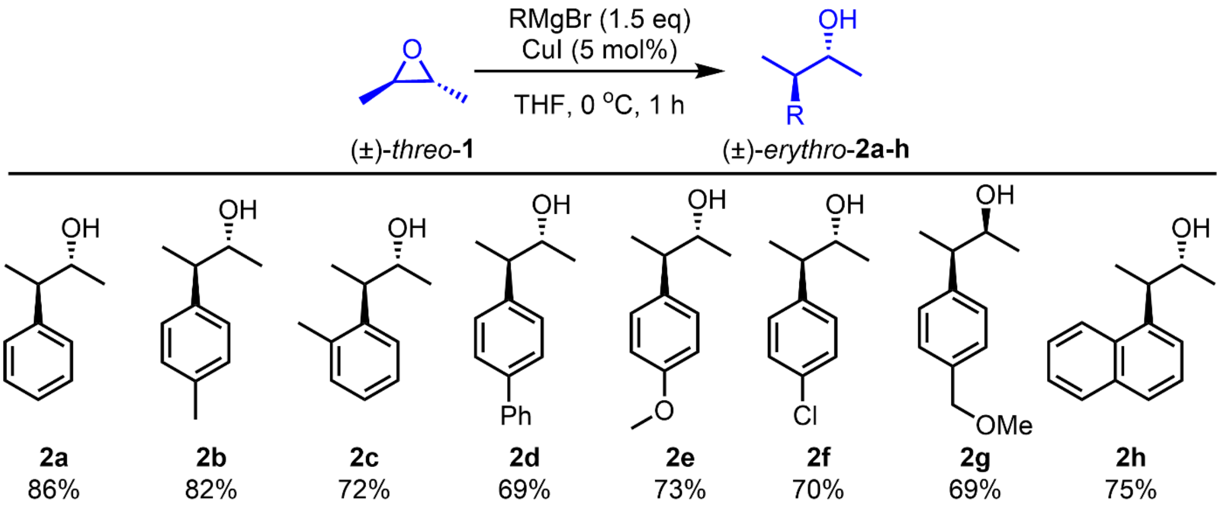

Scheme S1. Synthesis of (±)-*erythro*-3-aryl-2-butanol derivatives, **2a-h**.

2.1.1 (±)-*erythro*-3-phenyl-2-butanol (**2a**).

Colorless liquid. R_f = 0.50 (silica gel, 20% ethyl acetate in hexanes, visualized with *p*-anisaldehyde stain); ^{1}H NMR (400 MHz, $CDCl_3$): δ 7.25-7.17 (m, 2H), 7.17-7.06 (m, 3H), 3.77 (quint, J = 6.3 Hz, 1H), 2.63 (quint, J = 6.9 Hz, 1H), 1.87 (bs, 1H), 1.23 (d, J = 7.0 Hz, 3H), 0.98 (d, J = 6.3 Hz, 3H) ppm; ^{13}C NMR (100 MHz, $CDCl_3$): δ 144.0, 128.0 (2C), 127.5 (2C), 126.0, 71.9, 46.9, 20.7, 15.9 ppm.

2.1.2 (±)-*erythro*-3-(*p*-tolyl)-2-butanol (**2b**).

Colorless liquid. R_f = 0.50 (silica gel, 20% ethyl acetate in hexanes, visualized with *p*-anisaldehyde stain); ^{1}H NMR (400 MHz, $CDCl_3$): δ 7.07-6.88 (m, 4H), 3.77 (quint, J = 6.3 Hz, 1H), 2.62 (quint, J = 6.9 Hz, 1H), 2.24 (s, 3H), 1.47 (bs, 1H), 1.22 (d, J = 7.0 Hz, 3H), 0.99 (d, J = 6.3 Hz, 3H) ppm; ^{13}C NMR (100 MHz, $CDCl_3$): δ 140.7, 135.5, 128.7 (2C), 127.3 (2C), 71.9, 46.3, 20.6, 15.7 ppm.

2.1.3 (±)-*erythro*-3-(*o*-tolyl)-2-butanol (**2c**).

Colorless liquid. R_f = 0.50 (silica gel, 20% ethyl acetate in hexanes, visualized with *p*-anisaldehyde stain); ^{1}H NMR (400 MHz, $CDCl_3$): δ 7.12-7.06 (m, 3H), 7.05-7.01 (m, 1H), 3.83 (quint, J = 6.3 Hz, 1H), 2.93 (quint, J = 6.8 Hz, 1H), 2.27 (s, 3H), 1.22 (d, J = 6.9 Hz, 3H), 1.04 (d, J = 6.3 Hz, 3H) ppm; ^{13}C NMR (100 MHz, $CDCl_3$): δ 142.7, 135.2, 130.1, 125.8, 125.7, 125.6, 71.5, 41.3, 20.9, 19.6, 15.6 ppm.

2.1.4 (±)-*erythro*-3-([1,1'-biphenyl]-4-yl)-2-butanol (**2d**).

Colorless liquid. R_f = 0.50 (silica gel, 20% ethyl acetate in hexanes, visualized with *p*-anisaldehyde stain); ^{1}H NMR (400 MHz, $CDCl_3$): δ 7.55-7.42 (m, 4H), 7.40-7.32 (m, 2H), 7.29-7.23 (m, 1H), 7.23-7.16 (m, 2H), 3.85 (quint, J = 6.1 Hz, 1H), 2.72 (quint, J = 6.7 Hz, 1H), 1.42 (bs, 1H), 1.29 (d, J = 7.4 Hz, 3H), 1.06 (d, J = 6.5 Hz, 3H) ppm; ^{13}C NMR (100 MHz, $CDCl_3$): δ 142.9, 140.5, 138.9, 128.3 (2C), 127.9 (2C), 126.7 (3C), 126.6 (2C), 71.9, 46.4, 20.7, 15.5 ppm.

2.1.5 (±)-*erythro*-3-(4-methoxyphenyl)-2-butanol (**2e**).

Colorless liquid. R_f = 0.50 (silica gel, 20% ethyl acetate in hexanes, visualized with *p*-anisaldehyde stain); ^{1}H NMR (400 MHz, $CDCl_3$): δ 7.04 (dt, J = 8.5 and 2.1 Hz, 2H), 6.78 (dt, J = 8.6 and 2.2 Hz, 2H), 3.76 (quint, J = 6.2 Hz, 1H), 3.71 (s, 3H), 2.62 (quint, J = 6.8 Hz, 1H), 1.61 (bs, 1H), 1.22(d, J = 7.1 Hz, 3H), 1.00 (d, J = 6.3 Hz, 3H) ppm; ^{13}C NMR (100 MHz, $CDCl_3$): δ 157.7, 135.8, 128.3 (2C), 113.4 (2C), 72.1, 54.8, 45.8, 20.5, 15.7 ppm.

2.1.6 (±)-*erythro*-3-(4-chlorophenyl)-2-butanol (**2f**).

Colorless liquid. R_f = 0.50 (silica gel, 20% ethyl acetate in hexanes, visualized with *p*-anisaldehyde stain); ^{1}H NMR (400 MHz, $CDCl_3$): δ 7.23-7.17 (m, 2H), 7.11-7.03 (m, 2H), 3.77 (quint, J = 7.0 Hz, 1H), 2.64 (quint, J = 7.0 Hz, 1H), 1.63 (bs, 3H), 1.23 (d, J = 7.0 Hz, 3H), 1.01 (d, J = 6.2 Hz, 3H) ppm; ^{13}C NMR (100 MHz, $CDCl_3$): δ 142.3, 131.7, 128.8 (2C), 128.1 (2C), 71.8, 46.2, 20.7, 15.7 ppm.

2.1.7 (±)-*erythro*-3-(4-(methoxymethyl)phenyl)-2-butanol (**2g**).

Colorless liquid. R_f = 0.50 (silica gel, 20% ethyl acetate in hexanes, visualized with *p*-anisaldehyde stain); ^{1}H NMR (400 MHz, $CDCl_3$): δ 7.21 (d, J = 7.9 Hz, 2H), 7.13-7.09 (m, 2H), 4.35 (s, 2H), 3.79 (quint, J = 6.2 Hz, 1H), 3.32 (s, 3H), 2.66 (quint, J = 6.7 Hz, 1H), 1.50 (bs, 1H), 1.24 (d, J = 6.7 Hz, 3H), 1.00 (d, J = 6.2 Hz, 3H) ppm. ^{13}C NMR (100 MHz, $CDCl_3$): δ 143.3, 129.2, 127.6 (2C), 127.5 (2C), 74.1, 71.9, 57.7, 46.5, 20.6, 15.6 ppm.

2.1.8 (±)-*erythro*-3-(naphthalen-1-yl)-2-butanol (**2h**).

Colorless liquid. R_f = 0.50 (silica gel, 20% ethyl acetate in hexanes, visualized with *p*-anisaldehyde stain); ^{1}H NMR (400 MHz, $CDCl_3$): δ 8.03 (d, J = 8.3 Hz, 1H), 7.82-7.76 (m, 1H), 7.67 (d, J = 8.1 Hz, 1H), 7.47-7.31 (m, 4H), 4.02 (sext, J = 6.2 Hz, 1H), 3.60 (quint, J = 6.6 Hz, 1H), 1.47 (bs, 1H), 1.38 (d, J = 6.9 Hz, 3H), 1.13 (d, J = 6.3 Hz, 3H) ppm; ^{13}C NMR (100 MHz, $CDCl_3$): δ 140.3, 133.7, 131.3, 128.6, 126.5, 125.5, 125.0 (2C), 123.5, 122.7, 71.2, 40.1, 20.8, 14.8 ppm.

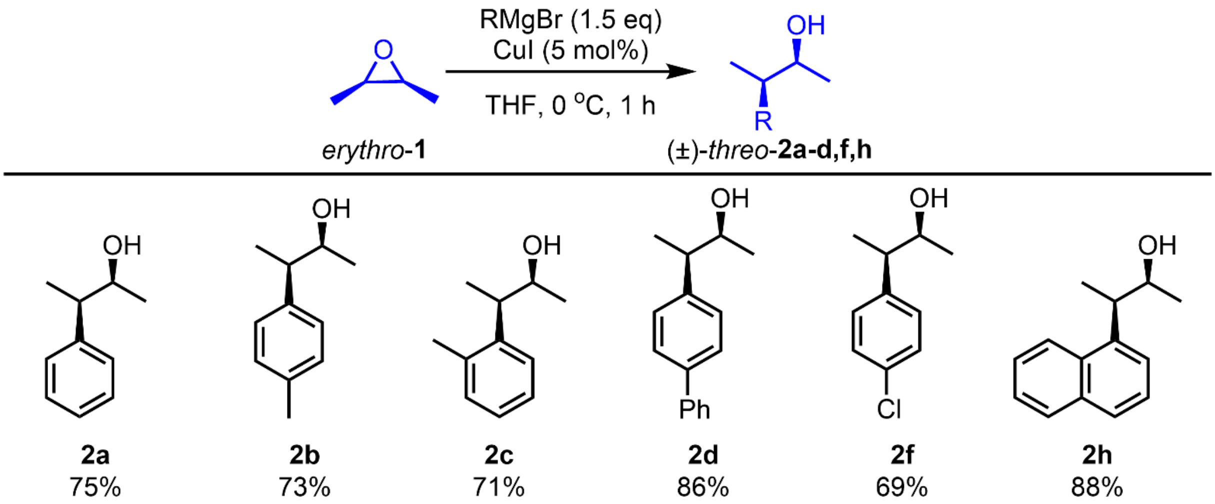

Scheme S2. Synthesis of (±)-*erythro*-3-aryl-2-butanol derivatives, **2a-d**, **2f**, and **2h**.

2.1.9 (±)-*threo*-3-phenyl-2-butanol (**2a**).

Colorless liquid. R_f = 0.50 (silica gel, 20% ethyl acetate in hexanes, visualized with *p*-anisaldehyde stain); ^{1}H NMR (400 MHz, $CDCl_3$): δ 7.30-7.21 (m, 2H), 7.18-7.12 (m, 3H), 3.81-3.72 (m, 1H), 2.59 (quint, J = 7.2 Hz, 1H), 1.57 (bs, 1H), 1.18 (d, J = 7.1 Hz, 3H), 1.15 (d, J = 6.2 Hz, 3H) ppm; ^{13}C NMR (100 MHz, $CDCl_3$): δ 143.2, 128.2 (2C), 127.6 (2C), 126.3, 72.0, 47.5, 20.1, 17.4; ppm.

2.1.10 (±)-*threo*-3-(*p*-tolyl)-2-butanol (**2b**).

Colorless liquid. R_f = 0.50 (silica gel, 20% ethyl acetate in hexanes, visualized with *p*-anisaldehyde stain); ^{1}H NMR (400 MHz, $CDCl_3$): δ 7.07 (s, 4H), 3.75 (quint, J = 5.9 Hz, 1H), 2.55 (quint, J = 6.9 Hz, 1H), 2.26 (s, 3H), 1.42 (bs, 1H), 1.17 (d, J = 7.2 Hz, 3H), 1.15 (d, J = 6.2 Hz, 3H) ppm; ^{13}C NMR (100 MHz, $CDCl_3$): δ 140.0, 135.9, 128.9 (2C), 127.4 (2C), 72.0, 47.2, 20.6, 20.2, 17.5 ppm.

2.1.11 (±)-*threo*-3-(*o*-tolyl)-2-butanol (**2c**).

Colorless liquid. R_f = 0.50 (silica gel, 20% ethyl acetate in hexanes, visualized with *p*-anisaldehyde stain); ^{1}H NMR (400 MHz, $CDCl_3$): δ 7.12-6.94 (m, 4H), 3.79-3.67 (m, 1H), 2.87 (quint, J = 7.1 Hz, 1H), 2.23 (s, 3H), 1.92 (bs, 1H), 1.11 (d, J = 6.2 Hz, 3H), 1.06 (d, J = 6.9 Hz, 3H) ppm; ^{13}C NMR (100 MHz, $CDCl_3$): δ 141.9, 136.4, 130.2, 126.0, 125.8, 125.6, 71.8, 42.1, 19.9, 19.6, 17.3; ppm.

2.1.12 (±)-*threo*-3-([1,1'-biphenyl]-4-yl)-2-butanol (**2d**).

Colorless liquid. R_f = 0.50 (silica gel, 20% ethyl acetate in hexanes, visualized with *p*-anisaldehyde stain); ^{1}H NMR (400 MHz, $CDCl_3$): δ 7.51-7.42 (m, 4H), 7.36-7.29 (m, 2H), 7.26-7.18 (m, 3H), 3.79 (sext, J = 6.2 Hz, 1H), 2.63 (quint, J = 7.2 Hz, 1H), 1.54 (bs, 1H), 1.20 (d, J = 7.1 Hz, 3H), 1.16 (d, J = 6.3 Hz, 3H) ppm; ^{13}C NMR (100 MHz, $CDCl_3$): δ 142.2, 140.4, 139.2, 128.3 (2C), 128.1 (2C), 126.9 (2C), 126.8, 126.6 (2C), 72.1, 47.2, 20.3, 17.5 ppm.

2.1.13 (±)-*threo*-3-(4-chlorophenyl)-2-butanol (**2f**).

Colorless liquid. R_f = 0.50 (silica gel, 20% ethyl acetate in hexanes, visualized with *p*-anisaldehyde stain); ^{1}H NMR (400 MHz, $CDCl_3$): δ 7.24-7.19 (m, 2H), 7.13-7.07 (m, 2H), 3.82-3.68 (m, 1H), 2.59 (quint, J = 6.9 Hz, 1H), 1.33 (bs, 1H), 1.17 (d, J = 7.0 Hz, 3H), 1.12 (d, J = 6.2 Hz, 3H) ppm; ^{13}C NMR (100 MHz, $CDCl_3$): δ 141.7, 131.9, 129.0 (2C), 128.2 (2C), 71.8, 46.8, 20.3, 17.4 ppm.

2.1.14 (±)-*threo*-3-(naphthalen-1-yl)-2-butanol (**2h**).

Colorless liquid. R_f = 0.50 (silica gel, 20% ethyl acetate in hexanes, visualized with *p*-anisaldehyde stain); ^{1}H NMR (400 MHz, $CDCl_3$): δ 8.26-8.22 (m, 1H), 8.04-7.99 (m, 1H), 7.69 (d, J = 7.9 Hz, 1H), 7.59-7.48 (m, 3H), 7.22 (d, J = 7.9 Hz, 1H), 4.50-4.42 (m, 1H), 4.10-4.02 (m, 1H), 1.49 (d, J = 6.8 Hz, 3H), 1.46 (d, J = 7.0 Hz, 3H) ppm; ^{13}C NMR (100 MHz, $CDCl_3$): δ 138.7, 132.4, 131.8, 129.0, 127.9, 126.7, 126.6, 124.0, 122.9 (2C), 53.5, 41.3, 20.7, 14.6 ppm.

2.2 General synthesis of (±)-*erythro*- and (±)-*threo*-**5a-h**

To an oven-dried 25 mL round bottom flask filled with 1,2-dichloroethane (DCE) (2 mL) under nitrogen atmosphere, (±)-*erythro*- or (±)-*threo*-**2a-h** (0.33 mmol) and propyl methyl thiourea (PMTU) (0.3 equiv) were added and the solution was stirred at *RT* till PMTU was completely dissolved in DCE. After 15 min, *N*-bromosuccinimide (NBS) (2.3 equiv) was added in two equal portions to the reaction mixture. The first portion was added to the reaction mixture and allowed to stir for 30 min. and then, a second portion was introduced to the reaction mixture and stirred at *RT* until the alcohol disappeared according to TLC and GC (5-6 h). Then, the reaction mixture was quenched with saturated aqueous ammonium chloride solution and extracted with EDC (3×3 mL). The organic extracts were combined, dried over anhydrous sodium sulfate and concentrated. The crude product was purified by silica gel column chromatography using hexane/ethyl acetate (9.5:0.5) as eluent to afford the products in 56-85% yield.

2.2.1 (±)-*erythro*-3-phenyl-2-bromobutane, (±)-*erythro*-**5a**.

Colorless liquid. R_f = 0.80 (silica gel, 5% ethyl acetate in hexanes, visualized with *p*-anisaldehyde stain); ^{1}H NMR (400 MHz, $CDCl_3$): δ 7.35-7.29 (m, 2H), 7.27-7.23 (m, 1H), 7.22-7.19 (m, 2H), 4.33-4.22 (m, 1H), 2.87 (quint, J = 7.3 Hz, 1H), 1.49 (d, J = 6.7 Hz, 3H), 1.39 (d,

$J = 6.9$ Hz, 3H) ppm; ^{13}C NMR (100 MHz, $CDCl_3$): δ 143.1, 128.1 (2C), 127.1 (2C), 126.4, 57.3, 48.2, 24.8, 19.6 ppm.

2.2.2 (±)-*threo*-3-phenyl-2-bromobutane, (±)-*threo*-**5a**.

Colorless liquid. R_f = 0.80 (silica gel, 5% ethyl acetate in hexanes, visualized with *p*-anisaldehyde stain); ^{1}H NMR (400 MHz, $CDCl_3$): δ 7.27-7.21 (m, 2H), 7.20-7.14 (m, 3H), 4.35-4.26 (m, 1H), 3.06 (quint, $J = 6.7$ Hz, 1H), 1.55 (d, $J = 6.6$ Hz, 3H), 1.33 (d, $J = 7.1$ Hz, 3H) ppm; ^{13}C NMR (100 MHz, $CDCl_3$): δ 142.5, 127.8 (2C), 127.6 (2C), 126.4, 6.1, 47.1, 22.2, 16.9 ppm.

2.2.3 (±)-*erythro*-3-(*p*-tolyl)-2-bromobutane, (±)-*erythro*-**5b**.

Colorless liquid. R_f = 0.80 (silica gel, 5% ethyl acetate in hexanes, visualized with *p*-anisaldehyde stain); ^{1}H NMR (400 MHz, $CDCl_3$): δ 7.08-6.99 (m, 4H), 4.20-4.13 (m, 1H), 2.89-2.80 (m, 1H), 2.25 (s, 3H), 1.49 (d, $J = 6.7$ Hz, 3H), 1.37 (d, $J = 6.9$ Hz, 3H) ppm; ^{13}C NMR (100 MHz, $CDCl_3$): δ 140.1, 136.0, 128.7 (2C), 126.9 (2C), 57.5, 47.8, 24.8, 20.6, 19.6 ppm.

2.2.4 (±)-*threo*-3-(*p*-tolyl)-2-bromobutane, (±)-*threo*-**5b**.

Colorless liquid. R_f = 0.50 (silica gel, 20% ethyl acetate in hexanes, visualized with *p*-anisaldehyde stain); ^{1}H NMR (400 MHz, $CDCl_3$): δ 7.15 (s, 4H), 4.41-4.34 (m, 1H), 3.12 (quint, J = 5.6 Hz, 1H), 2.35 (s, 3H), 1.63 (d, J = 6.8 Hz, 3H), 1.41 (d, J = 7.0 Hz, 3H) ppm; ^{13}C NMR (100 MHz, $CDCl_3$): δ 139.5, 136.0, 128.5 (2C), 127.5 (2C), 56.4, 46.7, 22.2, 20.6, 16.9 ppm.

2.2.5 (±)-*erythro*-3-(*o*-tolyl)-2-bromobutane, (±)-*erythro*-**5c**.

Colorless liquid. R_f = 0.80 (silica gel, 5% ethyl acetate in hexanes, visualized with *p*-anisaldehyde stain); ^{1}H NMR (400 MHz, $CDCl_3$): δ 7.14-7.01 (m, 4H), 4.22 (quint, $J = 6.7$ Hz, 1H), 3.15 (sext, $J = 6.8$ Hz, 1H), 2.27 (s, 3H), 1.49 (d, $J = 6.7$ Hz, 3H), 1.36 (d, $J = 6.8$ Hz, 3H) ppm; ^{13}C NMR (100 MHz, $CDCl_3$): δ 141.8, 135.0, 130.2, 125.99, 125.89, 125.4, 57.4, 43.0, 24.7, 20.2, 19.6 ppm.

2.2.6 (±)-*threo*-3-(*o*-tolyl)-2-bromobutane, (±)-*threo*-**5c**

Colorless liquid. R_f = 0.50 (silica gel, 20% ethyl acetate in hexanes, visualized with *p*-anisaldehyde stain); ^{1}H NMR (400 MHz, $CDCl_3$): δ 7.18-7.04 (m, 4H), 4.27 (quint, J = 6.5 Hz, 1H), 3.41 (quint, J = 6.9 Hz, 1H), 2.30 (s, 3H), 1.60 (d, J = 6.7 Hz, 3H), 1.29 (d, J = 6.9 Hz, 3H) ppm; ^{13}C NMR (100 MHz, $CDCl_3$): δ 141.7, 135.5, 130.1, 126.1, 125.7, 125.5, 54.6, 42.6, 21.6, 19.3, 15.9 ppm.

2.2.7 (±)-*erythro*-3-([1,1'-biphenyl]-4-yl)-2-bromobutane, (±)-*erythro*-**5d**.

Colorless liquid. R_f = 0.80 (silica gel, 5% ethyl acetate in hexanes, visualized with *p*-anisaldehyde stain); ^{1}H NMR (400 MHz, $CDCl_3$): δ 7.54-7.44 (m, 4H), 7.39-7.32 (m, 2H), 7.29-7.23 (m, 1H), 7.23-7.18 (m, 2H), 4.23 (sext, $J = 6.7$ Hz, 1H), 2.93 (quint, $J = 7.0$ Hz, 1H), 1.54 (d, $J = 6.7$ Hz, 3H), 1.42 (d, $J = 6.7$ Hz, 3H) ppm; ^{13}C NMR (100 MHz, $CDCl_3$): δ 142.2, 140.3, 139.4, 128.3 (2C), 127.5 (2C), 126.8, 126.7 (2C), 126.6 (2C), 57.2, 47.8, 24.8, 19.5 ppm.

2.2.8 (±)-*threo*-3-([1,1'-biphenyl]-4-yl)-2- bromobutane, (±)-*threo*-**5d**.

Colorless liquid. R_f = 0.50 (silica gel, 20% ethyl acetate in hexanes, visualized with *p*-anisaldehyde stain); ^{1}H NMR (400 MHz, $CDCl_3$): δ 7.55-7.44 (m, 4H), 7.40-7.32 (m, 2H), 7.29-

7.21 (m, 3H), 4.34 (quint, J = 6.8 Hz, 1H), 3.11 (quint, J = 5.6 Hz, 1H), 1.58 (d, J = 6.8 Hz, 3H), 1.37 (d, J = 7.2 Hz, 3H) ppm; ^{13}C NMR (100 MHz, CDCl3): δ 142.9, 141.8, 140.7, 129.7, 129.4, 128.1, 128.0 (2C), 127.9 (2C), 57.4, 48.1, 23.6, 18.4 ppm.

2.2.9 (±)-*erythro*-3-(4-methoxyphenyl)-2-bromobutane, (±)-*erythro*-**5e**.

Colorless liquid. R_f = 0.80 (silica gel, 5% ethyl acetate in hexanes, visualized with *p*-anisaldehyde stain); ^{1}H NMR (400 MHz, $CDCl_3$): δ 7.31 (d, J = 2.2 Hz, 1H), 7.04 (dd, J = 8.5 and 2.2 Hz, 1H), 6.81-6.74 (m, 1H), 4.13 (sext, J = 6.7 Hz, 1H), 3.81 (s, 3H), 2.82 (quint, J = 7.0 Hz, 1H), 1.51 (d, J = 6.9 Hz, 3H), 1.35 (d, J = 6.9 Hz, 3H) ppm; ^{13}C NMR (100 MHz, $CDCl_3$): δ 154.3, 136.7, 131.9, 128.0, 127.1, 111.4, 56.9, 55.8, 46.9, 24.6, 19.0 ppm.

2.2.10 (±)-*erythro*-3-(4-chlorophenyl)-2-bromobutane, (±)-*erythro*-**5f**.

Colorless liquid. R_f = 0.80 (silica gel, 5% ethyl acetate in hexanes, visualized with *p*-anisaldehyde stain); ^{1}H NMR (400 MHz, $CDCl_3$): δ 7.24-7.15 (m, 2H), 7.09-7.03 (m, 2H), 4.14 (sext, J = 6.7 Hz, 1H), 2.86 (quint, J = 6.9 Hz, 1H), 1.50 (d, J = 6.8 Hz, 3H), 1.35 (d, J = 6.8 Hz, 3H) ppm; ^{13}C NMR (100 MHz, $CDCl_3$): δ 141.5, 132.2, 128.5 (2C), 128.2 (2C), 56.6, 47.4, 24.5, 19.0 ppm.

2.2.11 (±)-*threo*-3-(4-chlorophenyl)-2-bromobutane ((±)-*threo*-**5f**.

Colorless liquid. R_f = 0.50 (silica gel, 20% ethyl acetate in hexanes, visualized with *p*-anisaldehyde stain); ^{1}H NMR (400 MHz, $CDCl_3$): δ 7.24-7.19 (m, 2H), 7.14-7.08 (m, 2H), 4.29-4.21 (m, 1H), 3.0 (quint, J = 6.9 Hz, 1H), 1.55 (d, J = 7.1 Hz, 3H), 1.31 (d, J = 7.1 Hz, 3H) ppm; ^{13}C NMR (100 MHz, $CDCl_3$): δ 142.3, 131.8, 130.4 (2C), 129.3 (2C), 57.1, 47.9, 23.9, 18.9 ppm.

2.2.12 (±)-*erythro*-3-(4-(caboxaldehyde)phenyl)-2-bromobutane, (±)-*erythro*-**5g**.

Colorless liquid. R_f = 0.60 (silica gel, 10% ethyl acetate in hexanes, visualized with *p*-anisaldehyde stain); ^{1}H NMR (400 MHz, $CDCl_3$): δ 9.92 (s, 1H), 7.77 (dt, J = 8.3 and 1.8 Hz, 2H), 7.33-7.28 (m, 2H), 4.21 (quint, J = 7.1 Hz, 1H), 2.98 (quint, J = 6.6 Hz, 1H), 1.53 (d, J = 6.8 Hz, 3H), 1.41 (d, J = 6.8 Hz, 3H) ppm; ^{13}C NMR (100 MHz, $CDCl_3$): δ 191.4, 150.2, 134.9, 129.5 (2C), 127.9 (2C), 55.9, 48.1, 24.6, 18.7 ppm.

2.2.13 (±)-*erythro*-3-(naphthalen-1-yl)-2-bromobutane, (±)-*erythro*-**5h**.

Colorless liquid. R_f = 0.80 (silica gel, 5% ethyl acetate in hexanes, visualized with *p*-anisaldehyde stain); ^{1}H NMR (400 MHz, $CDCl_3$): δ 8.29-8.19 (m, 1H), 8.03-7.92 (m, 1H), 7.70 (d, J = 7.8 Hz, 1H), 7.59-7.43 (m, 3H), 7.17 (d, J = 2.8 Hz, 1H), 4.38 (quint, J = 6.9 Hz, 1H), 3.71 (quint, J = 6.9 Hz, 1H), 1.58 (d, J = 6.9 Hz, 3H), 1.50 (d, J = 6.9 Hz, 3H) ppm; ^{13}C NMR (100 MHz, $CDCl_3$): δ 139.7, 132.3, 131.8, 129.2, 127.9, 126.6, 124.1, 122.7, 56.9, 41.3, 25.0, 18.8 ppm.

2.2.14 (±)-*threo*-3-(naphthalen-1-yl)-2-bromobutane, (±)-*threo*-**5h**.

Colorless liquid. R_f = 0.50 (silica gel, 20% ethyl acetate in hexanes, visualized with *p*-anisaldehyde stain); ^{1}H NMR (400 MHz, $CDCl_3$): δ 8.26-8.22 (m, 1H), 8.04-7.99 (m, 1H), 7.69 (d, J = 7.9 Hz, 1H), 7.59-7.48 (m, 3H), 7.22 (d, J = 7.9 Hz, 1H), 4.50-4.42 (m, 1H), 4.10-4.02 (m, 1H), 1.49 (d, J = 6.8 Hz, 3H), 1.46 (d, J = 7.0 Hz, 3H) ppm; ^{13}C NMR (100 MHz, $CDCl_3$): δ 138.7, 132.4, 131.8, 129.0, 127.9, 126.7, 126.6, 124.0, 122.9 (2C), 53.5, 41.3, 20.7, 14.6 ppm.

2.3 Synthesis of (±)-**5i**

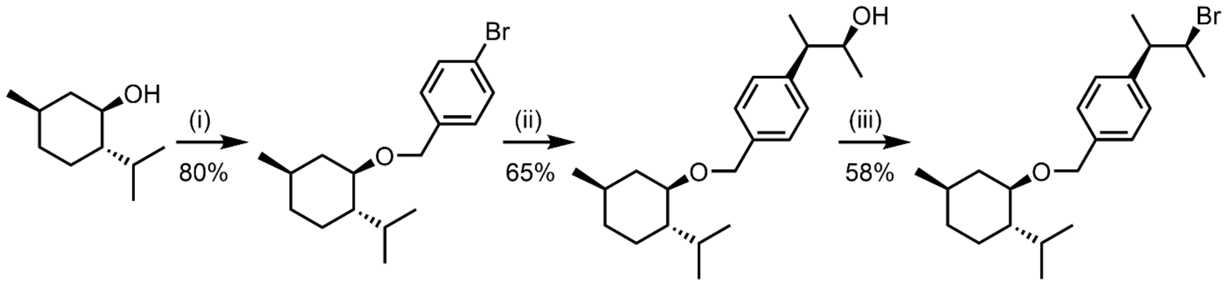

(−)-menthol 4-bromobenzyl (−)-menthyl ether (±)-*threo*-**2i** (±)-*threo*-**5i**

Scheme S3. Synthesis of (±)-*threo*-**2i** and **5i**. Reaction conditions: (i) 1-bromo-4-(bromomethyl)benzene (1.5 equiv), NaH (6.5 equiv) dry THF, reflux, 12 h; (ii) Mg (1.5 eq), CuI (0.05 equiv), dry THF, 50 °C to RT, then adding (2R,3S)-2,3-dimethyl oxirane (1 equiv); (iii) NBS (2.3 equiv), PMTU (0.3 equiv), DCE, *RT*, 3 h.

2.3.1 Synthesis of 4-bromobenzyl (-)menthyl ether

An oven-dried 50 mL round bottom flask was charged with NaH (199.6 mg, 8.32 mmol). The flask was sealed with a rubber septum, and a vacuum was applied to remove air present inside the flask over 10 min. Then, the flask was refilled with nitrogen gas and dry THF (10 mL) was added under N_2 atmosphere. Next, (-)-menthol (200 mg, 1.28 mmol) in dry THF solution (4 mL) was added dropwise over 15 min, and then the reaction mixture was stirred for 30 min at room temperature. After 30 min, 1-bromo-4-(bromomethyl)benzene (383.9 mg, 1.54 mmol) was dissolved in dry THF (4 mL) and added dropwise to the reaction mixture over 10 min. The entire reaction mixture was stirred at reflux temperature for an additional 11 h. TLC monitored the reaction progress. Upon complete consumption of the starting compound, the reaction mixture was then quenched with saturated aqueous ammonium chloride solution at 0 oC and extracted with ethyl acetate. The organic extracts were combined, dried over anhydrous sodium sulfate and concentrated. The crude product was purified by silica gel column chromatography using 5% ethyl acetate in hexane as eluent to afford the desired product as a pale yellow solid (335 mg) in 80% yield.

^{1}H NMR (400 MHz, CDCl3): δ 7.38 (dd, ^3J = 6.4 and ^4J = 2.4 Hz, H_a, 2H), 7.15 (dd, ^3J = 6.4 and ^4J = 2.4 Hz, H_b, 2H), 4.53 (d, ^2J = 11.6 Hz, H_c, 1H), 4.27 (d, ^2J = 11.6 Hz, $H_{c'}$, 1H), 3.08 (td, ^3J = 10.8 and 4.0 Hz, H_d, 1H), 2.20 (m, H_e, 1H), 2.09 (m, $H_{e'}$, 1H), 1.55 (m, 2H), 1.22 (m, 3H), 0.88-0.80 (m, 10H), 0.64 (d, J = 6.8 Hz, 3H) ppm.

2.3.2 Synthesis of (±)-*threo*-**2i**

Mg turnings (50.0 mg, 2.08 mmol) and a catalytic amount of I_2 (17.8 mg, 0.07 mmol) were placed in an oven-dried 50 mL round bottom flask. The flask was sealed with a rubber septum, and a vacuum was applied to remove air from the flask over 10 min. Then, the flask was refilled with N_2 gas and heated gently by a heat gun until the evolution of purple fumes (Iodine color) was observed. After that, dry THF (10 mL) and 4-bromobenzyl (-)menthyl ether (678.2 mg, 2.08 mmol) solution in THF (10 mL) were simultaneously slowly added to the flask by two separate syringes under the nitrogen atmosphere over 5 min. The whole mixture was stirred at 50 °C for 1.5 h until the disappearance of Mg turnings. In parallel, another oven-dried 50 mL round bottom flask was charged with (2R,3S)-2,3-dimethyloxirane (100 mg, 1.39 mmol) and CuI (13.3 mg,

0.07 mmol) and closed with a rubber septum. The inert atmosphere inside the flask was kept by purging N_2 gas. Dry THF (10 mL) was added to the reaction mixture, and the flask was kept at 0 °C under stirring. The Grignard solution generated in the first flask was taken out by syringe and immediately added dropwise to the reaction mixture of the second flask while the temperature was kept at 0 °C over 5 min. TLC monitored the reaction progress. Upon complete consumption of the starting compound (~1 h), the reaction mixture was then quenched with saturated aqueous ammonium chloride solution at 0 ºC and extracted with ethyl acetate. The organic extracts were combined, dried over anhydrous sodium sulfate and concentrated under reduced pressure. The crude product was purified by silica gel column chromatography using 20% ethyl acetate in hexane as eluent to afford compound **2i** as a colorless liquid (64 mg) in 65% yield.

^{1}H NMR (400 MHz, CDCl3): δ 7.25 (d, ^3J = 8.0 Hz, H_a, 2H), 7.14 (d, ^3J = 8.4 Hz, H_b, 2H), 4.56 (d, ^2J = 11.2 Hz, H_c, 1H), 4.29 (d, ^2J = 11.2 Hz, $H_{c'}$, 1H), 3.75 (m, H_n, 1H), 3.10 (td, J = 10.4 and 2.8 Hz, H_d, 1H), 2.59 (quint, ^3J = 7.2 Hz, H_m, 1H), 2.27-2.06 (m, H_e, 2H), 1.63-1.49 (m, 2H), 1.34-1.24 (m, 2H), 1.17 (d, J = 7.2 Hz, 3H), 1.15 (d, J = 6.4 Hz, 3H), 0.92-0.76 (m, 10H), 0.63 (dd, J = 7.2 and 1.6 Hz, 3H) ppm; **^{13}C NMR** (125 MHz, CDCl3): δ 142.3 (C_{quat}), 137.1 (C_{quat}), 127.8, 127.5 (4CH_{aryl}), 78.3 (C_d), 71.9 (C_n), 69.8 (C_c), 47.8 (C_j), 47.3 (C_m), 39.9 (C_e), 34.1 (C_h), 31.1(C_g) , 25.1 (C_k), 22.8 (C_i), 21.9 (C_o), 20.5, and 20.1 ($C(CH_3)_2$), 17.5 (C_f), 15.6 (C_l) ppm.

2.3.3 Synthesis of (±)-*threo*-**5i**

To an oven-dried 25 mL round bottom flask filled with dichloroethane (DCE) (2 mL) under nitrogen atmosphere, (±)-*threo*-**2i** (50 mg, 0.157 mmol) and propyl methyl thiourea (PMTU) (0.3 equiv) were added and the solution was stirred at *RT* till PMTU was completely dissolved in DCE. After 15 min, *N*-bromosuccinimide (NBS) (2.3 equiv) was added in two equal portions to the reaction mixture. The first portion was added to the reaction mixture and allowed to stir for 30 min. and then, a second portion was introduced to the reaction mixture and stirred at *RT* until the alcohol disappeared according to TLC and GC (5-6 h). Then, the reaction mixture was quenched with saturated aqueous ammonium chloride solution and extracted with EDC (3×3 mL). The organic extracts were combined, dried over anhydrous sodium sulfate and concentrated. The crude product was purified by silica gel column chromatography using hexane/ethyl acetate (9.5:0.5) as eluent to afford (±)-*threo*-**5i** as a colorless liquid (35 mg) in 58% yield.

^{1}H NMR (400 MHz, CDCl3): δ δ 7.23 (d, ^3J = 8.0 Hz, H_a, 2H), 7.14 (d, ^3J = 8.4 Hz, H_b, 2H), 4.56 (d, ^2J = 11.6 Hz, H_c, 1H), 4.29 (d, ^2J = 11.6 Hz, $H_{c'}$, 1H), 4.33-4.25 (m, H_n, 1H), 3.16-2.98 (m, H_d + H_m, 2H), 2.24-2.10 (m, H_e, 2H), 1.56 (m, 2H), 1.53 (d, ^3J = 6.8 Hz, 3H), 1.32 (d, J = 7.2 Hz, 3H), 1.19 (m, 2H), 0.92-0.64 (m, 10H), 0.63 (dd, ^3J = 6.8 and 2.8 Hz, 3H) ppm; **^{13}C NMR** (125 MHz, CDCl3): δ 141.6 (C_{quat}), 137.2 (C_{quat}), 127.5 and 127.4 (4CH_{aryl}), 78.3 (C_d), 69.7 (C_c), 56.0 (C_n), 47.8 (C_j), 46.8 (C_m), 39.9 (C_e), 34.1 (C_h), 31.1 (C_g), 25.0 (C_k), 22.8 (C_i), 22.1 (C_o), 21.9 (C_f), 20.6 and 16.9 ($C(CH_3)_2$), 15.6 (C_l) ppm.

2.4 Stereoisomer formation in *E*2 reactions of mesylated (±)-*erythro*- and (±)-*threo*-**2a.**

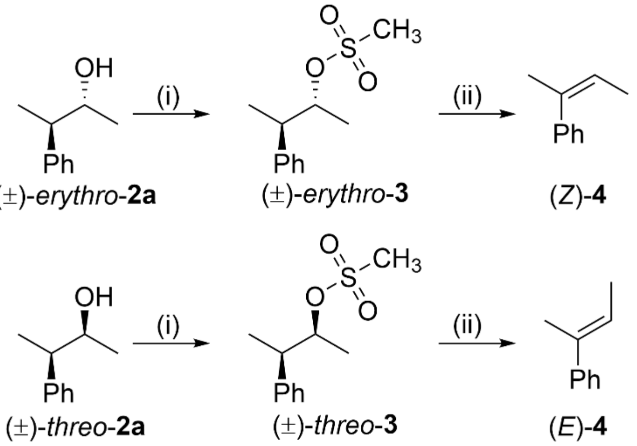

Scheme S4. Stereoselective synthesis of alkene **4** via mesylation (i) and elimination (ii) reactions of 3-phenyl-2-butanol diastereomers.

2.4.1. (±)-*erythro- and threo*-3-phenyl-2-butyl methanesulfonate, **3**.

To a 50 mL round bottom flask, a solution of either (±)-*erythro*- or *threo*-**2a** (100 mg, 0.67 mmol) in dry DCM (10 mL) was introduced. Then, pyridine (0.1 mL, 0.80 mmol) was added dropwise over 10 min., and the solution was kept stirring at room temperature. After 30 min, the flask was immersed in an ice bath, keeping the solution cold at 0 °C and methane sulfonyl chloride (92.1 mg, 0.80 mmol) was added dropwisely. Then, the ice bath was removed, and the reaction mixture was allowed to stir at room temperature until the alcohol was consumed (according to TLC and GC). The reaction mixture was extracted with dichloromethane (5x3 mL) after adding saturated aqueous ammonium chloride solution. The organic extracts were combined, dried over anhydrous sodium sulfate and concentrated under reduced pressure. The crude product was purified by silica gel column chromatography using 10% ethyl acetate in hexane as eluent to afford (±)-*erythro*-**3**- or (±)-*threo*-**3**, respectively.

(±)-*erythro*-**3**.

Colorless oil (135 mg) in 88% yield. $\mathbf{R_f}$ = 0.50 (silica gel, 10% EtOAc in hexanes, visualized with *p*-anisaldehyde stain); ^{1}H NMR (400 MHz, CDCl3): δ 6.53-6.45 (m, 2H), 6.44-6.40 (m, 3H), 4.01 (quint, J = 6.4 Hz, 1H), 2.15 (quint, J = 6.8 Hz, 1H), 1.89 (s, 3H), 0.57 (d, J = 6.8 Hz, 3H), 0.52 (d, J = 6.4 Hz, 3H)ppm; ^{13}C NMR (100 MHz, CDCl3): δ 141.8, 128.2 ($2C_{ortho}$), 127.5 ($2C_{meta}$), 126.7 (C_{para}), 83.9 (COMs), 44.8 (C_{benzyl}), 37.6 (CH_3SO_3), 19.4, 15.7 ppm.

(±)-*threo*-**3**

Colorless oil (100 mg) in 78% yield. $\mathbf{R_f}$ = 0.50 (silica gel, 10% EtOAc in hexanes, visualized with *p*-anisaldehyde stain); ^{1}H NMR (400 MHz, CDCl3): δ 7.27-7.24 (m, 2H), 7.19-7.15 (m, 3H), 4.73 (m, 1H), 2.92 (quint, J = 7.2 Hz, 1H), 2.35 (s, 3H), 1.36 (d, J = 6.4 Hz, 3H), 1.27 (d, J = 7.2 Hz, 3H)ppm; ^{13}C NMR (100 MHz, CDCl3): δ 142.0, 128.1 ($2C_{ortho}$), 127.6 ($2C_{meta}$), 126.6 (C_{para}), 84.3 (COMs), 44.8 (C_{benzyl}), 37.2 (CH_3SO_3), 18.9, 16.1 ppm.

2.4.2. (*E*)- *and* (*Z*)-2-phenyl-2-butene, **4**.

In a 25 mL round bottom flask, (±)-*erythro* or (±)-*threo*-**3** (100 mg, 4.38 mmol) was dissolved in a mixture of 25% dichloromethane in methanol (10 mL) and KOH (1.2 equiv) was added and

then stirred under reflux till **3** was fully consumed (~3 h, indicated by TLC or GC). Then, the reaction mixture was extracted with ethyl acetate (3×5 mL) after adding saturated aqueous ammonium chloride solution. The organic extracts were combined, dried over anhydrous sodium sulfate and concentrated under reduced pressure. The crude product was purified by silica gel column chromatography using 5% ethyl acetate in hexane as eluent to afford (*E*)-**4** or (*Z*)-**4**, respectively.

(*E*)-**4**.

Colorless oil (49 mg) in 85% yield. $\mathbf{R_f}$ = 0.50 (silica gel, 1% EtOAc in hexanes, visualized with *p*-anisaldehyde stain); ^{1}H NMR (400 MHz, CDCl3): δ 7.29-7.21 (m, 2H), 7.20-7.19 (m, 2H), 7.14-7.10 (m, 1H), 5.78 (m, 1H), 1.94 (m, 3H), 1.71 (dd, J = 6.8 and 1.2 Hz, 3H) ppm; ^{13}C NMR (100 MHz, CDCl3): δ 143.6, 135.1 (Ph(Me)**C**=), 127.7 ($2C_{meta}$), 125.9 (C_{para}), 125.1 ($2C_{ortho}$), 122.0 (Me**C**=), 15.0, 13.9

(*Z*)-**4**.

Colorless oil (48 mg) in 84% yield. $\mathbf{R_f}$ = 0.50 (silica gel, 1% EtOAc in hexanes, visualized with *p*-anisaldehyde stain); ^{1}H NMR (400 MHz, CDCl3): δ 7.24-7.21 (m, 2H), 7.14-7.08 (m, 3H), 5.47 (m, 1H), 1.93 (quint, J = 1.2 Hz, 3H), 1.49 (dq, J = 6.8 and 1.6 Hz, 3H) ppm; ^{13}C NMR (100 MHz, CDCl3): δ 141.5, 136.4 (Ph(Me)**C**=), 127.7 ($2C_{meta}$), 127.6 ($2C_{ortho}$), 125.9 (C_{para}), 121.2 (Me**C**=), 25.0, 14.5.

2.5 Bromination of (±)-*erythro*-2-phenyl-3-pentanol.

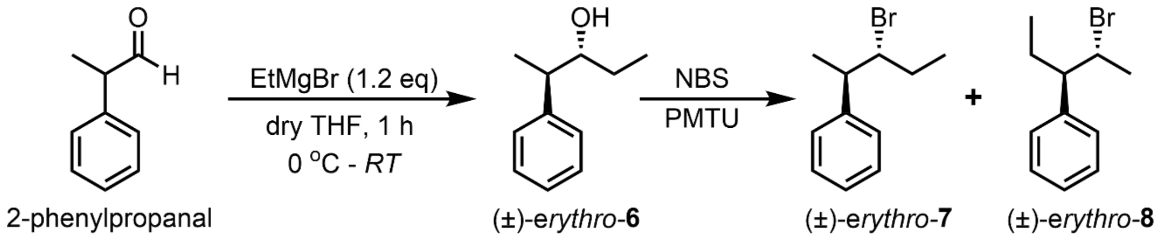

Scheme S5. Synthesis of (±)-*erythro*-**6** and its brominated products.

2.5.1 Synthesis of (±)-*erythro*-2-phenyl-3-pentanol, (±)-*erythro*-**6**:

To an oven-dried round-bottom flask containing a magnetic stirrer bar, Mg metal (0.455 g, 18.83 mmol) and anhydrous THF (50 mL) were added under a nitrogen atmosphere, and the flask was sealed with a rubber septum and cooled to 0 °C. Then, ethyl bromide (2.00 g, 18.83 mmol) was added dropwise over 5 min through the septum, and the reaction mixture was stirred for an additional hour to ensure complete *in situ* formation of the Grignard reagent. Next, 2-phenyl propanal (2 g, 14.91 mmol) was added dropwise over 10 min, and the reaction mixture was stirred at room temperature. TLC monitored the reaction progression. Upon complete consumption of the starting material, the reaction mixture was then quenched with saturated aqueous ammonium chloride solution and extracted with THF. The organic extracts were combined, dried over anhydrous sodium sulfate and then concentrated. The crude product was purified by silica gel column chromatography using 10% ethyl acetate in hexane as eluent to afford 1.85 g of (±)-*erythro*-2-phenyl-3-pentanol, **6** as colorless oil in 75% yield.

R_f = 0.50 (silica gel, 20% ethyl acetate in hexanes, visualized with *p*-anisaldehyde stain); ^{1}H NMR (400 MHz, $CDCl_3$): δ 7.35-7.32 (m, 2H), 7.25-7.22 (m, 3H), 3.62 (m, 1H), 7.59-7.48 (q, ^{3}J = 14 and 7.1 Hz,1H), 1.47 (m, 2H), 1.32 (d, ^{3}J = 7.1 H, 3H), 0.96 (d, ^{3}J = 7.4 Hz, 3H) ppm; ^{13}C NMR (100 MHz, $CDCl_3$): δ 144.8 (C_{quat}), 128.6 ($2C_{metq}$), 127.9 ($2C_{ortho}$), 126.5 (C_{para}) 77.9 (CH-OH); 45.4 (CH) 27.7 (CH_3), 15.6 (CH_3).

2.5.2 Synthesis of (±)-*erythro*-**7** and (±)-*erythro*-**8**.

The starting material, (±)-*erythro*-**6** (100 mg, 0.61 mmol), was dissolved in dichloroethane (4 mL) in an oven-dried 25 mL round bottom flask, and PMTU (24.2 mg, 0.18 mmol) was added, and the mixture was stirred at *RT* until PMTU was completely dissolved. Then, NBS (249 mg, 1.4 mmol) was added in two portions to the reaction mixture. The first portion of NBS (125 mg) was added to the reaction mixture and allowed to stir for 30 min. at *RT.* After 30 min, the second portion of NBS (124 mg) was introduced to the reaction mixture and stirred at *RT* until the alcohol **6** disappeared, as indicated by GC-MS. The reaction mixture was quenched with saturated aqueous ammonium chloride solution and extracted with DCM (3x5 mL). The organic extracts were combined, dried over anhydrous sodium sulfate and concentrated. The crude product was purified by preparative TLC using 1% ethyl acetate in hexane as eluent to afford (±)-*erythro*-2-phenyl-3-bromopentane, (±)-*erythro*-**7** (43.8% yield), and (±)-*erythro*-3-phenyl-2-bromopentane, (±)-*erythro*-**8** (56.2% yield) as a colorless oil (105 mg) in 75% overall yield.

(±)-*erythro*-2-phenyl-3-bromopentane, (±)-*erythro*-**7**.

^{1}H NMR (400 MHz, $CDCl_3$): δ 7.32-7.13 (m, 5H), 4.13 (m, 1H), 3.03 (m, 1H), 1.75 + 1.65 (m, 2H), 1.45 (d, ^{3}J = 5.5 Hz, 3H), 1.01 (t, ^{3}J = 5.8 Hz, 3H) ppm; ^{13}C NMR (100 MHz, $CDCl_3$): δ 144.2 (C_{quat}), 128.5 ($2C_{meta}$), 127.1 (C_{para}), 126.9 ($2C_{ortho}$) 67.3 (CH-Br); 46.6 (CH) 30.6 (CH_2), 25.2 (CH_3), 12.5 (CH_3) ppm.

(±)-*erythro*-3-phenyl-2-bromopentane, (±)-*erythro*-**8**.

^{1}H NMR (400 MHz, $CDCl_3$): δ 7.32-7.13 (m, 5H), 4.27 (m, 1H), 2.68 (m, 1H), 2.21 + 1.63 (m, 2H), 1.51 (d, ^{3}J = 5.4 Hz, 3H), 0.73 (t, ^{3}J = 5.9 Hz, 3H) ppm; ^{13}C NMR (100 MHz, $CDCl_3$): δ 141.5 (C_{quat}), 128.6 ($2C_{meta}$), 127.1 (C_{para}), 127.0 ($2C_{ortho}$) 56.9 (CH-Br); 56.5 (CH) 27.6 (CH_2), 19.9 (CH_3), 12.2 (CH_3) ppm.

2.6 Stereochemical *E*2 reaction of mesylated (±)-*erythro*-2-phenyl-3-pentanol.

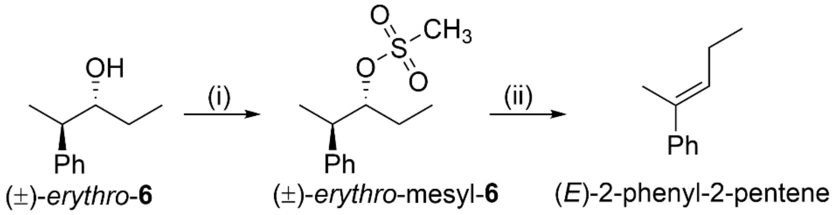

Scheme S6. Stereochemical *E*2 reaction of (±)-*erythro*-**6** after mesylation and elimination.

2.6.1 Synthesis of (±)-*erythro*-2-phenyl-3-pentyl methanesulfonate.

To a 50 mL round bottom flask, a solution of either (±)-*erythro*-**6** (150 mg, 0.91 mmol) in dry DCM (10 mL) was introduced. Then, pyridine (1.2 equiv) was added dropwise over 10 min., and the solution was kept stirring at room temperature. After 30 min, the flask was immersed in an

ice bath, keeping the solution cold at 0 °C and methane sulfonyl chloride (1.2 equiv) was added dropwisely. Then, the ice bath was removed, and the reaction mixture was allowed to stir at room temperature until the alcohol was consumed (according to TLC and GC). The reaction mixture was quenched by the addition of saturated aqueous ammonium chloride solution and extracted with dichloromethane (3x5 mL). The organic extracts were combined, dried over anhydrous sodium sulfate and concentrated under reduced pressure. The crude product was purified by silica gel column chromatography using 10% ethyl acetate in hexane as eluent to afford (±)-*erythro*-mesylated-**3** as a colorless oil (210 mg) in 95% yield.

^{1}H NMR (400 MHz, CDCl3): δ 7.27-7.23 (m, 2H), 7.18-7.14 (m, 3H), 4.66 (q, ^3J = 5.6-6.4 Hz, 1H), 3.04 (quint, ^3J = 6.8 Hz, 1H), 2.58 (s, 3H), 1.70-1.63 (m, 1H), 1.58-1.50 (m, 1H), 1.29 (d, ^3J = 6.8 Hz, 3H), 0.89 (t, ^3J = 7.6 Hz, 3H) ppm; ^{13}C NMR (100 MHz, CDCl3): δ 142.5, 128.6 (2C_{ortho}), 127.9 (2C_{meta}), 127.0 (C_{para}), 89.4 (COMs), 42.2 (C_{benzyl}), 38.0 (CH_3SO_3), 25.6 (CH_2), 15.8 (CH_3), 9.3 (CH_3) ppm.

2.6.2 Synthesis of (*E*)-2-phenyl-2-pentene.

In a 25 mL round bottom flask (±)-*erythro*-mesyl-**3** (100 mg, 0.4 mmol) was dissolved in a mixture of 25% dichloromethane in methanol (10 mL) and KOH (1.2 equiv) was added and then stirred under reflux till the starting material was fully consumed (~3 h, indicated by TLC or GC). Then, the reaction mixture was extracted with ethyl acetate (3×5 mL) after adding saturated aqueous ammonium chloride solution. The organic extracts were combined, dried over anhydrous sodium sulfate and concentrated under reduced pressure. The crude product was purified by silica gel column chromatography using 5% ethyl acetate in hexane as eluent to afford (*E*)-2-phenyl-2-pentene as a colorless oil (50 mg) in 83% yield.

^{1}H NMR (400 MHz, CDCl3): δ 7.31 (d, J = 8.4 Hz, 2H_{meta}), 7.22 (t, J = 8.0 Hz, 2H_{otho}), 7.13 (t, J = 7.6 Hz, 1H_{para}), 5.75-5.64 (m, 1H), 2.14 (quint, J = 6.4 Hz, 2H), 1.96 (s, 3H), 0.98 (dt, J = 7.6 and 0.8 Hz, 3H) ppm; **^{13}C NMR** (100 MHz, CDCl3): δ 144.0 (C_{quat}), 134.1 (R_2C=), 130.3 (2C_{ortho}), 128.1 (2C_{meta}), 126.4 (H(R)C=), 125.6 (C_{para}), 22.1 (C_d), 15.6 (C_a), 14.1 (C_e) ppm.

2.7 Bromination of (*R*)-1,1-diphenyl-2-propanol.

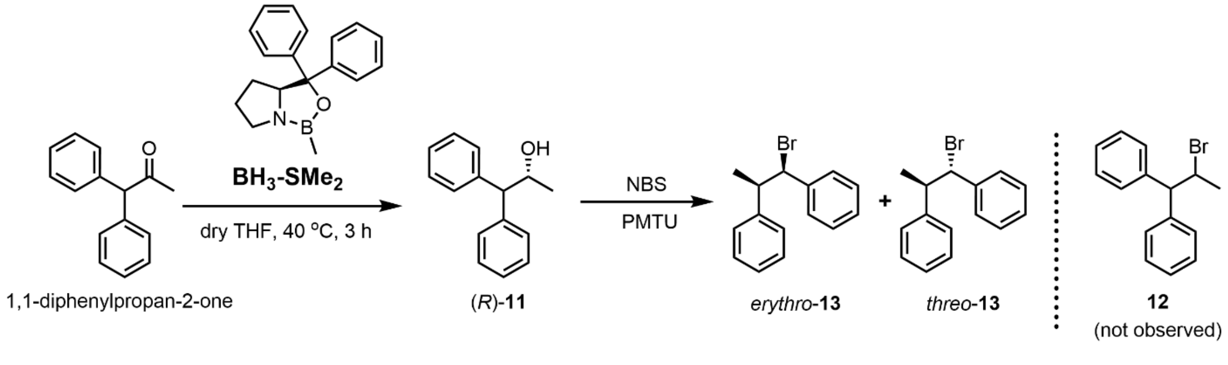

Scheme S7. Synthesis of (*R*)-**11** and its brominated products

2.7.1 Synthesis of (*R*)-1,1-diphenyl-2-propanol, (*R*)-11:

To an oven-dried round-bottom flask containing a stirred solution of anhydrous THF (10 mL) was added (*S*)-B-Me oxazaborolidine (0.263 g, 0.95 mmol) under nitrogen atmosphere followed by addition of borohydride-dimethyl sulfide complex (0.795 g, 10.5 mmol) dissolved in

anhydrous THF (5.5 mL). The reaction mixture was stirred for 5 min. and then 1,1-diphenyl-2-propanone (2 g, 9.5 mmol) dissolved in anhydrous THF (7 mL) was added dropwise over 5 min, and the reaction mixture was stirred for an additional 3 hours at 40°C. The reaction mixture was then quenched with a solution of 2M HCl and extracted with ether. The organic extracts were combined, dried over anhydrous sodium sulfate and concentrated. The crude product was purified by silica gel column chromatography using 20% ethyl acetate in hexane as eluent to afford 1.88 g of compound **11** as colorless oil in 93% yield. The ee was determined to be 70% by HPLC analysis using Daicel Chiralpak AS-H column using 1% isopropanol in hexane as eluent mixture at a flow rate of 1 mL min^{-1}.

Colorless liquid. **R_f** = 0.50 (silica gel, 20% EtOAc in hexanes, visualized with *p*-anisaldehyde stain); **^{1}H NMR** (400 MHz, CDCl3): δ 7.40-7.19 (m, 10H), 4.55 (m, 1H), 3.81 (d, ^3J = 8.8 Hz, 1H), 1.54 (s, 1H), 1.20 (d, ^3J = 6.0 Hz, 3H) ppm; ^{13}C NMR (100 MHz, $CDCl_3$): δ 142.6+141.6 ($2C_{quat}$), 129.0-126.7 ($10C_{phenyl}$), 70.2 (CH-OH); 60.8 (Ph_2CH) 21.6 (CH_3).

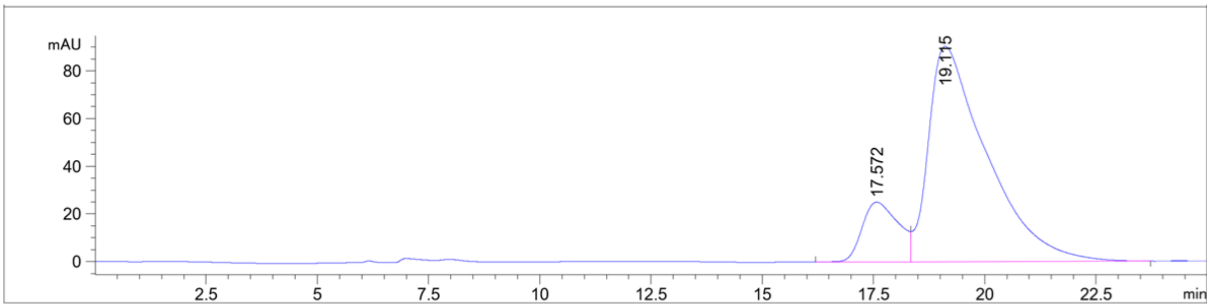

Peak (#)	Retention time (min)	Integration notation	Width (mAU*s)	Area (mAU)	Hight	Area (%)
**1**	17.572	MF	0.9805	218.08350	3.70707	15.24
**2**	19.115	FM	1.4756	1213.10156	13.70213	84.76

Figure S1. HPLC chromatography of (*R*)-1,1-diphenyl-2-propanol and *ee* determination. M means manual, and F means forced. Hence, MF means manual-to-forced integration and vice versa for FM.

2.7.2 Bromination of (*R*)-1,1-diphenyl-2-propanol, (*R*)-**11:**

The starting material, (*R*)-**11** (70% ee, 100 mg, 0.47 mmol), was dissolved in dichloroethane (4 mL) in an oven-dried 25 mL round bottom flask, and PMTU (18.7 mg, 0.14 mmol) was added, and the mixture was stirred at *RT* until PMTU was completely dissolved. Then, NBS (192.8 mg, 1.08 mmol) was added in two portions to the reaction mixture. The first portion of NBS (96.4 mg) was added to the reaction mixture and allowed to stir for 30 min. at *RT*. After 30 min, the second portion of NBS (96.4 mg) was introduced to the reaction mixture and stirred at *RT* for 5 h. The reaction mixture was quenched with saturated aqueous ammonium chloride solution and extracted with DCM (3×5 mL). The organic extracts were combined, dried over anhydrous sodium sulfate and concentrated. The crude product was purified by preparative TLC using 1% ethyl acetate in hexane as eluent to afford the *erythro*-**13** in 85% purity as a colorless oil (26 mg) in 20% overall yield (see also Figure S63).

erythro-1-bromo-1,2-diphenyl propane, *erythro*-**13**:

^{1}H NMR (400 MHz, CDCl3): δ 7.22-7.00 (m, 10H), 5.08 (d, ^3J = 9.4 Hz, 1H) 3.44 (m, 1H), 1.61 (d, ^3J = 6.9 Hz, 3H) ppm; ^{13}C NMR (100 MHz, $CDCl_3$): δ 142.8+141.6 ($2C_{quat}$), 128.4-127.8 ($4C_{meta}$+$4C_{ortho}$), 126.7 ($2C_{para}$), 62.7 (CHBr); 48.5 ($PhCH_2$) 21.5 (CH_3).

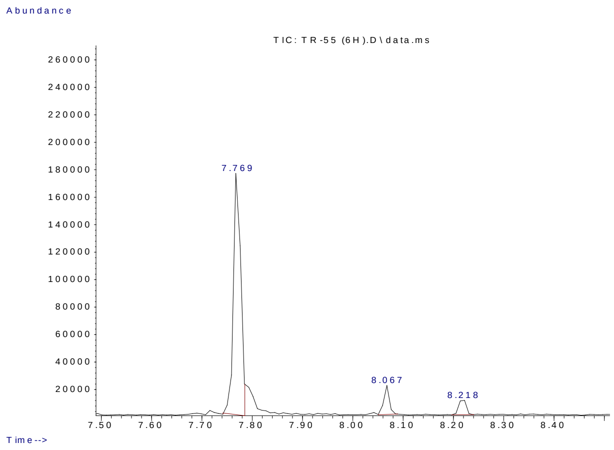

Entry	Time (min)	Identification	% of total	*dr*
1	7.759	(*R*)-**11** (*ee*-70%)	87.1	
2	8.058	*erythro*-**13**	7.6	59%
3	8.207	*threo*-**13**	5.3	41%

Figure S2. GC-MS chromatograph showing the distribution of all main components in the bromination reaction of (*R*)-11.

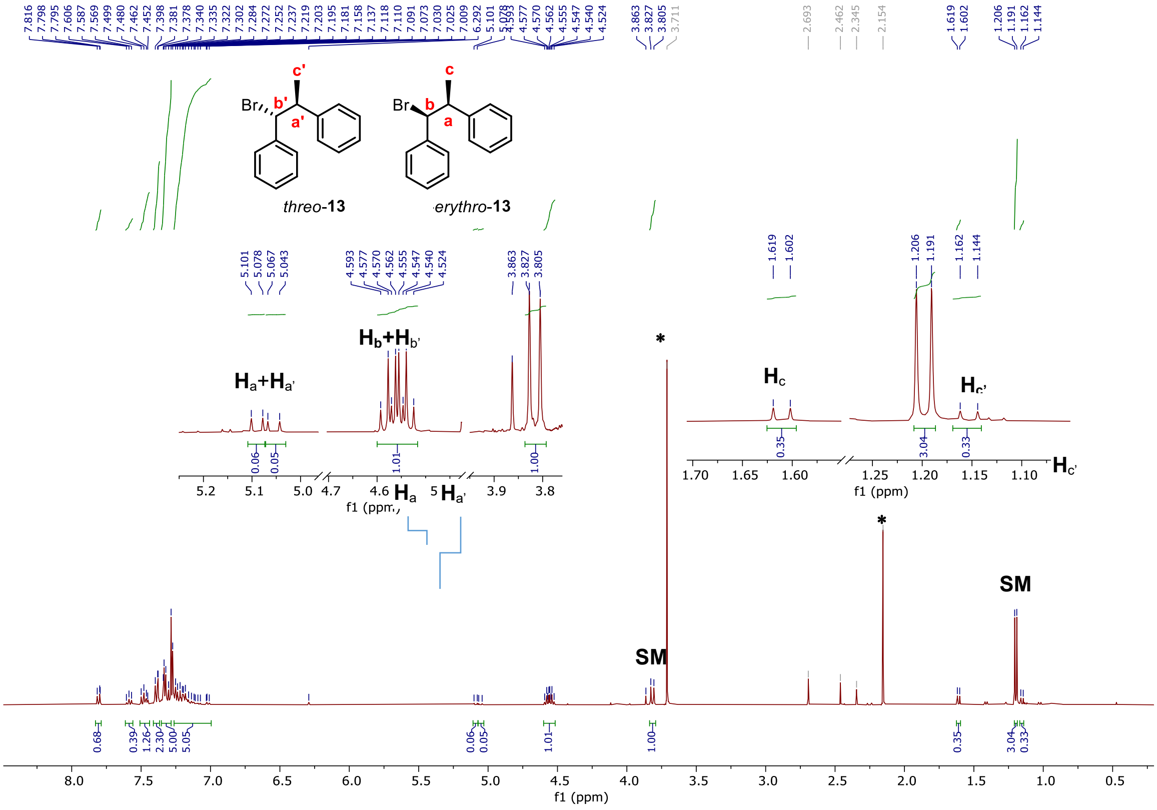

Figure S3. ^{1}H NMR (400 MHz, $CDCl_3$) spectrum of crude reaction following bromination of (*R*)-**11**. The doublet signals at δ = 1.1-1.7 ppm assigned for methyl groups in **11** (**SM**: δ = 1.19 ppm) and *erythro*-**13** (**P$_1$**: δ = 1.61 ppm) and *threo*-**13** (**P$_2$**: δ = 1.13 ppm). were used for

determining the reaction yield (≈18%). The diastereomeric ratio (*dr* 60:40) was determined by comparing the integration of the two sets of doublet signals at δ = 5.0-5.1 ppm . The asterisks denote trace solvents.

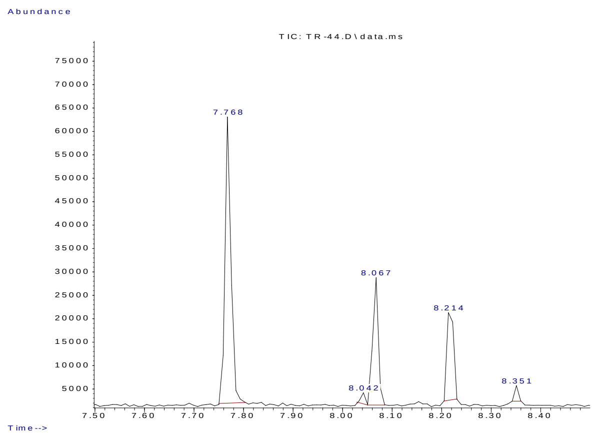

Entry	Time (min)	Identification	% of total	*dr*
1	7.759	(*R*)-**11** (*ee*-70%)	59.360	
2	8.042	Unknown side product	1.500	
3	8.067	*erythro*-**13**	21.851	59%
4	8.214	*threo*-**13**	15.352	41%
5	8.351	Unknown side product	1.938	

Figure S4. GC-MS chromatograph showing the separation of all products in the bromination reaction of (*R*)-11 at 40 °C over 20 h.

2.8 Bromination of (±)-11.

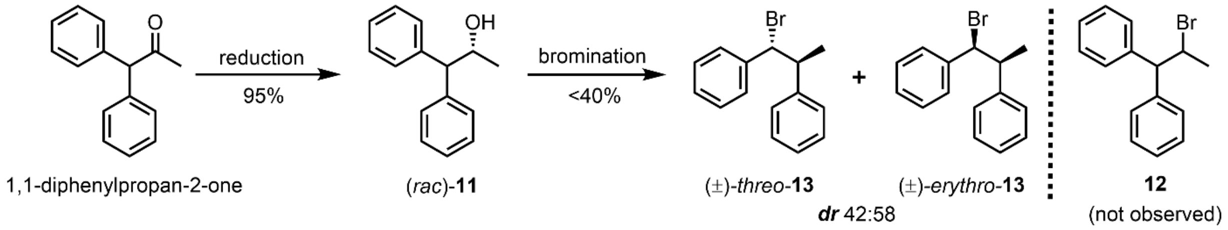

Scheme S8. Synthesis of (±)-**11** and its brominated products.

2.8.1 Synthesis of (±)-1,1-diphenyl-2-propanol, (±)-11:

To an oven-dried round-bottom flask containing a stirred solution of dry THF (4 mL) was added lithium aluminium hydride (43.4 mg, 1.14 mmol) and the reaction was cooled to 0 °C. Then, 1,1-diphenyl-2-propanone (200 mg, 0.95 mmol) was added, and the reaction mixture was stirred for 2 h at 0 °C. The reaction mixture was then quenched with a solution of 2M HCl and extracted with ether. The organic extracts were combined, dried over anhydrous sodium sulfate and concentrated. The crude product was purified by silica gel column chromatography using 25% ethyl acetate in hexane as eluent to afford 190 mg of compound **11** as colorless oil in 93% yield. The *ee* value was determined to be 70% by HPLC analysis using Daicel Chiralpak AS-H column using 1% isopropanol in hexane as an eluent mixture at a flow rate of 1 mL min^{-1}.

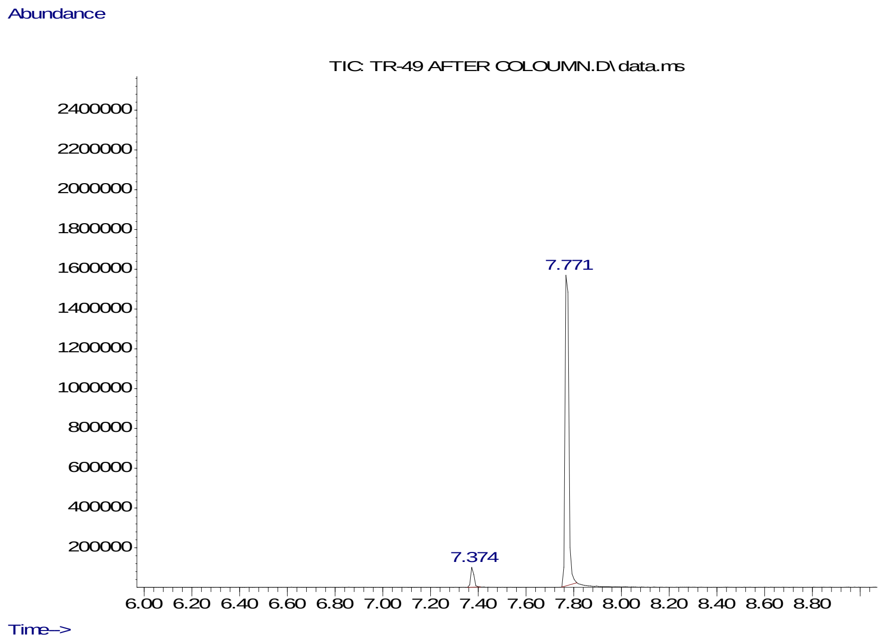

Entry	Time (min)	Identification	% of total
1	7.374	Unidentified impurity	5.1
2	7.771	(±)-**11**	94.9

Figure S5. GC-MS chromatograph presenting the purity of (±)-**11**.

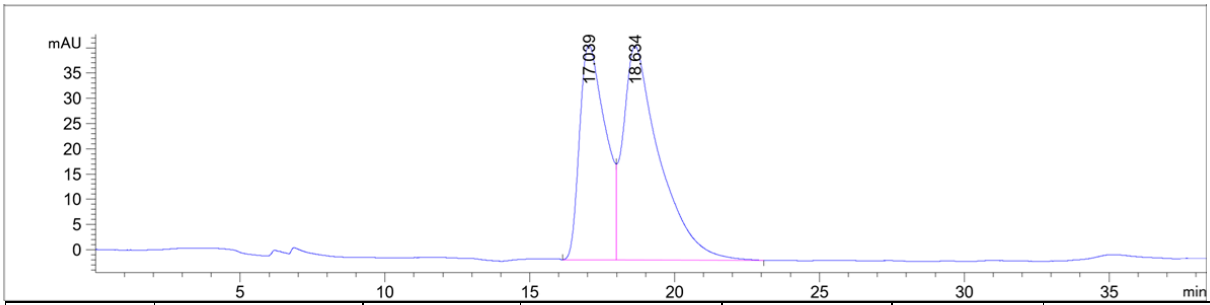

Peak (#)	Retention time (min)	Integration notation	Width (mAU*s)	Area (mAU)	Hight	Area (%)
**1**	17.038	BB	0.6870	241.05038	4.99554	48.8
**2**	18.643	BB	0.8823	253.03265	4.00933	51.2

Figure S6. HPLC chromatography of (±)-**11** and *ee* determination. BB means baseline-to-baseline integration.

2.8.2 Bromination of (±)-**11**:

The starting material, (±)-**11** (50 mg, 0.24 mmol), was dissolved in dichloroethane (4 mL) in an oven-dried 25 mL round bottom flask, and PMTU (9.3 mg, 0.07 mmol) was added, and the mixture was stirred at *RT* until PMTU was completely dissolved. Then, NBS (96.4 mg, 0.54 mmol) was added in two portions to the reaction mixture. The first portion of NBS (48.2 mg) was added to the reaction mixture and allowed to stir for 30 min. at *RT*. After 30 min, the second portion of NBS (48.2 mg) was introduced to the reaction mixture and stirred at *RT* for 5 h. The reaction mixture was quenched with saturated aqueous ammonium chloride solution and extracted with DCM (3×5 mL). The organic extracts were combined, dried over anhydrous sodium sulfate and concentrated. The crude reaction was run on a preparative TLC plate using

1% ethyl acetate in hexane as eluent to afford a mixture of diastereomers in *dr* 58:42 in favor of (±)-*erythro*-**13**.

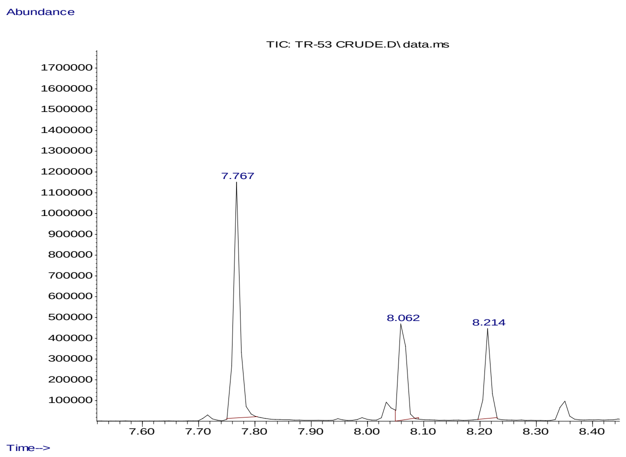

Entry	Time (min)	Identification	% of total	*dr*
1	7.767	(±)-**11**	53.9	
2	8.062	(±)-*erythro*-**13**	26.6	58%
3	8.214	(±)-*threo*-**13**	19.5	42%

Figure S7. GC-MS chromatograph presenting the diastereomeric ratio (58:42) between (±)-*erythro*- and (±)-*threo*-**13**.

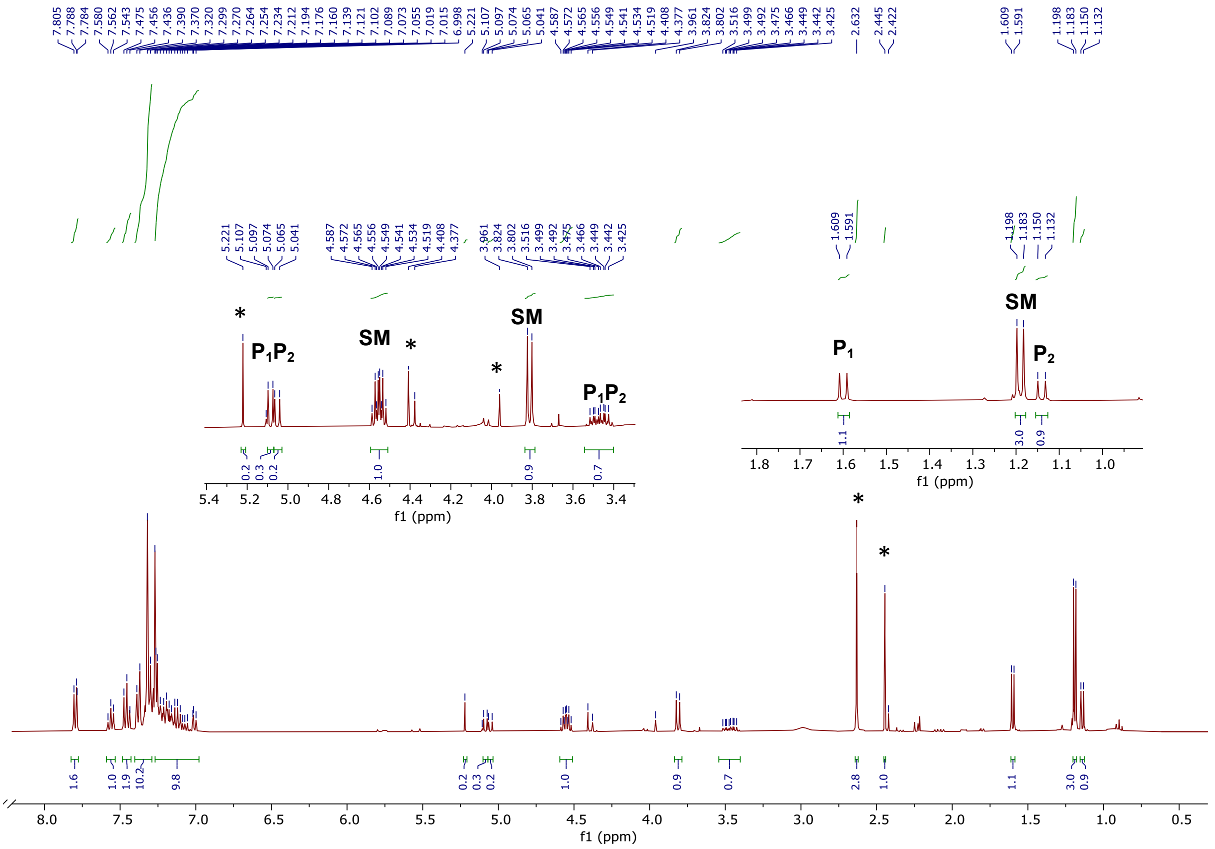

Figure S8. ^{1}H NMR (400 MHz, $CDCl_3$) spectrum of crude reaction following bromination of (±)-**11**. The doublet signals at δ = 1.1-1.7 ppm assigned for methyl groups in **11** (**SM**: δ = 1.19 ppm) and **13** were used to determine yields and the *dr* between (±)-*erythro*-**13** (**P1**: δ = 1.61 ppm) and (±)-*threo*-**13** (**P2**: δ = 1.13 ppm). The asterisks denote unidentified side-products.

2.9 Bromination of (±)-*threo*-1,2-diphenyl-2-butanol, (±)-*threo*-**14**.

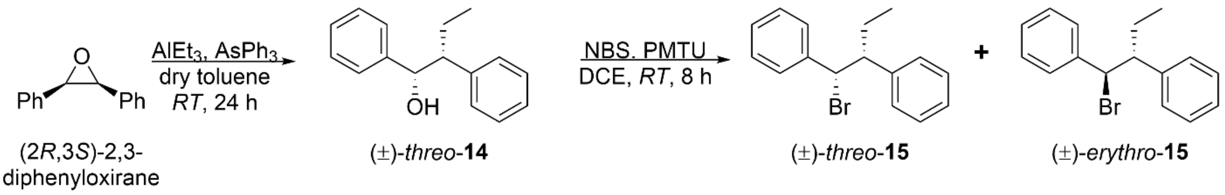

Scheme S9. Synthesis of (±)-*threo*-**14** and its brominated products.

2.9.1 Synthesis of (±)-*threo*-14:

To an oven-dried round-bottom flask containing a stirred solution of 2,3-diphenyloxirane (100 mg, 0.51 mmol) in dry toluene (6 mL) was added $AsPh_3$ (10 mol-%) as a Lewis base and subsequently, 1.02 mmol of triethylaluminum (2 equiv) was added as a solution in either hexane or toluene. The mixture was stirred for 24 hours at room temperature under a nitrogen atmosphere. Then, the crude was quenched with 5 mL of a 1 M HCl solution. The phases were separated and the aqueous layer was extracted twice with ether. The combined organic extracts were dried over $MgSO_4$, and the solvents evaporated in vacuo. The crude products were purified by flash chromatography over silica gel with ethyl acetate/hexane mixture. The product was isolated as a colorless liquid single diastereomer (93 mg, 90% *de*) in 81% yield.

^{1}H NMR (400 MHz, $CDCl_3$): δ 7.26-7.05 (m, 8H), 6.97 (m, 2H), 4.71 (dd, H_d, ^3J = 6.4 and 3.6 Hz, 1H), 2.76 (dt, H_c, ^3J = 10.8 and 4 Hz, 1H), 1.92 (m, H_b, 1H), 1.88 (bs, OH, 1H), 1.67 (m, $H_{b'}$, 1H), 0.67 (t, H_a, ^3J = 7.2 Hz, 3H) ppm. **^{13}C NMR** (100 MHz, $CDCl_3$): δ 142.6 and 140.9 (2×C_{quat}), 128.4 and 127.6 (4×C_{meta}), 127.4 (2×C_{ortho}), 126.7 (C_{para}), 126.0 (2×C'_{ortho}), 125.9 (C'_{para}), 77.9 (C_d), 55.1 (C_c), 22.2 (C_b), 11.7 (C_a) ppm.

2.9.2 Synthesis of (±)-*threo*- and (±)-ery*thro*-1,2-diphenyl-1-bromobutane, (±)-*threo*-**15** and (±)-ery*thro*-**15**.

The starting material, (±)-*threo*-**14** (80 mg, 0.35 mmol), was dissolved in dichloroethane (4 mL) in an oven-dried 25 mL round bottom flask, and PMTU (13.9 mg, 0.10 mmol) was added, and the mixture was stirred at *RT* until PMTU was completely dissolved. Then, NBS (144.7 mg, 0.81 mmol) was added in two portions to the reaction mixture. The first portion of NBS (72.3 mg) was added to the reaction mixture and allowed to stir for 30 min. at *RT*. After 30 min, the second portion of NBS (72.4 mg) was introduced to the reaction mixture and stirred at *RT* for 8 h. The reaction mixture was quenched with saturated aqueous ammonium chloride solution and extracted with DCM (3×5 mL). The organic extracts were combined, dried over anhydrous sodium sulfate and concentrated. The crude product was purified by preparative TLC using 1% ethyl acetate in hexane as eluent to afford a mixture of diastereomers (dr 3:1).

Major diastereomer (~70%):

^{1}H NMR (400 MHz, $CDCl_3$): δ 7.27-7.03 (m, 8H), 6.99-6.92 (m, 2H), 4.71 (dd, H_d, ^3J = 6.4 and 3.6 Hz, 1H), 2.76 (dt, H_c, ^3J = 10.8 and 4 Hz, 1H), 1.92 (m, H_b, 1H), 1.88 (bs, OH, 1H), 1.67 (m, $H_{b'}$, 1H), 0.67 (t, H_a, ^3J = 7.2 Hz, 3H) ppm.

Minor diastereomer (~30%):

^{1}H NMR (400 MHz, CDCl$_3$): δ 7.27-7.03 (m, 8H), 6.99-6.92 (m, 2H), 4.71 (dd, H$_d$, ^3J = 6.4 and 3.6 Hz, 1H), 2.76 (dt, H$_c$, ^3J = 10.8 and 4 Hz, 1H), 1.92 (m, H$_b$, 1H), 1.88 (bs, OH, 1H), 1.67 (m, H$_{b'}$, 1H), 0.67 (t, H$_a$, ^3J = 7.2 Hz, 3H) ppm.

3. Stereoselective bromination of chiral 1-phenyl-1-ethanol

Table S1. Stereoselective bromination of chiral 1-phenyl-1-ethanol in dichloromethane and hexane.

Reaction 1:	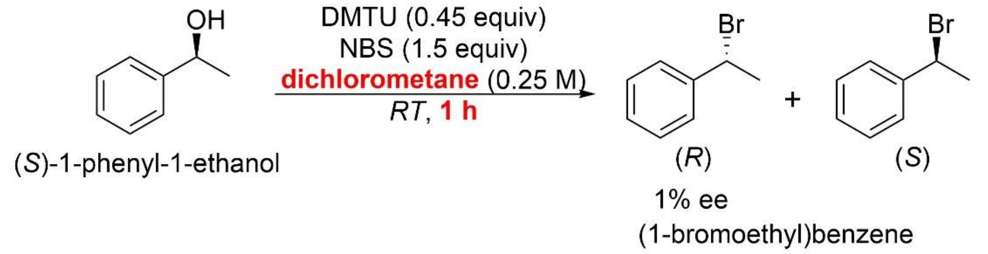
Chiral HPLC	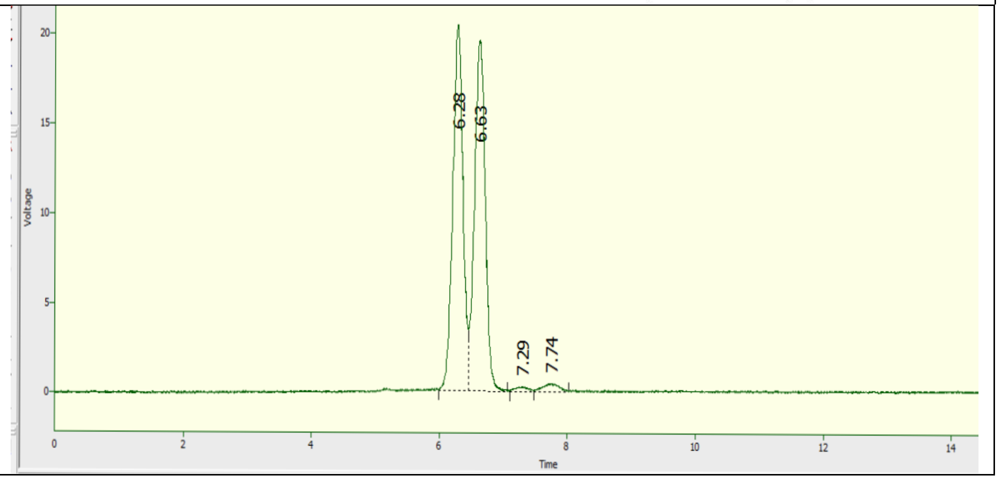

Reaction 2:	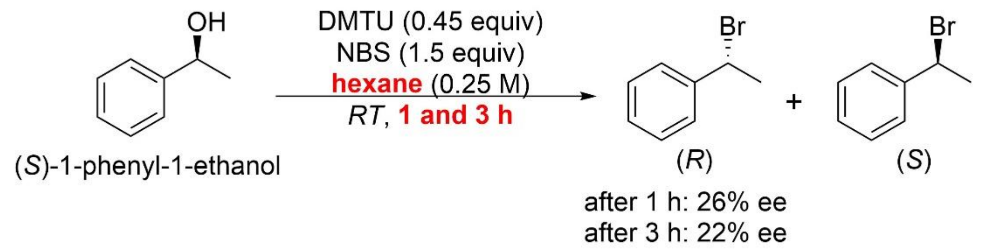
Chiral HPLC	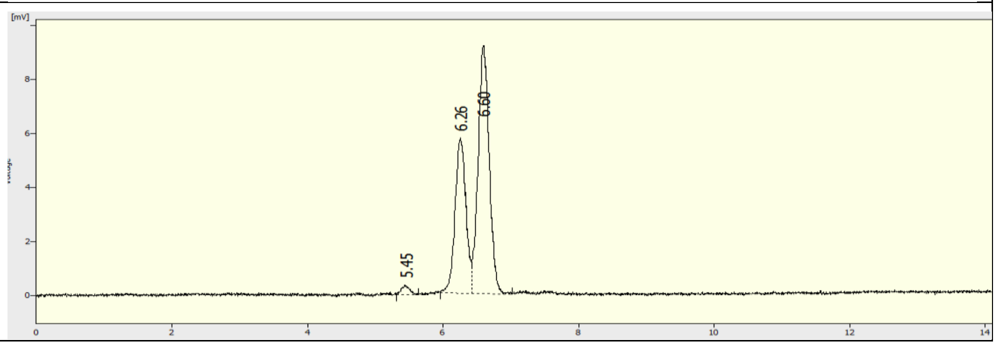

Reaction 3:	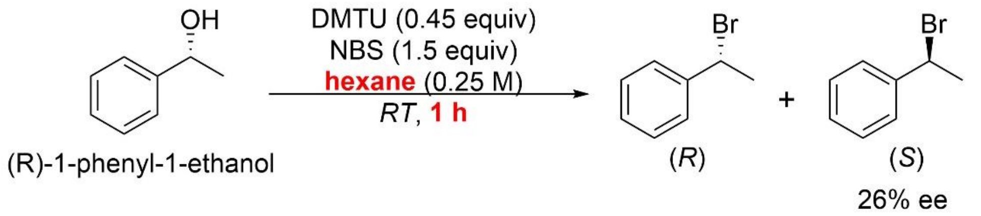
Chiral HPLC	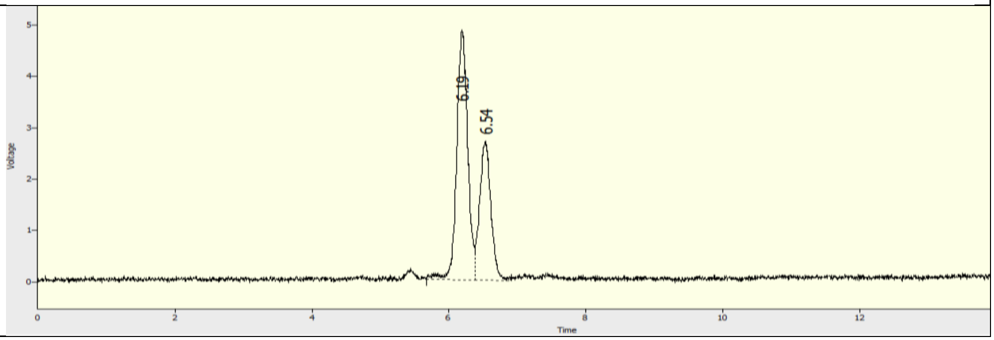

4. Proving the stereoselective bromination pathways of (±)-*erythro*-**2a.**

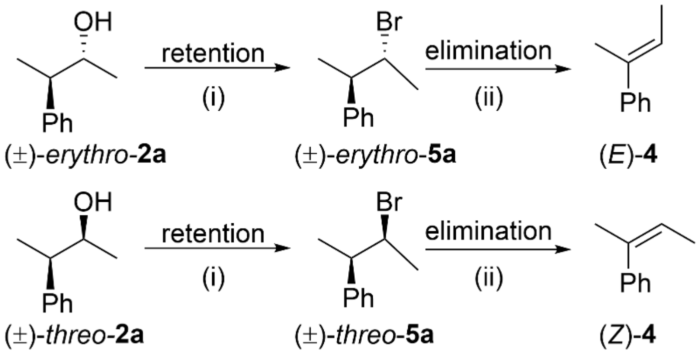

Scheme S10. Stereoselective synthesis of alkene **4** via bromination (i) and elimination (ii) reactions of 3-phenyl-2-butanol diastereomers. Reaction conditions: (i) NBS (2.3 equiv), PMTU (0.3 equiv), dichloroethane, *RT*, 6 h; (ii) KOH (1.2 equiv), 25% dichloromethane in methanol, 90 ºC, 3 h.

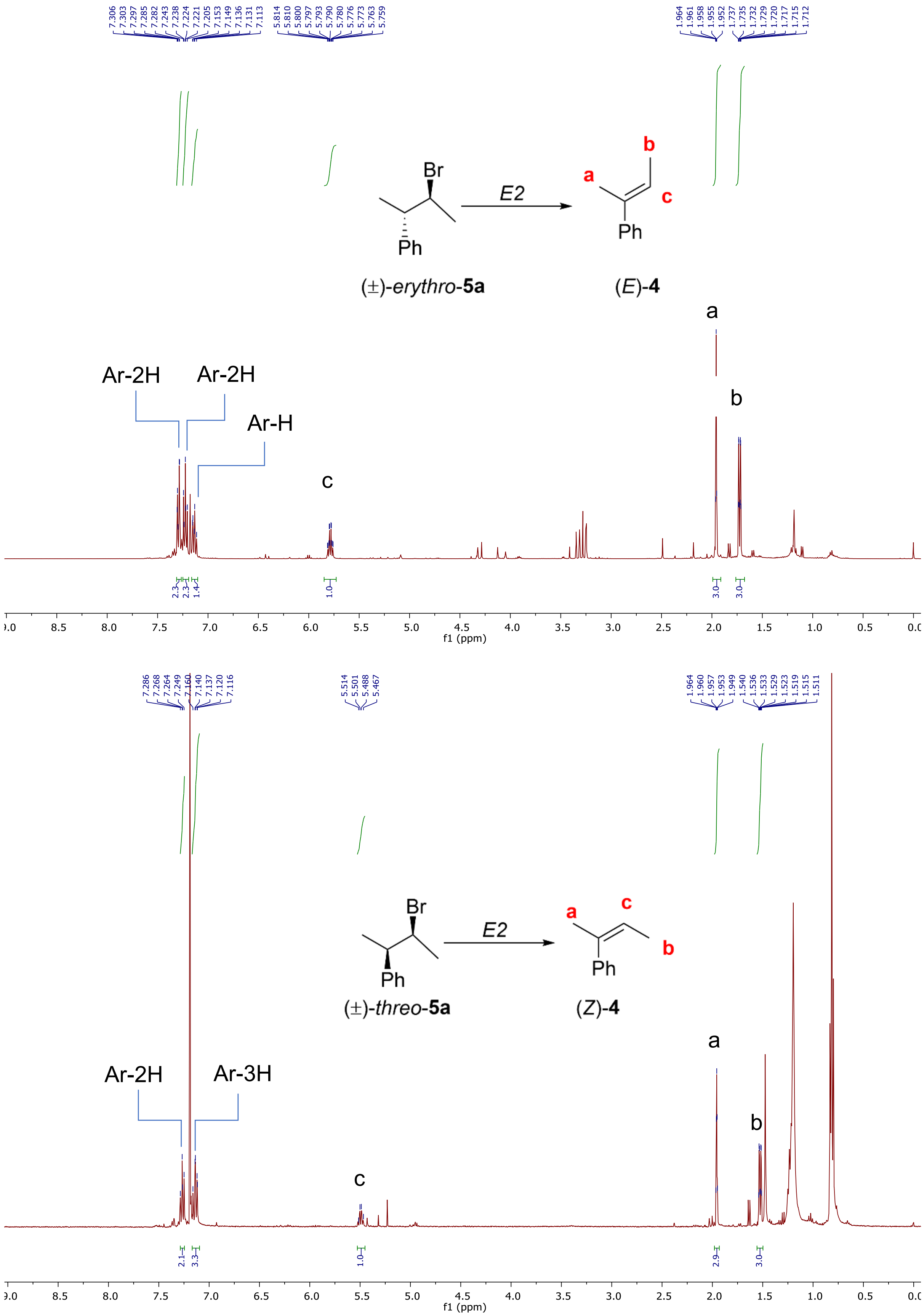

Figure S9. ^{1}H NMR (400 MHz) spectra of crude products resulted from elimination reactions of **5a**. *Top*: (±)-*erythro*-3-phenyl-2-butanol; *Bottom*: and (±)-*threo*-3-phenyl-2-butanol.

5. Optimization of reaction parameters in the bromination of (±)-*erythro*-**2a** with NBS and various disubstituted thioureas to afford (±)-*erythro*-**5a.**

5.1 General procedure.

To an oven-dried 10 mL round bottom flask filled with dichloroethane (DCE) (2 mL), nitrobenzene (10 mg, 0.081 mmol) under nitrogen atmosphere, (±)-*erythro*-**2a** (50 mg, 0.33 mmol) and various thioureas at different equivalents were added and the solution was stirred at *RT* till the thiourea was completely dissolved in DCE. After 15 min, *N*-bromosuccinimide (NBS) at different equivalents was added in two equal portions to the reaction mixture. The first portion was added and allowed to stir for 30 min. and then, a second portion was introduced to the reaction and stirred at *RT* for 6-24 h. Then, the reaction mixture was quenched with saturated aqueous ammonium chloride solution and extracted with DCE (3×3 mL). The organic extracts were combined, dried over anhydrous sodium sulfate and concentrated. The crude product was dissolved in $CDCl_3$ (0.5 mL), and ^{1}H NMR of the sample was recorded.

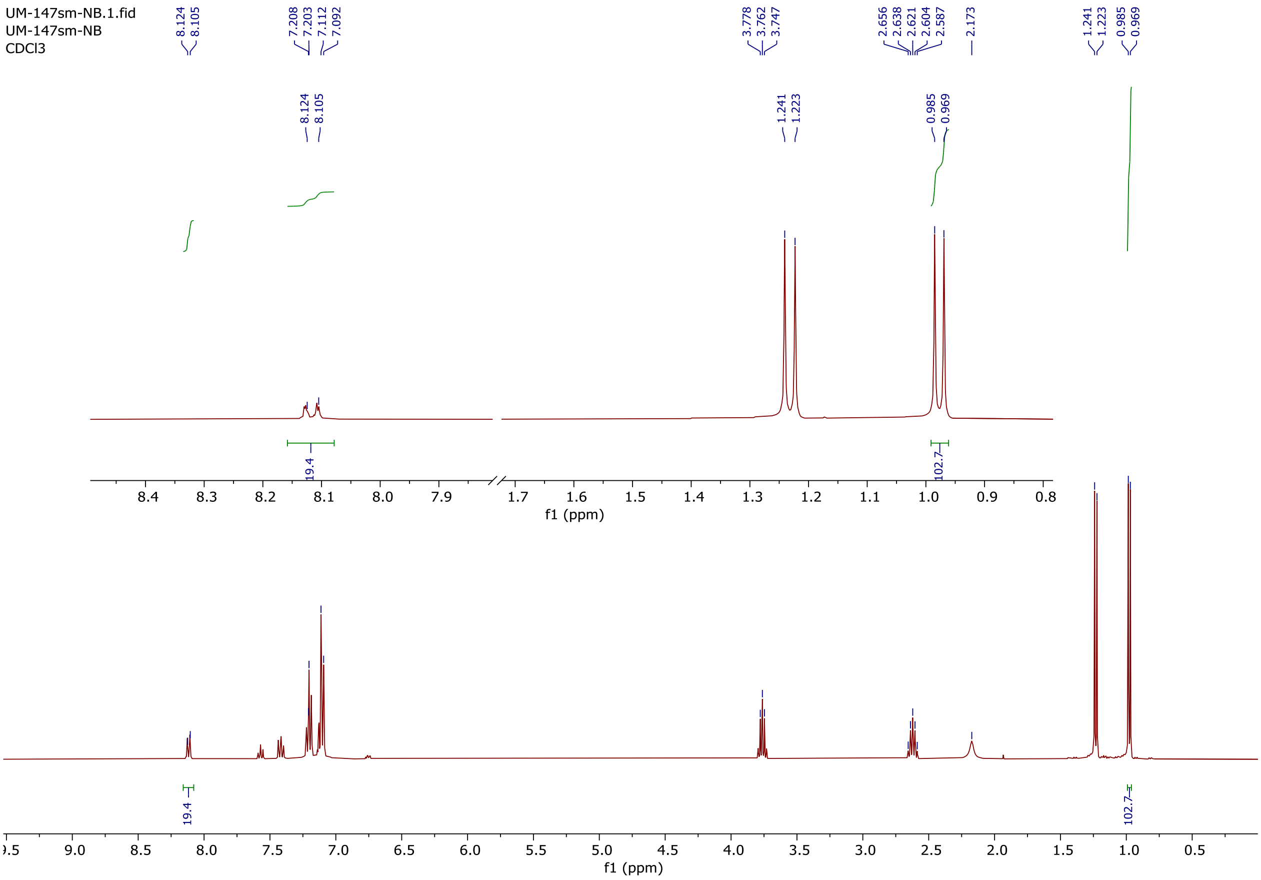

Figure S10. ^{1}H NMR (400 MHz) of (±)-*erythro*-**2a** with nitrobenzene.

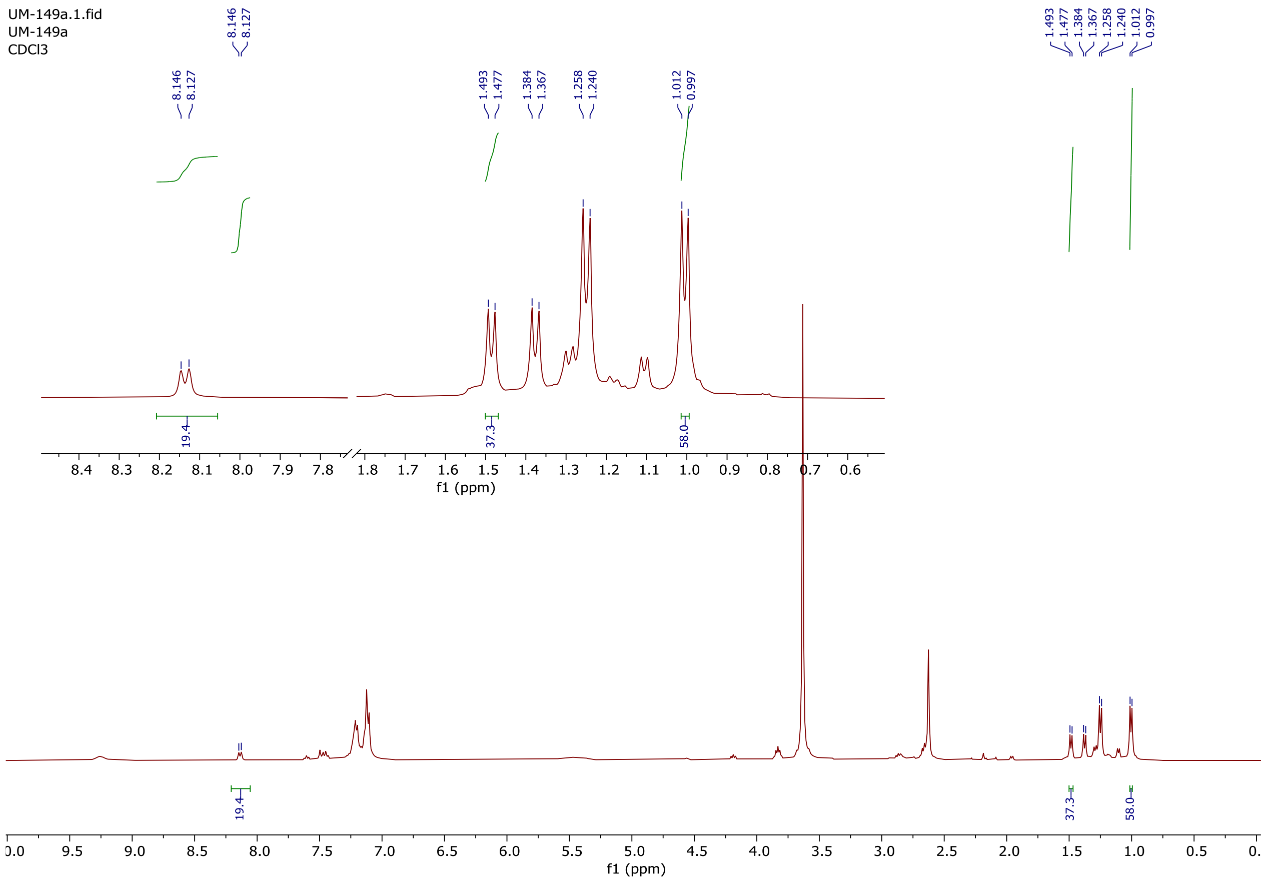

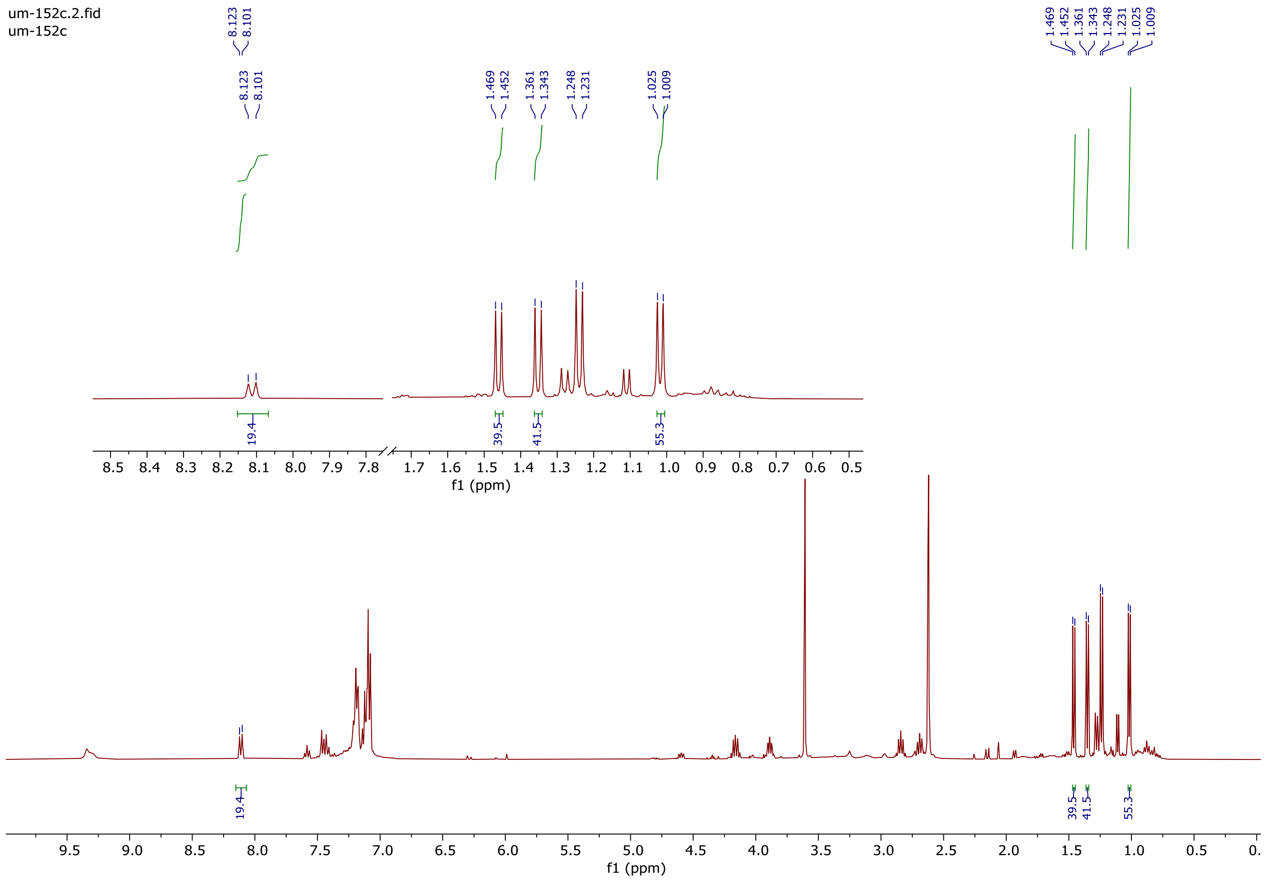

Figure S11. ^{1}H NMR (400 MHz) spectra of products following the bromination of (±)-*erythro*-**2a** under specific reaction conditions given in Table 1: *Top*: Entry 1.; *Bottom:* Entry 2.

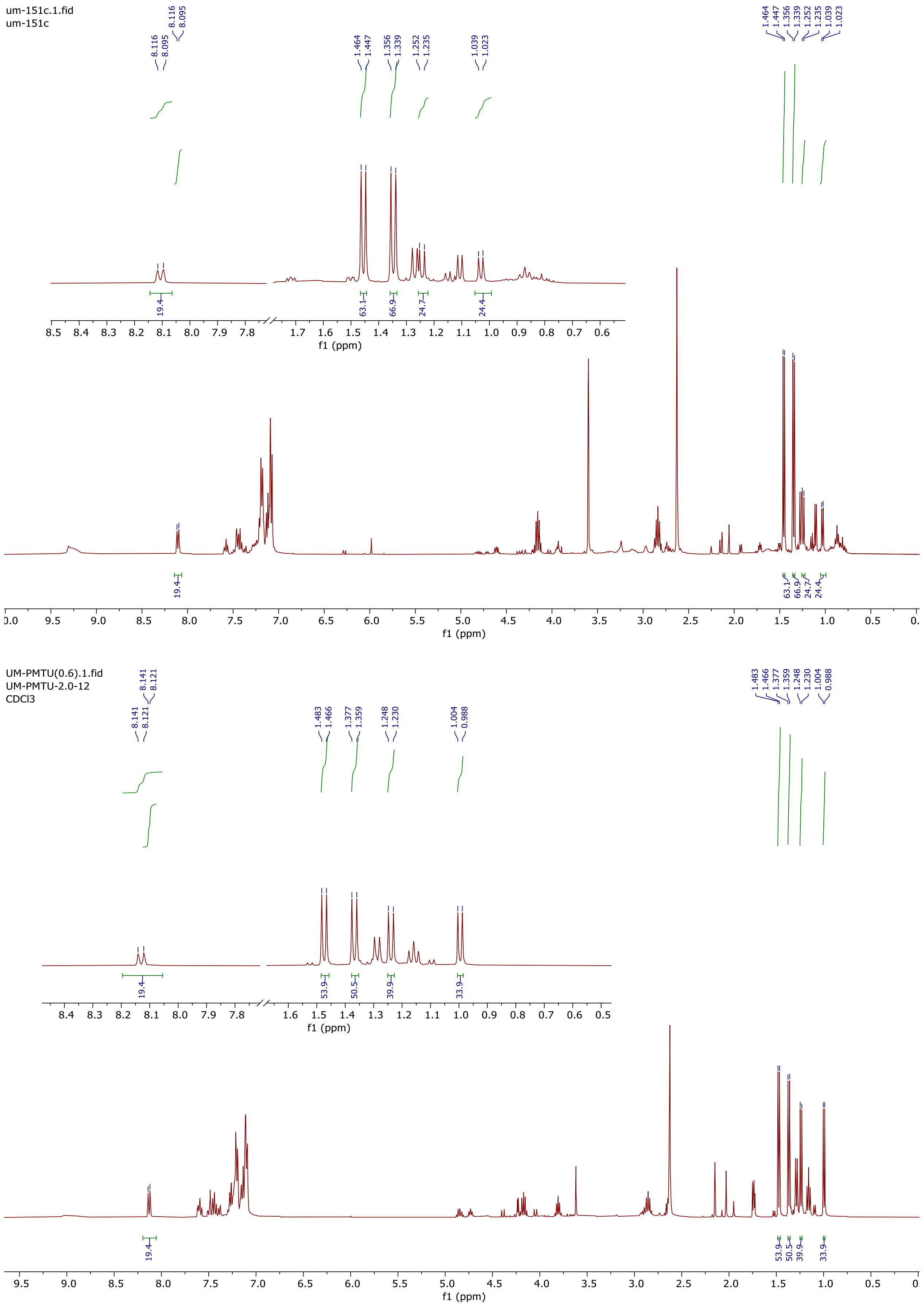

Figure S12. ^{1}H NMR (400 MHz) spectra of products following the bromination of (±)-*erythro*-**2a** under specific reaction conditions given in Table 1: *Top*: Entry 3.; *Bottom:* Entry 4.

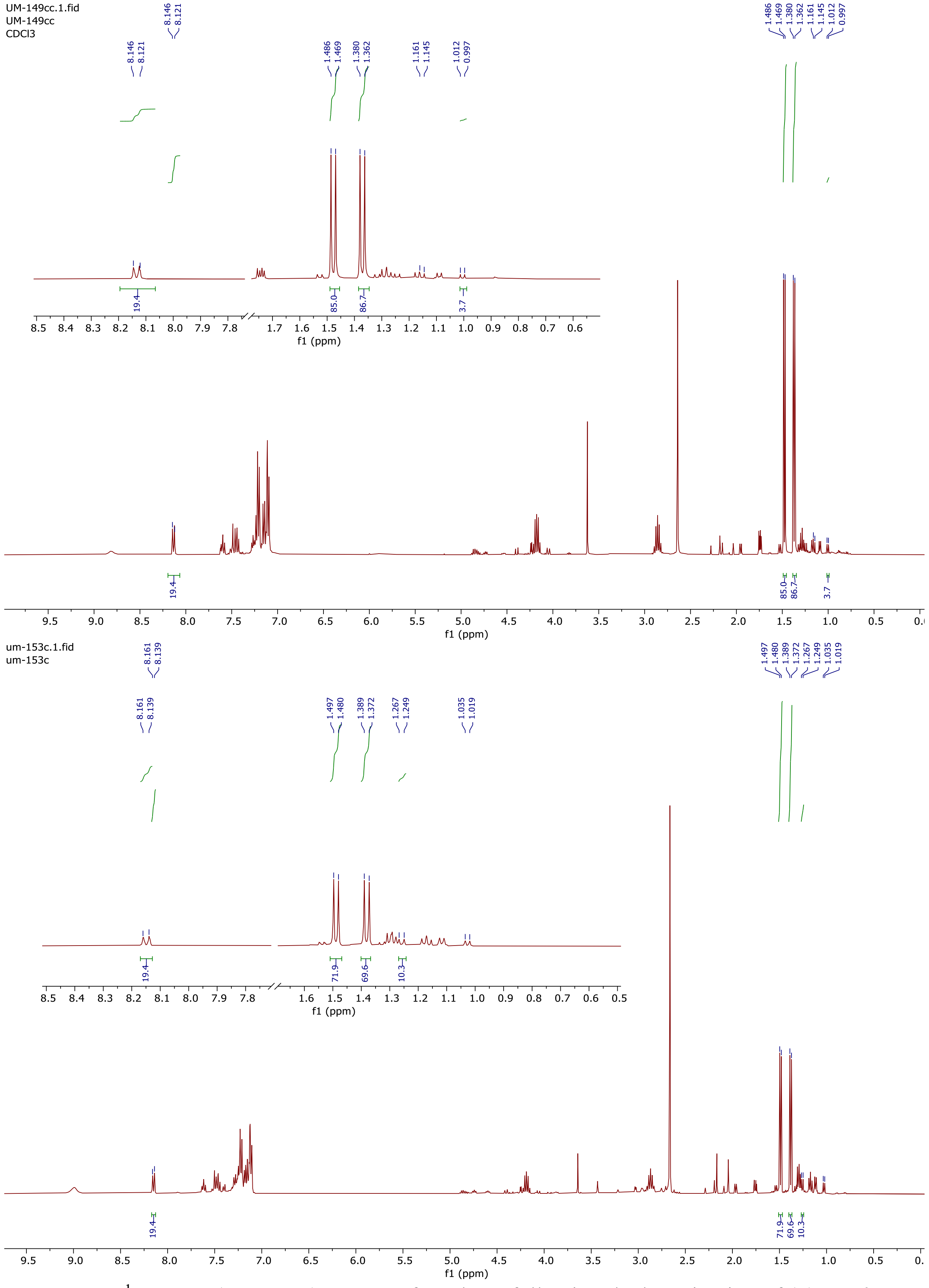

Figure S13. ^{1}H NMR (400 MHz) spectra of products following the bromination of (±)-*erythro*-**2a** under specific reaction conditions given in Table 1: *Top*: Entry 5.; *Bottom:* Entry 6.

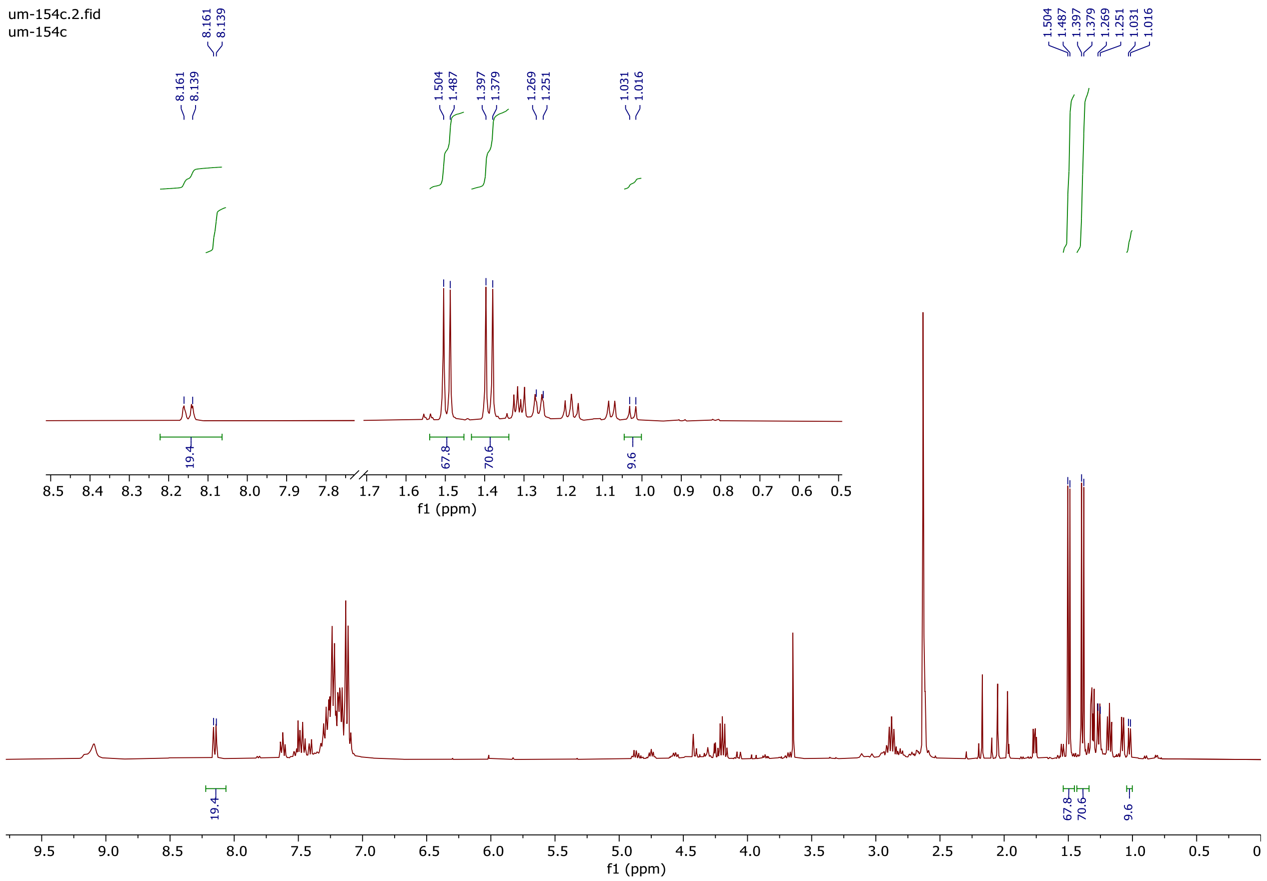

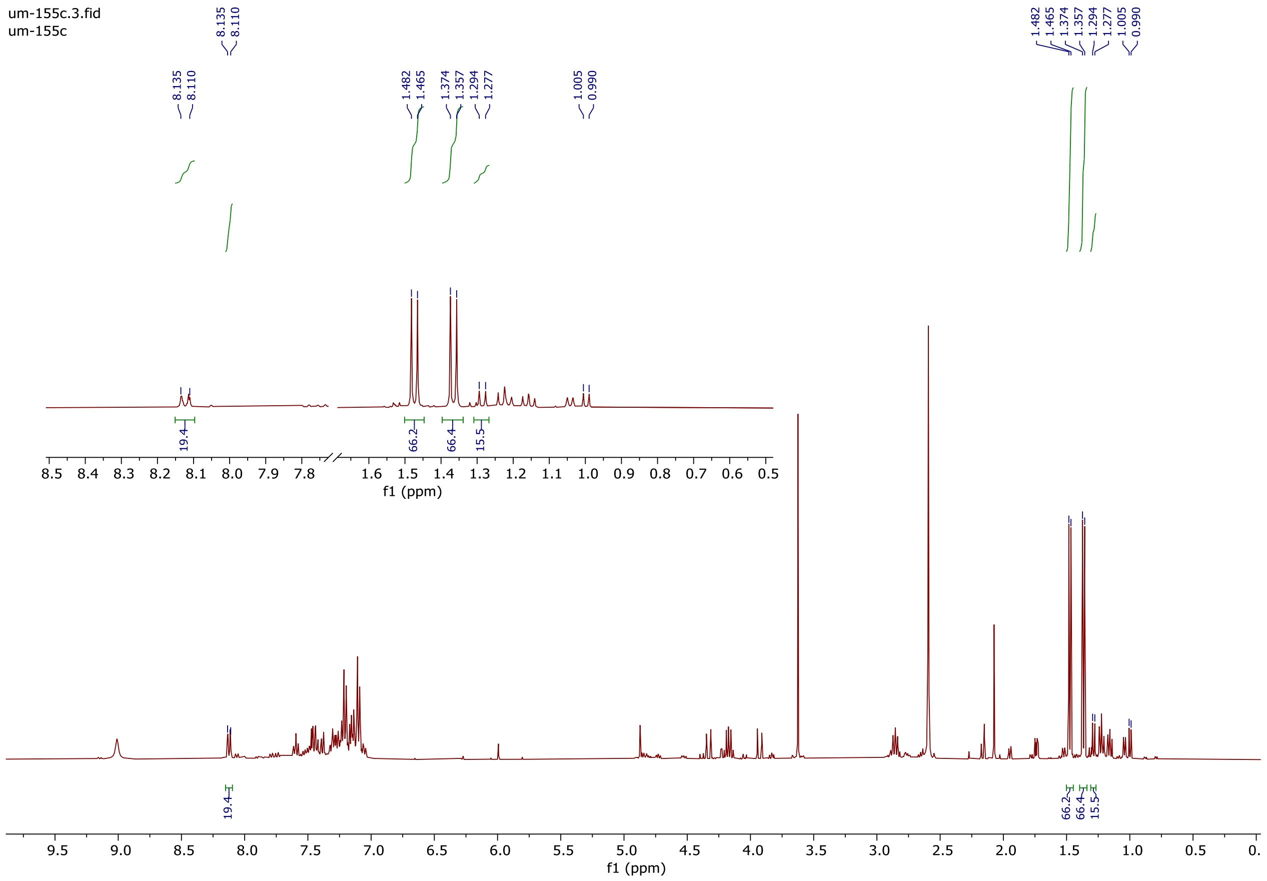

Figure S14. ^{1}H NMR (400 MHz) spectra of products following the bromination of (±)-*erythro*-**2a** under specific reaction conditions given in Table 1: *Top*: Entry 7; *Bottom:* Entry 8.

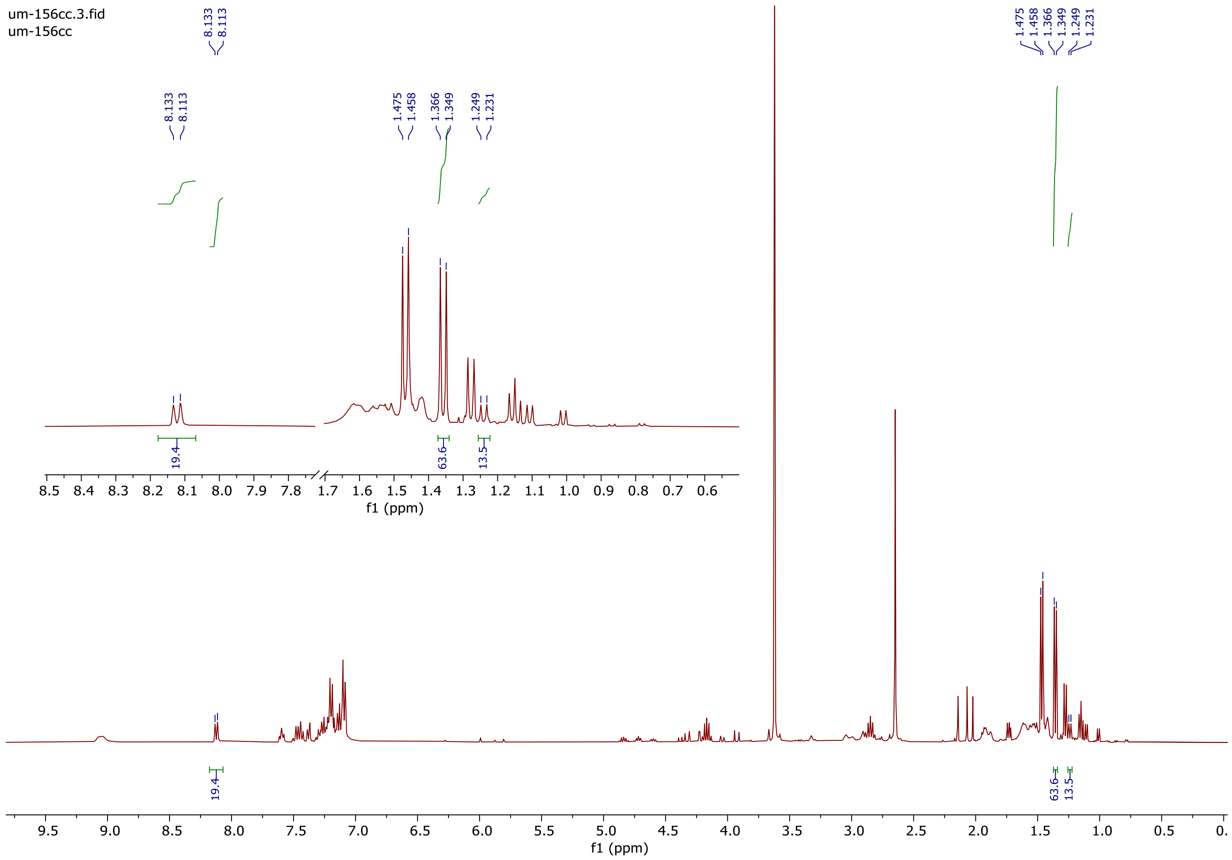

Figure S15. ^{1}H NMR (400 MHz) spectrum of products following the bromination of (±)-*erythro*-**2a** under specific reaction conditions given in Table 1, entry 9.

6. Transformation of (±)-erythro-3-phenyl-2-butanol to the corresponding bromoalkane stereoisomers under three different reaction conditions.

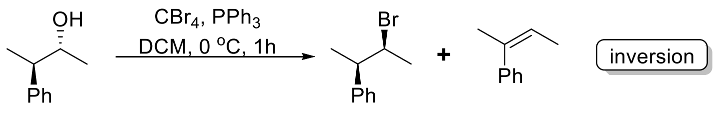

(±)-*erythro*-**2a** (±)-*threo*-**5a** (*Z*)-**4**

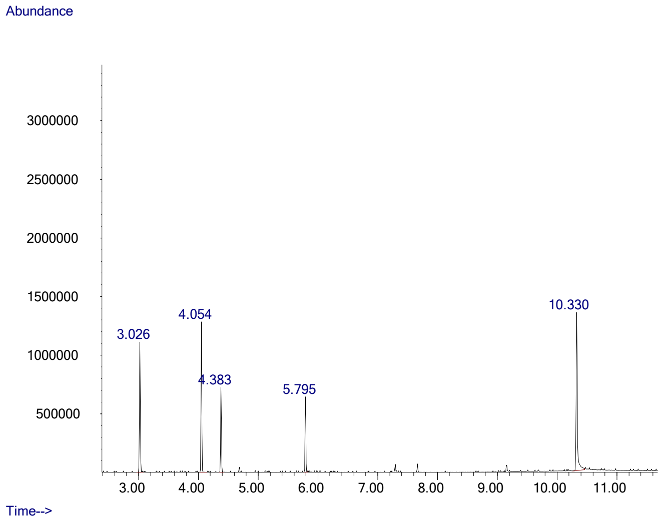

Entry	Time (min)	Identification
1	3.026	$CHBr_3$
2	4.054	(*Z*)-2-phenyl-2-butene, **4**
3	4.383	CBr_4
4	5.795	(±)-*threo*-**5a**
5	10.330	O=PPh_3

Figure S16. Appel-type bromination of (±)-*erythro*-3-phenyl-2-butanol and GC analysis of the crude reaction.

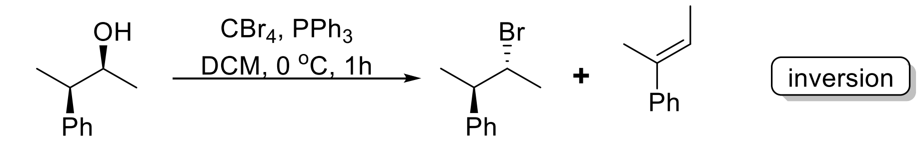

(±)-*threo*-**2a** (±)-*erythro*-**5a** (*E*)-**4**

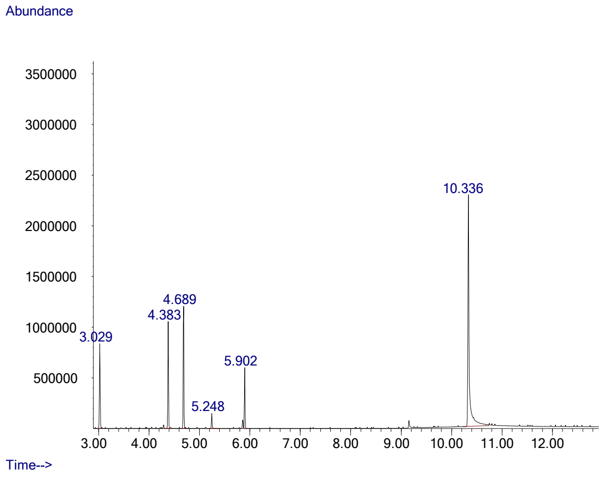

Entry	Time (min)	Identification
1	3.029	$CHBr_3$
2	4.383	CBr_4
3	4.689	(*E*)-2-phenyl-2-butene, **4**
4	5.248	(±)-*threo*-**2a**
5	5.902	(±)-*erythro*-**5a**
6	10.336	O=PPh_3

Figure S17. Appel-type bromination of (±)-*threo*-3-phenyl-2-butanol and GC analysis of the crude reaction.

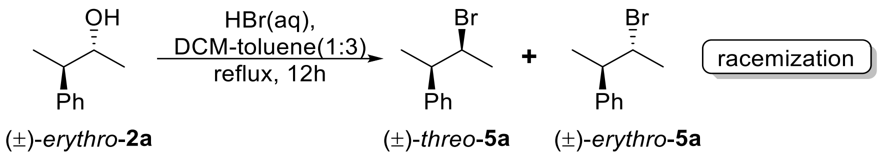

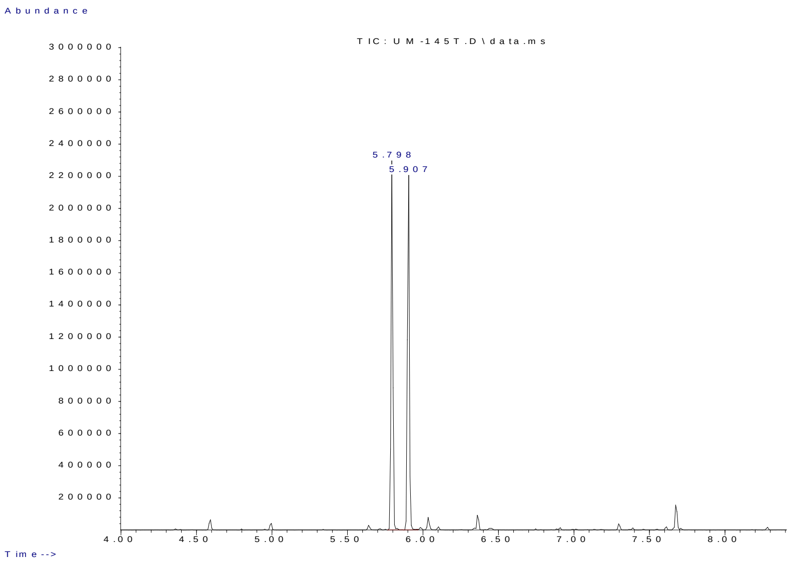

Entry	Time (min)	Identification
1	5.798	(±)-*threo*-**5a**
2	5.902	(±)-*erythro*-**5a**

Figure S18. Bromination of (±)-*erythro*-3-phenyl-2-butanol with aqueous HBr and GC analysis of the crude reaction.

7. NMR Spectra

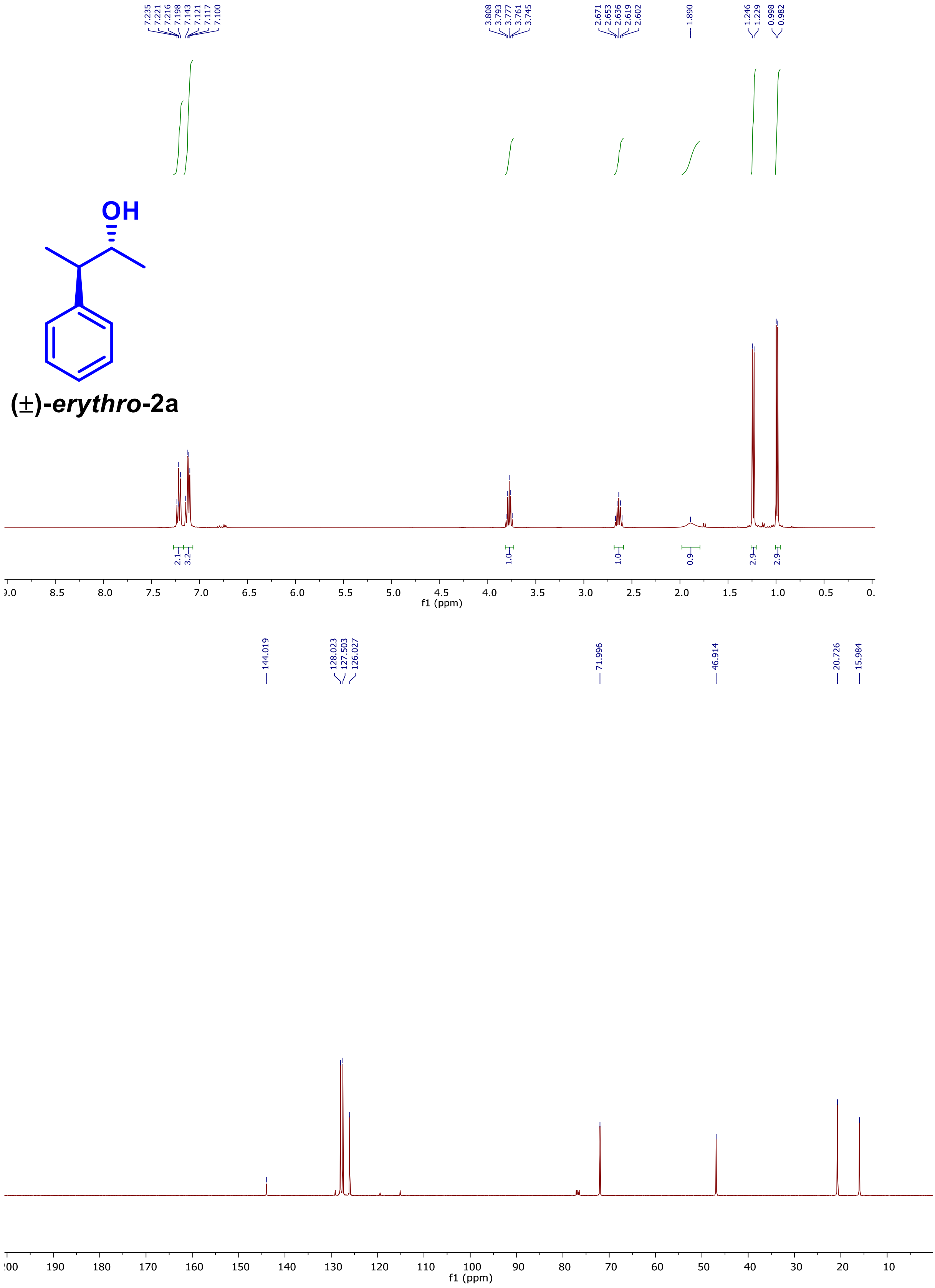

Figure S19. NMR spectra of (±)-*erythro*-3-phenyl-2-butanol in $CDCl_3$. *Top*: ^{1}H NMR (400 MHz), *Bottom:* ^{13}C NMR (100 MHz).

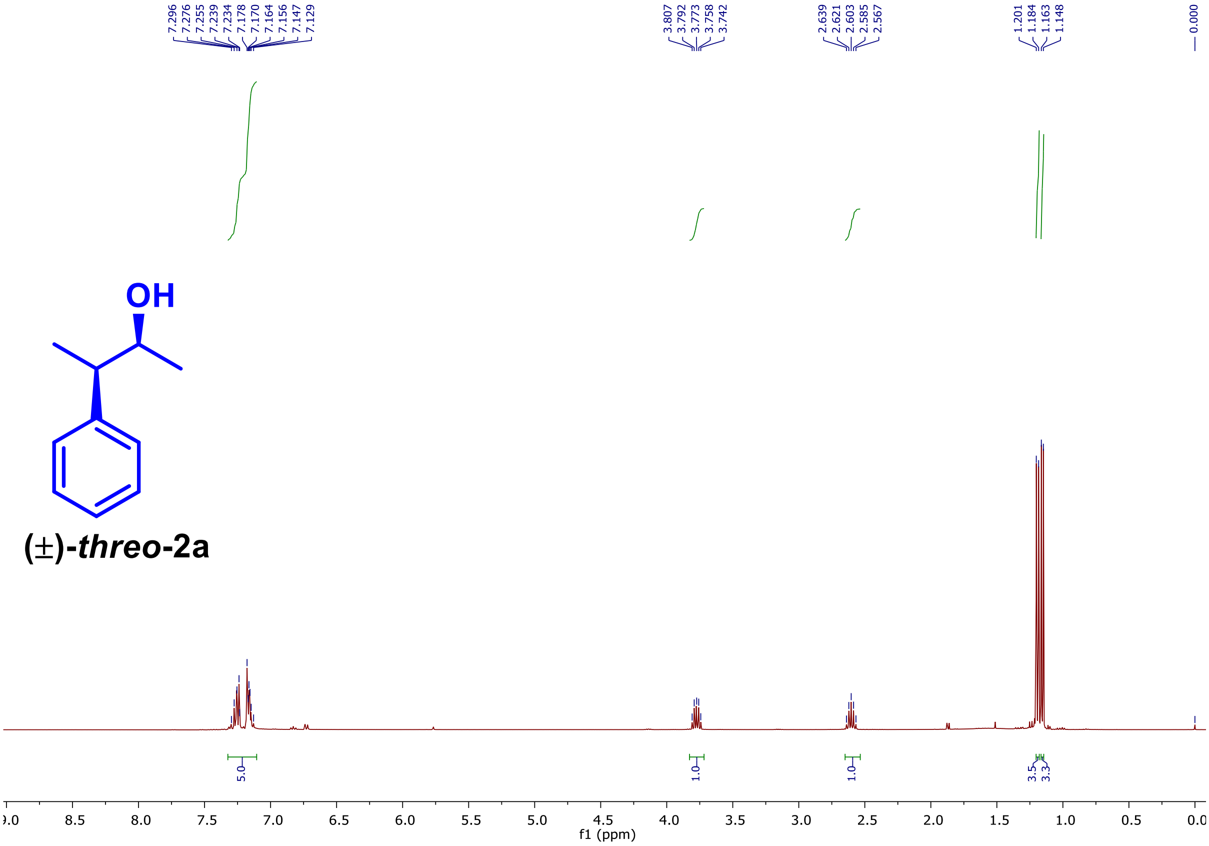

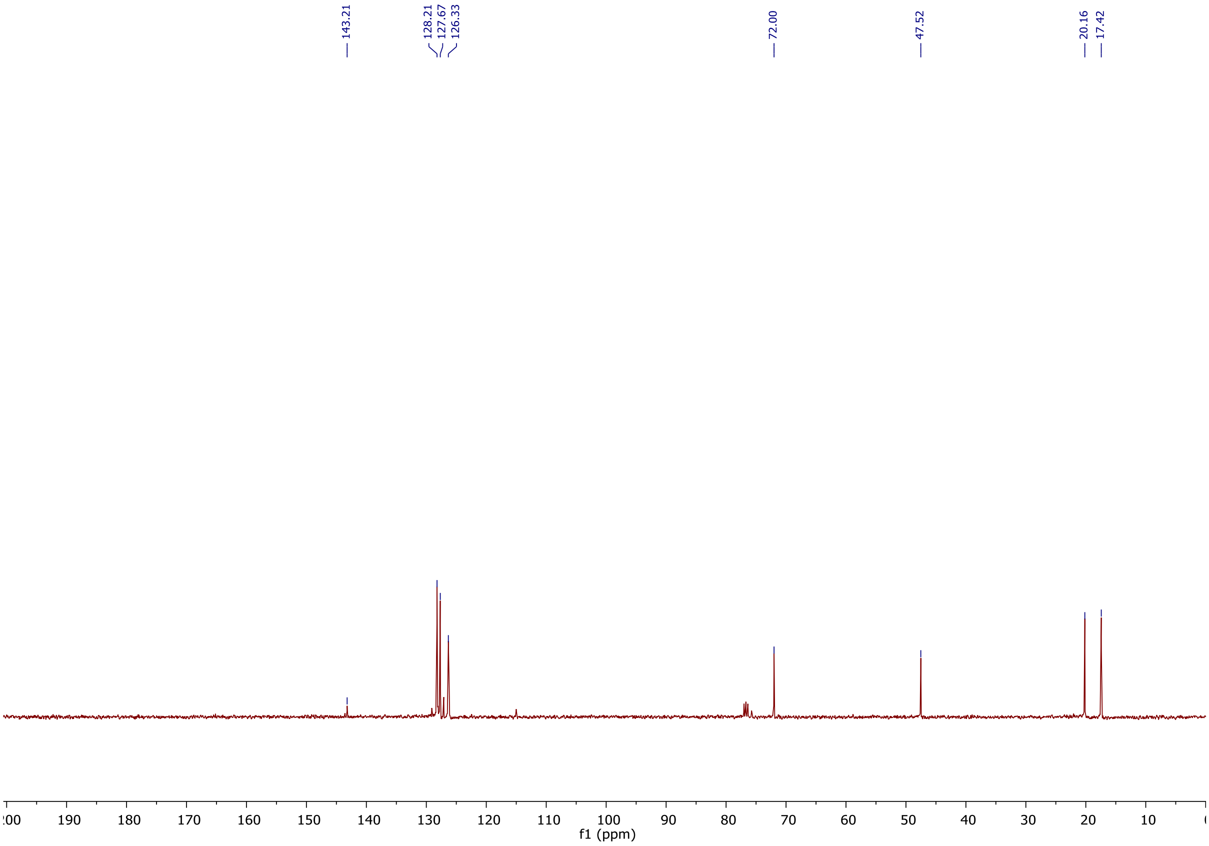

Figure S20. NMR spectra of (±)-*threo*-3-phenyl-2-butanol, (±)-*threo*-**2a**, (±)-*erythro*-**2a** in $CDCl_3$. *Top*: ^{1}H NMR (400 MHz), *Bottom:* ^{13}C NMR (100 MHz).

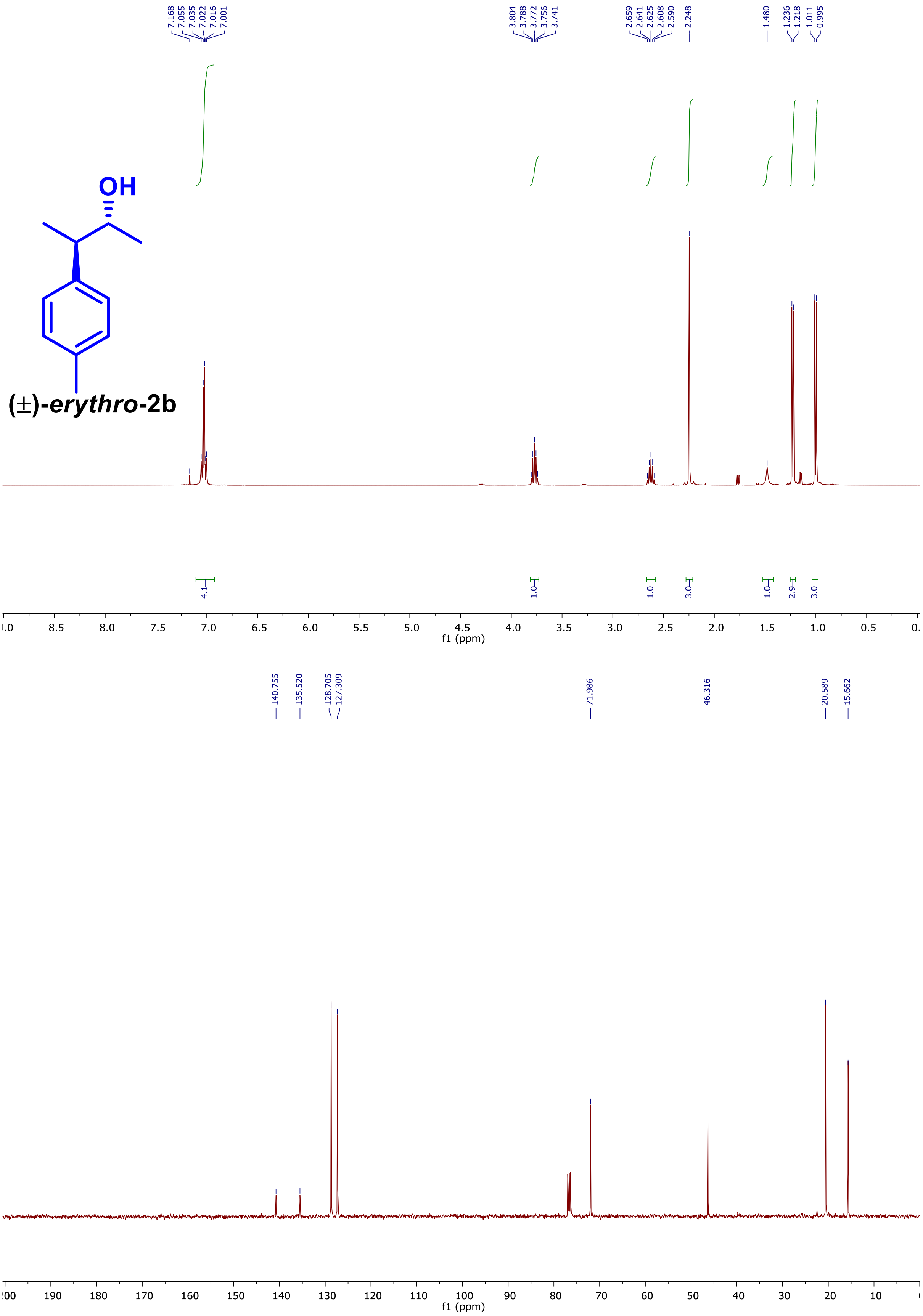

Figure S21. NMR spectra of (±)-*erythro*-3-(*p*-tolyl)-2-butanol, (±)-*erythro*-**2b** in $CDCl_3$. *Top*: ^1H NMR (400 MHz), *Bottom:* ^{13}C NMR (100 MHz).

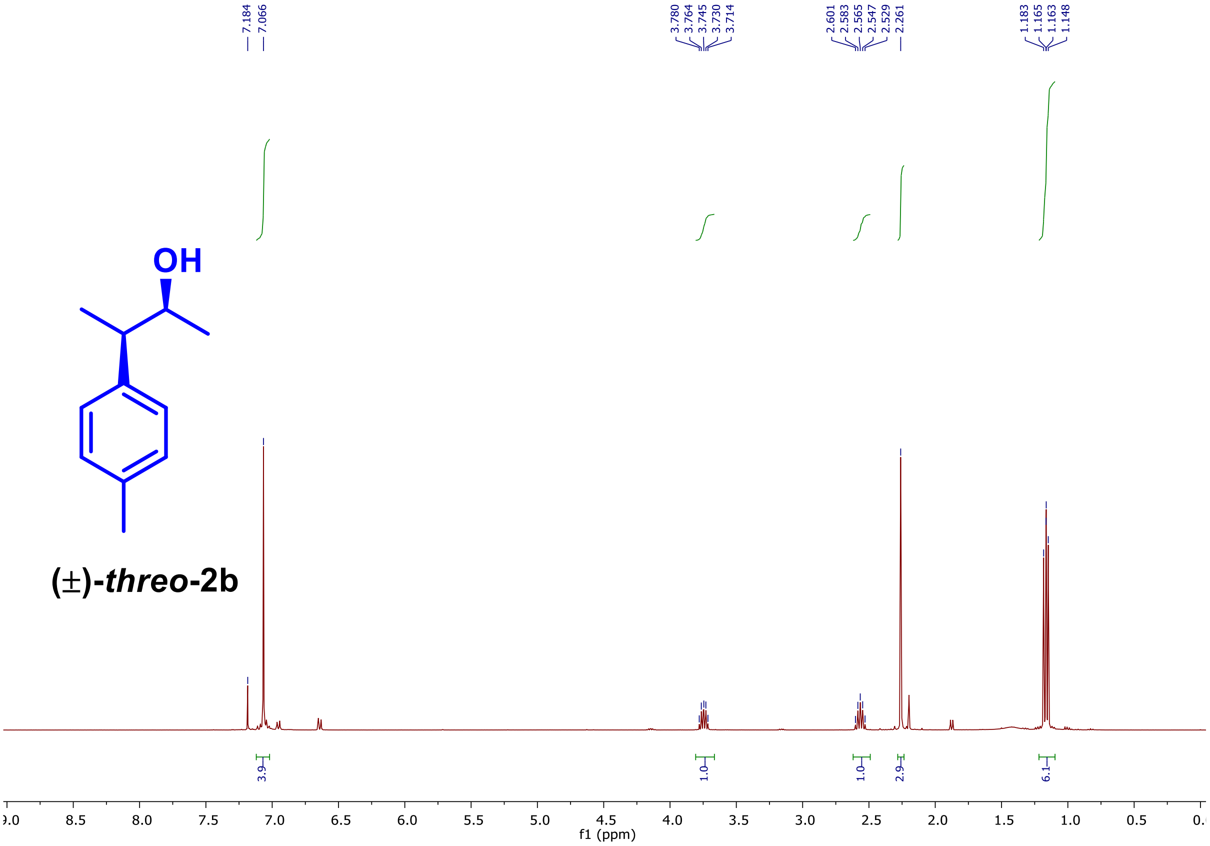

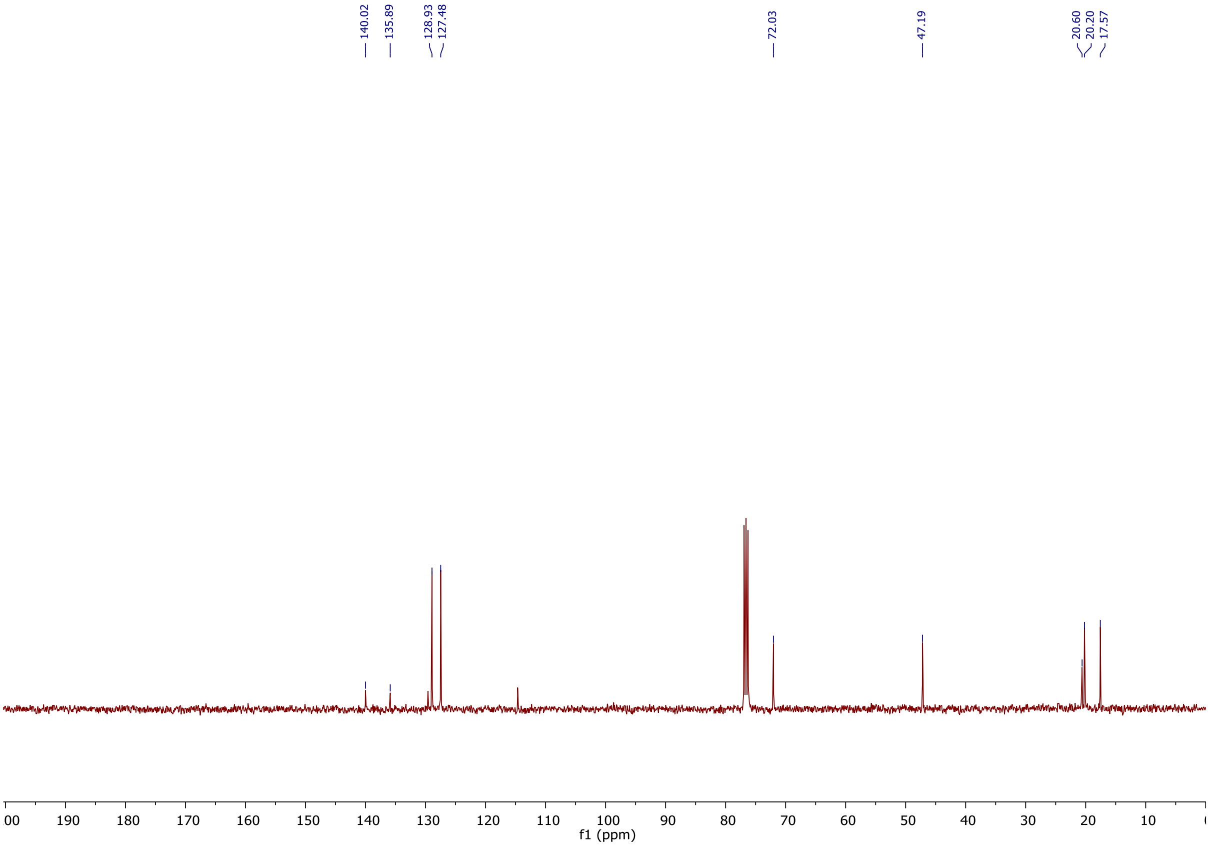

Figure S22. NMR spectra of (±)-*threo*-3-(*p*-tolyl)-2-butanol, (±)-*threo*-**2b** in $CDCl_3$. *Top*: ^1H NMR (400 MHz), *Bottom:* ^{13}C NMR (100 MHz).

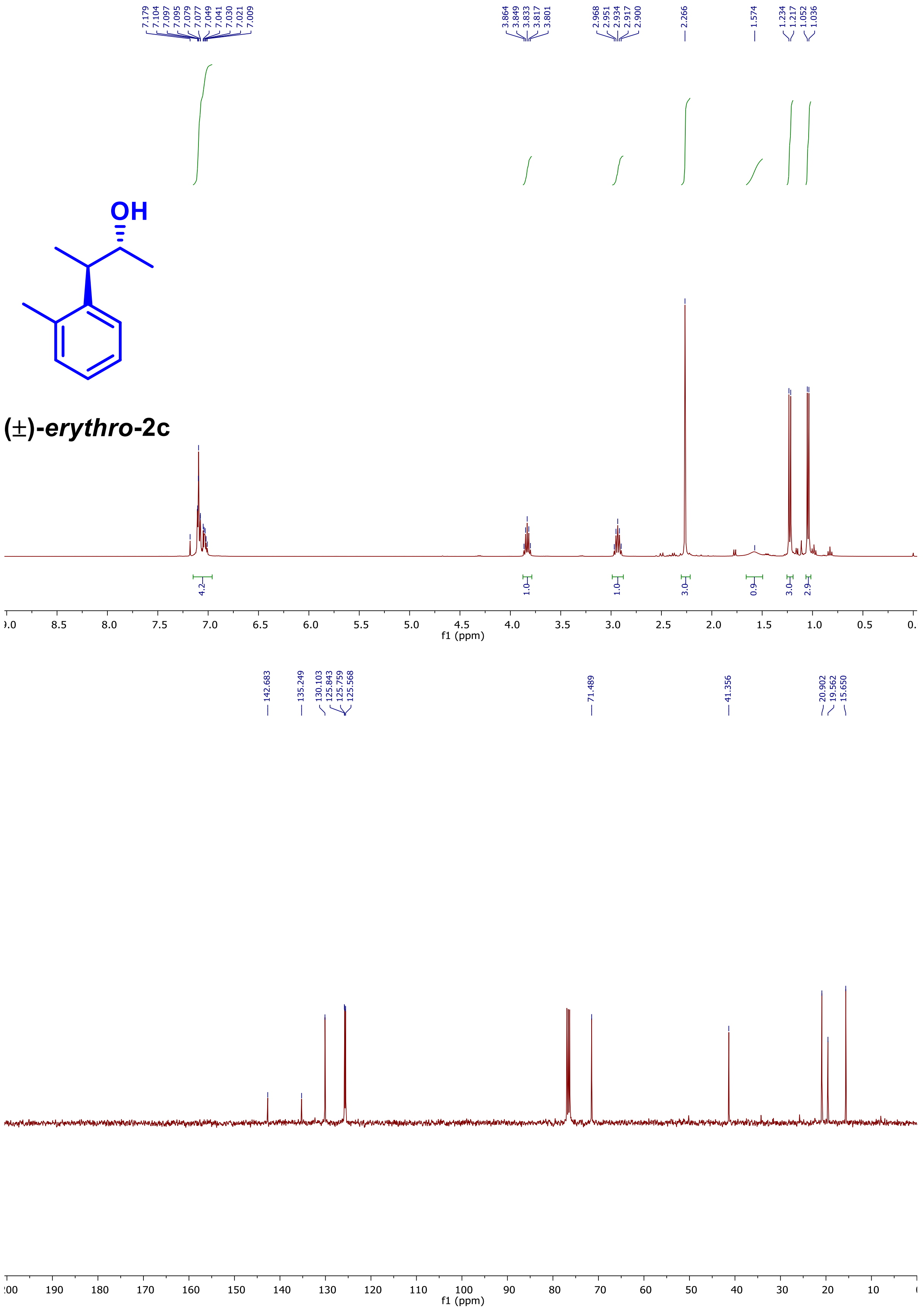

Figure S23. NMR spectra of (±)-*erythro*-3-(*o*-tolyl)-2-bromobutane, (±)-*erythro*-**2c** in $CDCl_3$. *Top*: ^{1}H NMR (400 MHz), *Bottom:* ^{13}C NMR (100 MHz).

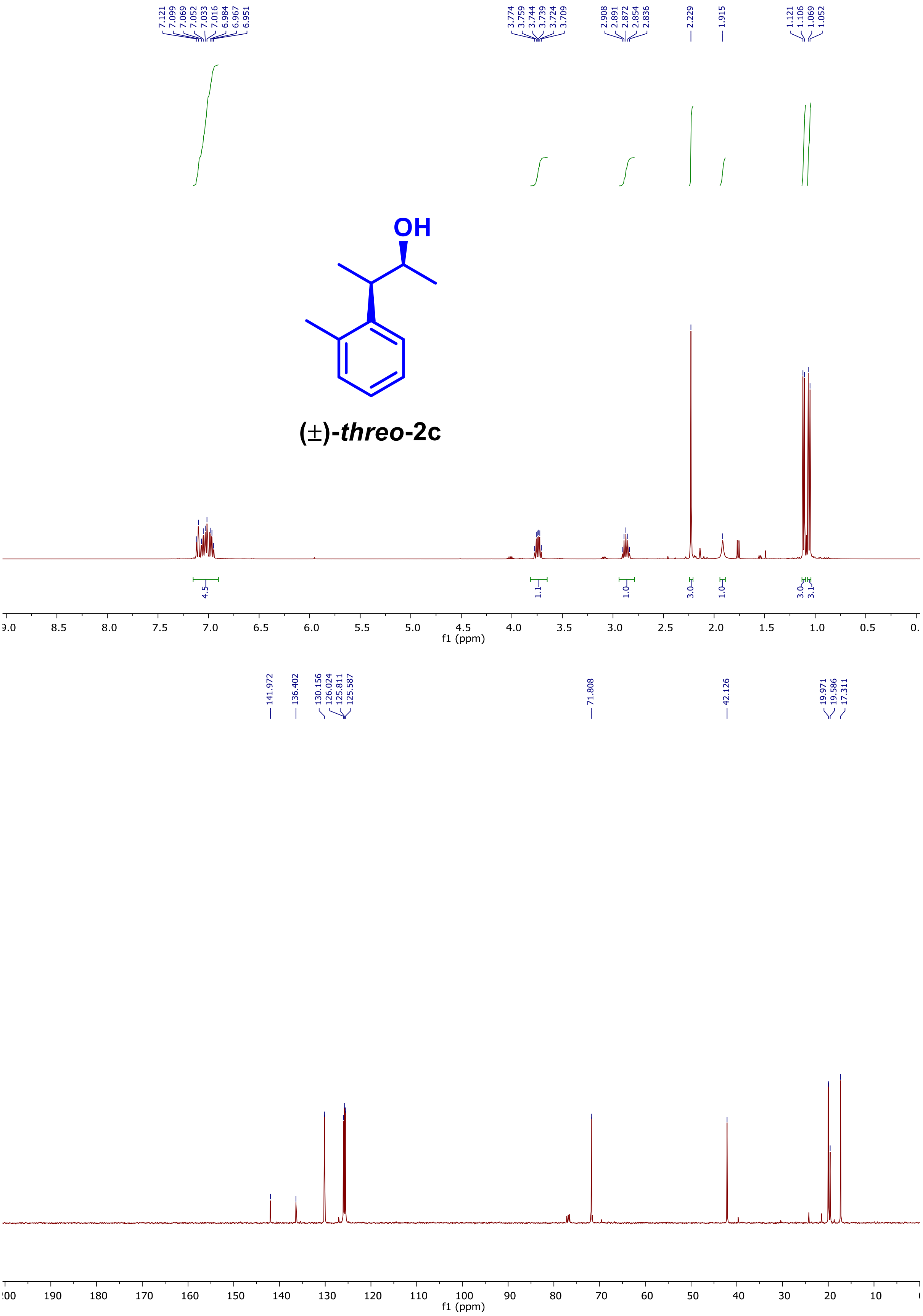

Figure S24. NMR spectra of (±)-*threo*-3-(*o*-tolyl)-2-butanol, (±)-*threo*-**2c** in $CDCl_3$. *Top*: ^{1}H NMR (400 MHz), *Bottom:* ^{13}C NMR (100 MHz).

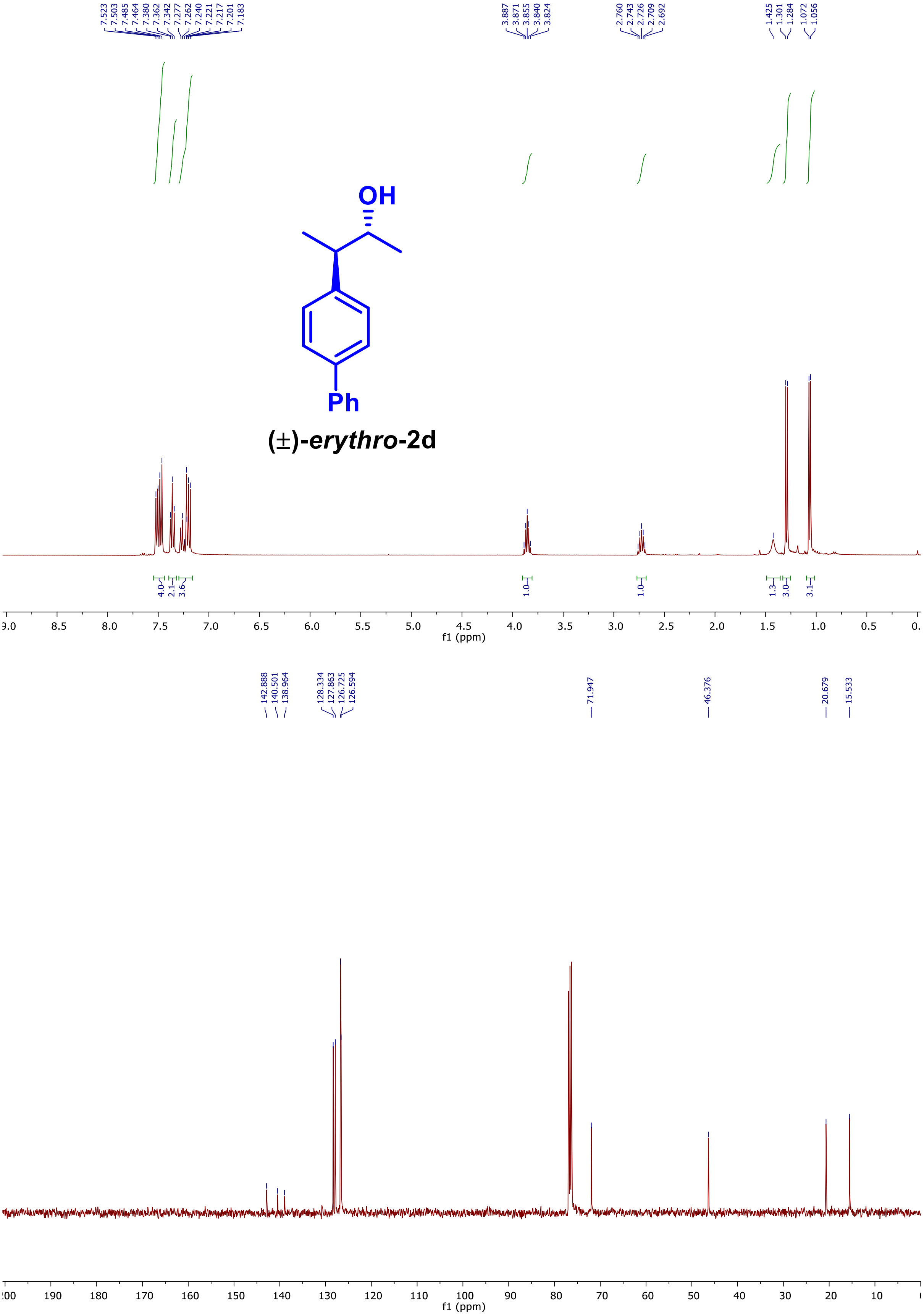

Figure S25. NMR spectra of (±)-*erythro*-3-([1,1'-biphenyl]-4-yl)-2-bromobutane, (±)-*erythro*-**2d** in $CDCl_3$. *Top*: ^{1}H NMR (400 MHz), *Bottom:* ^{13}C NMR (100 MHz).

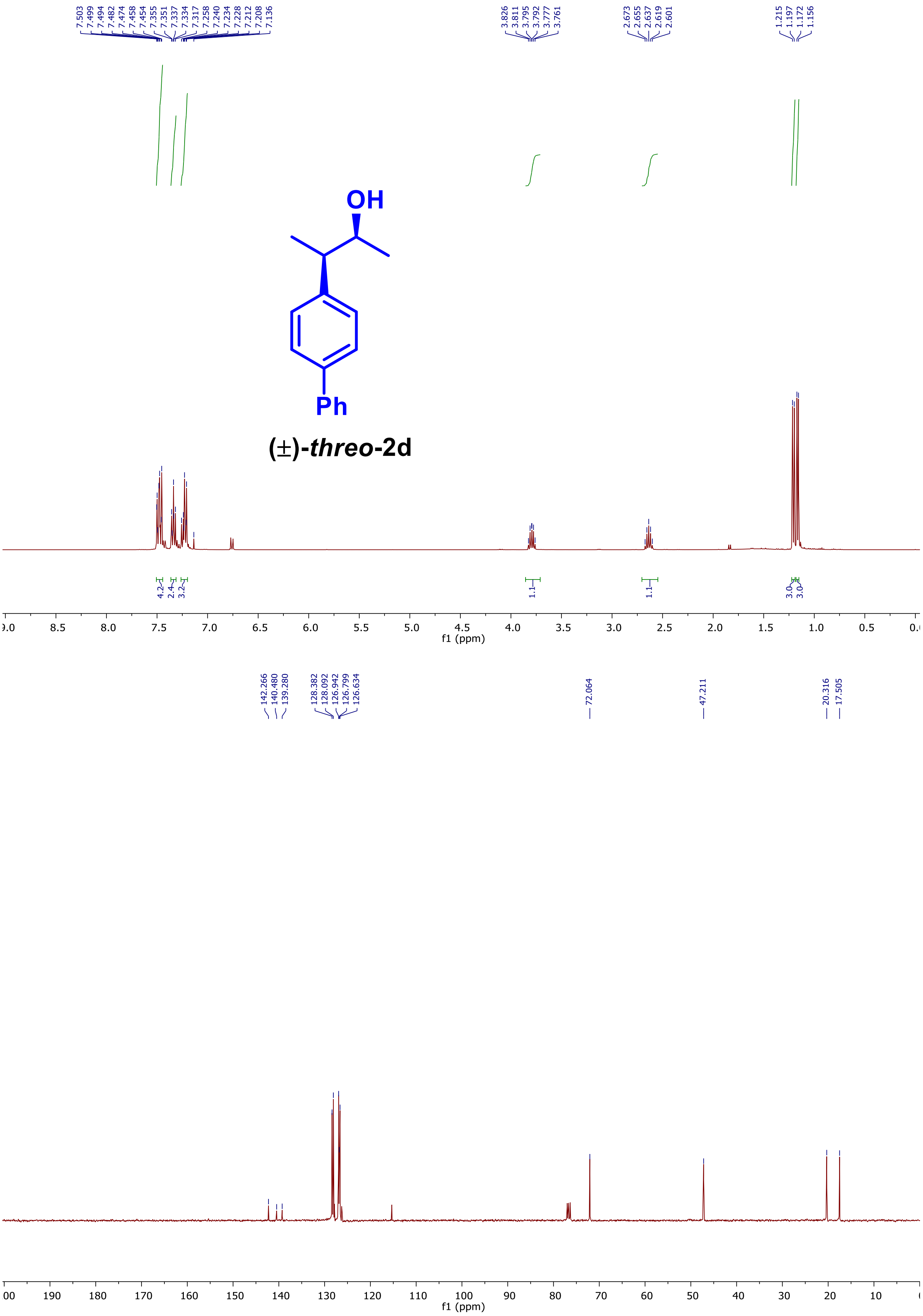

Figure S26. NMR spectra of (±)-*threo*-3-([1,1'-biphenyl]-4-yl)-2-bromobutane, (±)-*threo*-**2d** in $CDCl_3$. *Top*: ^{1}H NMR (400 MHz), *Bottom:* ^{13}C NMR (100 MHz).

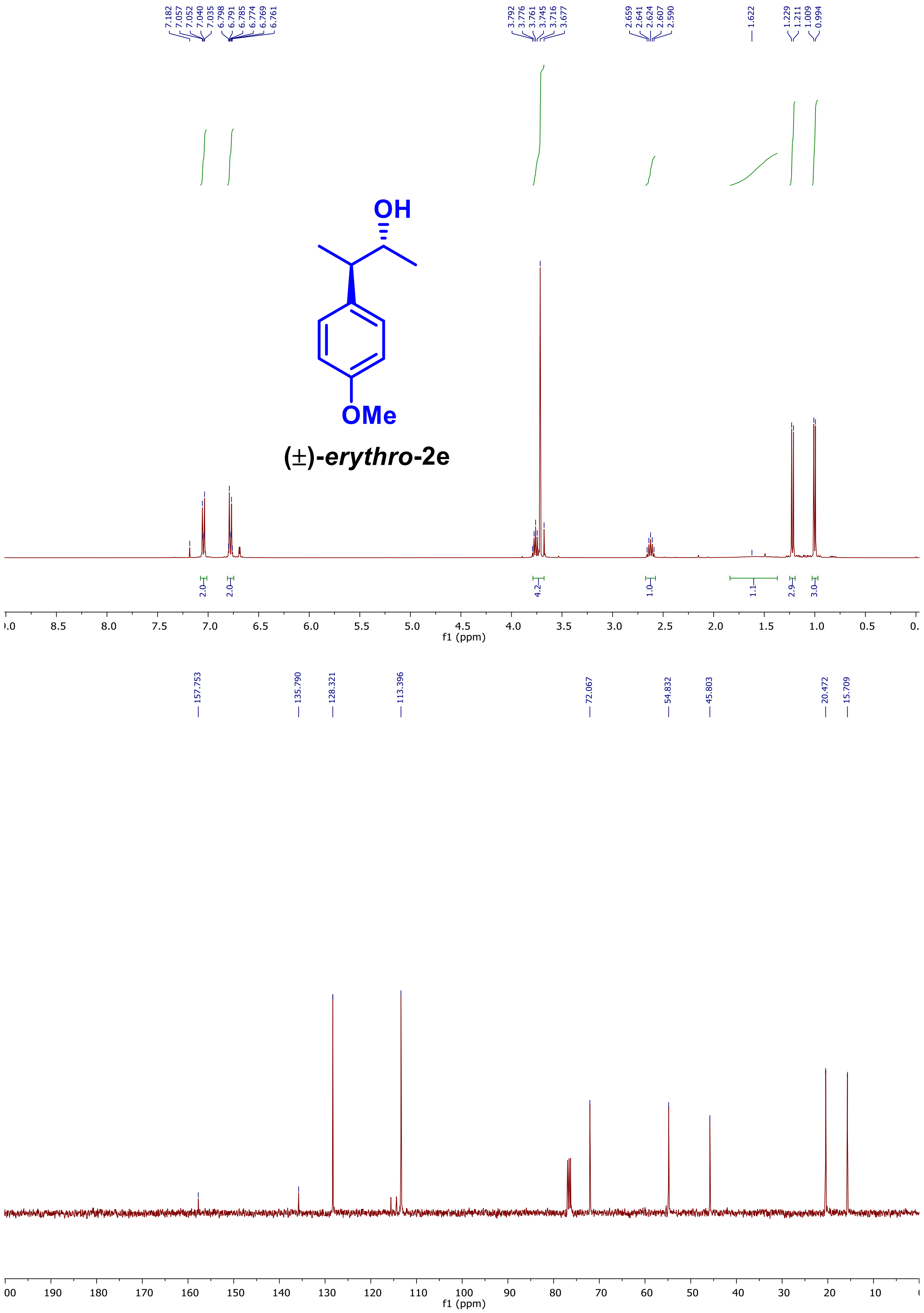

Figure S27. NMR spectra of (±)-*erythro*-3-(4-methoxyphenyl)-2-butanol, (±)-*erythro*-**2e** in $CDCl_3$. *Top*: ^{1}H NMR (400 MHz), *Bottom:* ^{13}C NMR (100 MHz).

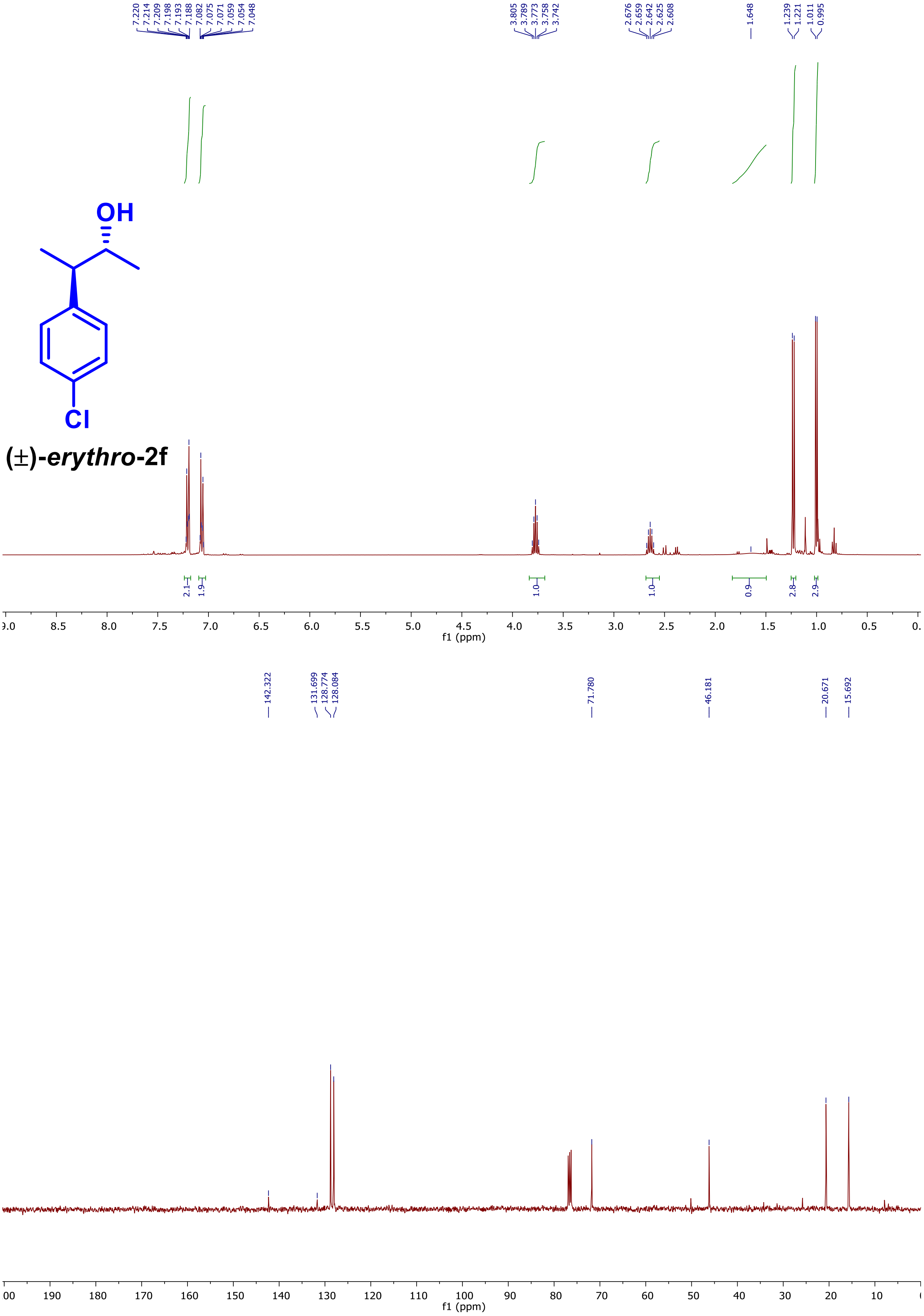

Figure S28. NMR spectra of (±)-*erythro*-3-(4-chlorophenyl)-2-butanol, (±)-*erythro*-**2f** in $CDCl_3$. *Top*: ^1H NMR (400 MHz), *Bottom:* ^{13}C NMR (100 MHz).

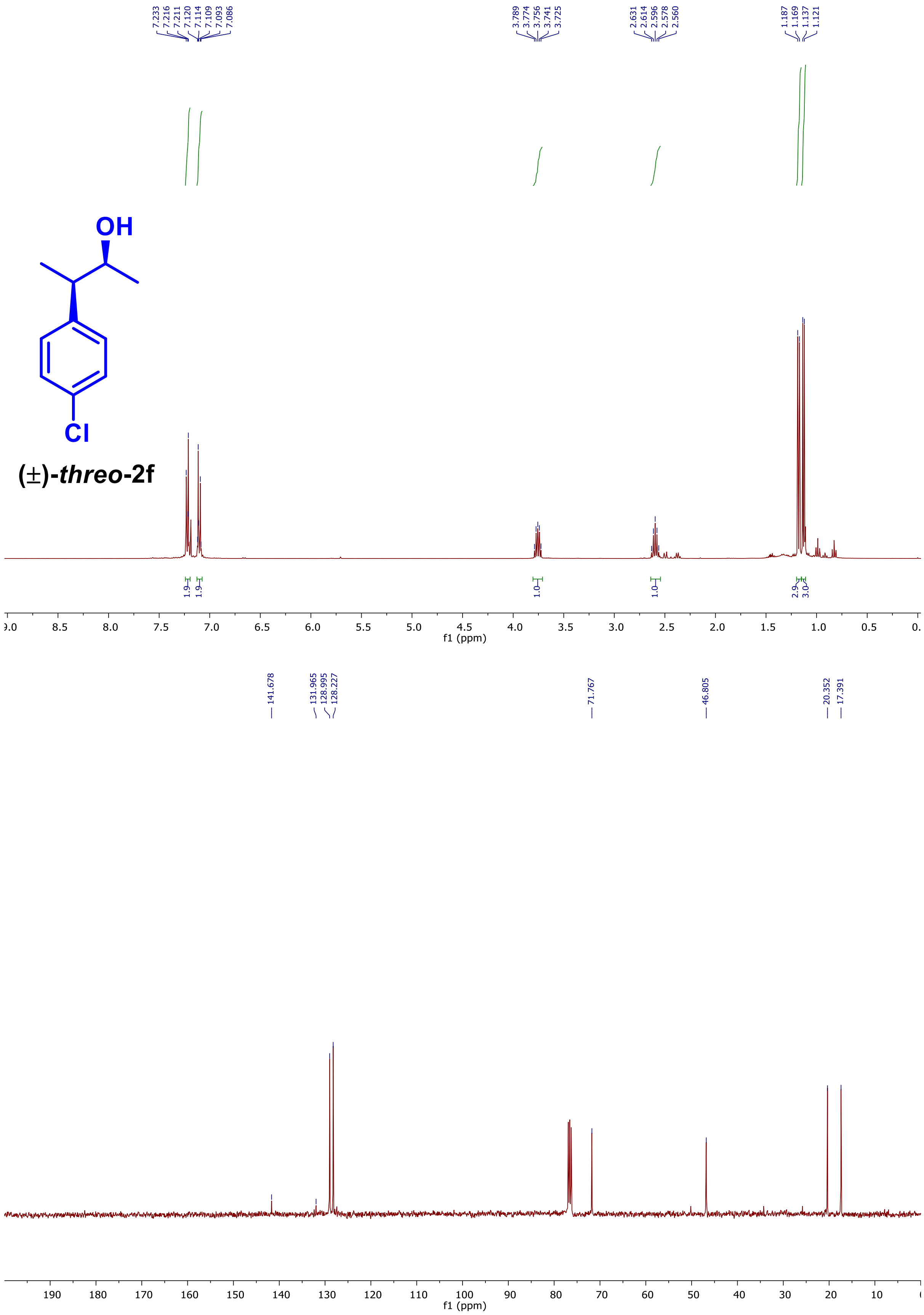

Figure S29. NMR spectra of (±)-*threo*-3-(4-chlorophenyl)-2-butanol, (±)-*threo*-**2f** in $CDCl_3$. *Top*: ^{1}H NMR (400 MHz), *Bottom:* ^{13}C NMR (100 MHz).

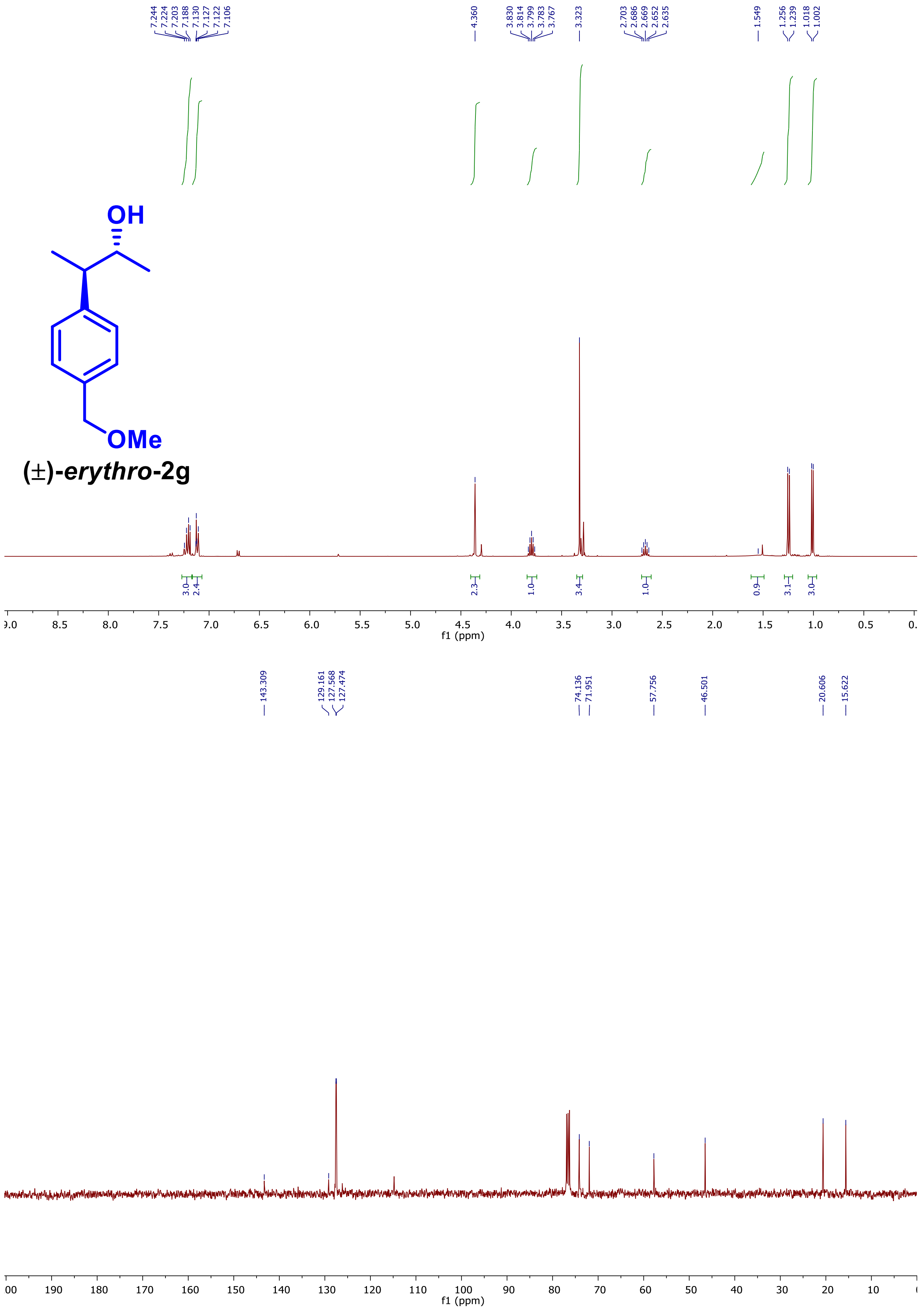

Figure S30. NMR spectra of (±)-*erythro*-3-(4-(methoxymethyl)phenyl)-2-butanol, (±)-*erythro*-**2g** in $CDCl_3$. *Top*: ^{1}H NMR (400 MHz), *Bottom:* ^{13}C NMR (100 MHz).

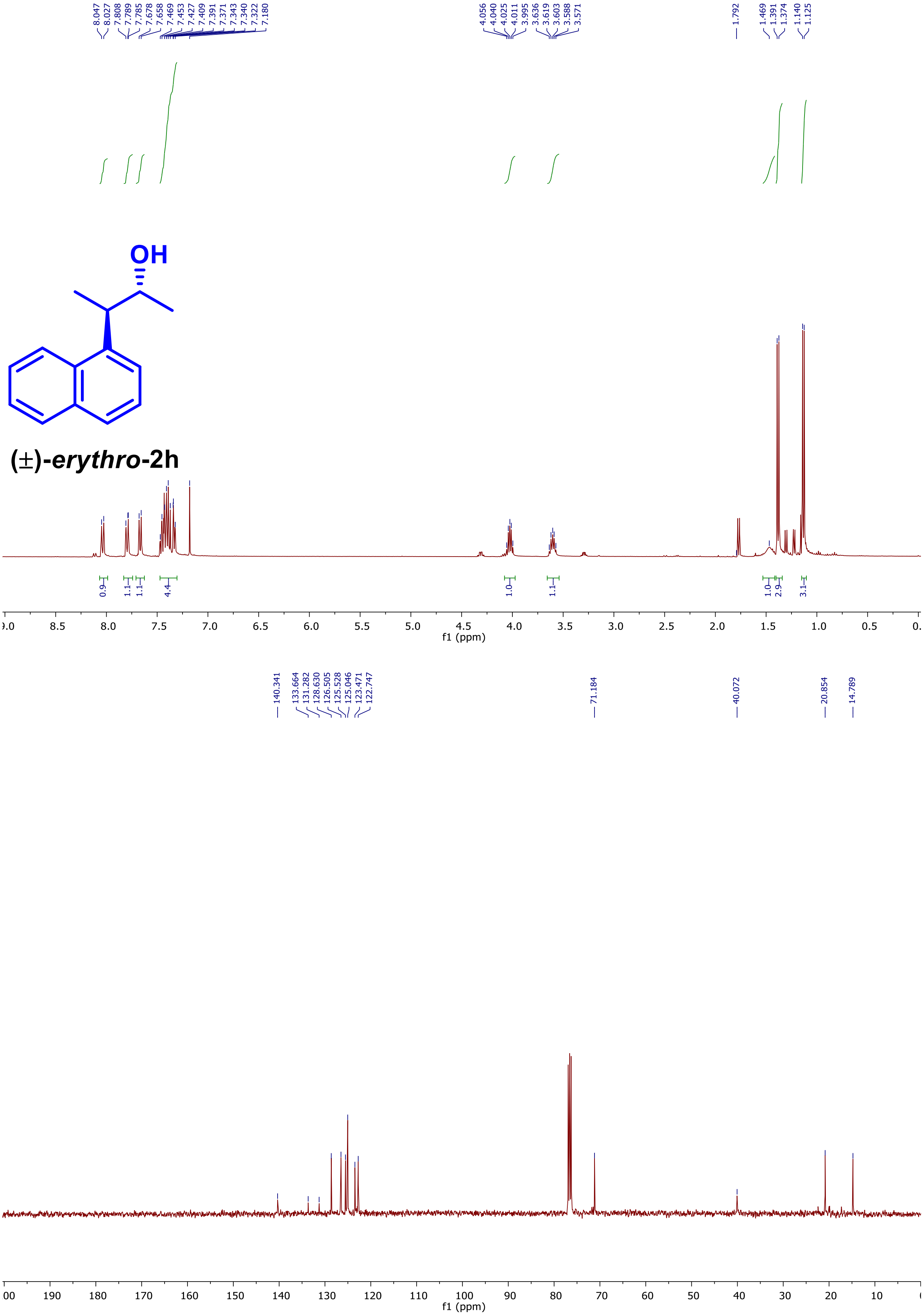

Figure S31. NMR spectra of (±)-*erythro*-3-(1-naphthyl)-2-butanol, (±)-*erythro*-**2h** in $CDCl_3$. *Top*: ^{1}H NMR (400 MHz), *Bottom:* ^{13}C NMR (100 MHz).

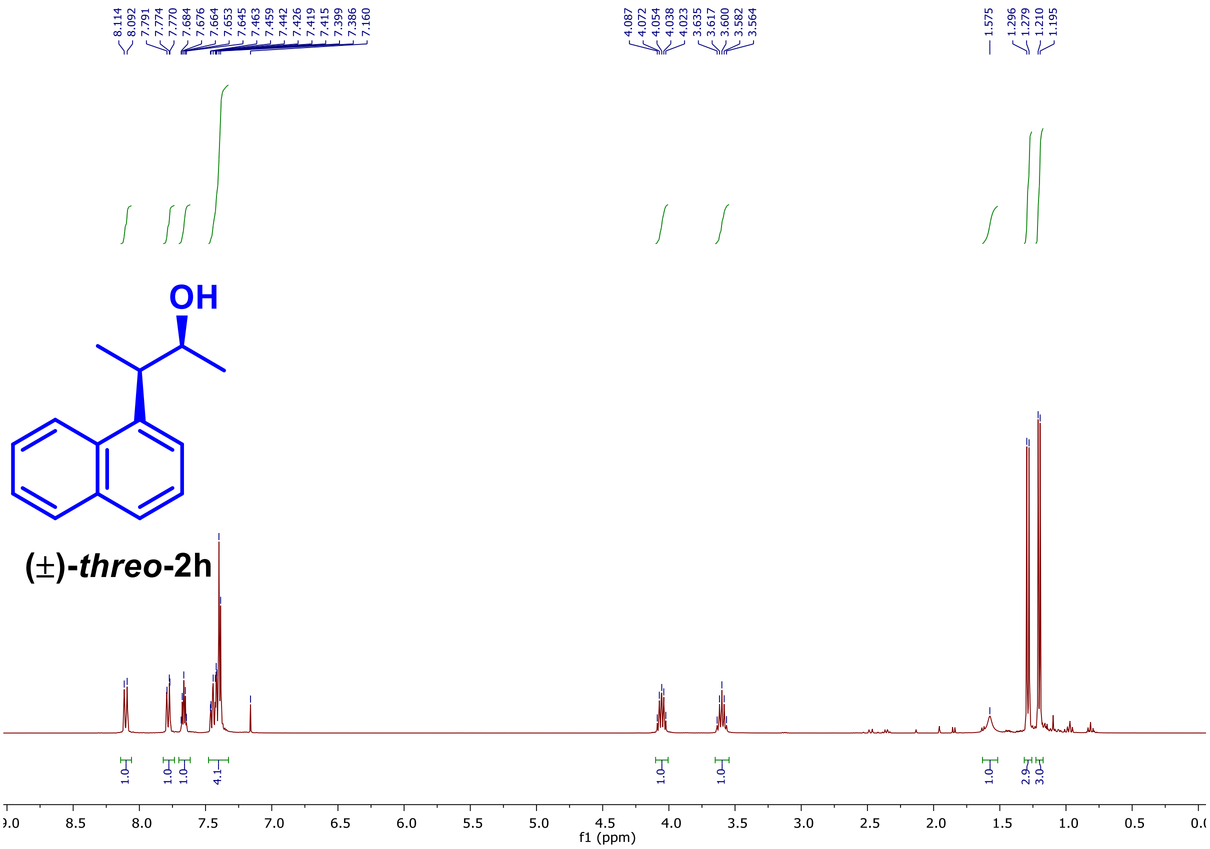

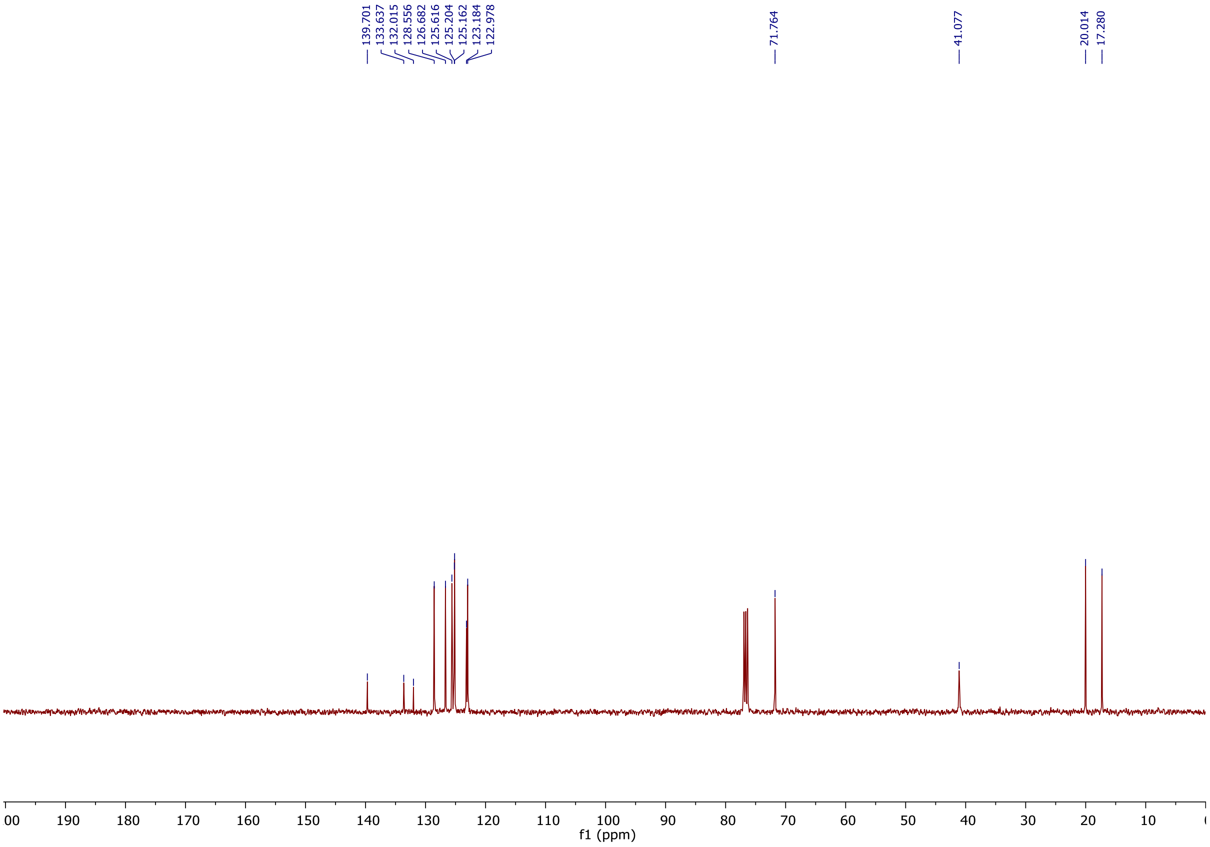

Figure S32. NMR spectra of (±)-*threo*-3-(1-naphthyl)-2-butanol, (±)-*threo*-**2h** in $CDCl_3$. *Top*: ^{1}H NMR (400 MHz), *Bottom:* ^{13}C NMR (100 MHz).

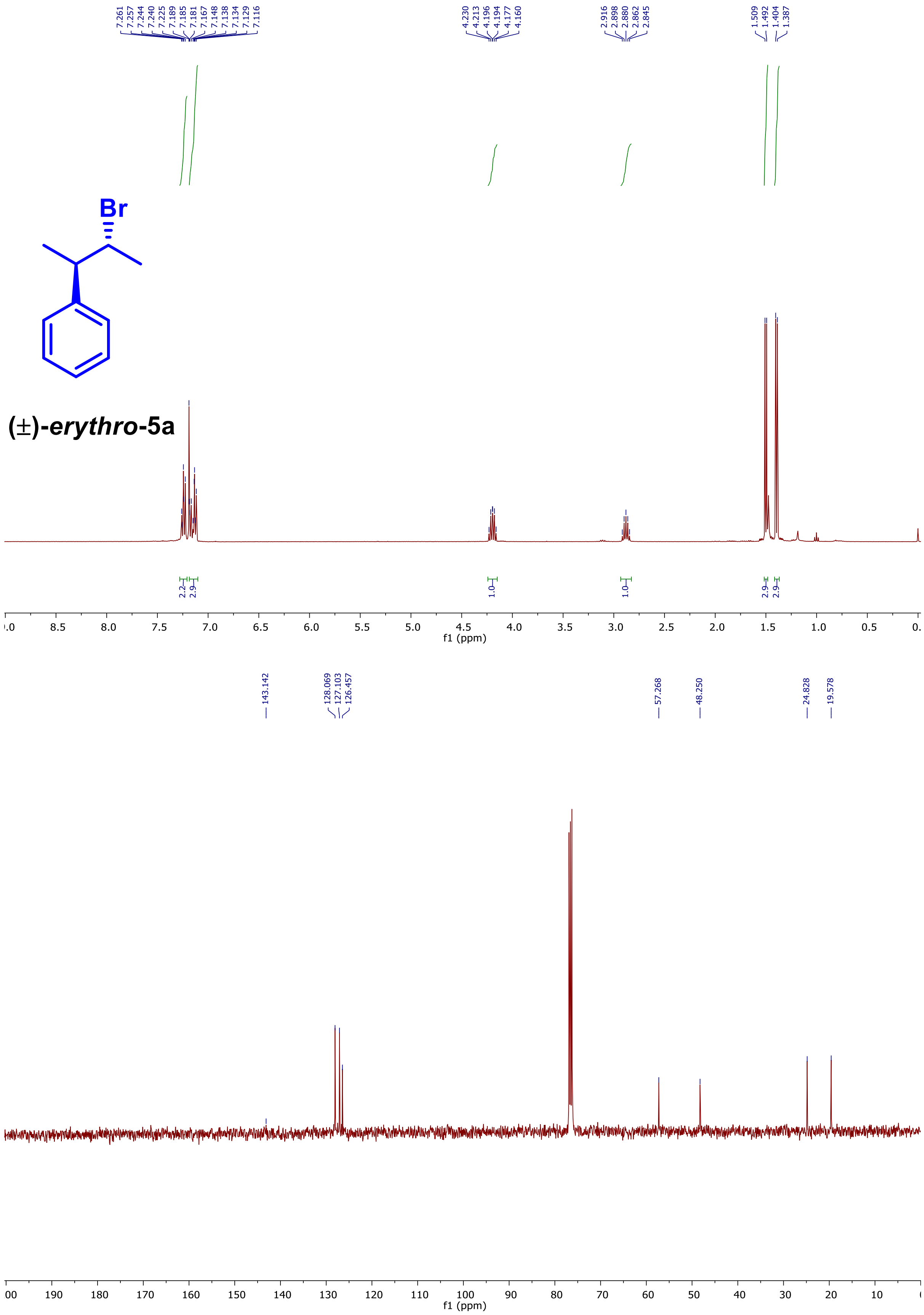

Figure S33. NMR spectra of (±)-*erythro*-3-phenyl-2-bromobutane, (±)-*erythro*-**5a** in $CDCl_3$. *Top*: ^{1}H NMR (400 MHz), *Bottom:* ^{13}C NMR (100 MHz).

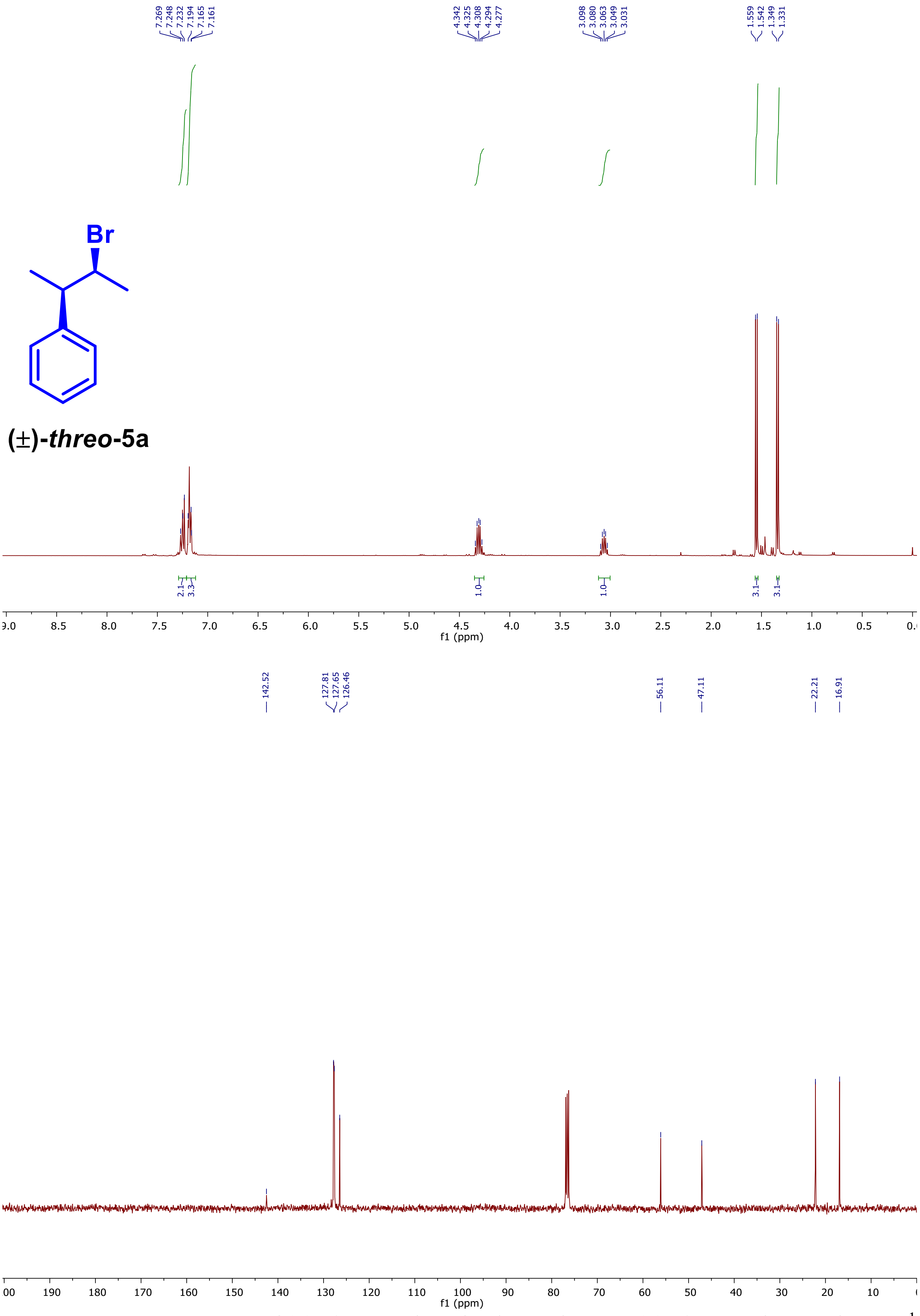

Figure S34. NMR spectra of (±)-*threo*-3-phenyl-2-bromobutane, (±)-*threo*-**5a** in $CDCl_3$. *Top*: ^1H NMR (400 MHz), *Bottom:* ^{13}C NMR (100 MHz).

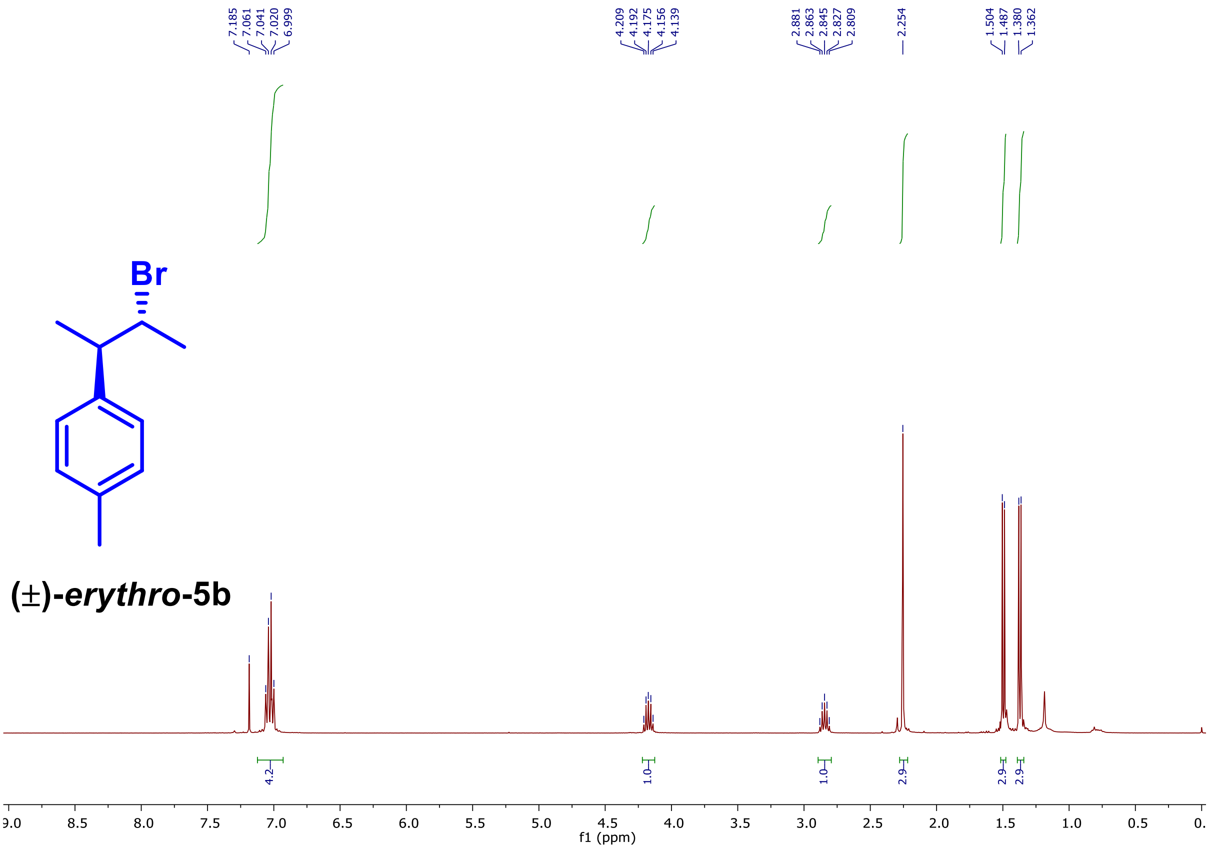

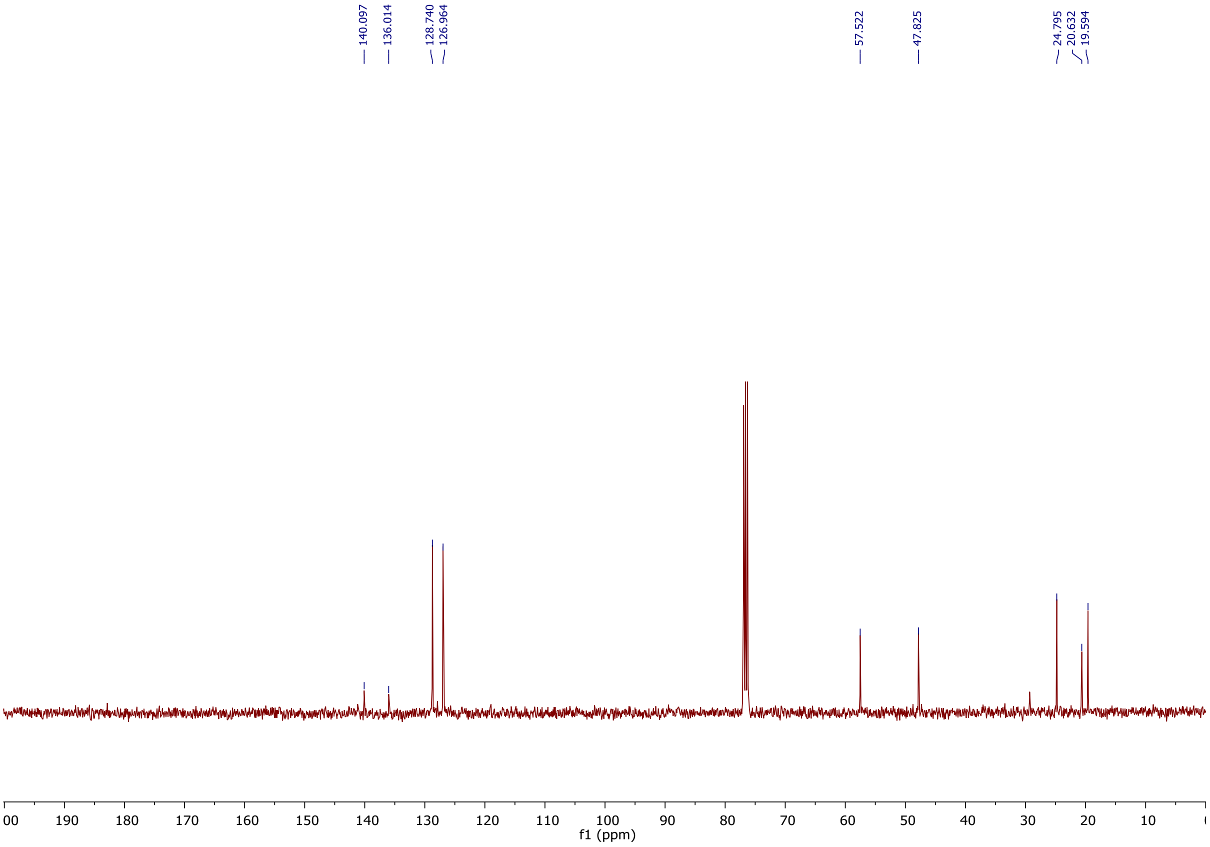

Figure S35. NMR spectra of (±)-*erythro*-3-(*p*-tolyl)-2-bromobutanel, (±)-*erythro*-**5b** in $CDCl_3$. *Top*: ^{1}H NMR (400 MHz), *Bottom:* ^{13}C NMR (100 MHz).

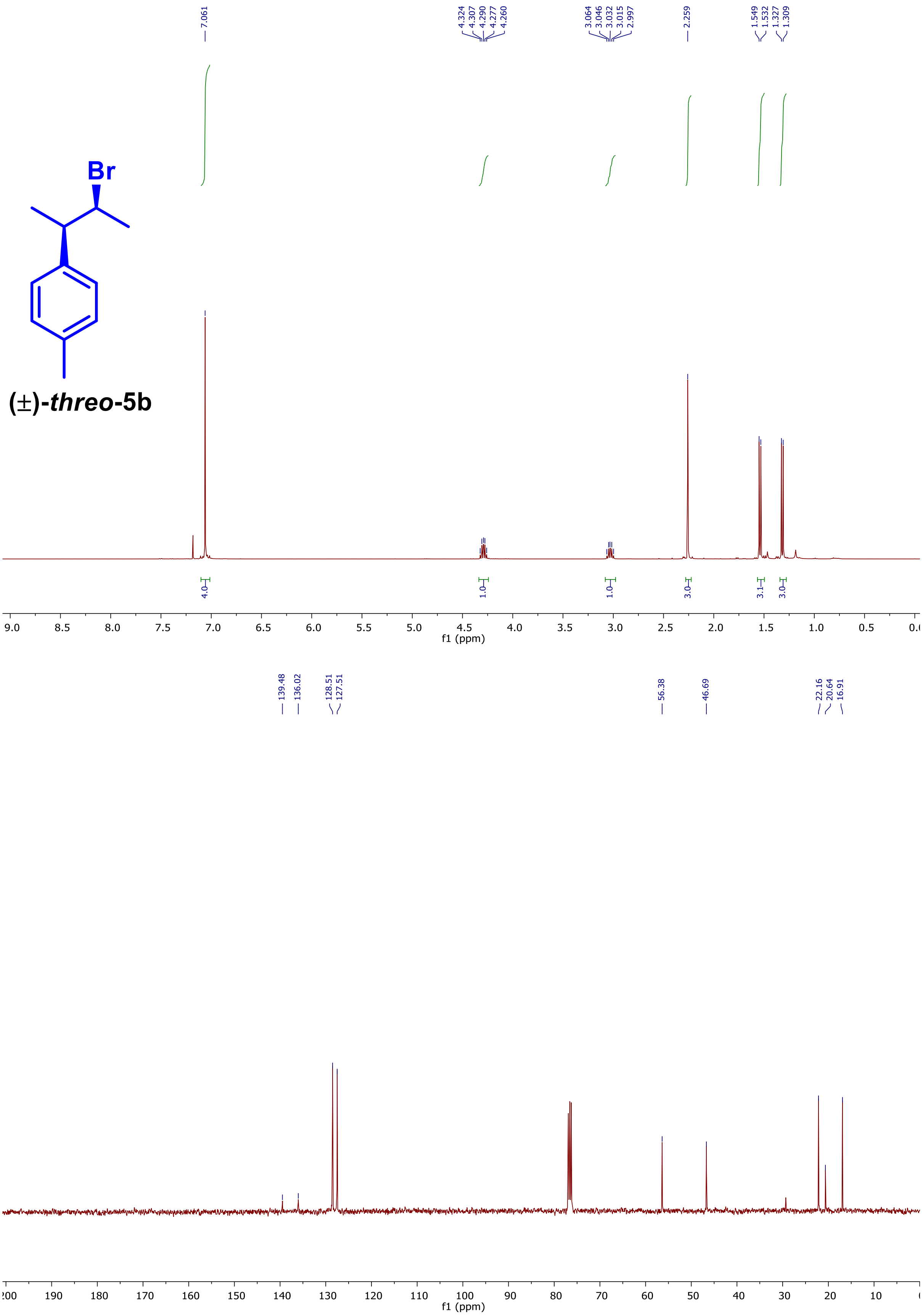

Figure S36. NMR spectra of (±)-*threo*-3-(*p*-tolyl)-2-bromobutane, (±)-*threo*-**5b** in $CDCl_3$. *Top*: ^{1}H NMR (400 MHz), *Bottom:* ^{13}C NMR (100 MHz).

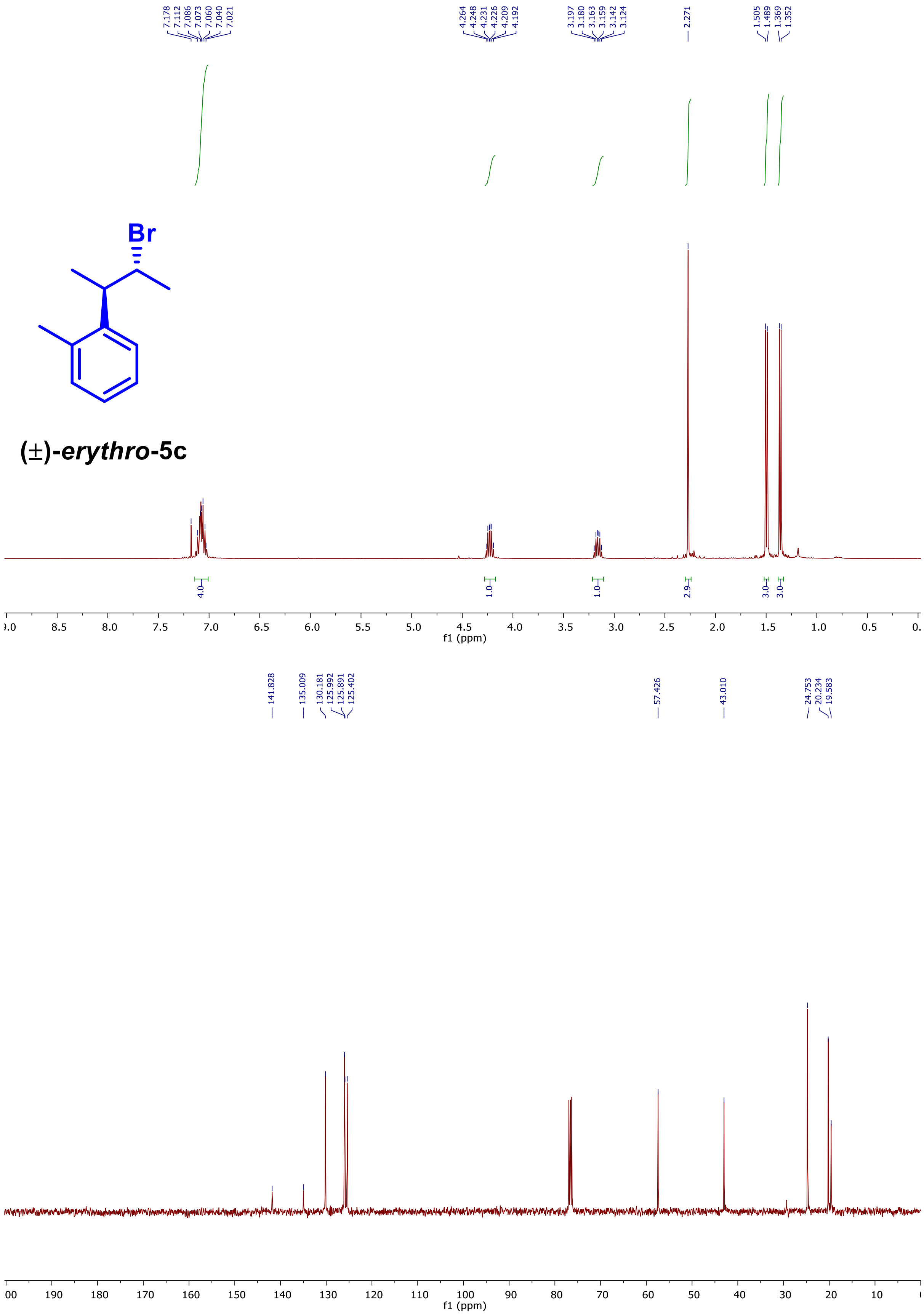

Figure S37. NMR spectra of (±)-*erythro*-3-(*o*-tolyl)-2-bromobutane, (±)-*erythro*-**5c** in $CDCl_3$. *Top*: ^{1}H NMR (400 MHz), *Bottom:* ^{13}C NMR (100 MHz).

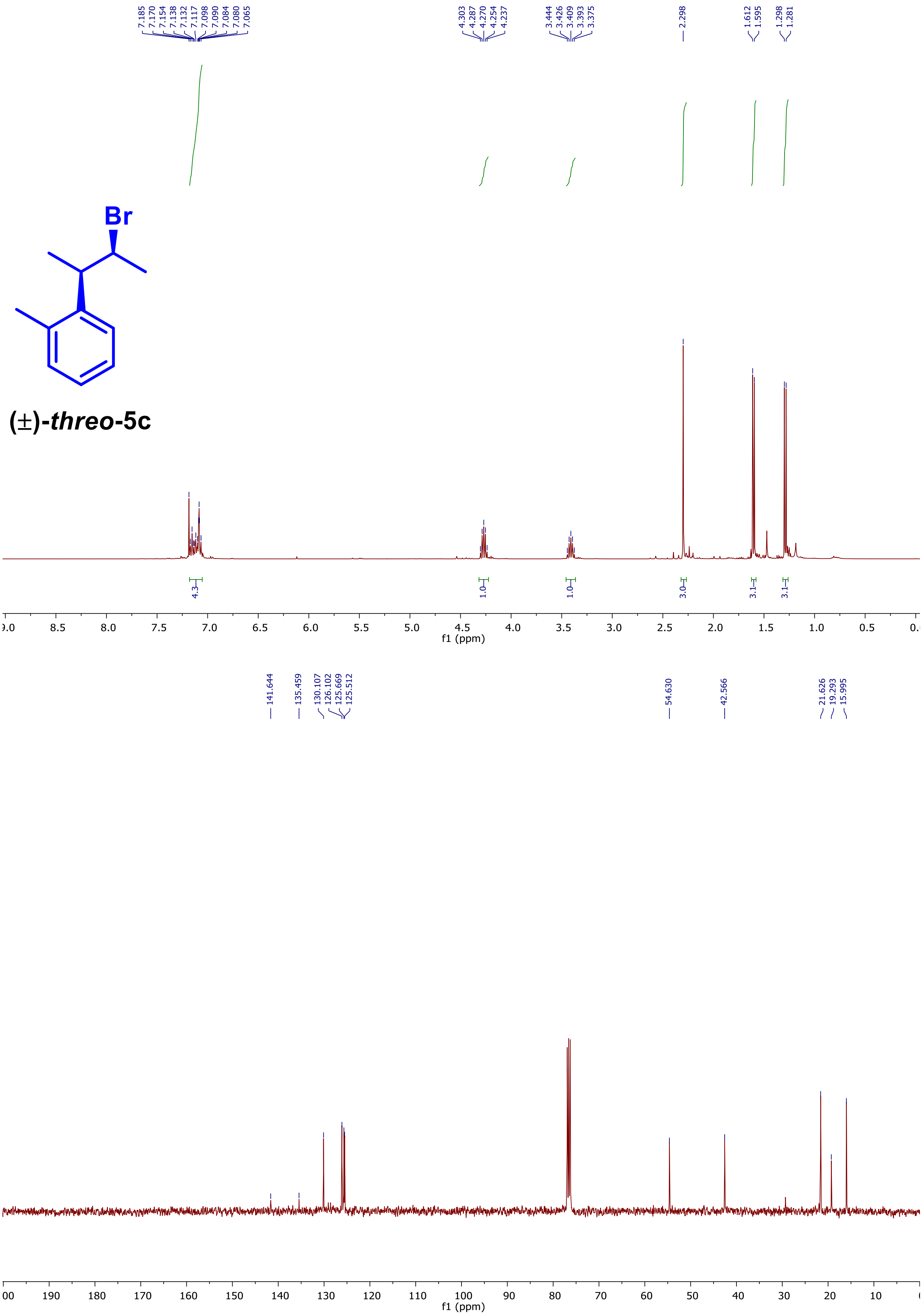

Figure S38. NMR spectra of (±)-*threo*-3-(*o*-tolyl)-2-bromobutane, (±)-*threo*-**5c** in $CDCl_3$. *Top*: ^{1}H NMR (400 MHz), *Bottom:* ^{13}C NMR (100 MHz).

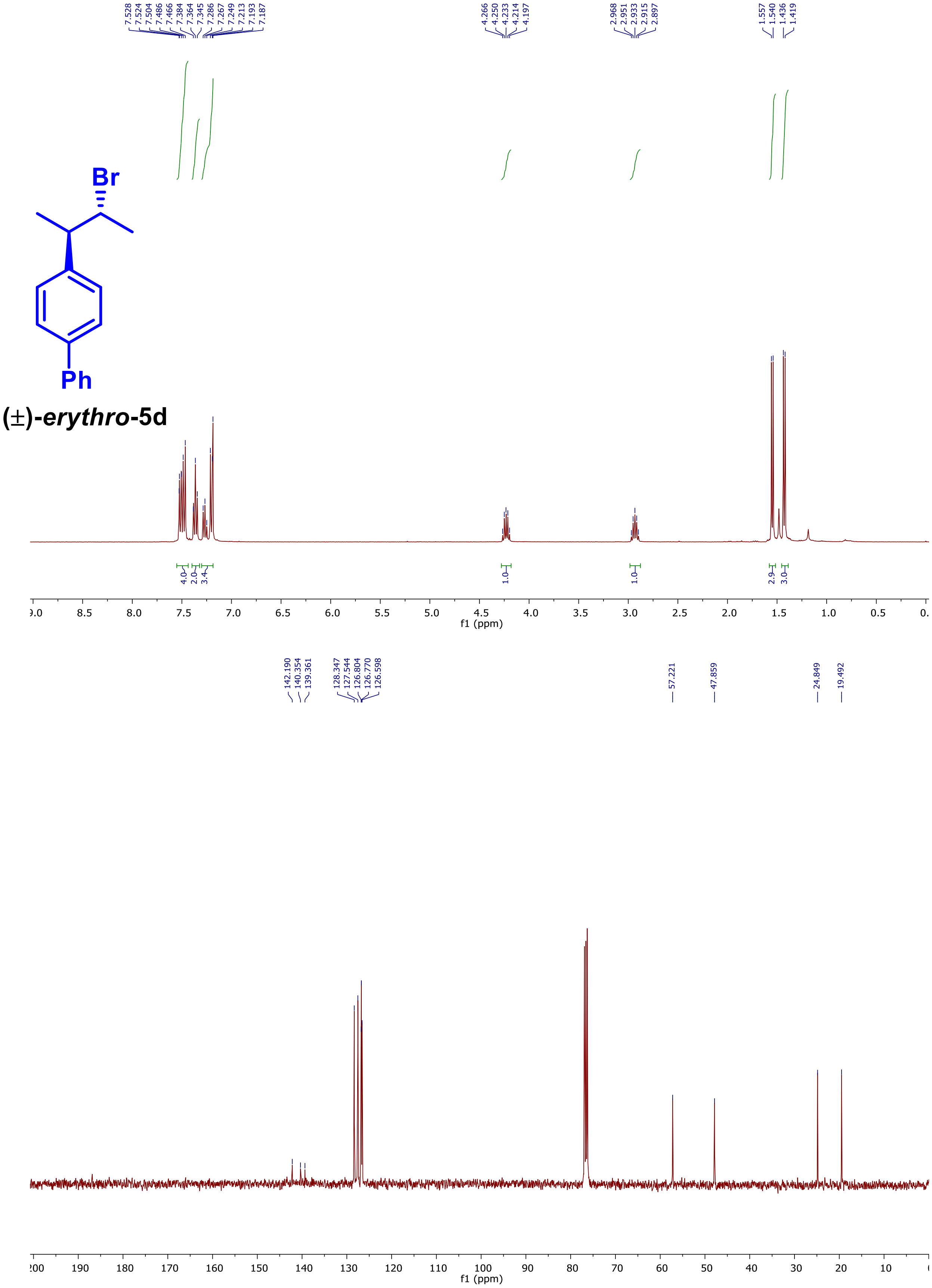

Figure S39. NMR spectra of (±)-*erythro*-3-([1,1'-biphenyl]-4-yl)-2-bromobutane, (±)-*erythro*-**5d** in $CDCl_3$. *Top*: ^{1}H NMR (400 MHz), *Bottom:* ^{13}C NMR (100 MHz).

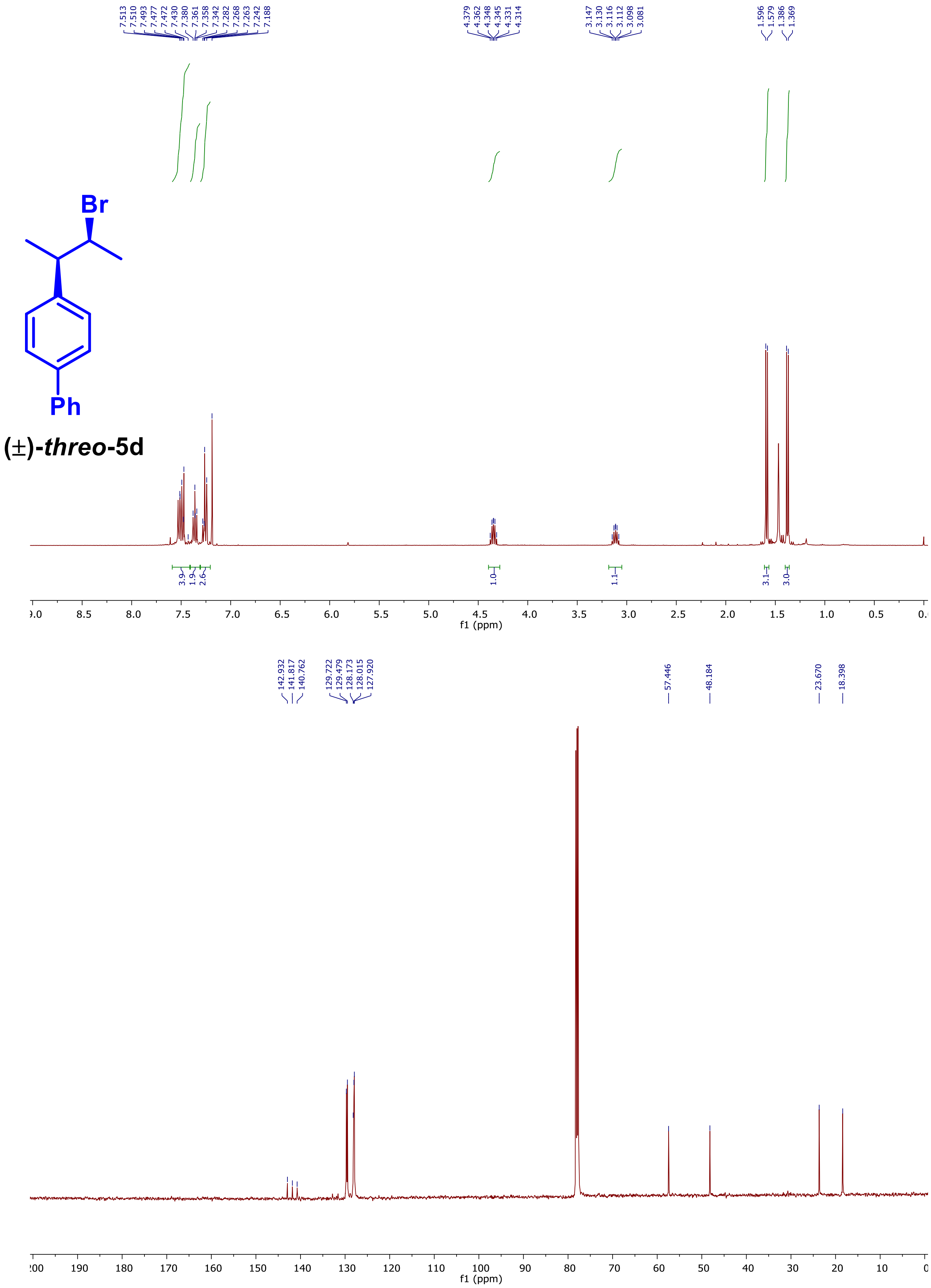

Figure S40. NMR spectra of (±)-*threo*-3-([1,1'-biphenyl]-4-yl)-2-bromobutane, (±)-*threo*-**5d** in $CDCl_3$. *Top*: ^{1}H NMR (400 MHz), *Bottom:* ^{13}C NMR (100 MHz).

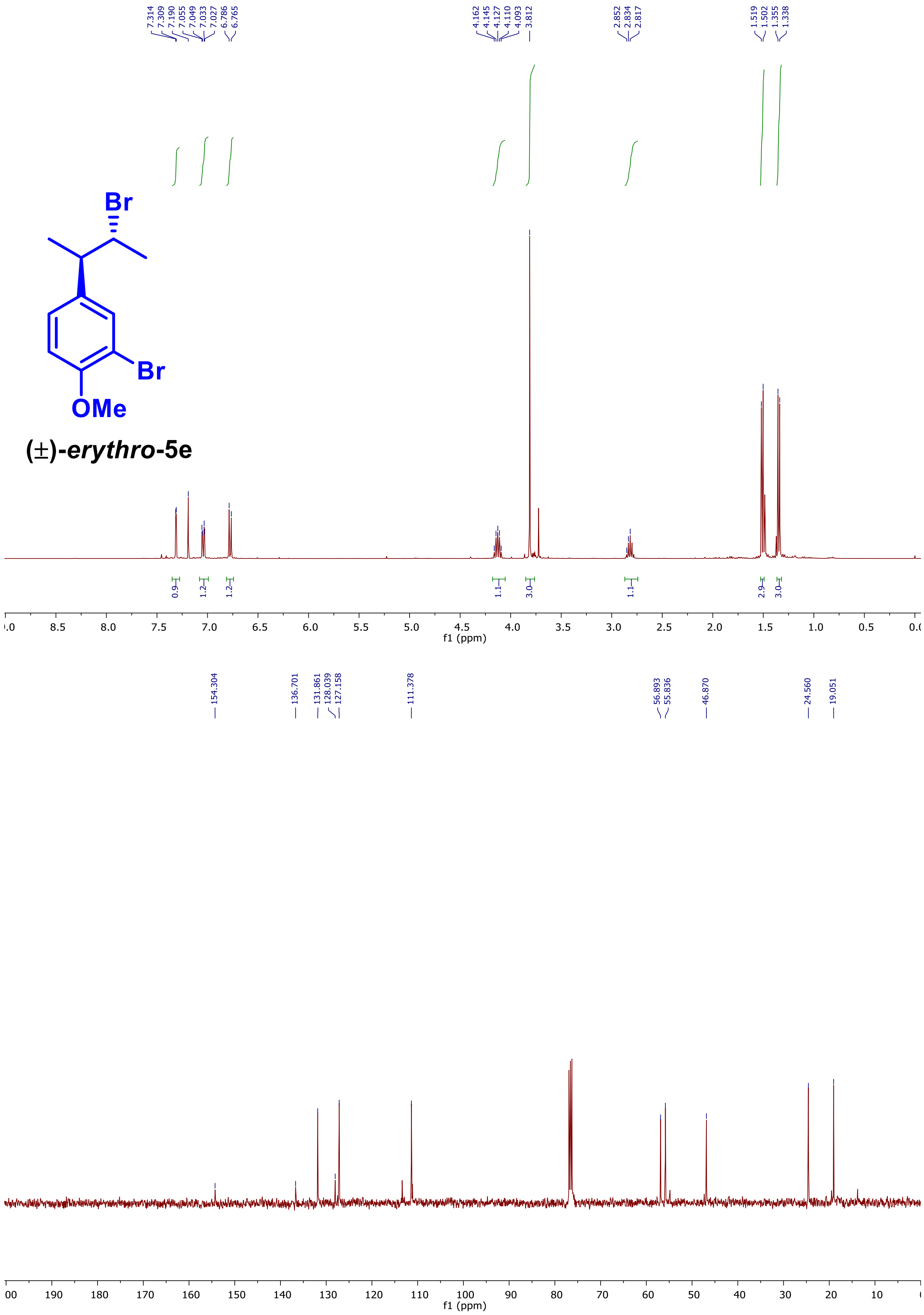

Figure S41. NMR spectra of (±)-*erythro*-3-(3-bromo-4-methoxyphenyl)-2-bromobutane, (±)-*erythro*-**5e** in $CDCl_3$. *Top*: ^1H NMR (400 MHz), *Bottom:* ^{13}C NMR (100 MHz).

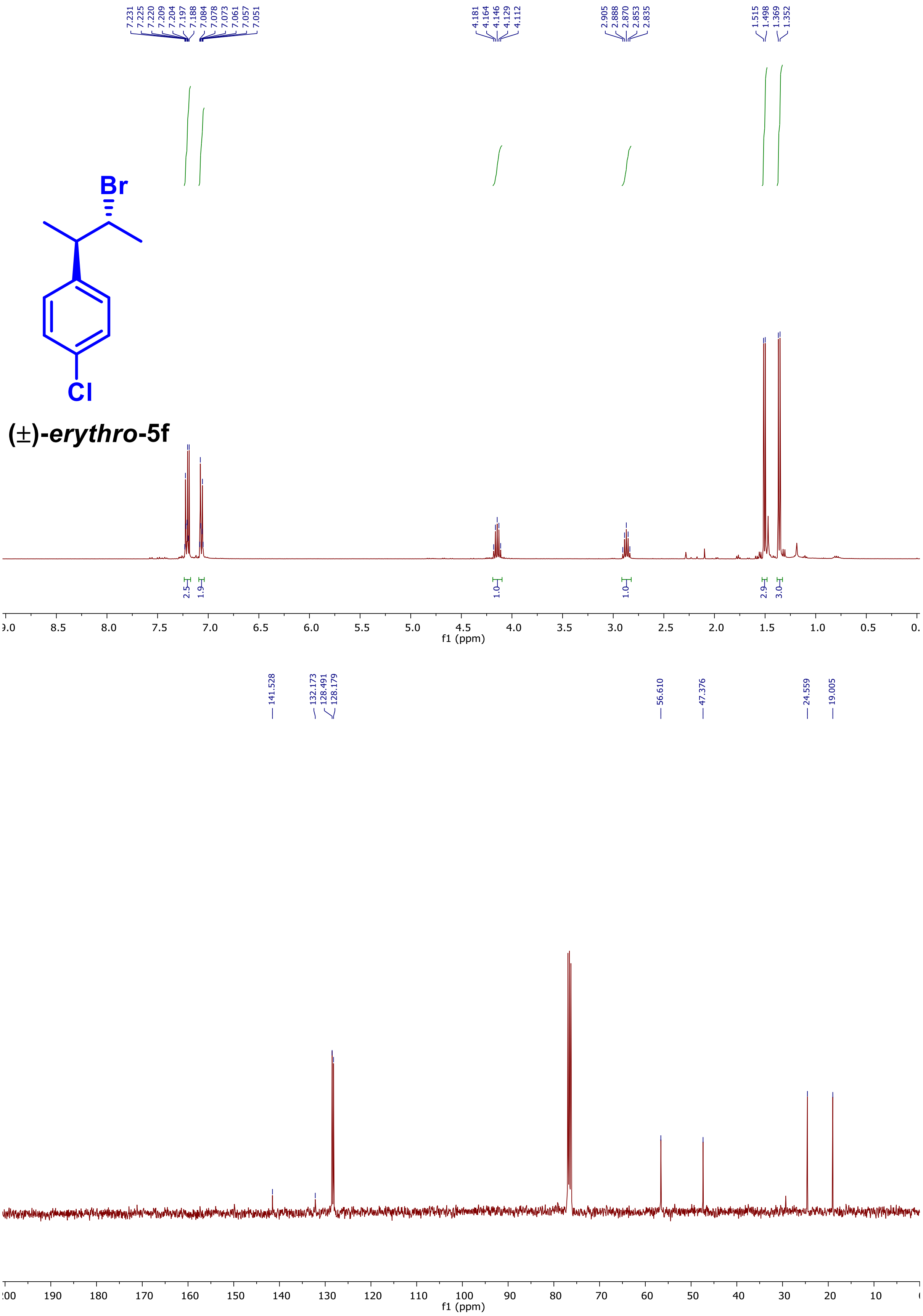

Figure S42. NMR spectra of (±)-*erythro*-3-(4-chlorophenyl)-2-bromobutane, (±)-*erythro*-**5f** in $CDCl_3$. *Top*: ^{1}H NMR (400 MHz), *Bottom:* ^{13}C NMR (100 MHz).

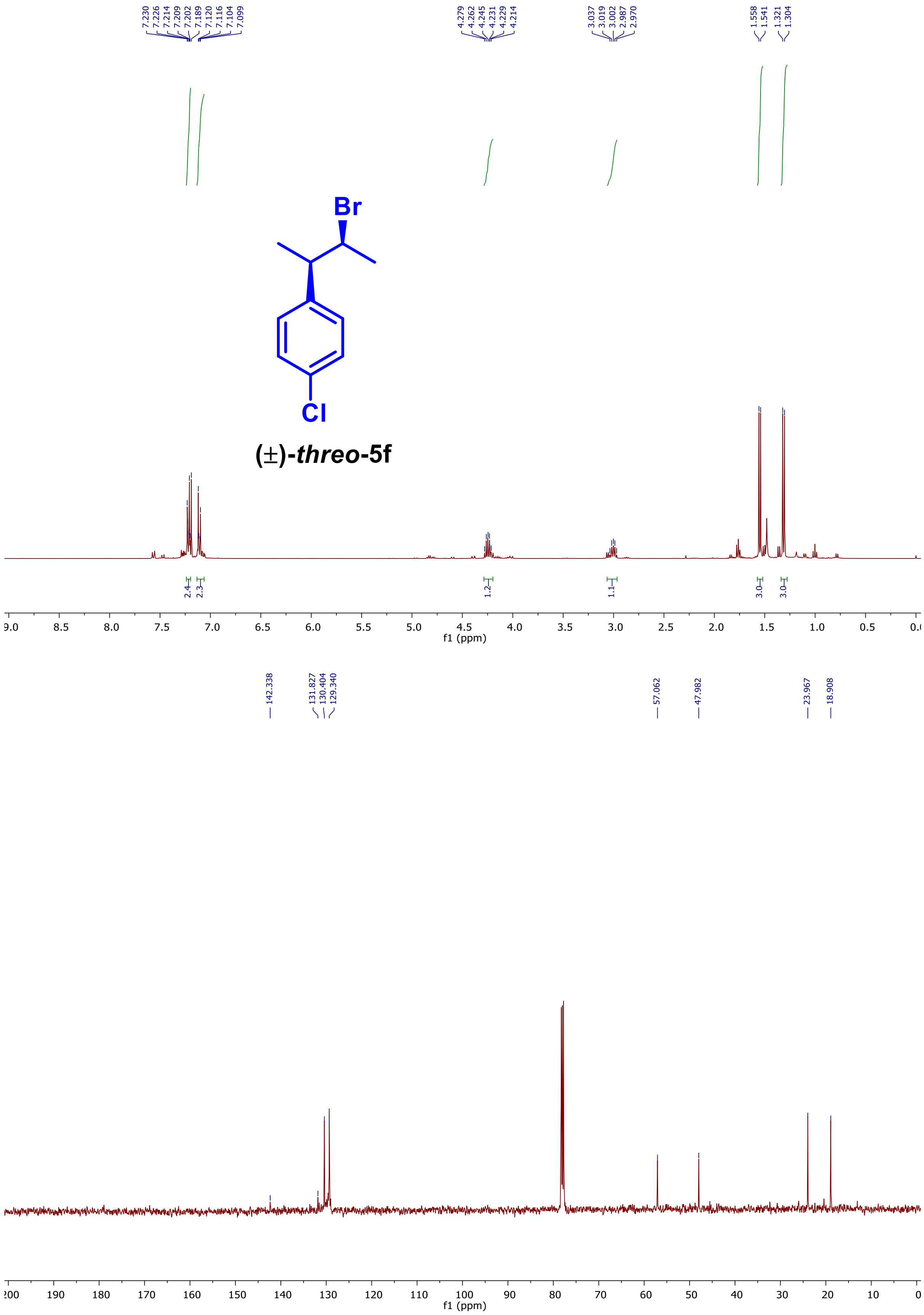

Figure S43. NMR spectra of (±)-*threo*-3-(4-chlorophenyl)-2-bromobutane, (±)-*threo*-**5f** in $CDCl_3$. *Top*: ^{1}H NMR (400 MHz), *Bottom:* ^{13}C NMR (100 MHz).

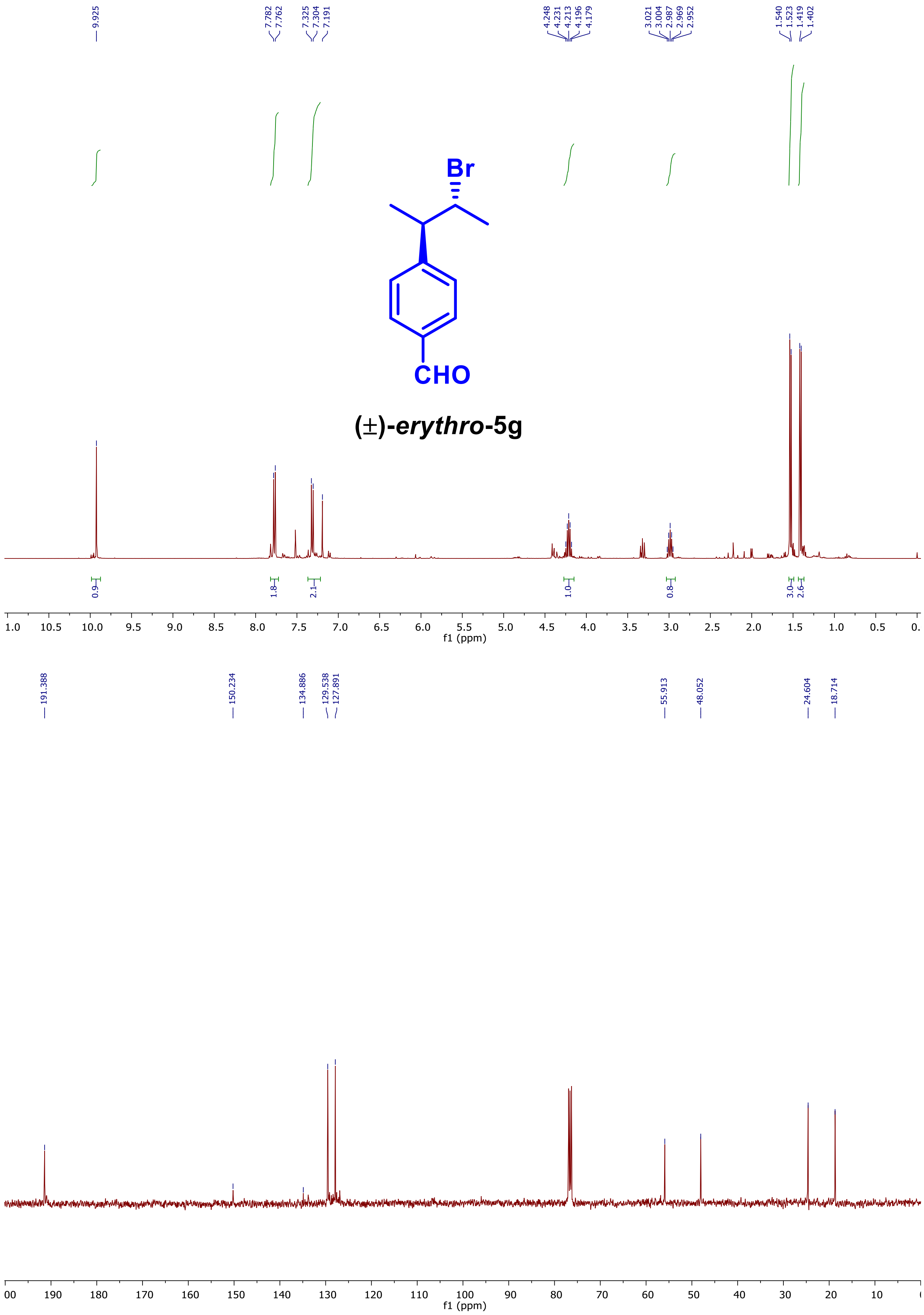

Figure S44. NMR spectra inof (±)-*erythro*-3-(4-(carbaldehyde)phenyl)-2-bromobutane, (±)-*erythro*-**5g** in $CDCl_3$. *Top*: ^{1}H NMR (400 MHz), *Bottom:* ^{13}C NMR (100 MHz).

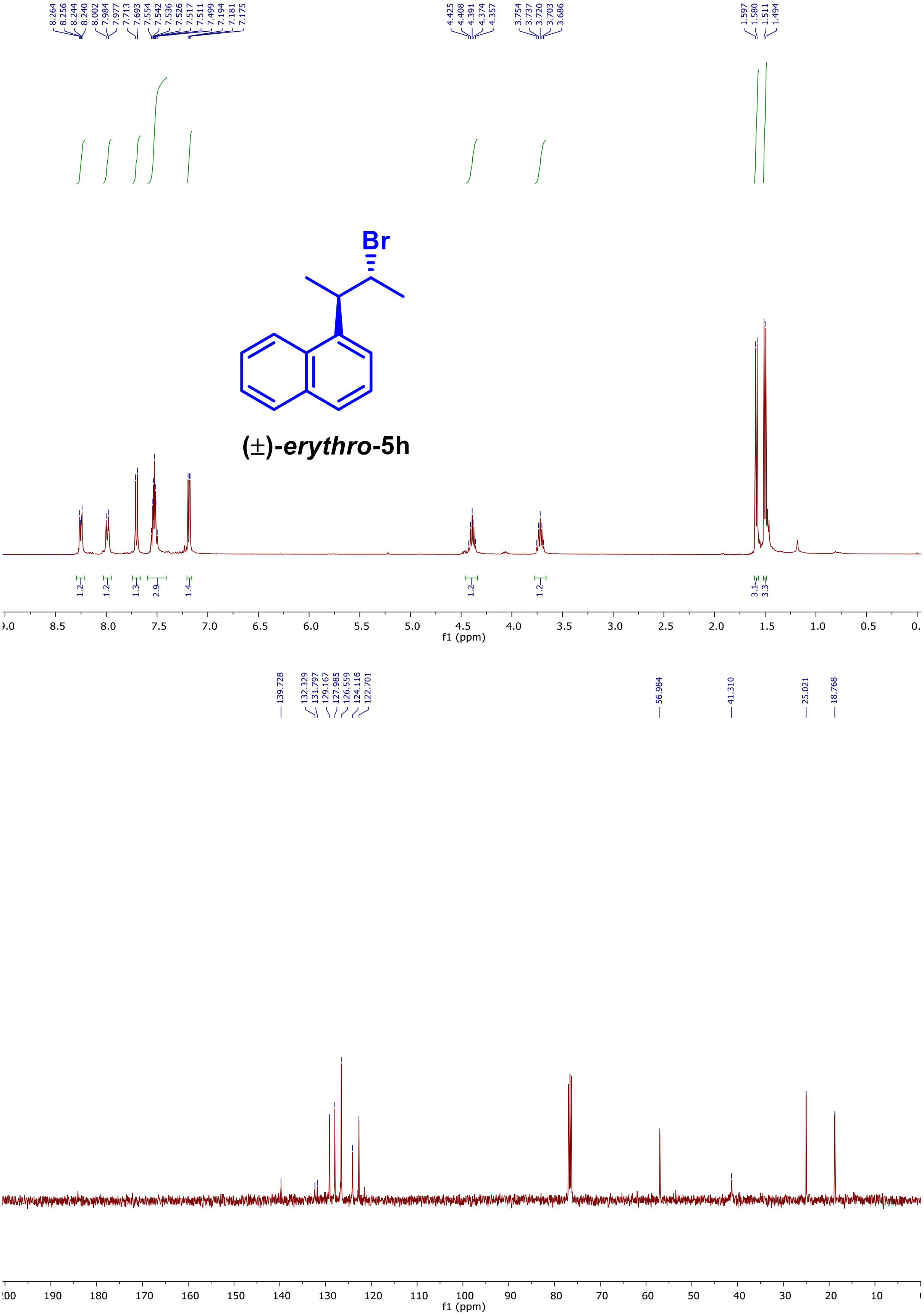

Figure S45. NMR spectra of (±)-*erythro*-3-(1-naphthyl)-2-bromobutane, (±)-*erythro*-**5h** in $CDCl_3$. *Top*: ^1H NMR (400 MHz), *Bottom:* ^{13}C NMR (100 MHz).

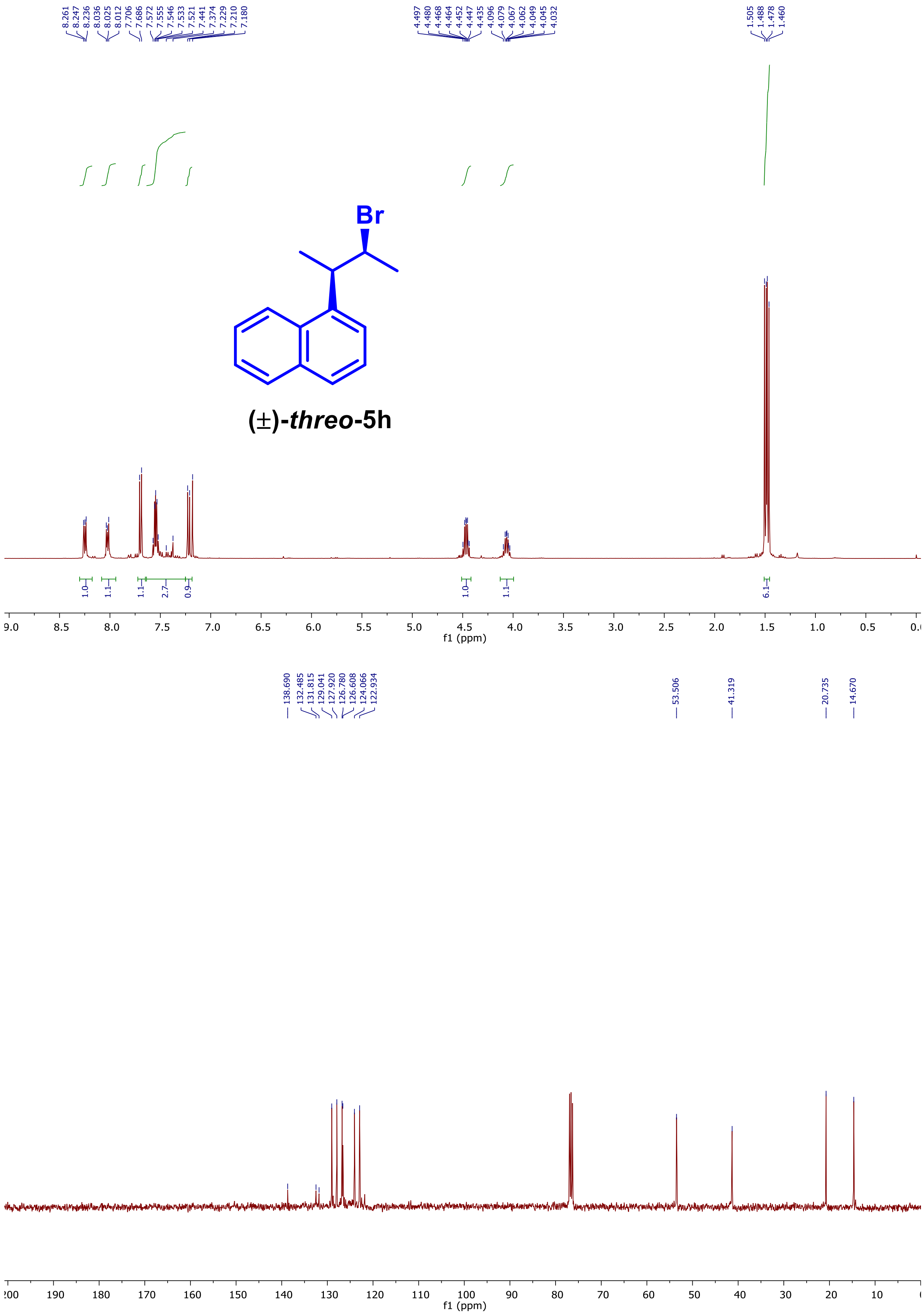

Figure S46. NMR spectra of (±)-*threo*-3-(1-naphthyl)-2-bromobutane, (±)-*threo* **-5h** in $CDCl_3$. *Top*: ^{1}H NMR (400 MHz), *Bottom:* ^{13}C NMR (100 MHz).

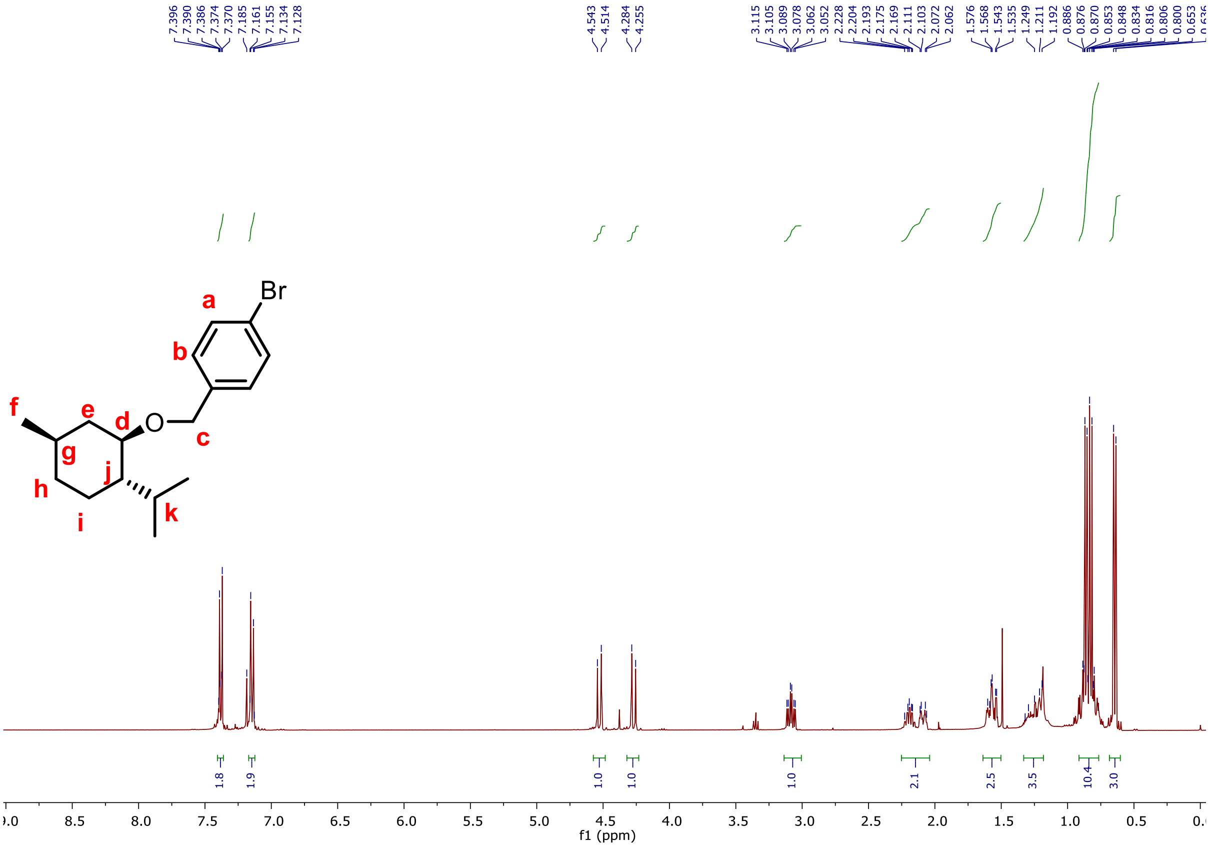

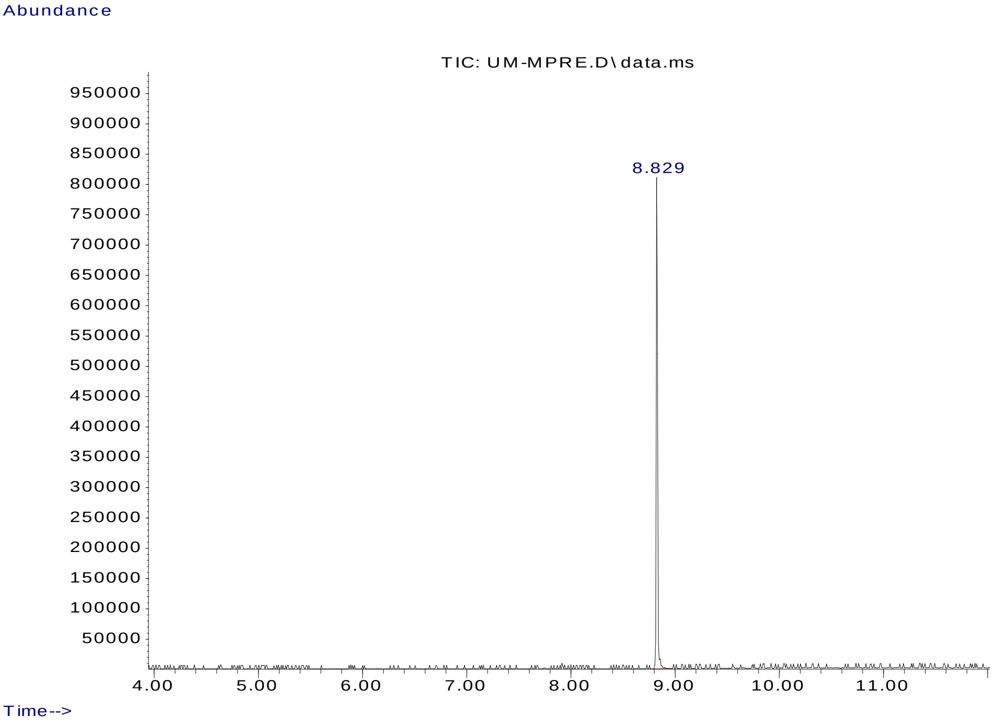

Figure S47. *Top:* ^{1}H NMR spectra of (±)-*threo*-4-bromobenzyl (-)-menthyl ether, **5i** in $CDCl_3$. *Bottom:* GC-MS chromatogram confirming a single diastereomer product.

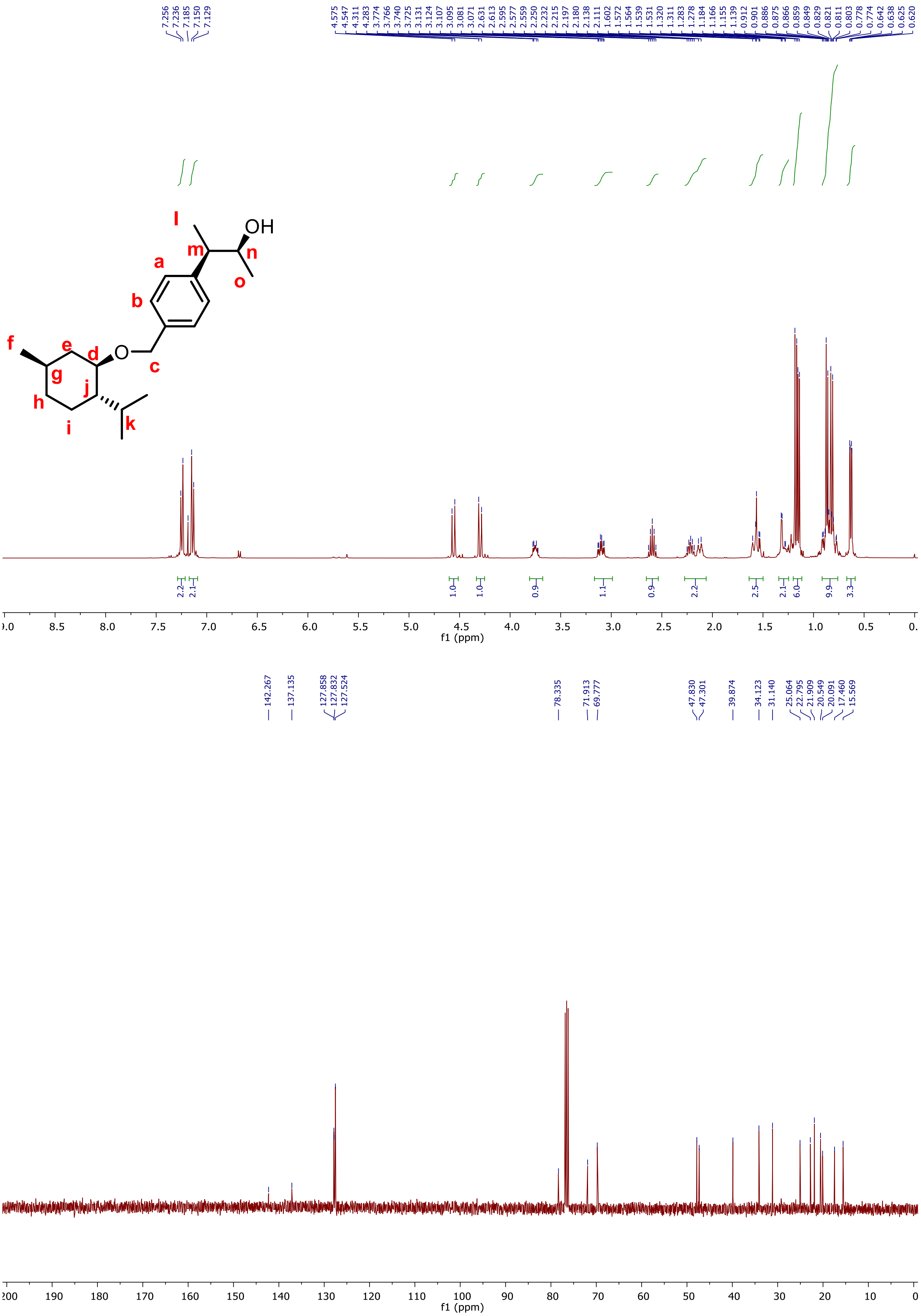

Figure S48. NMR spectra of (±)-*threo*-**2i** in $CDCl_3$. *Top*: ^{1}H NMR (400 MHz), *Bottom:* ^{13}C NMR (100 MHz).

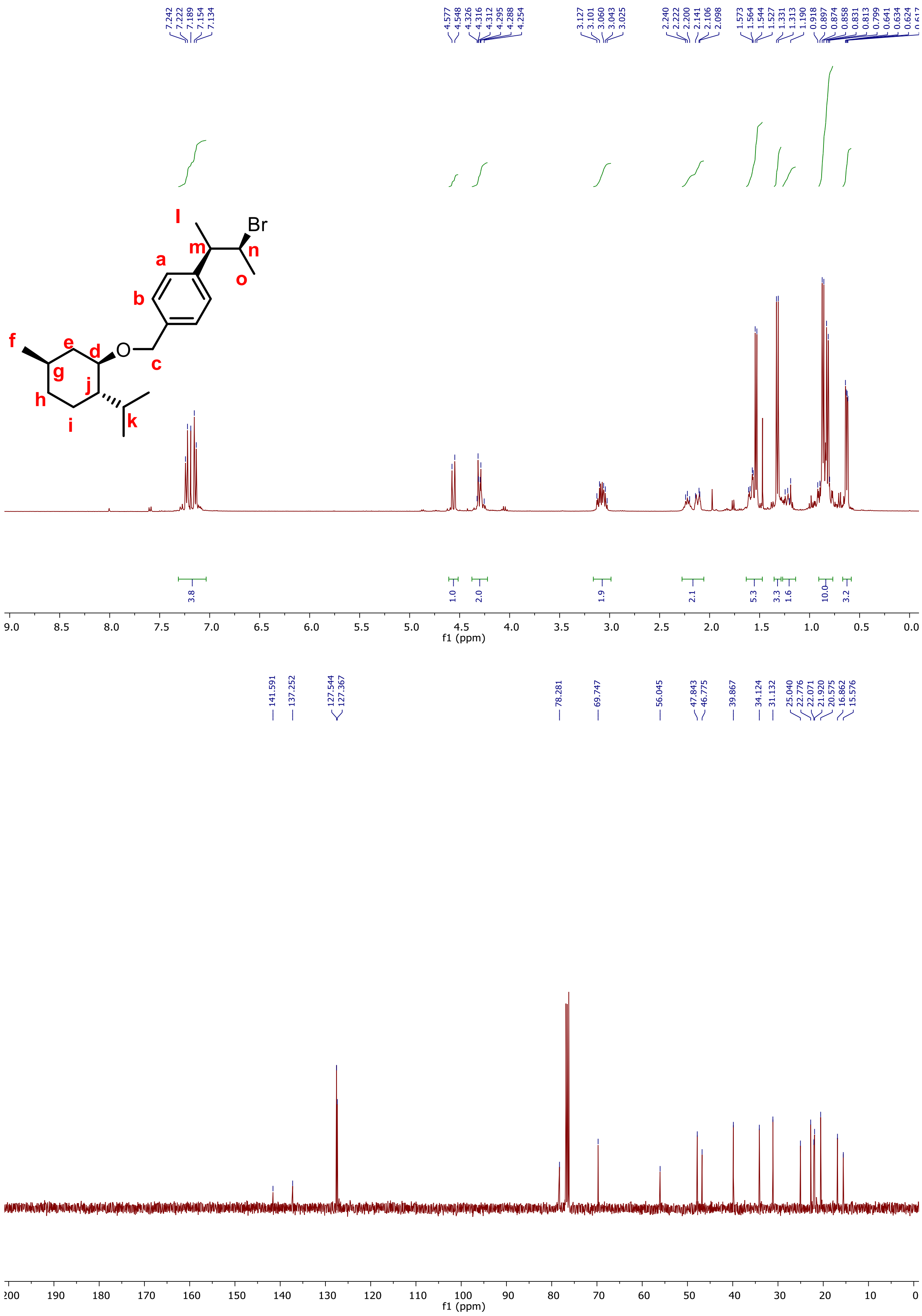

Figure S49. NMR spectra of (±)-*threo*-**5i** in CDCl$_3$. *Top*: ^{1}H NMR (400 MHz), *Bottom:* ^{13}C NMR (100 MHz).

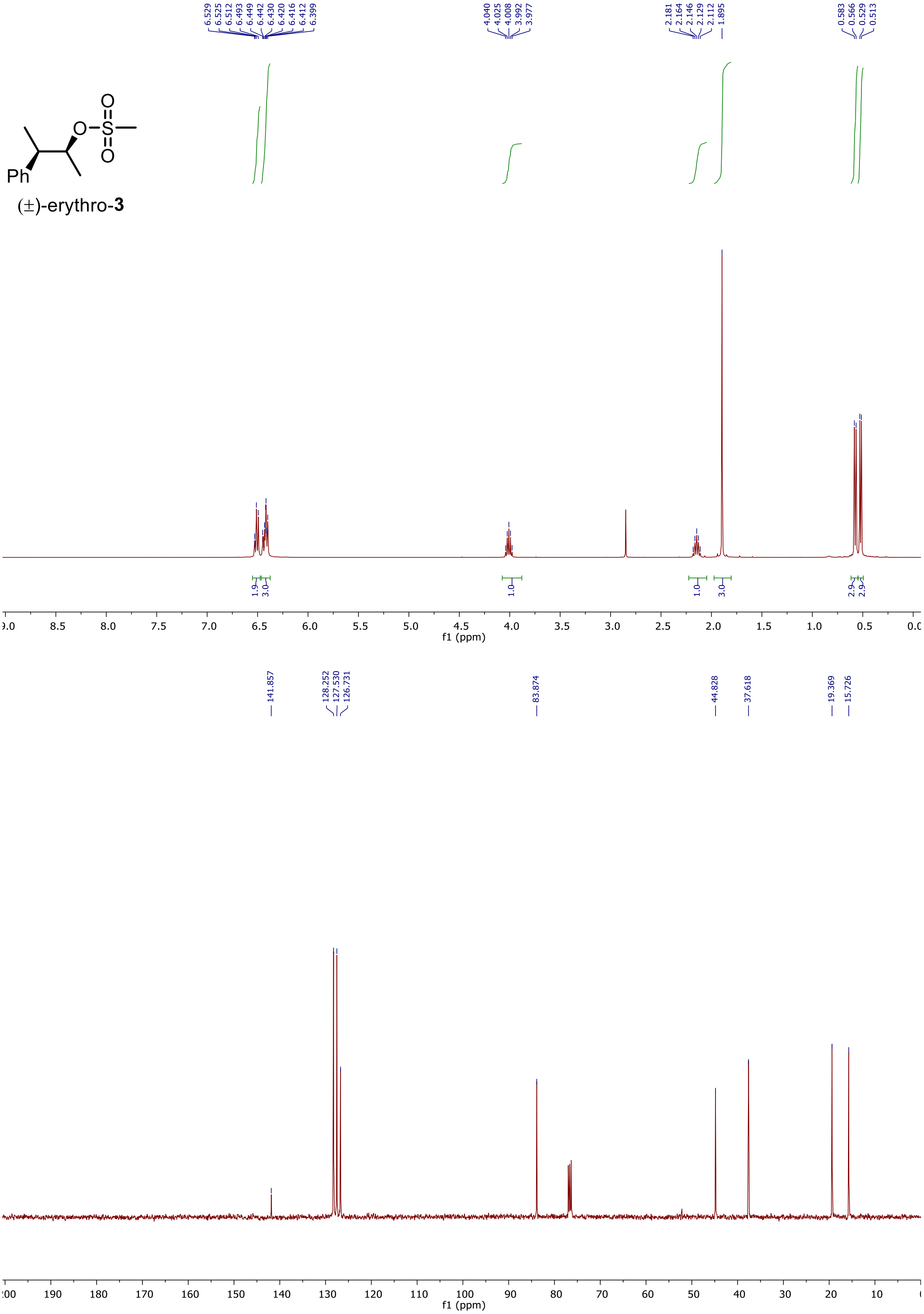

Figure S50. NMR spectra of (±)-*erythro* -3-phenyl-2-butyl methanesulfonate, (±)-*erythro*-**3** in $CDCl_3$. *Top*: ^{1}H NMR (400 MHz), *Bottom:* ^{13}C NMR (100 MHz).

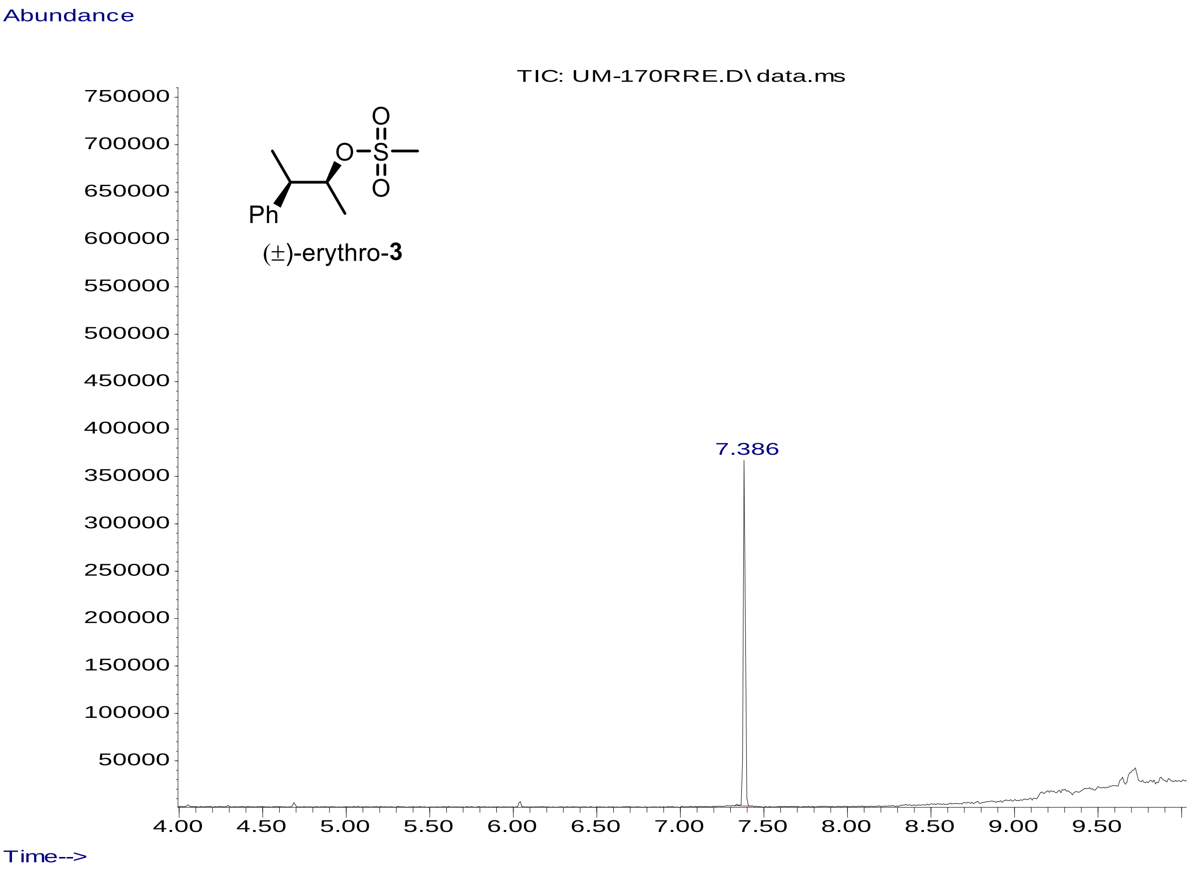

Figure S51. GC-MS of (±)-*erythro*-3-phenyl-2-butyl methanesulfonate, (±)-*erythro*-**3**.

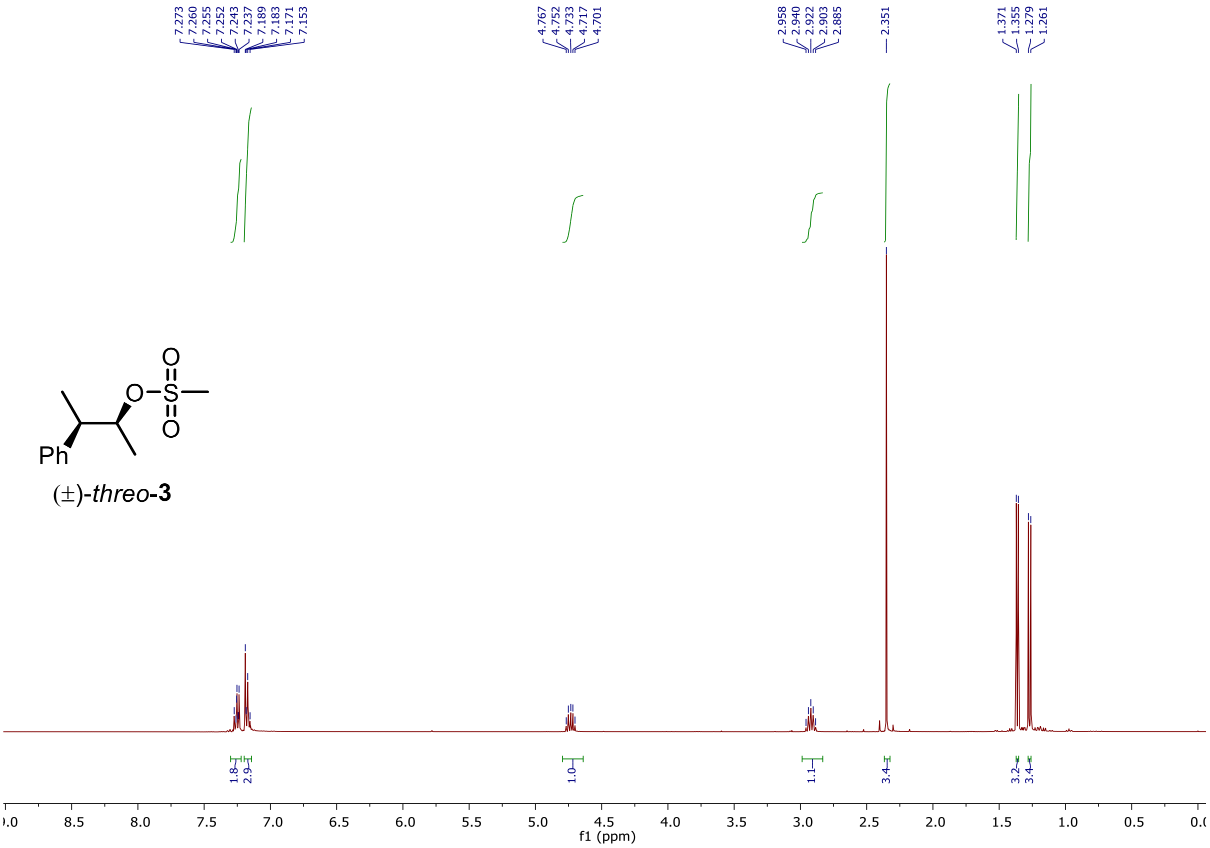

Figure S52. ^{1}H NMR (400 MHz) spectrum of (±)-*threo*-3-phenyl-2-butyl methanesulfonate, (±)-*threo*-**3** in $CDCl_3$

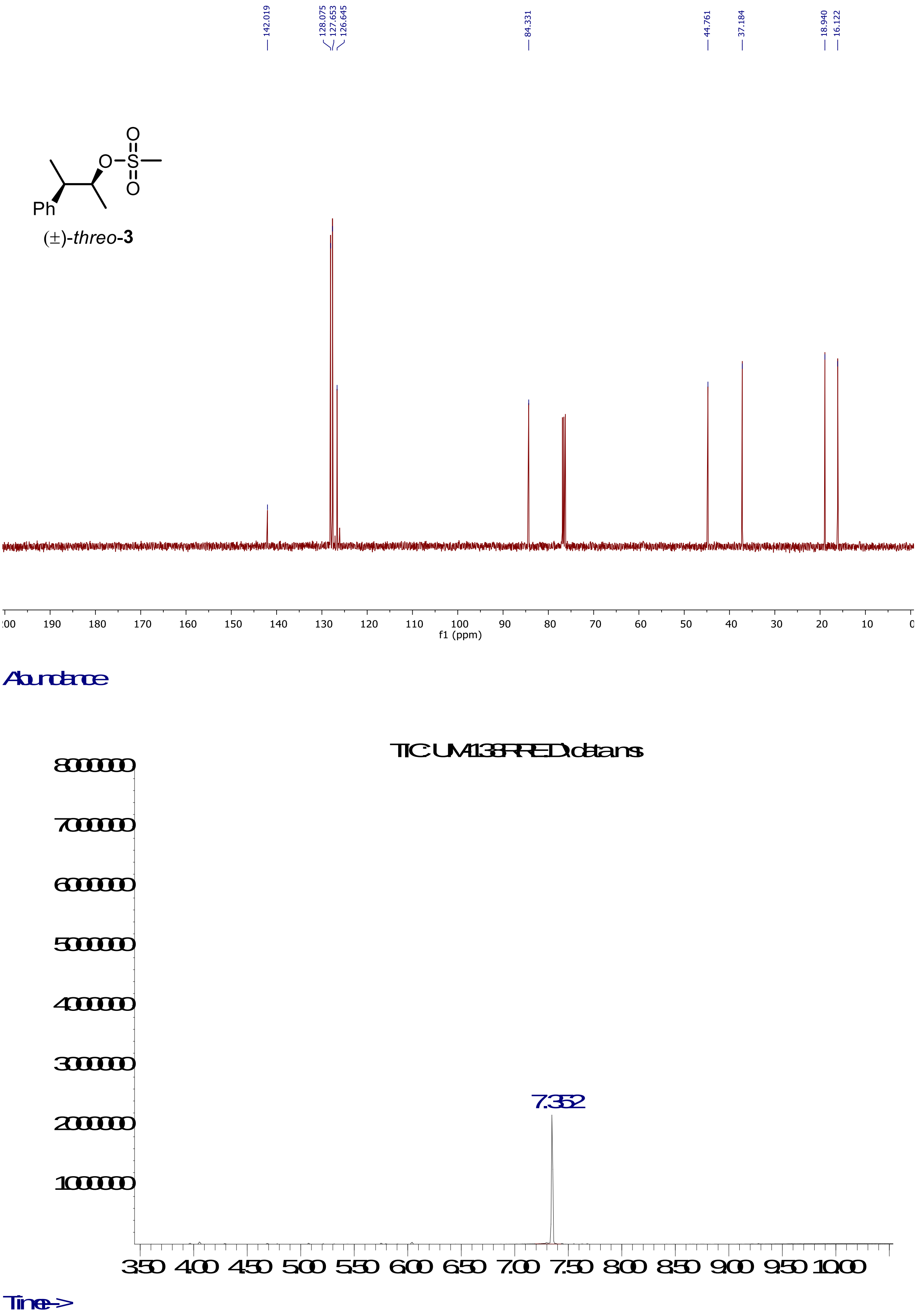

Figure S53. ^{13}C NMR (100 MHz) spectrum of (±)-*threo*-3-phenyl-2-butyl methanesulfonate, (±)-*threo*-**3** in $CDCl_3$ (*top)* and GC-MS chromatogram (*bottom*).

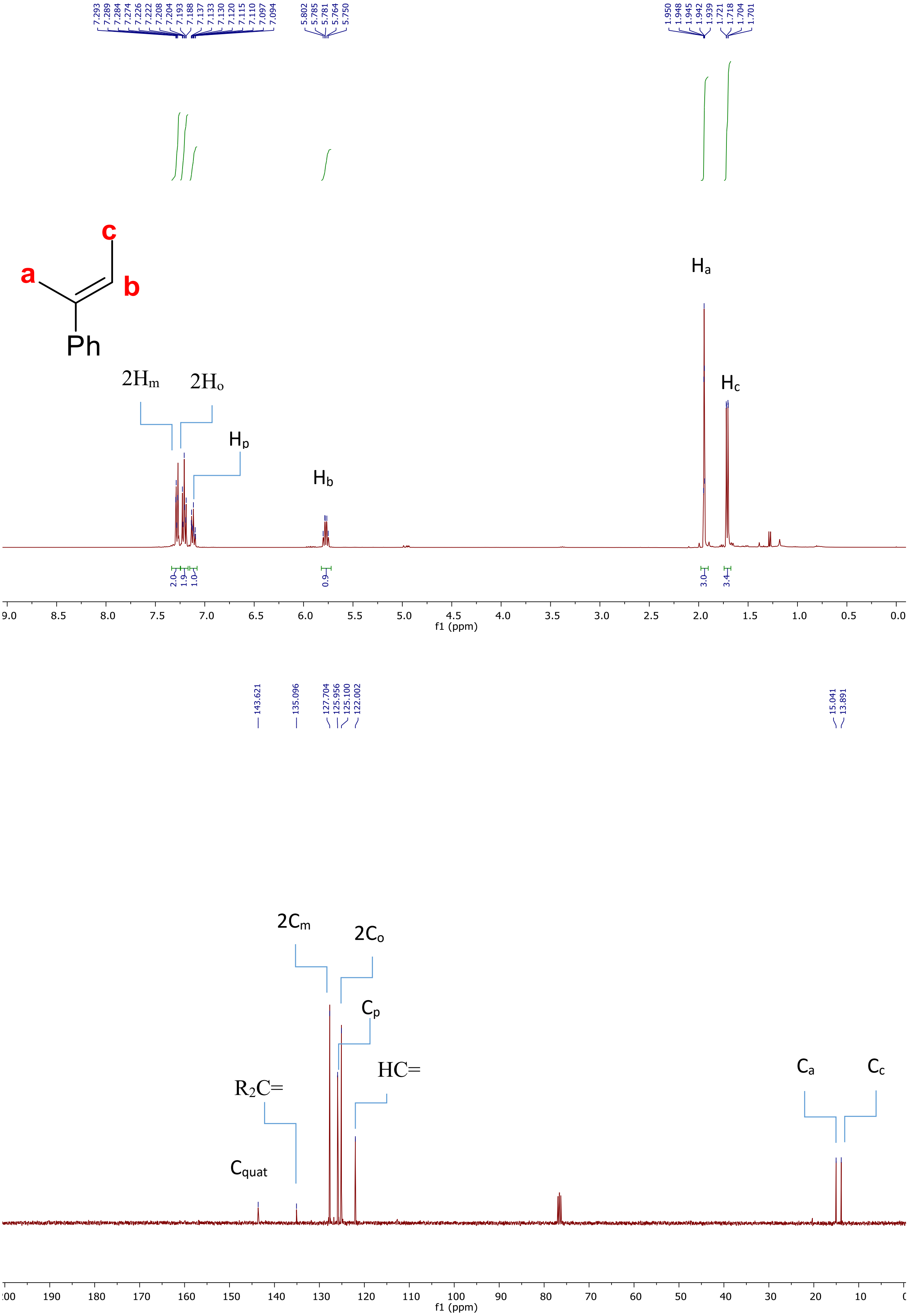

Figure S54. NMR spectra of (*E*)-2-phenyl-2-butene, (*E*)-**4** in $CDCl_3$. *Top*: ^{1}H NMR (400 MHz), *Bottom:* ^{13}C NMR (100 MHz).

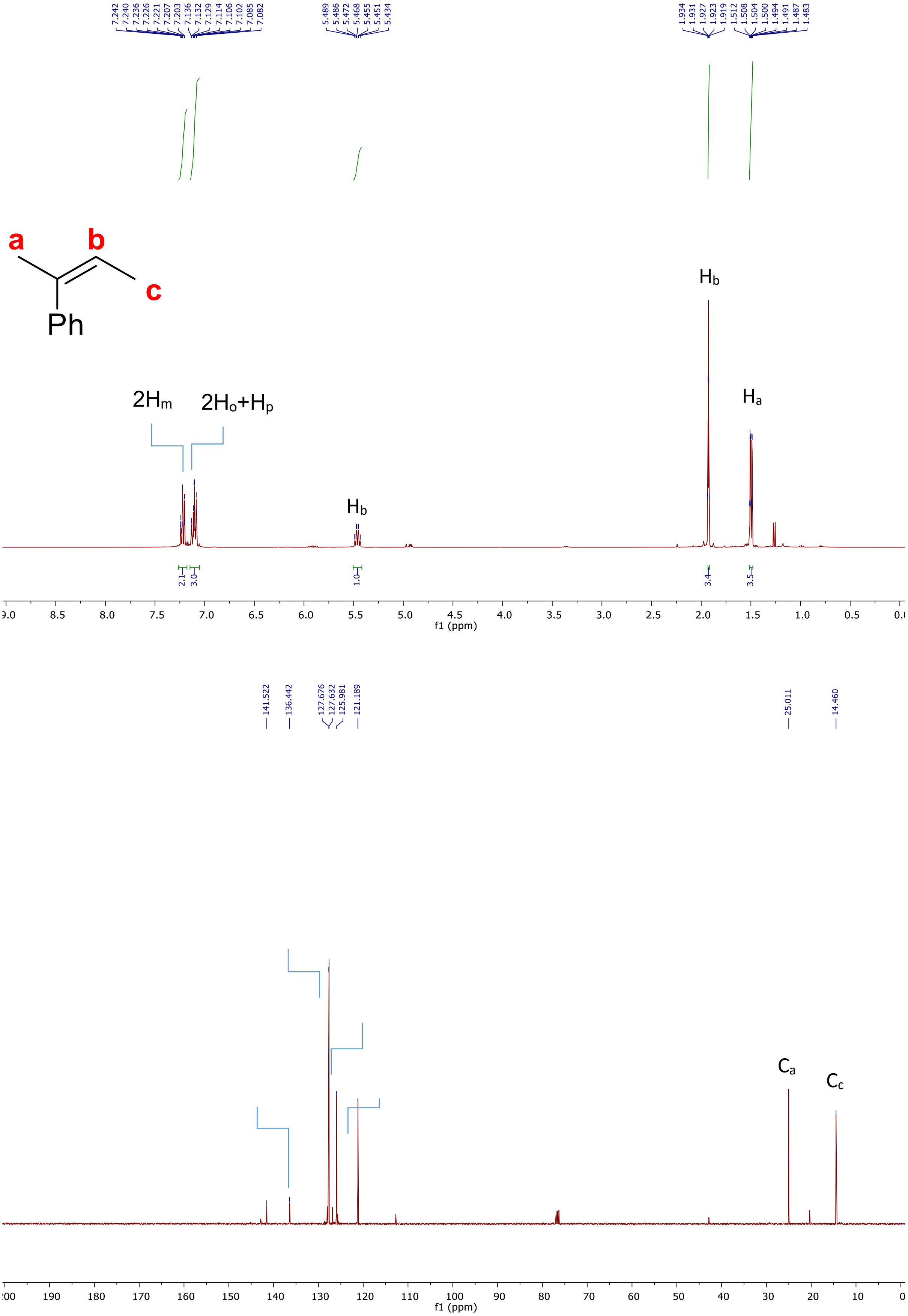

Figure S55. NMR spectra of (*Z*)-2-phenyl-2-butene, (*Z*)-**4** in $CDCl_3$. *Top*: ^{1}H NMR (400 MHz), *Bottom:* ^{13}C NMR (100 MHz).

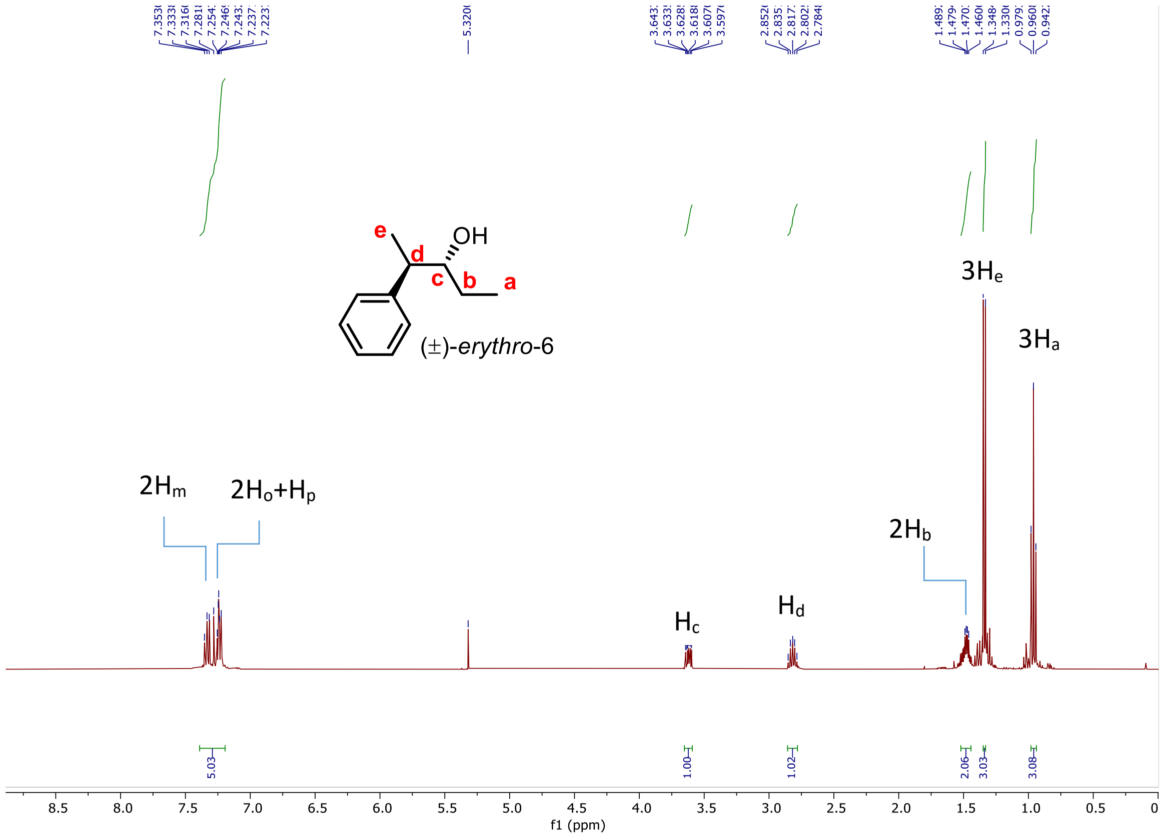

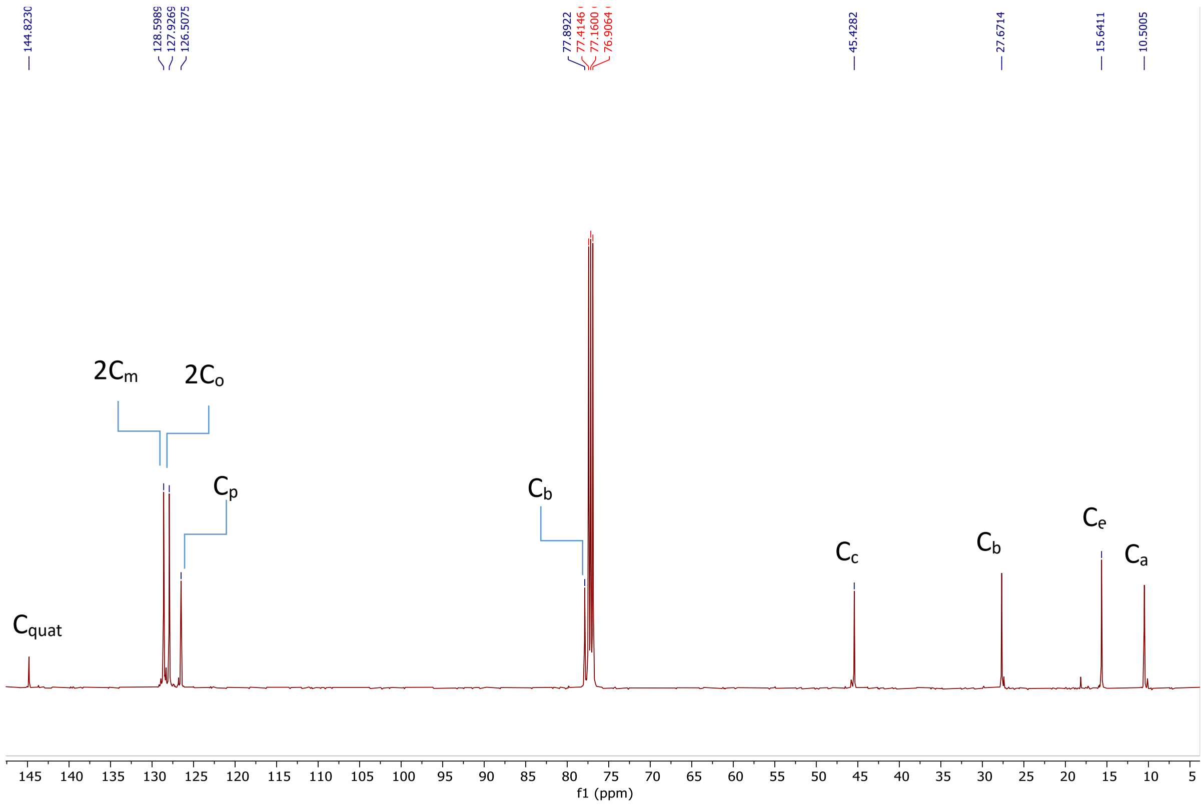

Figure S56. NMR spectra of (±)-*erythro*-2-phenyl-3-pentanol, (±)-*erythro*-**6** in $CDCl_3$. *Top*: ^{1}H NMR (400 MHz), *Bottom:* ^{13}C NMR (100 MHz).

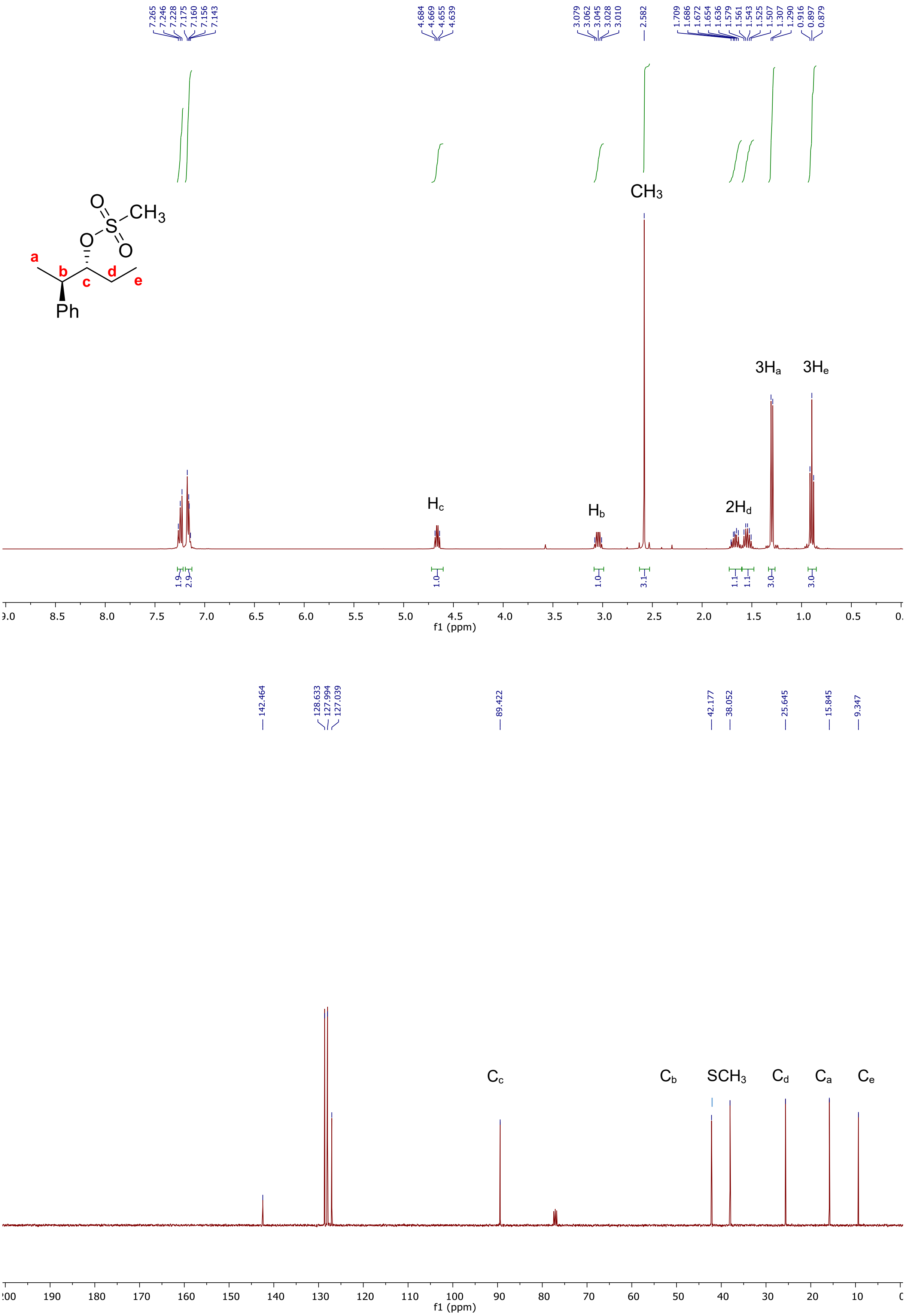

Figure S57. NMR spectra of (±)-*erythro*-2-phenyl-3-pentanyl methane sulfonate, (±)-*erythro*-mesylated-**6** in $CDCl_3$. *Top*: ^{1}H NMR (400 MHz), *Bottom:* ^{13}C NMR (100 MHz).

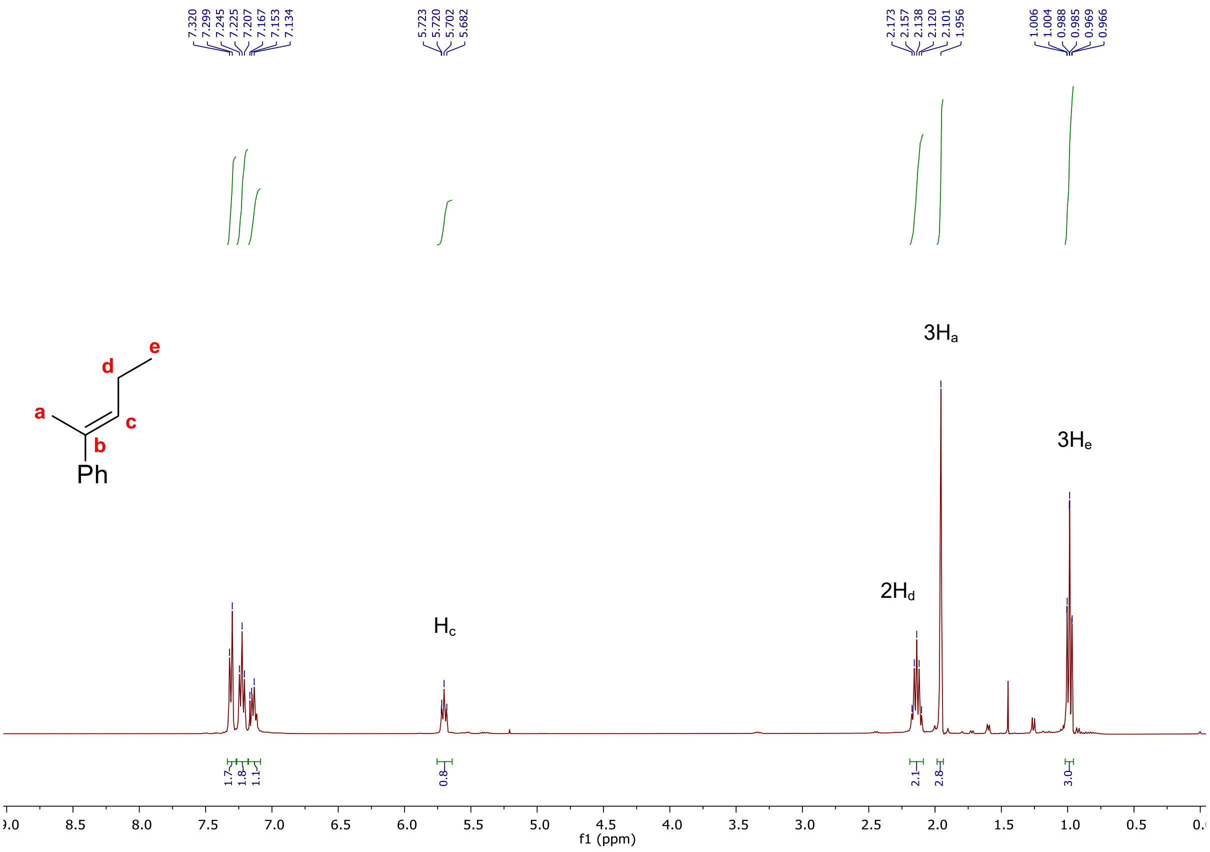

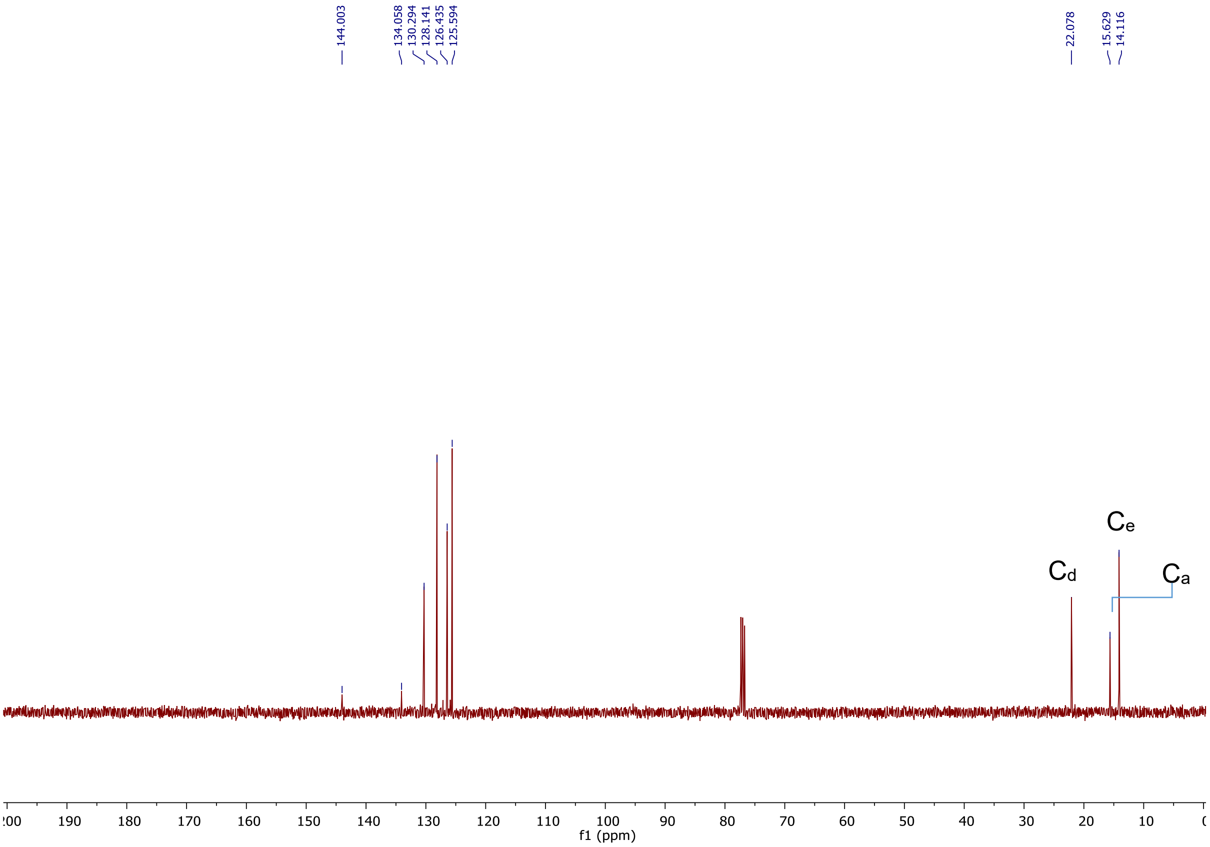

Figure S58. NMR spectra of (*E*)-2-phenyl-2-pentene, (*E*)-**9**, in $CDCl_3$. *Top*: ^{1}H NMR (400 MHz), *Bottom:* ^{13}C NMR (100 MHz).

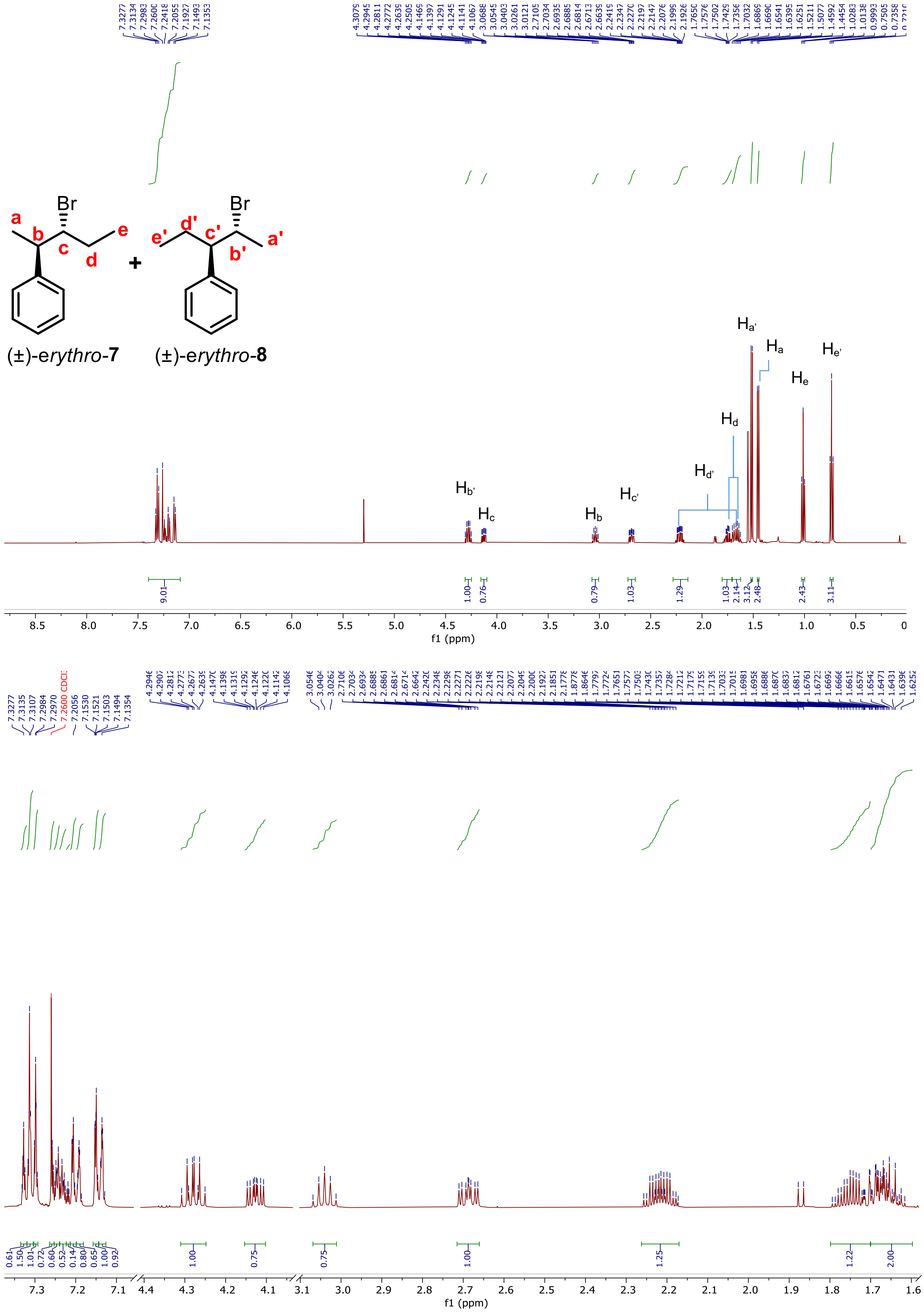

Figure S59. NMR spectra of (±)-*erythro*-2-phenyl-3-bromopentane, (±)-*erythro*-**7**, and (±)-*erythro*-3-phenyl-2-bromopentane, (±)-*erythro*-**8** in $CDCl_3$. *Top*: ^1H NMR (400 MHz), *Bottom:* Expansion of the NMR spectrum at selected chemical shift ranges.

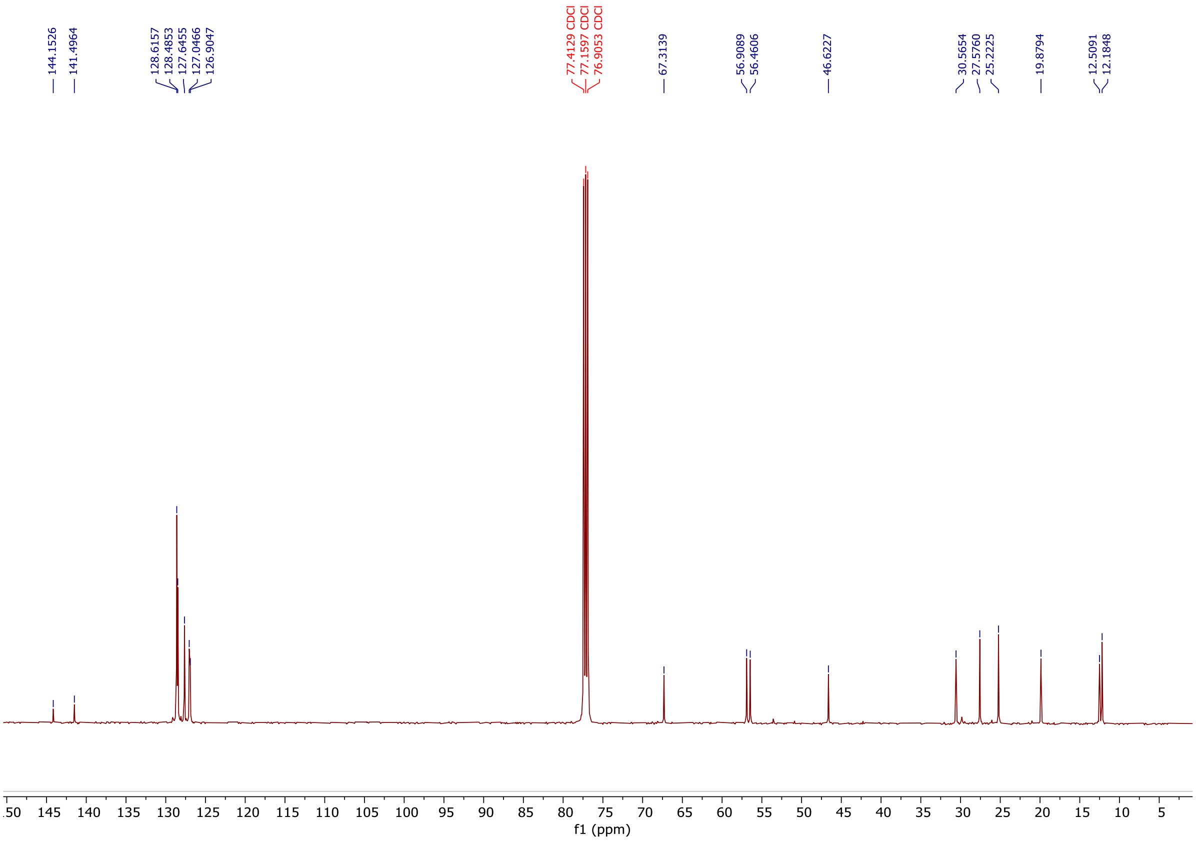

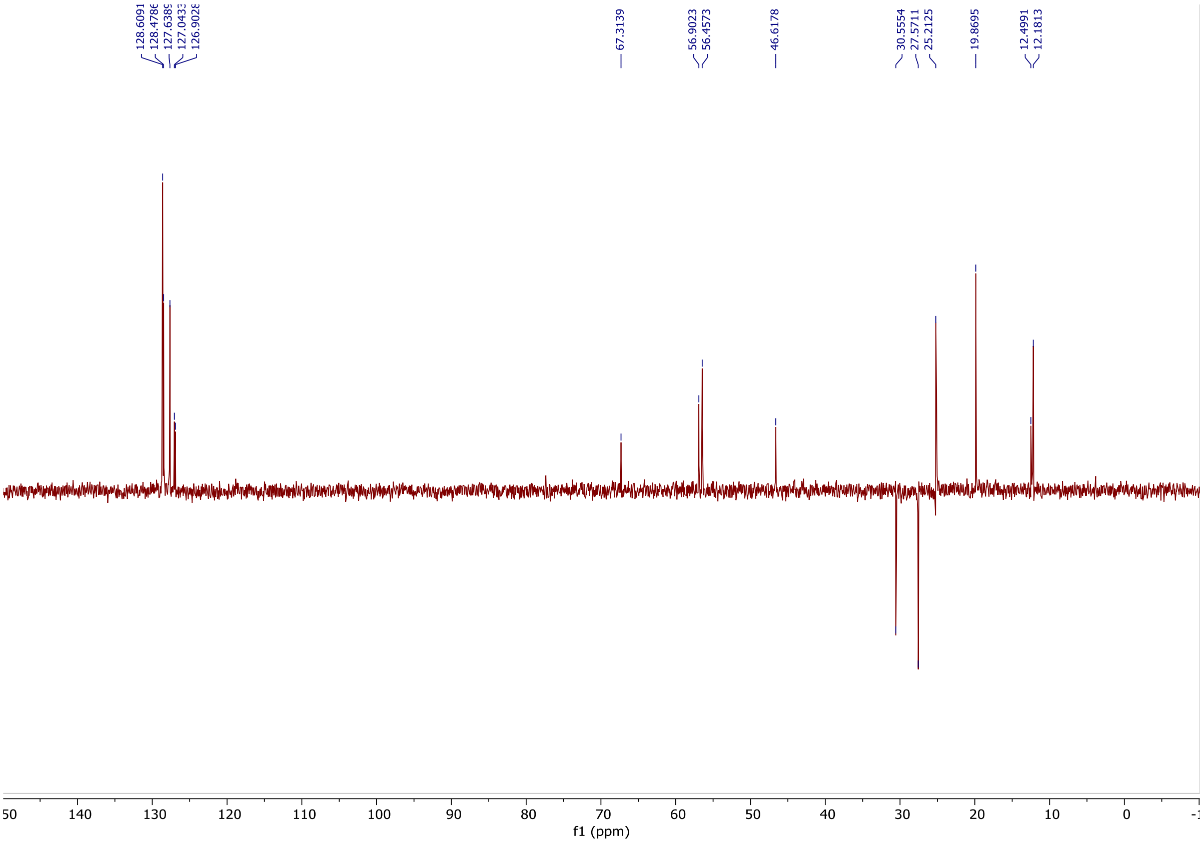

Figure S60. NMR spectra of (±)-*erythro*-2-phenyl-3-bromopentane, (±)-*erythro*-**7**, and (±)-*erythro*-3-phenyl-2-bromopentane, (±)-*erythro*-**8** in $CDCl_3$. *Top*: ^{13}C NMR (100 MHz), *Bottom:* DEPT-135 NMR experiment.

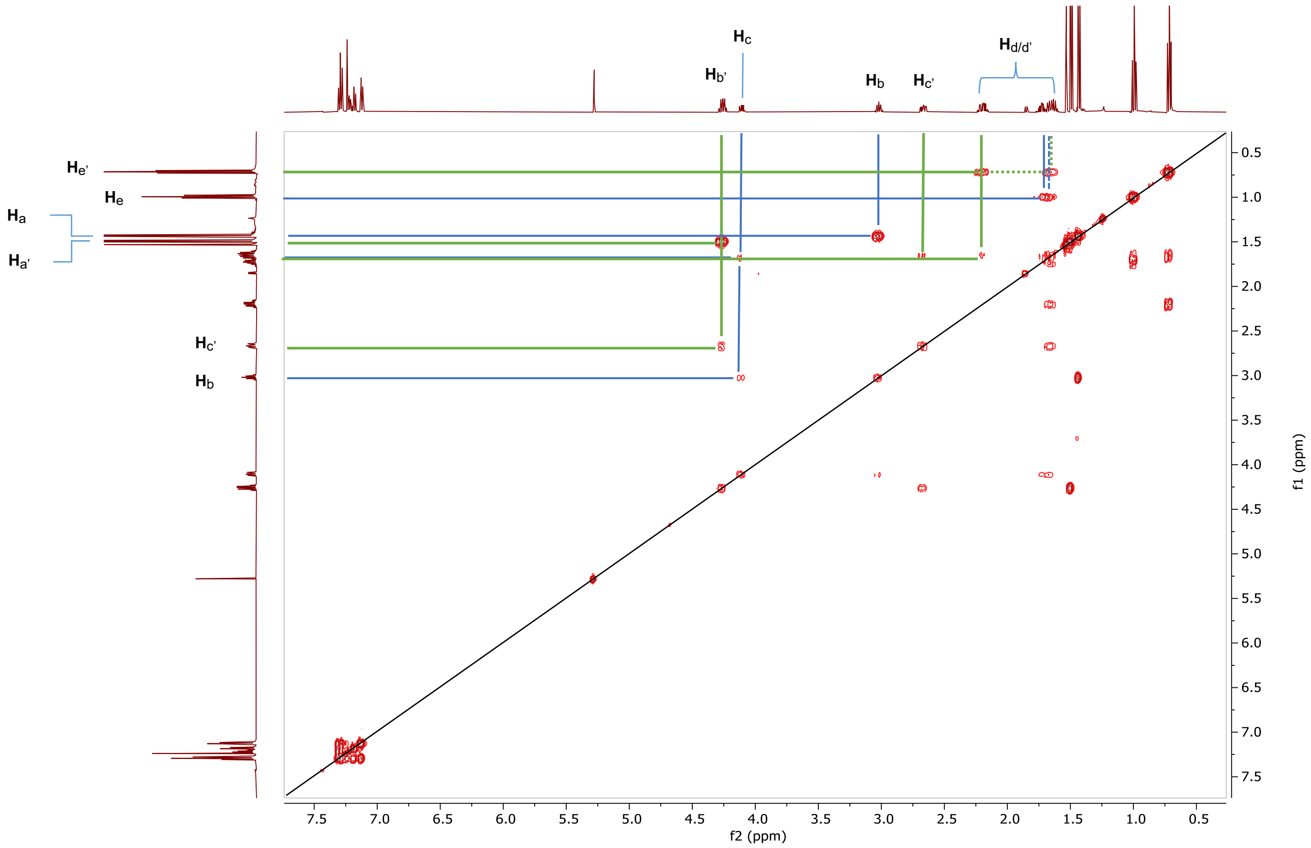

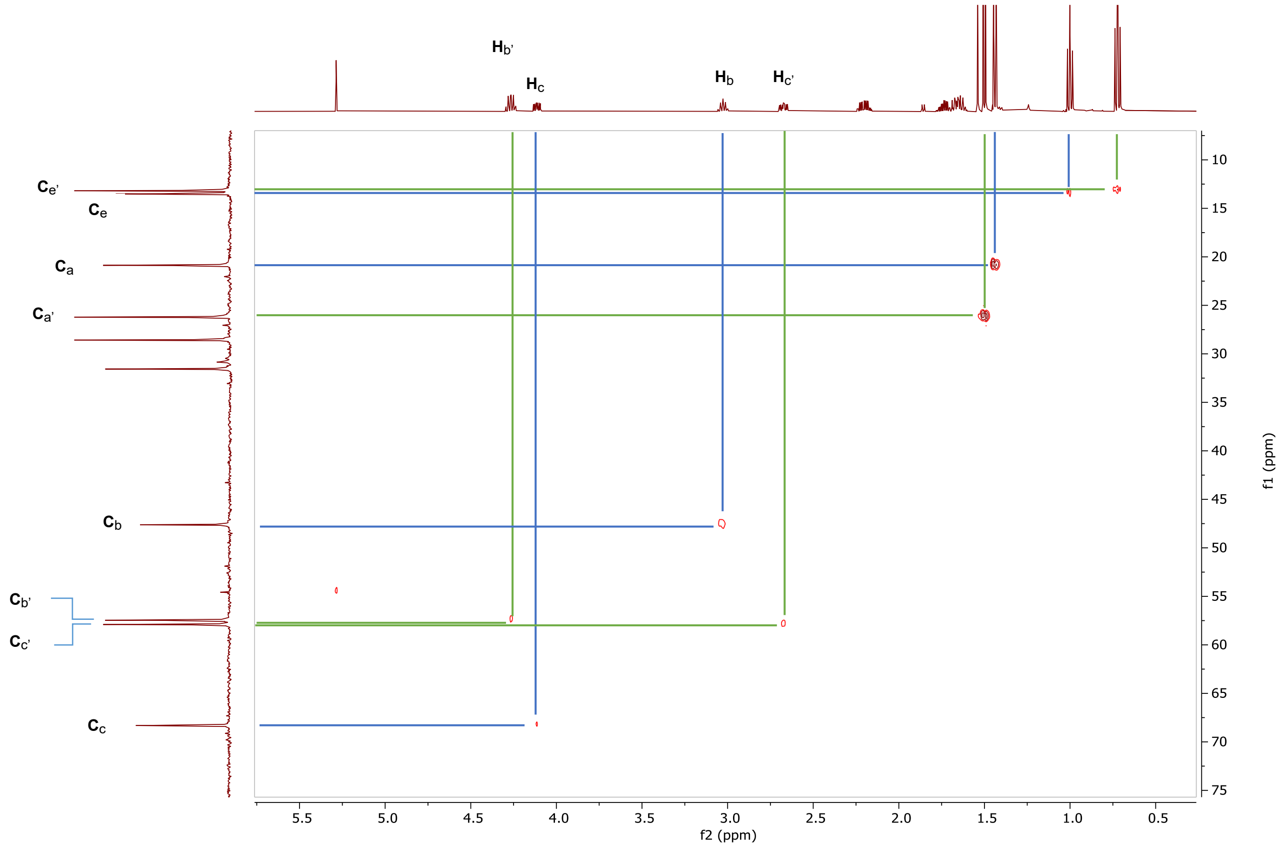

Figure S61. NMR spectra of (±)-*erythro*-2-phenyl-3-bromopentane, (±)-*erythro*-**7**, and (±)-*erythro*-3-phenyl-2-bromopentane, (±)-*erythro*-**8** in $CDCl_3$. *Top*: COSY 2D-NMR (400 MHz), *Bottom:* HMQC 2D-NMR.

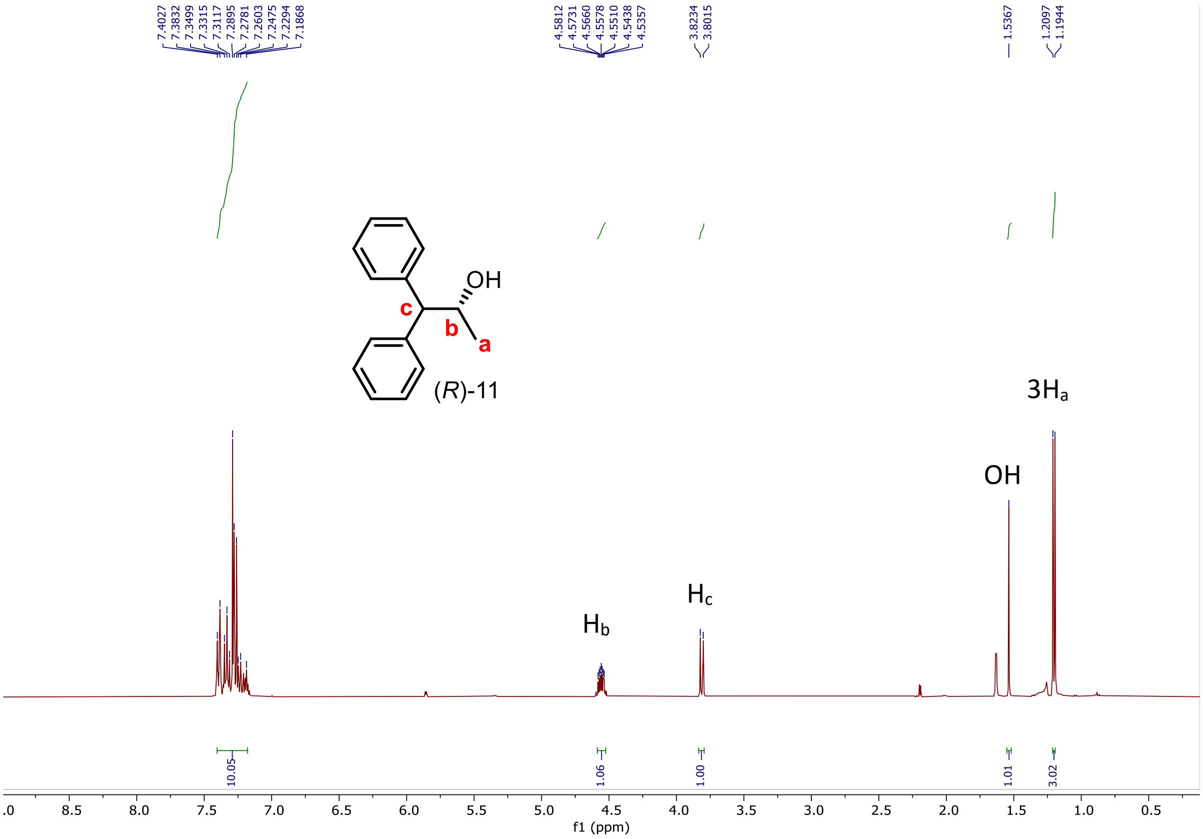

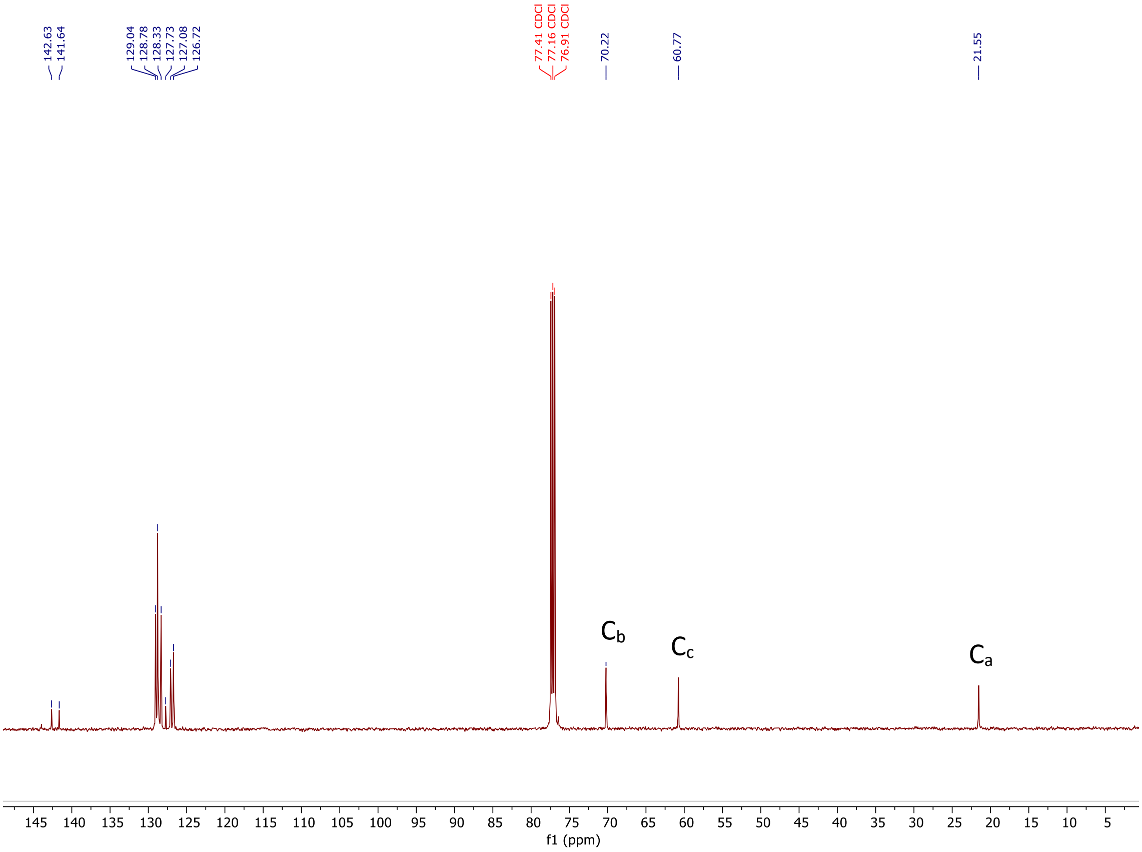

Figure S62. NMR spectra of (*R*)-1,1-diphenyl-2-propanol, (*R*)-**11** in $CDCl_3$. *Top*: ^{1}H NMR (400 MHz), *Bottom:* ^{13}C NMR (100 MHz).

(R)-11

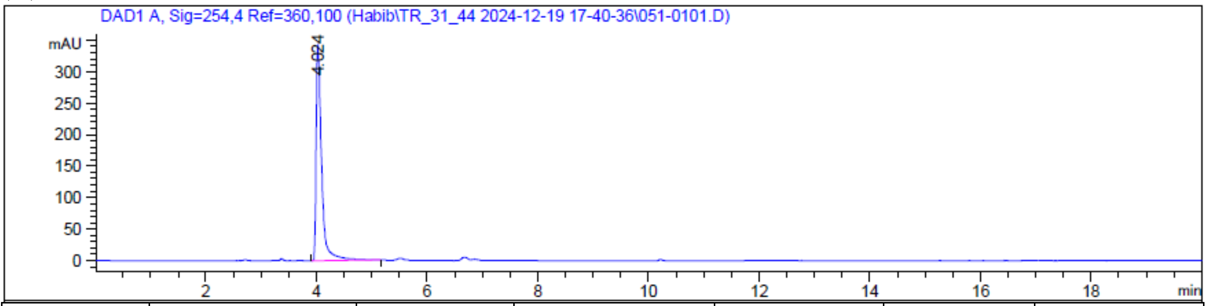

Peak (#)	Retention time (min)	Integration notation	Width (mAU*s)	Area (mAU)	Hight	Area (%)
**1**	4.024	BB	0.1037	2394.503	342.133	100

Erythro- and *threo*-**13**

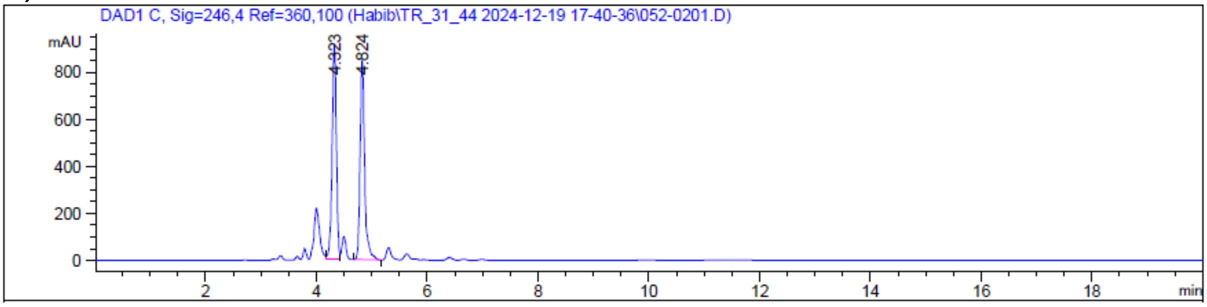

Peak (#)	Retention time (min)	Integration notation	Width (mAU*s)	Area (mAU)	Hight	Area (%)
**1**	4.323	VV	0.0827	4929.068	917.766	50.27
**2**	4.824	BB	0.0867	4874.485	852.801	49.73

Figure S63. Stereoselective bromination of (R)-**11**. *Top*: HPLC chromatograph of enantiopure substrate. *Bottom*: HPLC chromatographs of diastereomeric products of **13**.

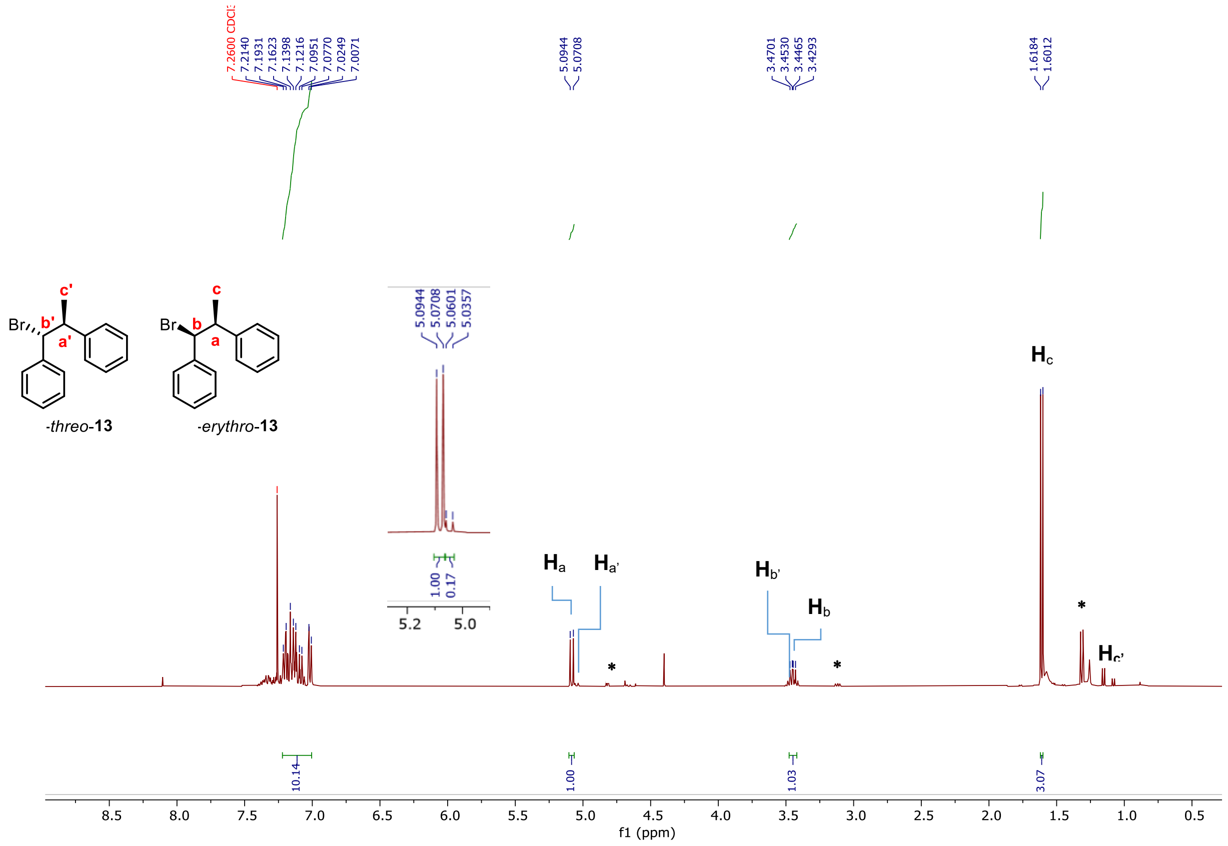

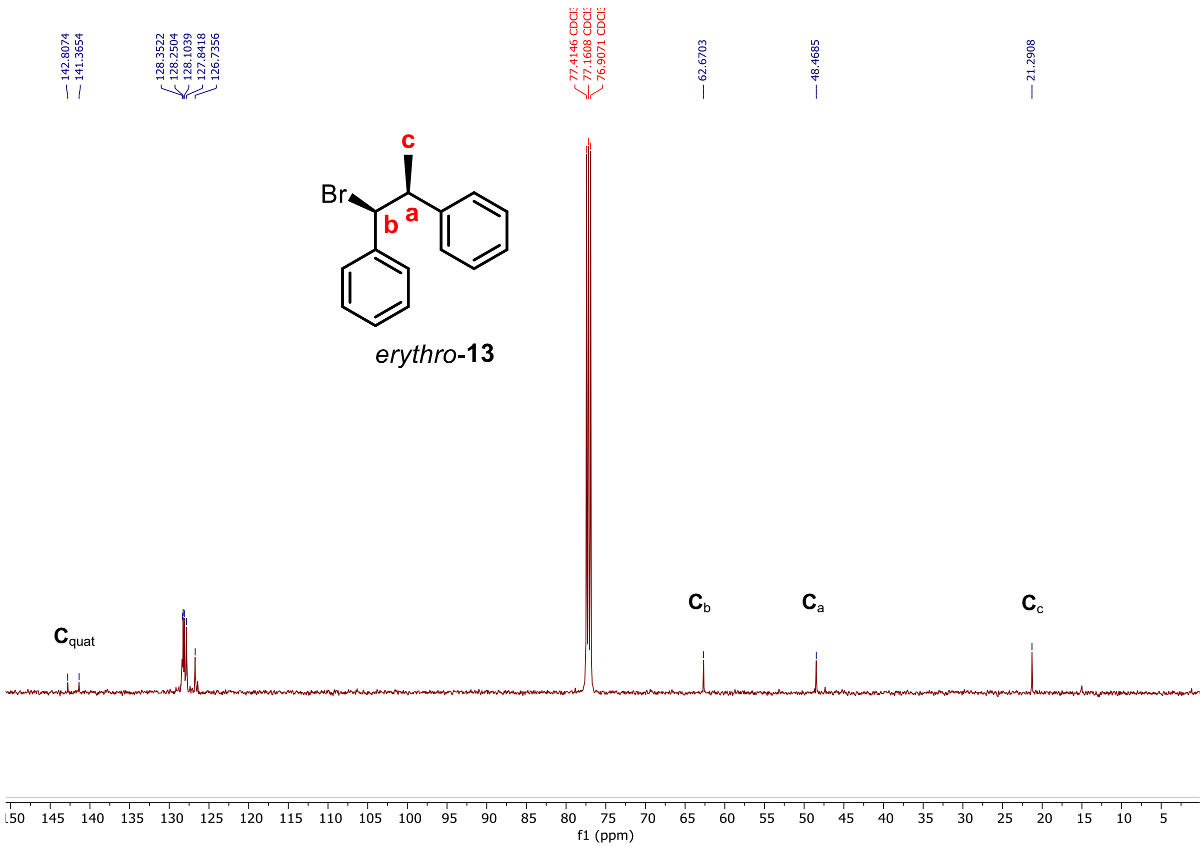

Figure S64. NMR spectra of *erythro*-**13** purified by preparative TLC (85% pure). *Top*: ^{1}H NMR (400 MHz); *Bottom*: ^{13}C NMR (100 MHz).

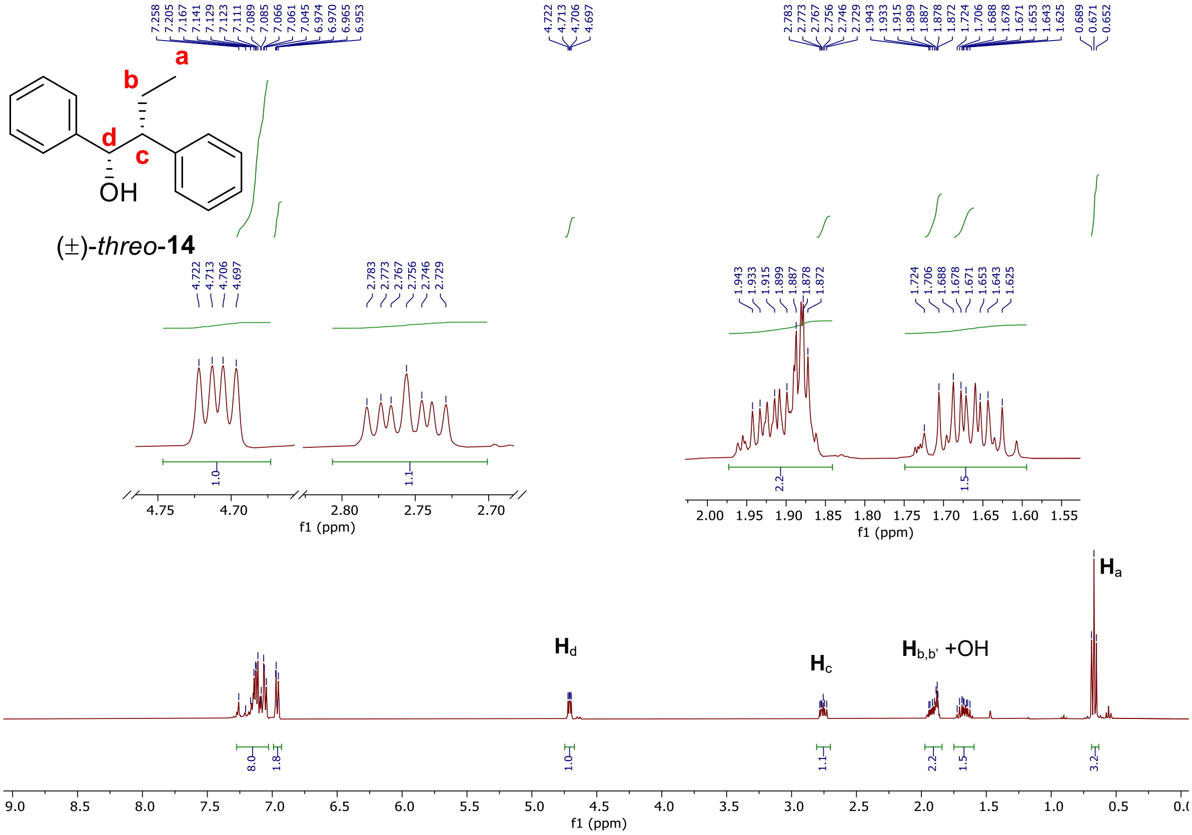

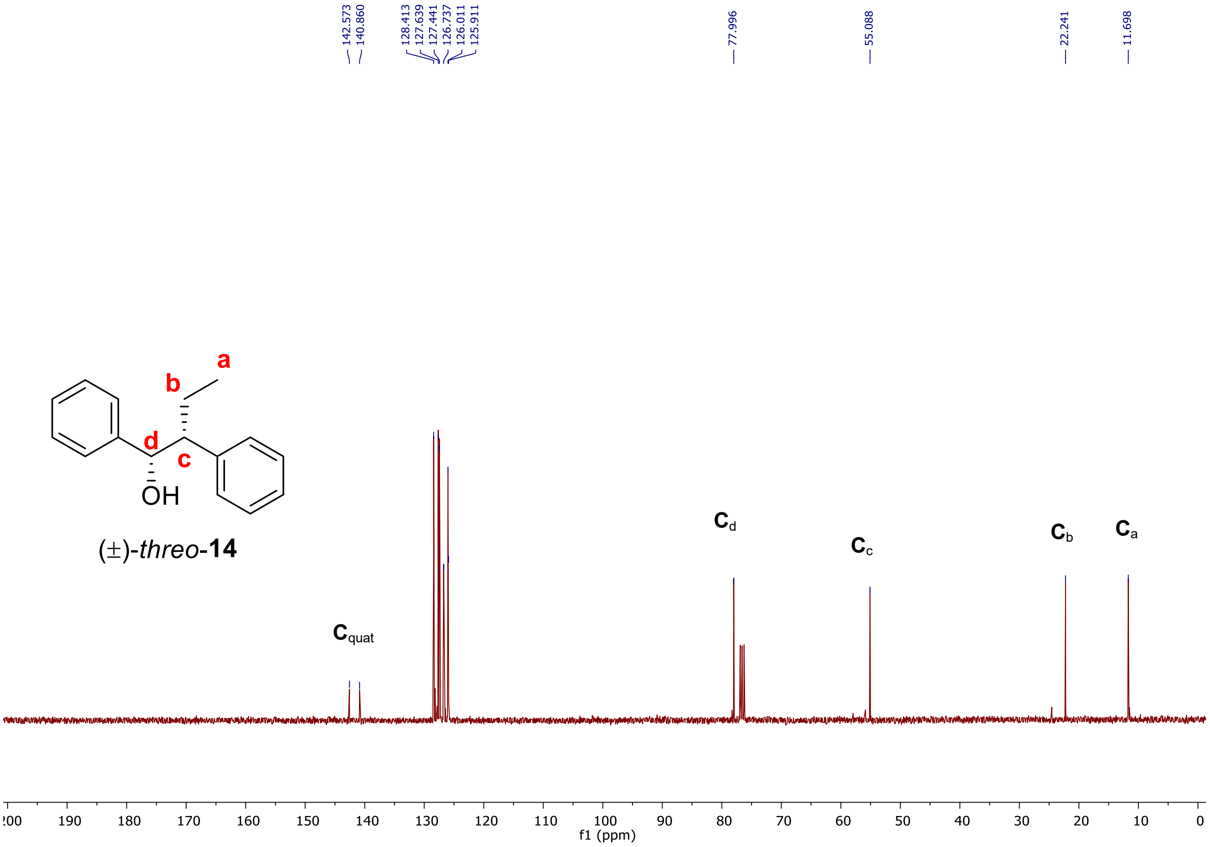

Figure S65. NMR data of (±)-*threo*-1,2,-diphenyl-1-butanol, (±)-*threo*-**14**. *Top*: ^{1}H NMR (400 MHz, $CDCl_3$). *Bottom*: ^{13}C NMR (100 MHz, $CDCl_3$)

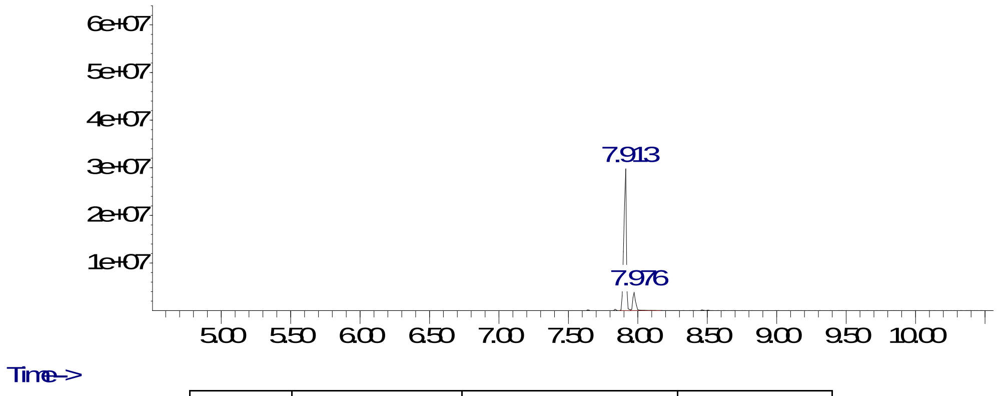

Entry	Time (min)	Identification	% of total
1	7.913	(±)-*threo*-**14**	90.45
2	7.976	(±)-*erythro*-**14**	9.55

Figure S66. GC-MS chromatogram presenting the *dr* between (±)-*threo*- and (±)-*erythro*-**14**.

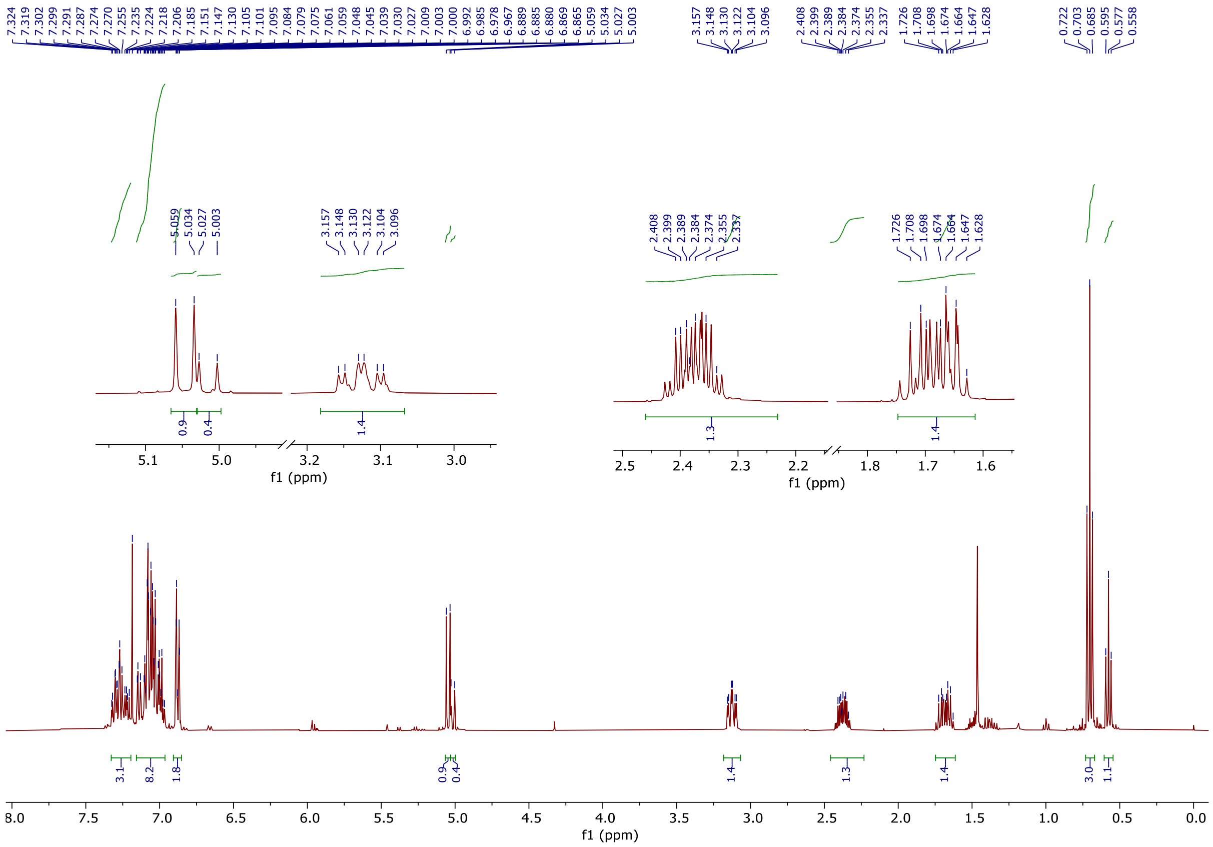

Figure S67. ^{1}H NMR spectrum (400 MHz, $CDCl_3$) of (±)-*threo* and (±)-*erythro*-1,2,-diphenyl-1-bromobutane, (±)-*threo*-**15** and (±)-*erythro*-**15** (*dr* 3:1).

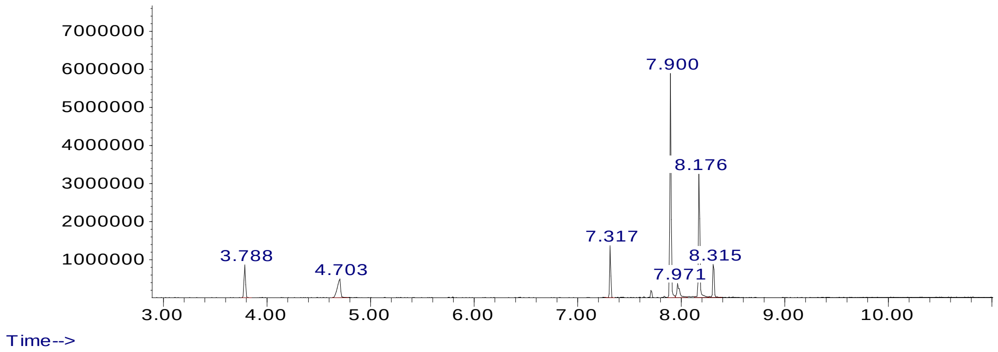

Entry	Time (min)	Identification	% of total
1	3.788	Unknown impurity	7.14
2	4.703	succinimide	8.49
3	7.317	Elimination product	8.30
4	7.900	(±)-*threo*-**14**	40.92
5	7.971	(±)-*erythro*-**14**	3.46
6	8.176	(±)-*threo*-**15**	24.74
7	8.315	(±)-*erythro*-**15**	6.95

Figure S68. GC-MS chromatogram of the crude reaction containing (±)-*threo*-**15** and (±)-*erythro*-**15** (*dr* 3:1).

8. Miscellaneous experiments in the bromination reaction of (±)-*erythro*-**6**.

8.1 Reaction with butylated hydroxyl toluene (BHT) as a radical scavenger.

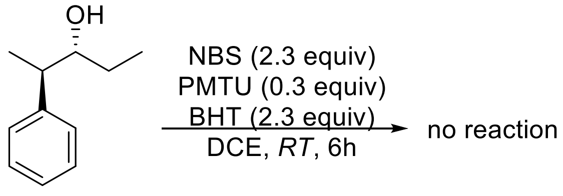

To an oven-dried 10 mL round bottom flask filled with dichloroethane (DCE) (2 mL), solution of (±)-*erythro*-**6** (50 mg, 0.30 mmol) under nitrogen atmosphere, and PMTU (12.1 mg, 0.09 mmol) was added, and the mixture was stirred at *RT* until PMTU was completely dissolved. Next, BHT (155 mg, 0.70 mmol) was added, and the mixture was stirred at *RT* until BHT was completely dissolved. Then, NBS (125 mg, 0.70 mmol) was added in two portions to the reaction mixture. The first portion of NBS (63 mg) was added to the reaction mixture and allowed to stir for 30 min. at *RT*, and then, the second portion of NBS (62 mg) was introduced to the reaction mixture and stirred at *RT* for 6 hours. After 6 hours, the reaction mixture was quenched with saturated aqueous ammonium chloride solution and extracted with DCE (3×3 mL). The organic extracts were combined, dried over anhydrous sodium sulfate and concentrated.

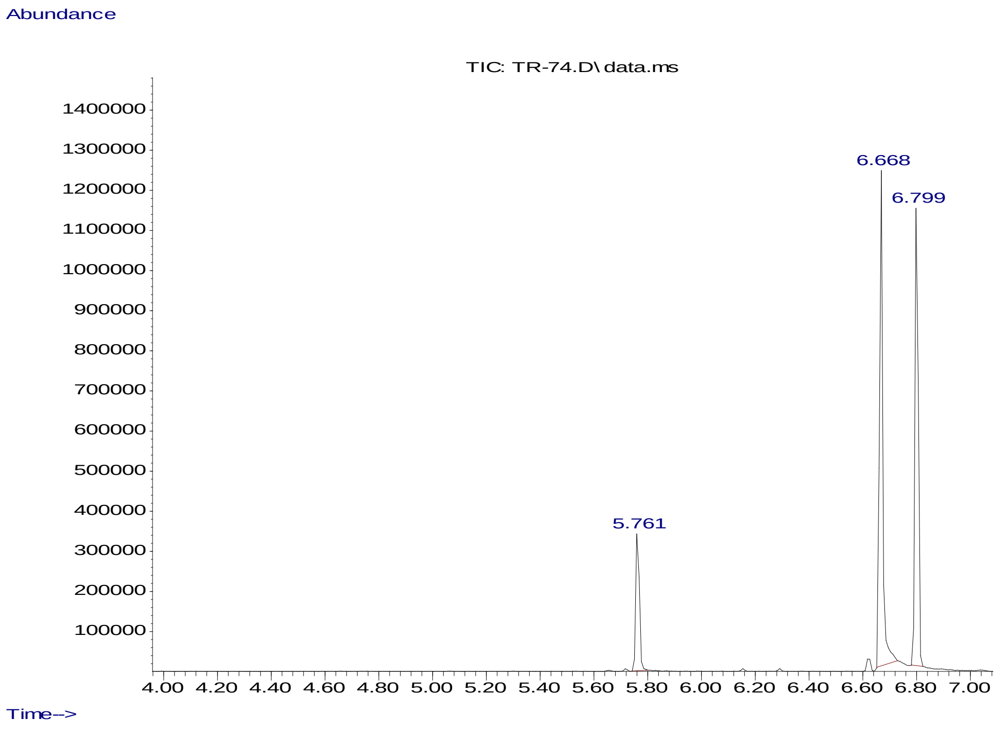

Entry	Time (min)	Identification	% of total
1	5.761	(±)-*erythro*-**6**	12.8
2	6.668	BHT radical	44.7
3	6.799	BHT	42.5

Figure S69. GC-MS chromatogram of the crude reaction containing (±)-*erythro*-**6** and BHT. The fact that neither the bromination products (±)-*erythro*-**7** and (±)-*erythro*-**8** nor their elimination products were found indicates that the radical bromination reaction did not occur at all.

8.2 Reaction without thiourea

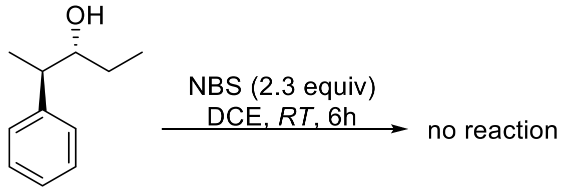

(±)-*erythro*-**6**

To an oven-dried 10 mL round bottom flask filled with dichloroethane (DCE) (2 mL), solution of (±)-*erythro*-**6** (50 mg, 0.30 mmol) under nitrogen atmosphere, and NBS (125 mg, 0.70 mmol) was added in two portions to the reaction mixture. The first portion of NBS (63 mg) was added to the reaction mixture and allowed to stir for 30 min. at *RT*, and then, the second portion of NBS (62 mg) was introduced to the reaction mixture and stirred at *RT* for 6 hours. After 6 hours, the reaction mixture was quenched with saturated aqueous ammonium chloride solution and extracted with DCE (3×3 mL). The organic extracts were combined, dried over anhydrous sodium sulfate and concentrated.

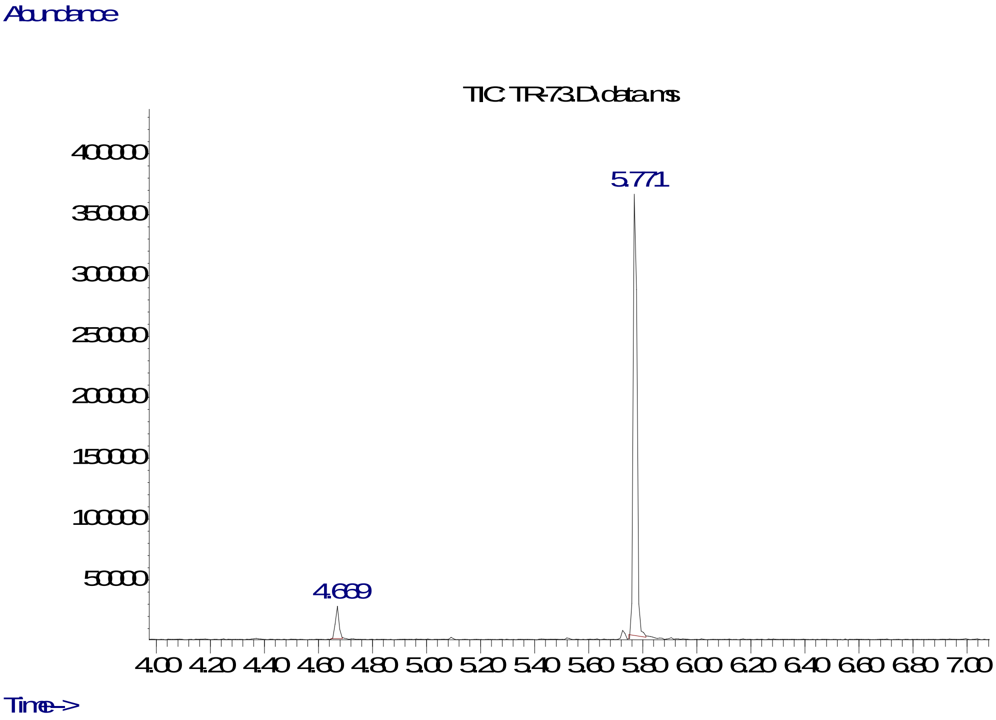

Entry	Time (min)	Identification	% of total
1	4.669	succinimide	6.1
2	5.761	(±)-*erythro*-**6**	93.9

Figure S70. GC-MS chromatogram of the crude reaction containing (±)-*erythro*-**6** and succinimide. The fact that neither the brominated products (±)-*erythro*-**7** and (±)-*erythro*-**8** nor their elimination products were found proves that radical bromination is suppressed if thioureas do not take place in this type of transformation.

8.3 Reaction with molecular bromine (Br_2) as a different bromine donor with or without thiourea.

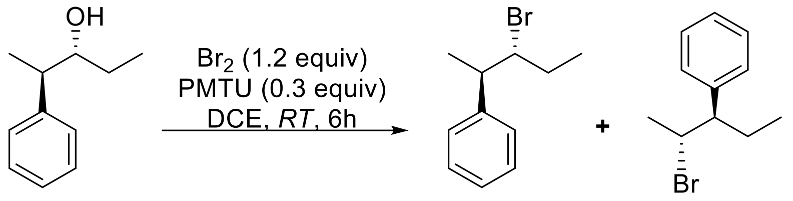

(±)-*erythro*-**6** (±)-*erythro*-**7** (±)-*erythro*-**8**

To an oven-dried 10 mL round bottom flask filled with dichloroethane (DCE) (2 mL), solution of (±)-*erythro*-**6** (50 mg, 0.30 mmol) under nitrogen atmosphere, and PMTU (12.1 mg, 0.09 mmol) was added, and the mixture was stirred at *RT* until PMTU was completely dissolved. Next, Br_2 (18 µL, 0.36 mmol) was added, and the mixture was stirred at *RT* for 6 hours. After 6 hours, the reaction mixture was quenched with saturated aqueous ammonium chloride solution and extracted with DCM (3×3 mL). The organic extracts were combined, dried over anhydrous sodium sulfate and concentrated.

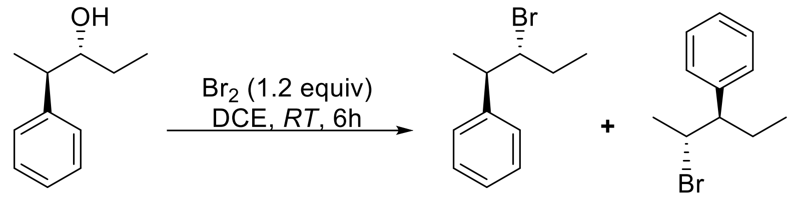

(±)-*erythro*-**6** (±)-*erythro*-**7** (±)-*erythro*-**8**

To an oven-dried 10 mL round bottom flask filled with dichloroethane (DCE) (2 mL), solution of (±)-*erythro*-**6** (50 mg, 0.30 mmol) under nitrogen atmosphere, and Br_2 (18 μL, 0.36 mmol) was added, and the mixture was stirred at *RT* for 6 hours. Then, the reaction mixture was quenched with saturated aqueous ammonium chloride solution and extracted with DCM (3×5 mL). The organic extracts were combined, dried over anhydrous sodium sulfate and concentrated.

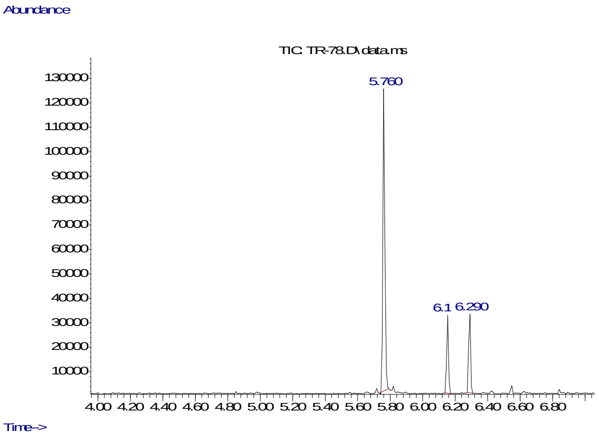

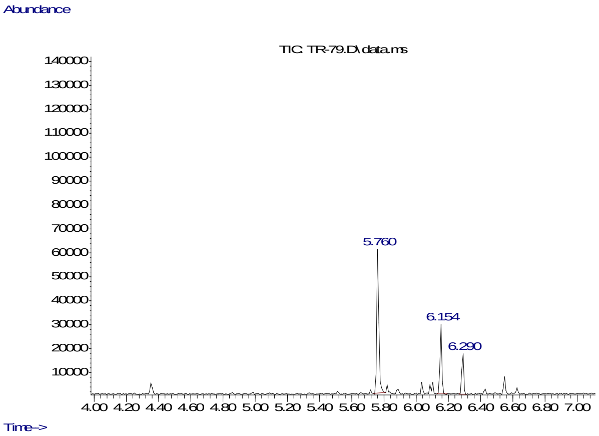

Entry	Time (min)	Identification	% of total
1	5.760	(±)-*erythro*-**6**	66.4
2	6.154	(±)-*erythro*-**7**	15.7
3	6.290	(±)-*erythro*-**8**	17.9

Entry	Time (min)	Identification	% of total
1	5.760	(±)-*erythro*-**6**	58.3
2	6.154	(±)-*erythro*-**7**	25.0
3	6.290	(±)-*erythro*-**8**	16.7

Figure S71. GC-MS chromatograms of the crude reaction containing (±)-*erythro*-**6** and Br_2 with (*left)* or without (*right*) PMTU. The fact that both brominated products (±)-*erythro*-**7** and (±)-*erythro*-**8** are present in the crude reaction indicates that both procedures followed radical bromination.

8.4 Reaction with Br_2 and BHT.

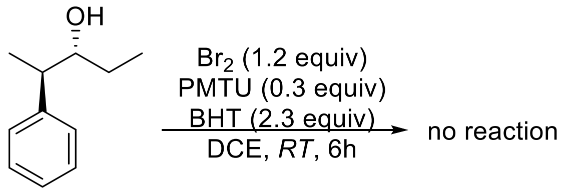

(±)-*erythro*-**6**

To an oven-dried 10 mL round bottom flask filled with dichloroethane (DCE) (2 mL), solution of (±)-*erythro*-**6** (50 mg, 0.30 mmol) under nitrogen atmosphere, and PMTU (12.1 mg, 0.09 mmol) was added, and the mixture was stirred at *RT* until PMTU was completely dissolved. Next, BHT (155 mg, 0.70 mmol) was added, and the mixture was stirred at *RT* until BHT was completely dissolved. Then, Br_2 (0.018 mL, 0.36 mmol) was added to the reaction mixture and

stirred at *RT* for 6 hours. Then, the reaction mixture was quenched with saturated aqueous ammonium chloride solution and extracted with DCE (3×5 mL). The organic extracts were combined, dried over anhydrous sodium sulfate and concentrated.

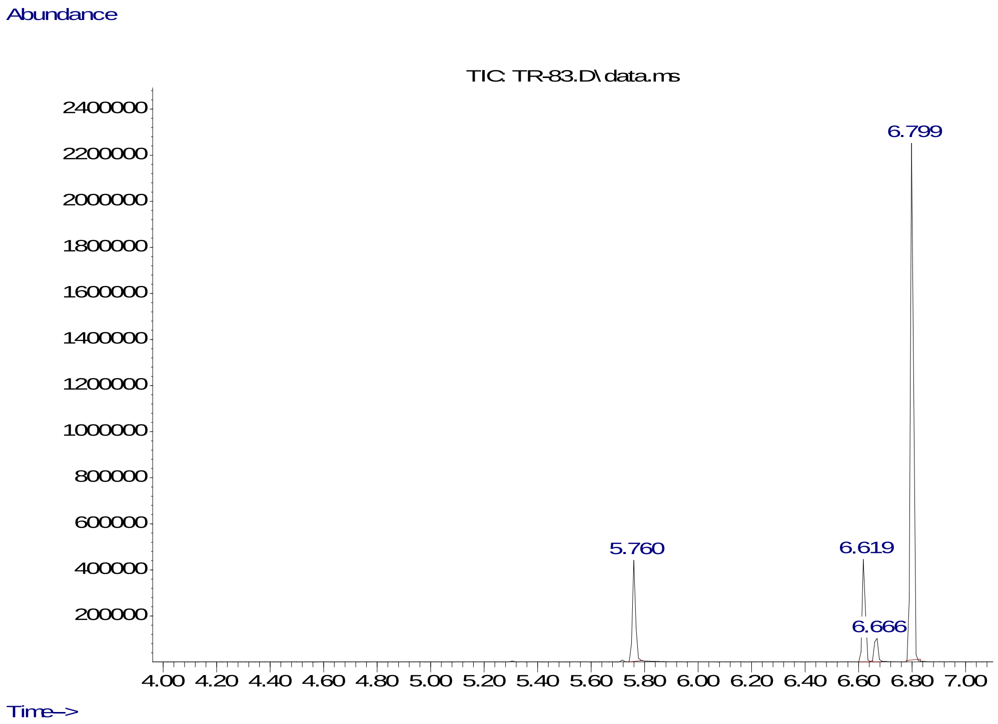

Entry	Time (min)	Identification	% of total
1	5.760	(±)-*erythro*-**6**	12.4
2	6.619	unidentified	12.7
3	6.666	BHT radical	3.5
4	6.799	BHT	71.4

Figure S72. GC-MS chromatogram of the crude reaction following the bromination of (±)-*erythro*-**6** with Br_2, PMTU and BHT. The fact that neither the bromination products (±)-*erythro*-**7** and (±)-*erythro*-**8** nor their elimination products were found indicates that the radical bromination reaction did not occur.

9. Bromination reactions of β-aryl propanols.

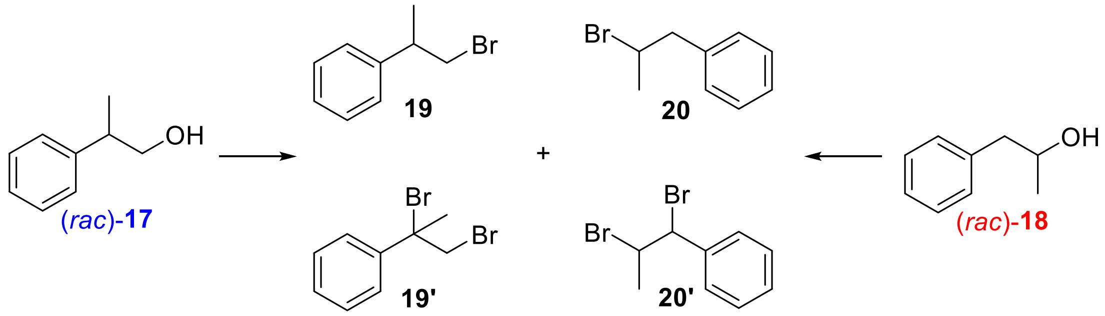

Scheme S11. Bromination *rac*-**17** and *rac*-**18**.

9.1 Reaction of (*rac*)-2-phenyl-1-propanol (*rac*-**17**) with NBS and PMTU

To an oven-dried 10 mL round bottom flask with dichloroethane (DCE) (2 mL), *rac*-**17** (50 mg, 0.37 mmol), and PMTU (14.6 mg, 0.11 mmol) were added, and the mixture was stirred at *RT*

until PMTU was completely dissolved. Next, NBS (150 mg, 0.84 mmol) was added in two portions to the reaction mixture. The first portion of NBS (75 mg) was allowed to stir for 30 min. at *RT*, and then, the second portion of NBS (75 mg) was introduced and stirred at *RT* for 6 hours. Then, the reaction mixture was quenched with saturated aqueous ammonium chloride solution and extracted with DCM (3×5 mL). The organic extracts were combined, dried over anhydrous sodium sulfate, and concentrated.

9.2 Reaction of (*rac*)-1-phenyl-2-propanol (*rac*-**18**) with NBS and PMTU.

To an oven-dried 10 mL round bottom flask with dichloroethane (DCE) (2 mL), *rac*-**18** (50 mg, 0.37 mmol), and PMTU (14.6 mg, 0.11 mmol) were added, and the mixture was stirred at RT until PMTU was completely dissolved. Next, NBS (150 mg, 0.84 mmol) was added in two portions to the reaction mixture. The first portion of NBS (75 mg) was allowed to stir for 30 min. at RT, and then, the second portion of NBS (75 mg) was introduced and stirred at RT for 6 hours. Then, the reaction mixture was quenched with saturated aqueous ammonium chloride solution and extracted with DCM (3×5 mL). The organic extracts were combined, dried over anhydrous sodium sulfate, and concentrated.

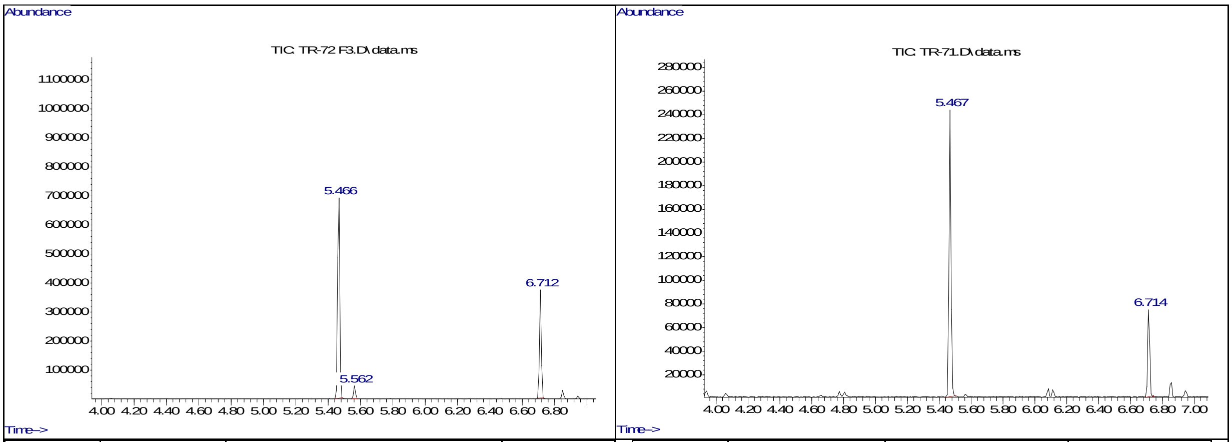

Entry	Time (min)	Identification	% of total
1	5.467	Compound **20**	64.9
2	5.562	Compound **19**	3.5
3	6.714	Dibromo-Ph-migration product	29.3
4	6.850	Direct dibromination product	2.3

Entry	Time (min)	Identification	% of total
1	5.467	Compound **20**	75.7
2	6.714	dibromide	24.3

Figure S73. GC-MS chromatogram of the crude reaction mixture after work-up following bromination of *rac*-**17** (*left*) and *rac*-**18** (*right*) with NBS and PMTU .

9.3 NMR data of compounds **20** and **20'**.

To obtain a more clear NMR spectrum, the mixture was cleaned by prep-TLC (1:99 ethyl acetate:hexane). The process enriched the debromination byproduct **20'** and provided a clearer NMR spectrum for both compounds. Compound **20** is a known literature compound, and its spectrum matches the literature. (compound **2g** in: Yan-Ling Liu, Xian-Chen He, Jie Gao, Ke-Rong Li, Kai Chen, Hao-Yue Xiang, and Hua Yang, *The Journal of Organic Chemistry* **2024**, *89*, 10987-10997. DOI: 10.1021/acs.joc.4c01422)

Compound **20**

^{1}H NMR (400 MHz, CDCl3): δ 7.38 – 7.19 (m, 5H), 4.32 (m, H_c, 1H), 3.23 (dd, ^2J = 14 Hz, ^3J = 6.7 Hz, H_a, 1H), 3.07 (dd, ^2J = 14 Hz, ^3J = 7.2 Hz, H_b, 1H), 1.70 (d, ^3J = 6.4 Hz, H_d, 3H) ppm.

Compound **20'**

^{1}H NMR (400 MHz, CDCl3): δ 7.38 – 7.19 (m, 5H), 5.04 (d, ^3J = 10 Hz, $H_{a'}$, 1H), 4.60 (m, $H_{b'}$, 1H), 2.04 (d, ^3J = 6.4 Hz, $H_{c'}$, 3H) ppm.

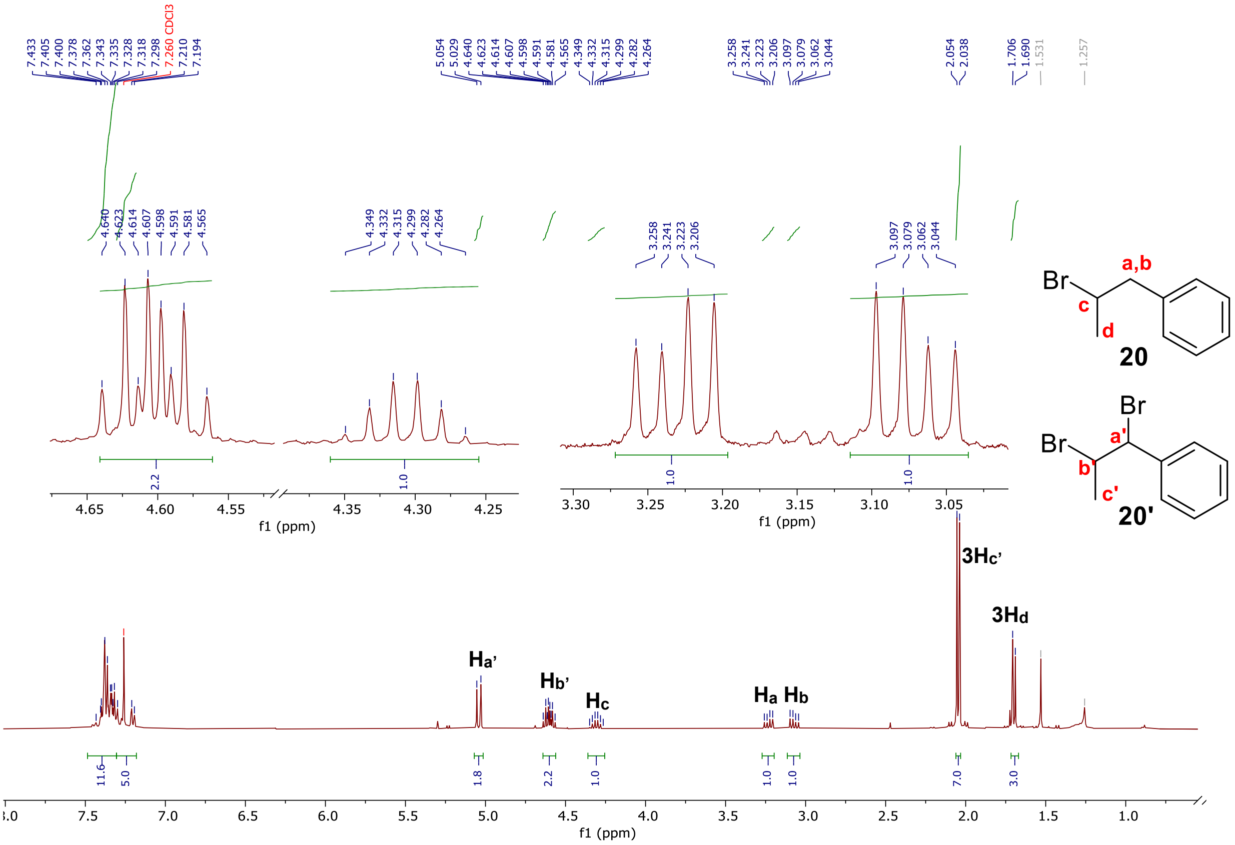

Figure S74. ^{1}H NMR data (400 MHz, $CDCl_3$) of compounds **20** and **20'** following bromination of substrate **17**.

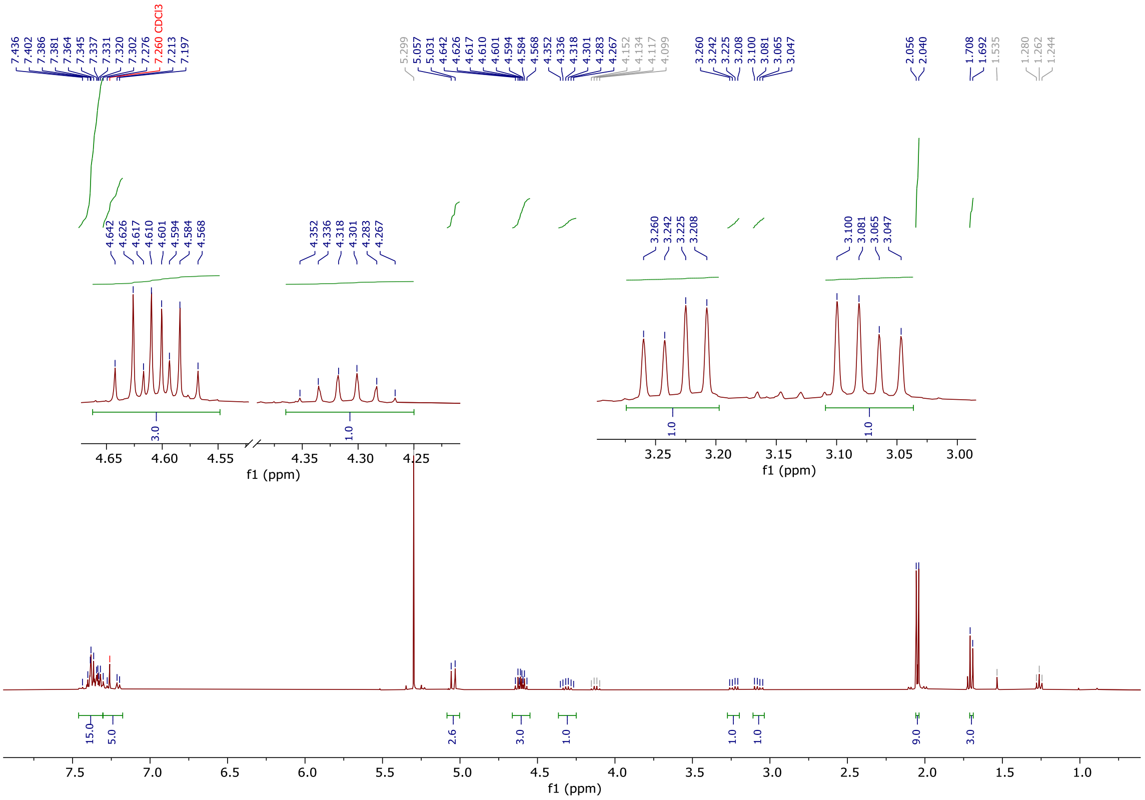

Figure S75. ^{1}H NMR data (400 MHz, $CDCl_3$) of compounds **20** and **20'** following bromination of substrate **18**.

10. Enantiospecificity in bromination of enantiomeric enriched 1-phenyl-2-propanol, 18.

10.1 Mosher ester of (*rac*)-1-phenyl-2-propanol (*rac*-18).

To a solution of (*rac*)-**18** (200 mg, 1.47 mmol), in freshly distilled THF (20 mL) was added S-(–)-(α)-MTPA (585 mg, 2.50 mmol). The mixture was homogenized, and then DCC (515 mg, 2.5 mmol) and DMAP (25 mg, 0.2 mmol) were added successively, and the reaction mixture was stirred at RT for 3 h. Upon completion consumption of the starting material, the solvent was removed, and the crude diastereomers were purified by preparative TLC using 10% ethyl acetate in hexane as eluent to afford the two diastereomeric products as colorless oil in 67% overall yield.

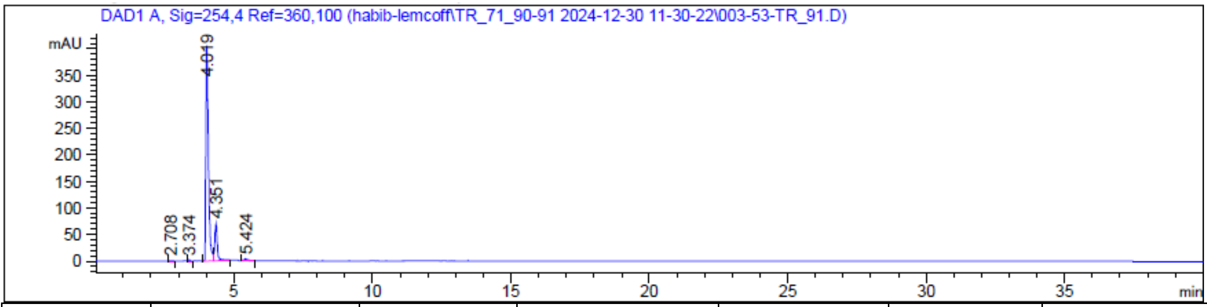

Peak (#)	Retention time (min)	Integration notation	Width (mAU*s)	Area (mAU)	Hight	Area (%)
**1**	4.019	BV	0.0952	2546.28491	405.65698	85.8
**2**	4.351	VB	0.0898	429.15518	69.69215	14.2

Figure S76. HPLC chromatogram of Mosher ester (isolated diastereomer by preparative TLC).

10.2 Hydrolysis of Mosher ester to afford enantiomerically enriched **18**

To a solution of the Mosher ester (100 mg, 0.28 mmol), in DCM (1.2 mL), a methanolic solution of 50% KOH was added, and the mixture was stirred at *RT* for 3 h. Upon completion consumption of the starting material, the solvent was removed, and the product was purified by preparative TLC using 10% ethyl acetate in hexane as eluent to afford the product as a colorless oil in 42% yield.

A.

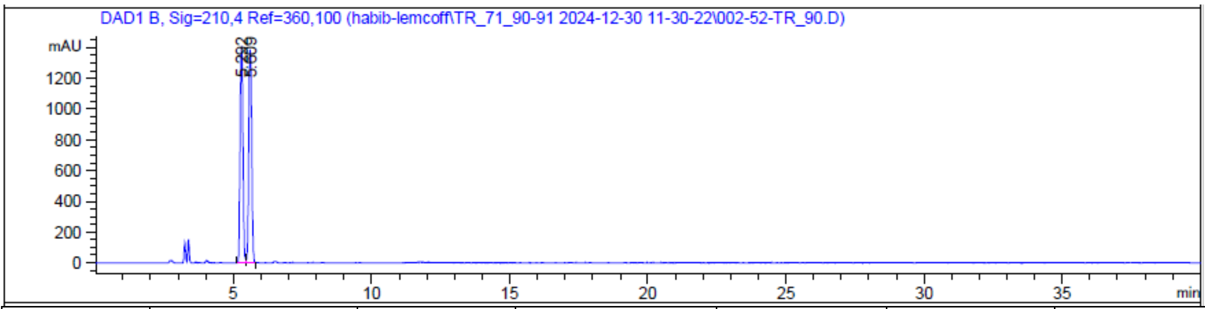

Peak (#)	Retention time (min)	Integration notation	Width (mAU*s)	Area (mAU)	Hight	Area (%)
**1**	5.292	BV	0.1197	1.02946e4	1401.58923	49.3
**2**	5.609	VV	0.1251	1.05922e4	1387.67896	50.7

B.

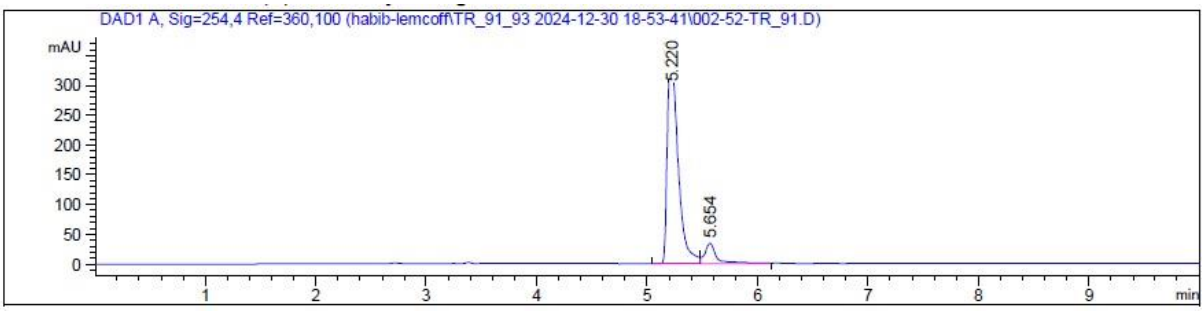

Peak (#)	Retention time (min)	Integration notation	Width (mAU*s)	Area (mAU)	Hight	Area (%)
**1**	5.220	BV	0.1010	2378.36719	360.53592	90.5
**2**	5.654	VB	0.1035	248.48103	33.91032	9.5

Figure S77. HPLC chromatogram of (*rac*)-18 (**A**) and enantiomerically enriched **18** after alkaline hydrolysis of Mosher ester (**B**).

10.3 Bromination of enantiomerically enriched **18**

The general procedure used in section 9.2 was applied in the bromination of enantiomerically enriched **18** to afford a single enantiomerically enriched **20**.

A.

Peak (#)	Retention time (min)	Integration notation	Width (mAU*s)	Area (mAU)	Hight	Area (%)
**1**	4.062	BV	0.0778	6016.90723	1173.76453	56.8
**2**	4.396	VB	0.0665	4575.07178	1057.64465	43.2

B.

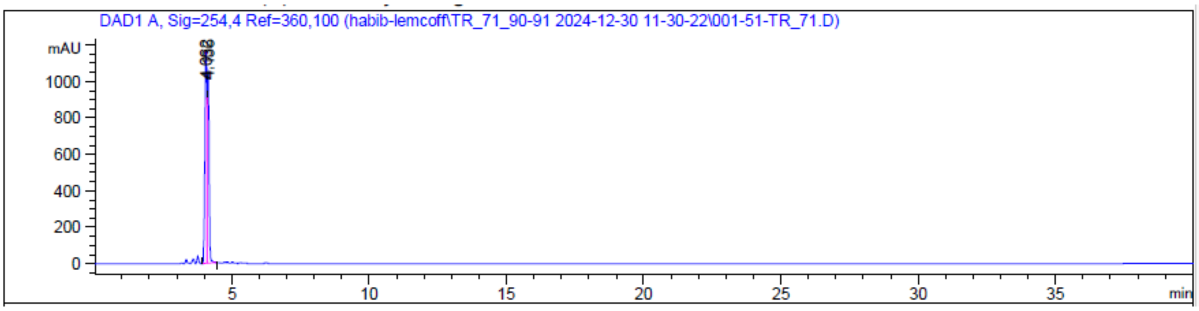

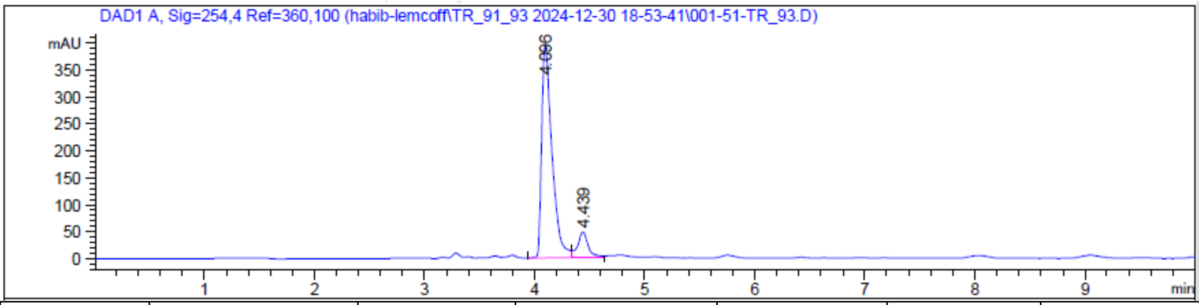

Peak (#)	Retention time (min)	Integration notation	Width (mAU*s)	Area (mAU)	Hight	Area (%)
**1**	4.096	BV	0.0979	2570.43530	395.32852	88.9
**2**	4.439	VV	0.0980	320.19815	47.87792	11.1

Figure S78. HPLC chromatogram of (*rac*)-**20** (**A**) and enantiomerically enriched **20** following bromination of enantiomerically enriched **18** (**B**).
